# Supplementary material for: The chemistry of branched condensed phosphates
Source: Nat Commun. 2021 Sep 10;12:5368. doi: 10.1038/s41467-021-25668-3 (PMC8433156; doi:10.1038/s41467-021-25668-3)
Supplement: Supplementary file 1 — Supplementary Info [file 41467_2021_25668_MOESM1_ESM.pdf]

Supplementary Information

The Chemistry of Branched Condensed Phosphates

Tobias Dürr-Mayer[1], Danye Qiu[1], Verena B. Eisenbeis[1], Nicole Steck[1], Markus Häner[1], Alexandre Hofer[2], Andreas Mayer[3], Jay S. Siegel[4,5], Kim K. Baldridge[4] and Henning J. Jessen[1,5,6]

[1] Institute of Organic Chemistry, University of Freiburg, Albertstrasse 21, 79104 Freiburg, Germany

[2] Department of Chemistry, University of Cambridge, Lensfield Road, Cambridge CB2 1EW, United Kingdom

[3] Département de Biochimie, Université de Lausanne, 1015 Epalinges, Switzerland

[4] Health Science Platform, Tianjin University, Nankai District, Tianjin, PRC 30072

[5] Freiburg Research Institute for Advanced Studies, University of Freiburg, Albertstrasse 21, 79104 Freiburg, Germany

[6] Cluster of Excellence livMatS @ FIT – Freiburg Center for Interactive Materials and Bioinspired Technologies, University of Freiburg, Georges-Köhler-Allee 105, 79110 Freiburg, Germany

Contents

Abbreviations 1
Supplementary Notes 2
  Supplementary Note 1. Experimental procedures 2
  Supplementary Note 2. Decomposition of singly and twofold modified ultraphosphates 4
  Supplementary Note 3. Salt metatheses on ultraphosphates 5
  Supplementary Note 4. X-ray structure of tris(9*H*-fluoren-9-yl)methyl ultraphosphate 6
  Supplementary Note 5. Stability of ultraphosphates in aqueous media 7
  Supplementary Note 6. Gel electrophoresis of ultraphosphates 8
  Supplementary Note 7. Polyphosphate analysis of yeast cell extracts 10
  Supplementary Note 8. Enzymatic digestion of ultraphosphates 12
  Supplementary Note 9. Ultraphosphate rearrangement 15
    Supplementary Note 9.1. Experimental results 15
    Supplementary Note 9.2. Computational results 17
  Supplementary Note 10. Reactivity of uP_4 as phosphorylating agent 23
Supplementary Methods 24
  Supplementary Methods 1. Monophosphate syntheses 24
  Supplementary Methods 2. Determination of purities and yields of ultraphosphates 26
  Supplementary Methods 3. Ultraphosphate syntheses 26
X-ray crystallography data 49
NMR-spectra 57
Mass spectrometry data 161
Raman spectra 195
Supplementary References 200

Abbreviations

A	ampere or adenine
Å	angstrom
AcOEt	ethyl acetate
AIEX	anion exchange
AMP	adenosine-5′-monophosphate
APS	ammonium persulfate
AU	absorbance unit
BArF	tetrakis[3,5-bis(trifluoromethyl)phenyl]borate
CE	capillary electrophoresis
DBU	1,8-diazabicyclo[5.4.0]undec-7-ene
DCI	4,5-dicyanoimidazole
DEA	denitrifying enzyme activity
DIPEA	diisopropylethylamine
DEACM	7-(diethylamino)-4-(hydroxymethyl)-coumarine
DMF	*N,N*-dimethylformamide
DMSO	dimethylsulfoxide
EDTA	ethylenediaminetetraacetic acid
eq.	equivalent
ESI	electrospray ionization
et al.	lat. *et alii, et aliae, et alia;* and others
Et_2O	diethyl ether
ETT	5-(ethylthio)-1*H*-tetrazole
FAM	carboxyfluorescein
Fm	(9*H*-fluoren-9-yl)methyl
Fmoc	fluorenylmethoxycarbonyl
HPLC	high performance liquid chromatography
HRMS	high resolution mass spectrometry
Hz	Hertz
in vacuo	under vacuum
K	Kelvin
λ	wavelength
m	multiplet (NMR), milli
M	molar
*m*CPBA	*meta*-chloroperoxybenzoic acid
MeCN	acetonitrile
MPLC	medium pressure liquid chromatography
MS	mass spectrometry
NMR	nuclear magnetic resonance
OGD	orange G dye
PAGE	polyacrylamide gel electrophoresis
PEG	polyethylene glycol
$P(NEt_2)_3$	tris(diethylamine)phosphine
PNPP	*para*-nitrophenyl phosphate
polyP	polyphosphate(s)
ppm	parts per million
PPN	(bis(triphenylphosphine)iminium)
room temp.	room temperature
RP	reversed-phase
SAX	strong anion exchange
TBA	tetrabutylammonium
TBE	Tris-borate-EDTA
TEAA	triethylammonium acetate
TEMED	*N*,*N*,*N*′,*N*′-tetramethylethylenediamine
Tf	trifluoromethanesulfonyl
THF	tetrahydrofuran
uP	ultraphosphate
UV	ultra-violet
V	voltage
YAG	yttrium aluminium garnet

Supplementary Notes

Supplementary Note 1. Experimental procedures

General methods

Reactions were performed under exclusion of air and moisture in oven-dried glassware under dry nitrogen or argon atmosphere. Reagents were purchased from commercial suppliers (Sigma Aldrich, Acros, TCI, Roth, ChemPur, Alfa Aesar, VWR/Merck) and used as received unless noted otherwise. Air-and moisture-sensitive liquids and solutions were transferred via syringe. Bases were distilled prior to use.

Solvents

Solvents were provided in analytical grade from the technical service of the University of Freiburg, institute of organic chemistry. Dry solvents were purified using *Braun Solvent Purification System 800* and stored under molecular sieves under dry argon atmosphere.
Deuterated solvents for NMR and reactions were obtained from Armar Chemicals, Switzerland, Deutero, Germany and Euriso-top, Germany, in the indicated purity grade and used as received for NMR spectroscopy.

Enzymes

Alkaline phosphatase from bovine intestinal mucosa was purchased from Sigma-Aldrich as lyophilized powder (≥10 DEA units/mg solid) and stored at −20°C.

Ion exchange chromatography

Anion exchange chromatography was performed using an automated ÄKTA™ pure system and DEAE-Sepharose® Fast Flow or Q Sepharose® Fast Flow (Sigma-Aldrich). Crude products were loaded as aqueous solutions and eluted using increasing concentrations of either NaCl, LiCl, NH_4HCO_3 or $NaClO_4$ solutions.

Cation exchange

For the preparation of TBA salts, Dowex 50WX8 H^+ form was used followed by neutralization with TBA hydroxide and subsequent lyophilization.

Lyophilization

Lyophilization was performed using Alpha 1-4 LDplus and Alpha 1-2 LDplus from Christ.

NMR spectroscopy

^{1}H-NMR spectra were measured on Bruker Avance III HD 300 MHz, Bruker Avance Neo 400 MHz (with cryoprobe) and Bruker Avance III HD 500 MHz spectrometers in the indicated deuterated solvents. All signals are referred to an internal solvent signal standard ($CHCl_3$: δ = 7.26 ppm, DHO: δ = 4.79 ppm, DMF-d_6: δ = 2.92 ppm, CD_2HCN: δ = 1.94 ppm). ^{13}C-NMR spectra were measured with ^{1}H-broad band decoupling on Bruker 101 MHz (with cryoprobe) spectrometer. All signals were referenced to the internal solvent signal (DMF-d_7: δ = 34.9 ppm, CD_3CN: δ = 1.32 ppm). ^{31}P-NMR spectra were measured either using ^{1}H-broad band decoupling or ^{1}H-coupling mode on Bruker 121 MHz, Bruker 162 MHz (with cryoprobe) or Bruker 202 MHZ spectrometer. All signals were referenced to an external standard.

Data are reported as follows: chemical shift (δ in ppm), multiplicity (s: singlet, d: doublet, t: triplet, q: quartet, m: multiplet, br. s: broad singlet), coupling constant (*J* in Hz), integration, assignment.

High resolution mass spectrometry (HRMS)

High resolution mass spectra were recorded by C. Warth (analytical department of the university of Freiburg, institute for organic chemistry) using a Thermo LCQ Advantage (spray voltage: 2.5 – 4.0 kV, spray current: 5 µA, ion transfer tube: 250 (150)°C, evaporation temperature: 50-400°C).

Analytical HPLC

Analytical HPLC was performed with a Dionex UltiMate 3000 system of Thermo Fisher. Experiments were run on a Hypersil GOLD C_{18} column (175 Å, 3.0 µm, 150 x 3 mm) or Hypersil GOLD aQ column (175 Å, 3.0 µm, 150 x 3 mm). Products were detected at λ = 220 nm.

Preparative RP-MPLC

For preparative RP-MPLC, an automated Interchim®-system was used. The AQ-solid phase was purchased from Interchim.

CE-ESI-MS analysis

All experiments were performed on a bare-fused silica capillary with a length of 100 cm (50 µm internal diameter and 365 µm outer diameter) on an Agilent 7100 capillary electrophoresis system coupled to a Q-TOF (6520, Agilent) equipped with a commercial CE-MS adapter and sprayer kit from Agilent. 35 mM ammonium acetate titrated by ammonia solution to pH 9.7 was background electrolyte. Samples were diluted 10 times with water and injected by applying 50 mbar pressure for 10 s. For each analysis, a constant CE current of either 23 µA was established by applying 30 kV over the capillary.

The sheath liquid was composed of a water-isopropanol (1:1) mixture spiked with mass references. It was introduced at a constant flow rate of 1.5 µL/min. ESI-TOF-MS was conducted in the negative ionization mode; the capillary voltage was set to -3000 V. Automatic recalibration of each acquired spectrum was performed using reference masses of reference standards (TFA anion, $[M-H]^-$, 112.9855), and (HP-0921, $[M-H+CH_3COOH]^-$, 980.0163). Peak assignment is according to the accurate m/z. Positional isomers are assigned by spiking with relative standards.

Raman spectroscopy

FT Raman spectra (range: 4000-50 cm^{-1}, resolution: 4 cm^{-1}, room temp.) were recorded on a Bruker VERTEX 70 spectrometer equipped with a RAM II module (1064 nm exciting line of a Nd-YAG laser) by using a highly sensitive liquid nitrogen cooled Ge-detector. The samples were prepared in flame-sealed soda-lime glass Pasteur pipettes to protect them against air and moisture. The data were processed with the Bruker OPUS 7.5 software package. Raman intensities are reported as follows: vw = very weak, w = weak, m = medium, s = strong, vs = very strong.

X-ray crystallography

Data were collected on a Bruker APEX2 QUAZAR three-circle diffractometer with a microfocus sealed X-ray tube using mirror optics as monochromator and a Bruker APEXII detector. The diffractometer was equipped with an Oxford Cryostream 800 low temperature device and used MoK_α radiation (λ = 0.71073 Å). All data were integrated with SAINT and a multi-scan absorption correction using SADABS was applied.

Supplementary Note 2. Decomposition of singly and twofold modified ultraphosphates

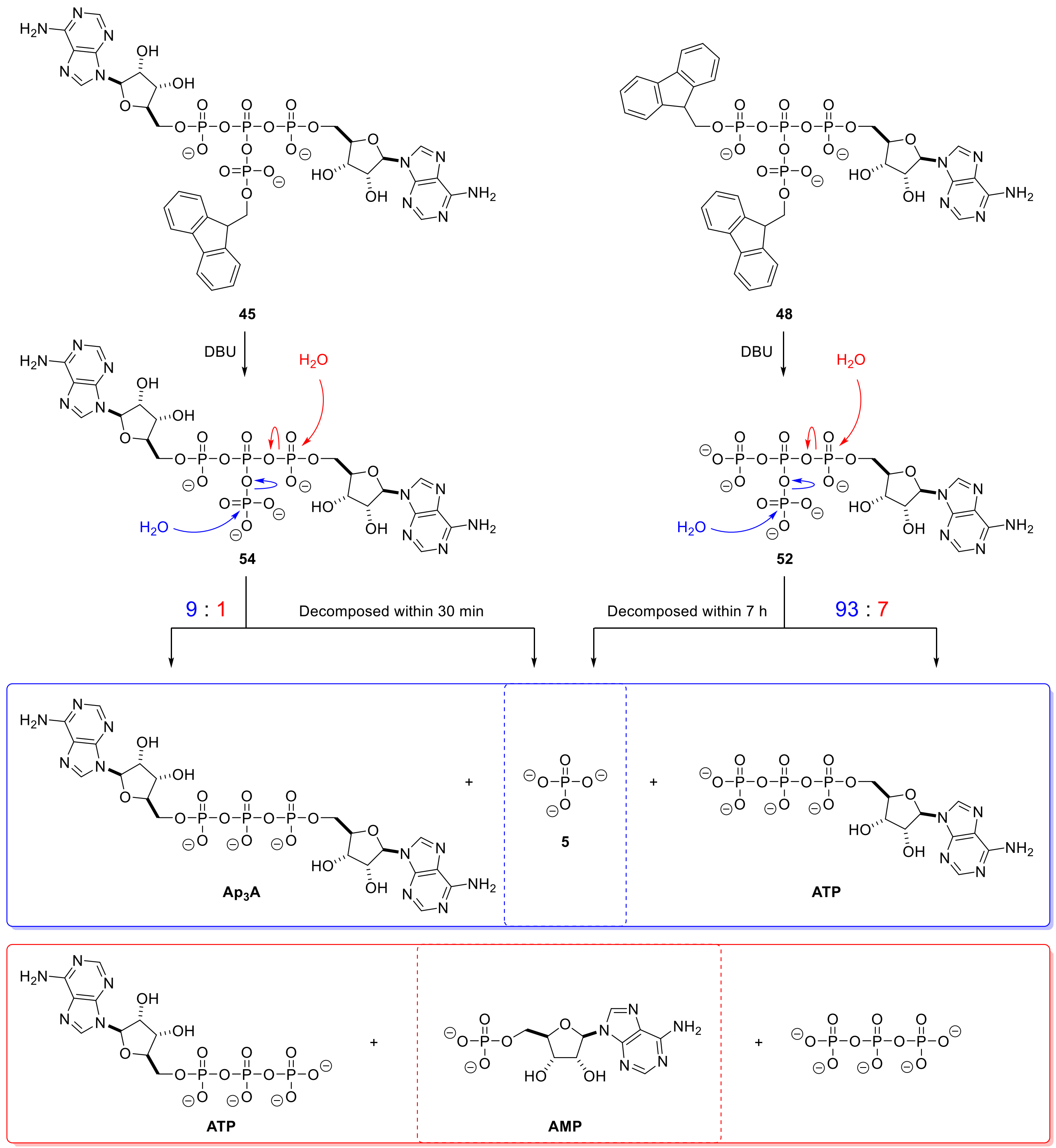

Supplementary Fig. 1│ Decomposition of singly and twofold modified ultraphosphates. The unsymmetrically Fm-modified ultraphosphates **45** and **52** were deprotected using DBU resulting in the twofold and singly adenosine-modified ultraphosphates **54** and **52**. The decomposition was monitored and the product distribution determined using $^{31}P\{^1H\}$-NMR.

Supplementary Note 3. Salt metatheses on ultraphosphates

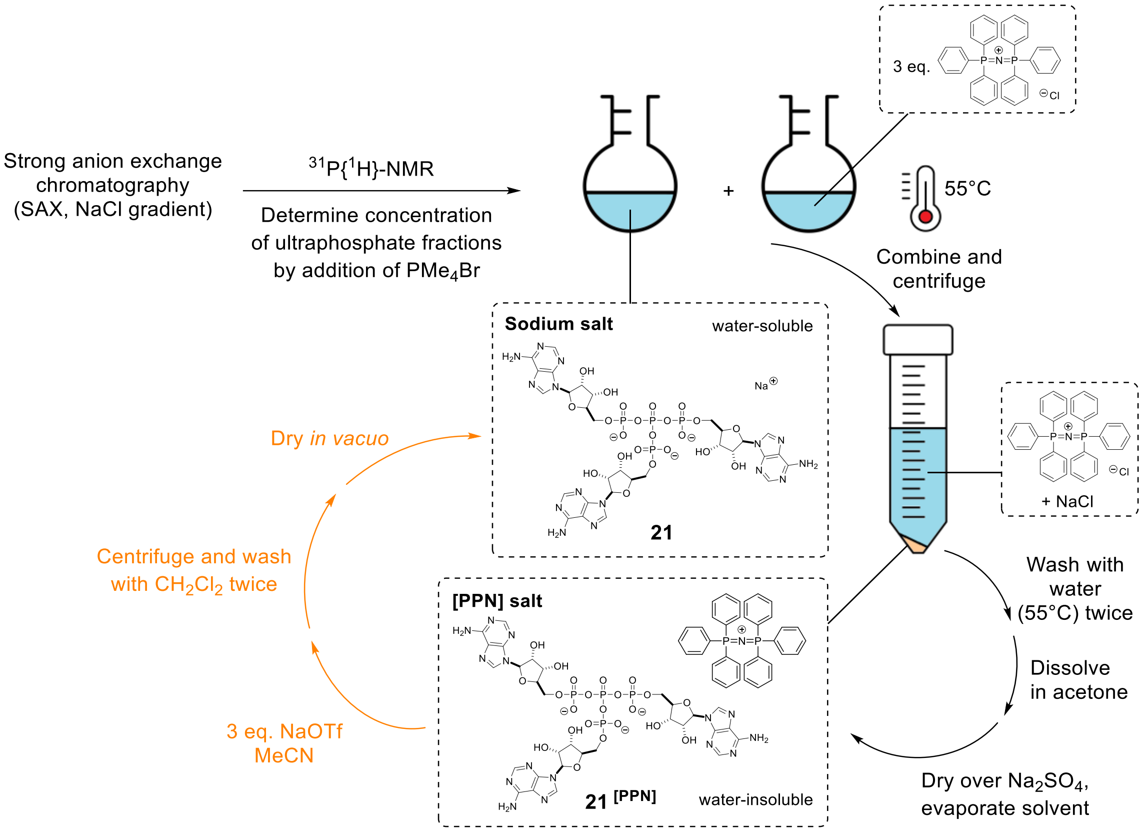

Supplementary Fig. 2│ Salt metatheses on ultraphosphates. Schematic representation of procedures for the synthesis of water-insoluble ultraphosphate [PPN] salts and the backward reaction to yield the water-soluble sodium salt (shown in orange) with trisadenosine ultraphosphate (**21**) as exemplary substrate.

General procedure for the salt exchange of ultraphosphates to [PPN] salts

The crude ultraphosphate is either purified by anion exchange chromatography using Q Sepharose® Fast Flow and a NaCl (1 M) gradient or by using a PuriFlash Column (30µ C18 AQ; water, MeCN gradient (0-45%), 10% TEAA (100 mM, pH 7.0)). Fractions containing ultraphosphate were analysed for their concentration by addition of a defined volume of a PMe_4Br solution in D_2O (1 mg/ml) and $^{31}P\{^1H\}$-NMR. Fractions were combined and heated to 55°C. [PPN]-Cl (2 to 3 eq.) was dissolved in H_2O (yielding approximately a 2-5 mM solution) at 55°C and added to the ultraphosphate solution. The precipitate was collected by centrifugation and washed with warm water (about 55°C). The residue was dissolved in acetone, dried over Na_2SO_4 and the solvent removed *in vacuo.*

Procedure for the salt exchange of ultraphosphate [PPN] to sodium salts[1]

To an ultraphosphate [PPN] solution in MeCN (about 5-10 mM) was added NaOTf (equal number of eq. as [PPN] present as counter-ion) in MeCN (about 75 mM). The precipitate was collected by centrifugation, washed with CH_2Cl_2 twice and dried *in vacuo.*

Supplementary Note 4. X-ray structure of tris(9*H*-fluoren-9-yl)methyl ultraphosphate

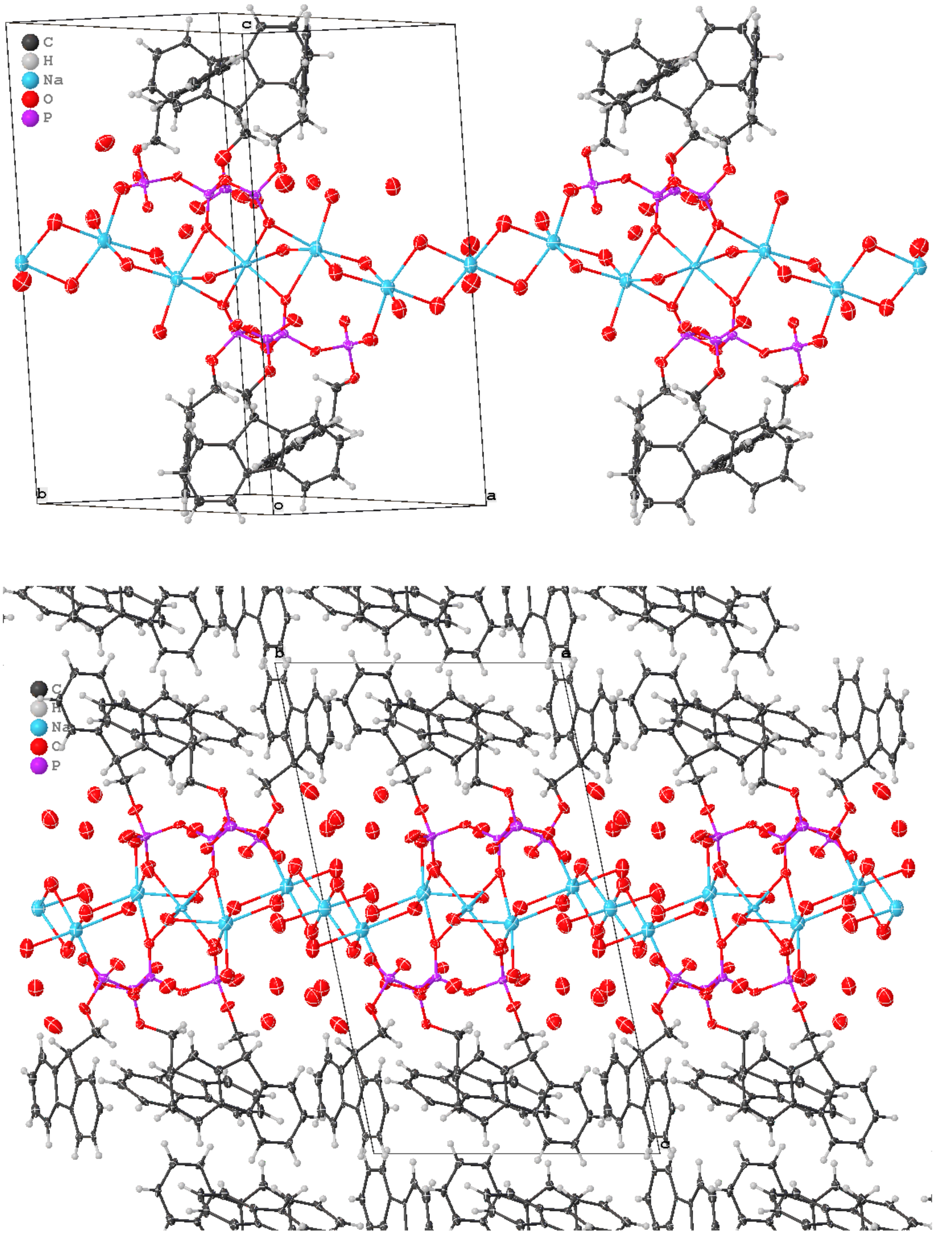

Supplementary Fig. 3│ X-ray structure of tris(9*H*-fluoren-9-yl)methyl ultraphosphate (37). Primitive cell and packing along a-axis.

Supplementary Note 5. Stability of ultraphosphates in aqueous media

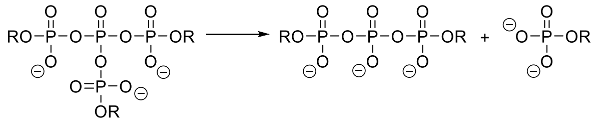

R	Addition of 0.1 eq. salt	pH 4.7	pH 6.0	pH 10.0	pH 13.9
R = ⊖ (uP_4)	■ No salt			12.5 h	47.4 h
	▼ $MgCl_2$			5.6 h	22.4 h
R = adenosine (**21**)	■ No salt	4.2 h	7.1 h	16.0 h	
	▲ NH_4Cl	4.1 h	6.5 h	15.4 h	
	▼ $MgCl_2$	3.3 h	4.8 h	7.5 h	
	● $CaCl_2$	2.0 h	2.5 h	3.1 h	

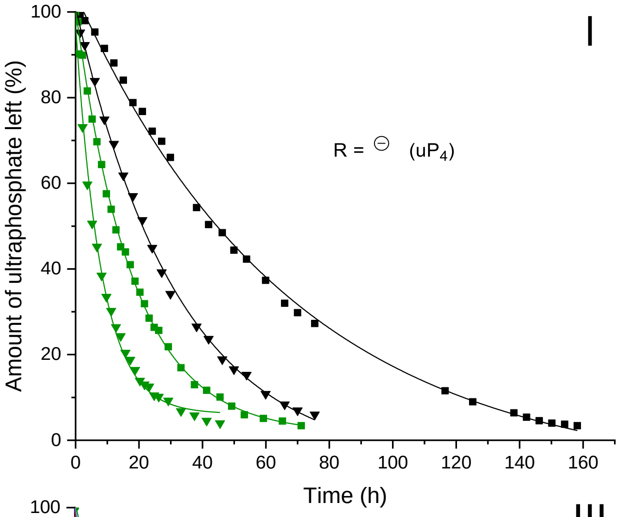

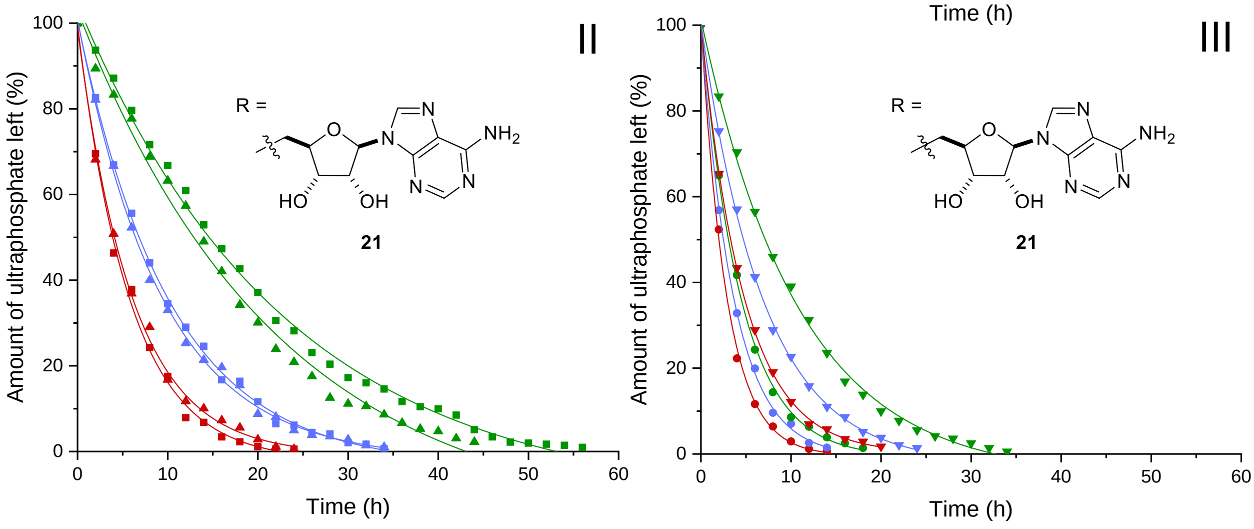

Supplementary Fig. 4 | Stability of ultraphosphates in aqueous media. Decomposition of trisadenosine ultraphosphate (**21**) and uP_4 analysed by $^{31}P\{^1H\}$-NMR under different pH values and in presence of 0.1 eq. of different cations. Half-lives were calculated assuming pseudo-first order reaction kinetics. **I**, uP_4 at pH 10.0 or 13.9; without salt addition or 0.1 eq. Mg^{2+}. **II**, **21** at pH 6.0, 10.0 or 13.9; without salt addition or 0.1 eq. NH_4^+. **III**, **21** at pH 6.0, 10.0 or 13.9; with 0.1 eq. Mg^{2+} or Ca^{2+}.

Supplementary Note 6. Gel electrophoresis of ultraphosphates

Polyacrylamide gel electrophoresis (PAGE) was carried out on a Hoefer SE660 Tall Standard Dual Cooled Vertical Unit. A stock solution of 10 × Tris-borate-EDTA (TBE) buffer (0.89 M Tris-HCl, 0.89 M boric acid, 20 mM EDTA; pH 8.3) was used for the preparation of 1 × TBE buffer. During pre-run and run, the lower buffer chamber was filled with 6 l of prechilled (4 °C) 1 × TBE buffer and the buffer was stirred. A recirculating cooler (Julabo F250) was used for chilling the buffer. Sample loading was performed with gel-loading pipet tips. The ultraphosphates were either used as aqueous solution of triethylammonium salts or as [PPN] salts dissolved in DMF. Based on the general procedure as described by Saiardi *et al*.[2], the PAGE procedure was conducted as follows:

1. The gel sandwich was assembled using glass plates (24 × 18 cm) and spacers (1 cm wide, 1.0 mm thick).
2. The monomer solution for the gel was prepared by stirring acrylamide (33.9 ml; 40 g/l acrylamide:bis-acrylamide 19:1, 3030 Carl Roth), 10 × TBE buffer (3.8 ml) and ammonium persulfate (APS) (200 µl, 0.1 g/ml APS in ddH_2O) for 2 min at 0 °C. *N*,*N*,*N*′,*N*′-tetramethylethylenediamine (TEMED) (20 µl) was added and the solution was stirred for 1 min. The mixture was poured between the precasted glass plates and a 15 lane comb was inserted. The solution was allowed to polymerize for 25-30 min at room temp.
3. After polymerization, the wells were washed with 1 × TBE buffer by using a syringe and needle to remove any precipitates and non-polymerized gel debris. The gel was prerun at 4 °C in 1 × TBE buffer for 30 min at 300 V.
4. Samples (22 µl volume per sample) were prepared by diluting a stock solution of the ultraphosphate (derivative) with ddH_2O. Orange G dye (OGD) (7 µl; 10 mM Tris-HCl, 1 mM EDTA, 0.3 g/ml glycerol, 1 mg/ml orange G, pH 7.0) was added to all samples prior to loading onto the gel (the final volume per well was 29 µl). The gel was loaded leaving 2-3 wells empty on each side.
5. The gel was run at 4 °C in 1 × TBE buffer for 20 h at 500 V.
6. After the run, the gel apparatus was disassembled. One glass plate was removed leaving the gel on the other glass plate.
7. Workup: The gel was stained for 30 min with toluidine blue staining solution (1 g/l toluidine blue, 200 g/l MeOH, 20 g/l glycerol) and then destained for 1.5 h with toluidine blue destaining solution (= toluidine staining solution without dye). The destaining solution was replaced once during the entire procedure.
8. The gel was scanned with a photo scanner or recorded with a camera.
9. Finally, the gel image was converted to greyscale and contrast and brightness were adjusted. The PAGE analysis of ultraphosphate and its Fm-protected derivatives is depicted in Supplementary Fig. 5 and the electrophoretic separation of adenosine ultraphosphate derivatives is shown in Supplementary Fig. 6.

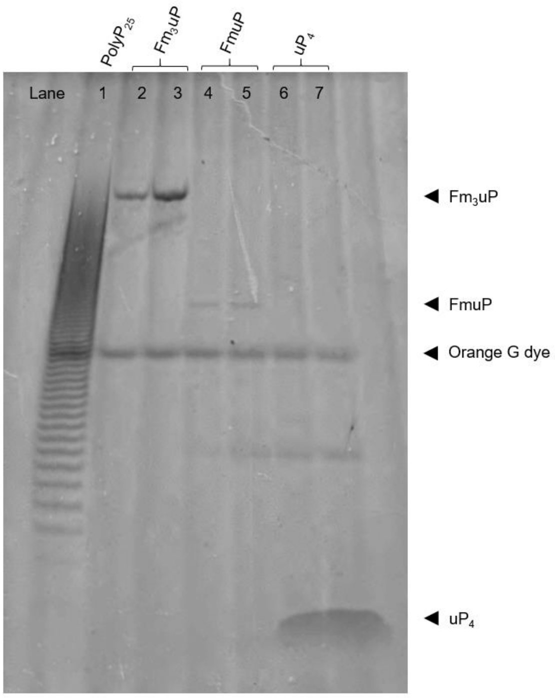

Supplementary Fig. 5│ PAGE of uP_4 and Fm-protected derivatives after staining with toluidine blue. Lane 1: $PolyP_{25}$ (vertical standard, 4 nmol). Lane 2: Fm_3uP (**37**, 5 nmol). Lane 3: Fm_3uP **(37**, 10 nmol). Lane 4: FmuP (**S1**, 10 nmol). Lane 5: FmuP (**S1**, 20 nmol). Lane 6: uP_4 (**2**, 100 nmol). Lane 7: uP_4 (**2**, 150 nmol).

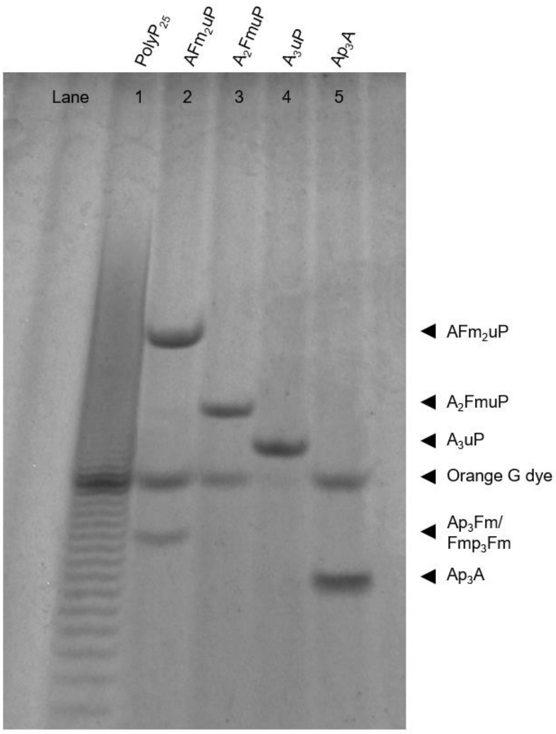

Supplementary Fig. 6│ PAGE of adenosine ultraphosphate derivatives after staining with toluidine blue. Lane 1: $PolyP_{25}$ (vertical standard, 4 nmol). Lane 2: AFm_2uP (**48**, 20 nmol). Lane 3: A_2FmuP (**45**, 15 nmol). Lane 4: A_3uP (**21**, 15 nmol). Lane 5: Ap_3A (25 nmol). The additional band in lane 1 might refer to Ap_3Fm or Fmp_3Fm that are possible decomposition products of AFm_2uP (**48**).

Supplementary Note 7. Polyphosphate analysis of yeast cell extracts

Preparation of yeast cell extracts (wild type, polyphosphate overexpressing strain GPD-vtc5)

Fractionation of yeast lysates was performed according to LANGEN and LISS.[3]

Yeast were shaken in YPD + 50 mM KH_2PO_4 (150 rpm, 30°C) and logarithmically grown overnight to an OD_{600} of 1-1.5. 500 OD_{600} units of cell suspension were centrifuged (4'600 x g, 5 min, JLA10.500 rotor) yielding a pellet of approx. 5 g of yeast (wet weight).

Fraction 1 (ortho-, tri-heptaphosphates, organic acid-soluble phosphates):
5 g wet weight of yeast was resuspended in 15 ml 1% trichloroacetic acid (TCA) at room temperature and shaken for 90 min. The suspension was centrifuged (5000 x g, 5 min, JA25.50 rotor). The supernatant was collected, neutralized with NaOH to pH 7.2 and frozen in five 1 ml aliquots at –20°C (= fraction 1). The pellets were subjected to a second extraction (for 15 min), as described above.

Fraction 2 (polyphosphates, 5% nucleic acid phosphates, phospholipids)
The remaining pellet from fraction 1 was shaken at room temp. for 15 min. with 15 ml H_2O, 1.5 g $NaClO_4$ and 0.5 ml 1 M TCA. After centrifugation (5000 x g, 5 min, JA25.50 rotor), the supernatant was collected and neutralized to pH 6.7-7.2. Five 1 ml aliquots (= fraction 2) were frozen at –20°C. The pellets were subjected to a second extraction, as before.

Fraction 3 (polyphosphates, 13% nucleic acids)
Remaining pellets from fraction 2 were resuspended in 5 ml H_2O. 4 ml 0.2 M NaOH were added and the suspension was shaken for 40 min at 0°C. After centrifugation (5000 x g, 5 min, JA25.50 rotor), the supernatant was collected and adjusted to pH 6.8 with HCl. Five 1 ml aliquots were frozen at –20°C (= fraction 3).

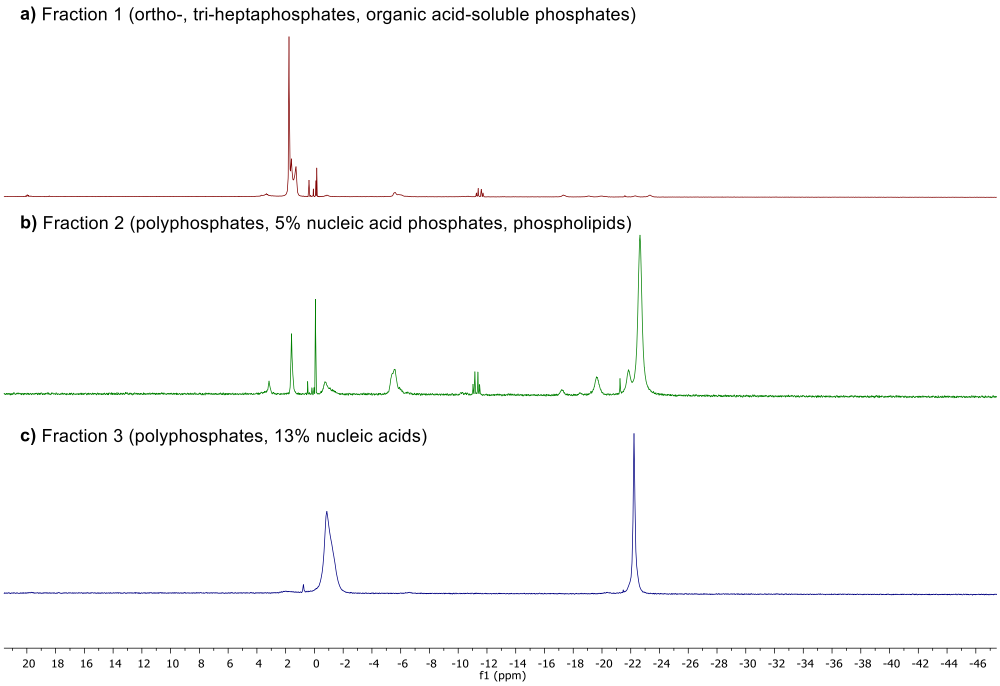

Supplementary Fig. 7| $^{31}P\{^1H\}$-NMR of fractionized GPD-vtc5 yeast strain polyphosphate extracts. a, Fraction 1: Ortho-, tri-heptaphosphates; organic acid-soluble phosphates. **b,** Fraction 2: Polyphosphates, 5% nucleic acid phosphates, phospholipids. **c**, Fraction 3: Polyphosphates, 13% nucleic acids.

Preparation of yeast cell extracts (polyP devoid Δ vtc4 and polyP accumulating Δ ppn1, ppn2, ppx1)

Six 1 l cultures in YPD medium were shaken (150 rpm, 30°C). Cells were logarithmically grown overnight to reach a final OD_{600} of 4. The suspensions were centrifuged (4'600 x g, 5 min, JLA9.100 rotor, 4°C) and the supernatants were discarded. Cells, beakers, and tubes were cooled on ice and all further steps were performed at 0-4°C. Pellets equivalent to a 1 l culture were resuspended in 150 ml of ice-cold washing buffer (100 mM NaCl, 50 mM PIPES/NaOH pH 7, 20 mM EDTA), pooled and transfered to 500 ml centrifuge bottles. Cells were sedimented (4'600 x g, 5 min, JLA10.500 rotor) and the supernatants were discarded. The pellets were resuspended in 50 ml ice-cold lysis-buffer (1 M NaCl, 50 mM PIPES/NaOH pH 7, 20 mM EDTA), transfered into 50 ml Falcon tubes and centrifuged (2'000 x g, 7 min, 4°C, JA25.50 rotor). Supernatants were discarded and the pellets were resuspended in 8 ml ice-cold lysis buffer, resulting in a thick slurry. The suspension was flash-frozen by pouring it as a thin stream under constant stirring into a 500 ml plastic beaker filled with 200 ml liquid nitrogen. This yields small nuggets that do not stick together. Avoid forming large clumps.

The frozen nuggets were transferred into a 2 kW Wareing blender filled with liquid nitrogen. The cells were blended for 5 min in liquid nitrogen, stopping every 30-60 s to replenish the evaporated nitrogen (top up 2-4x, reaching the upper leves of the blades). The cells never thawed during the procedure. After 5 min, blending was continued until almost all nitrogen had evaporated, yielding a frozen powder, which was transferred into a 500 ml beaker to let residual nitrogen evaporate. This powder could be stored at -80°C in an open plastic flask.

The powder was thawed under a flow of hand-warm water, under constant stirring. The lysate was centrifuged (12'000 x g, 10 min, 4°C, JLA25.50 rotor). The supernatant (approx. 6 ml) was transferred into polypropylene tubes for the TLA100-3 rotor and spun (55'000 x g, 30 min, 4°C). Floating lipid was aspirated. The cleared supernatant was flash-frozen in 500 µl aliquots in liquid nitrogen and stored at –80°C.

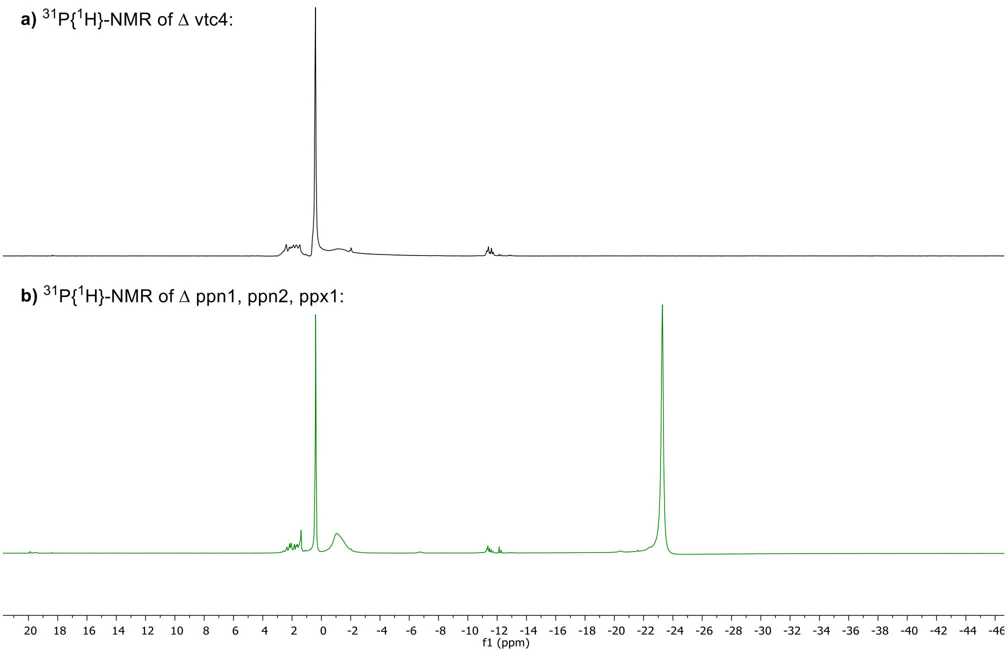

Supplementary Fig. 8 | $^{31}P\{^1H\}$-NMR of yeast polyphosphate extracts. **a**, Δ vtc4 yeast strain. **b**, Δ ppn1, ppn2, ppx1 yeast strain.

Supplementary Note 8. Enzymatic digestion of ultraphosphates

General procedure for kinetic measurements with ultraphosphate [PPN] salts and alkaline phosphatase from bovine intestinal mucosa

Enzyme solution (alkaline phosphatase from bovine intestinal mucosa, lyophilized powder, ≥10 DEA units/mg solid): (100 U/ml), Tris 10 mM pH 8.5, $MgCl_2$ 5 mM, $ZnCl_2$ 0.2 mM, glycerol 50%.
Enyzme blank: Tris 10 mM pH 8.5, $MgCl_2$ 5 mM, $ZnCl_2$ 0.2 mM, glycerol 50%.

An ultraphosphate [PPN] salt (1.00 µmol) was dissolved in acetone (360 µl) and D_2O (360 µl). Sodium carbonate/bicarbonate buffer (pH 9.5, 300 µl) and $MgCl_2$ solution (10 mM, 60 µl) were added. The solution was halved, either enzyme solution or enzyme blank (each 60 µl) added and the decomposition tracked by $^{31}P\{^1H\}$-NMR.

Kinetic measurement with denatured enzyme solution:
The enzyme solution was heated to 95°C for 20 min and used as described in the general procedure.

Kinetic measurement with EDTA:
EDTA was added to both the enzyme solution and enzyme blank to yield 20 mM. The solutions were used as described in the general procedure.

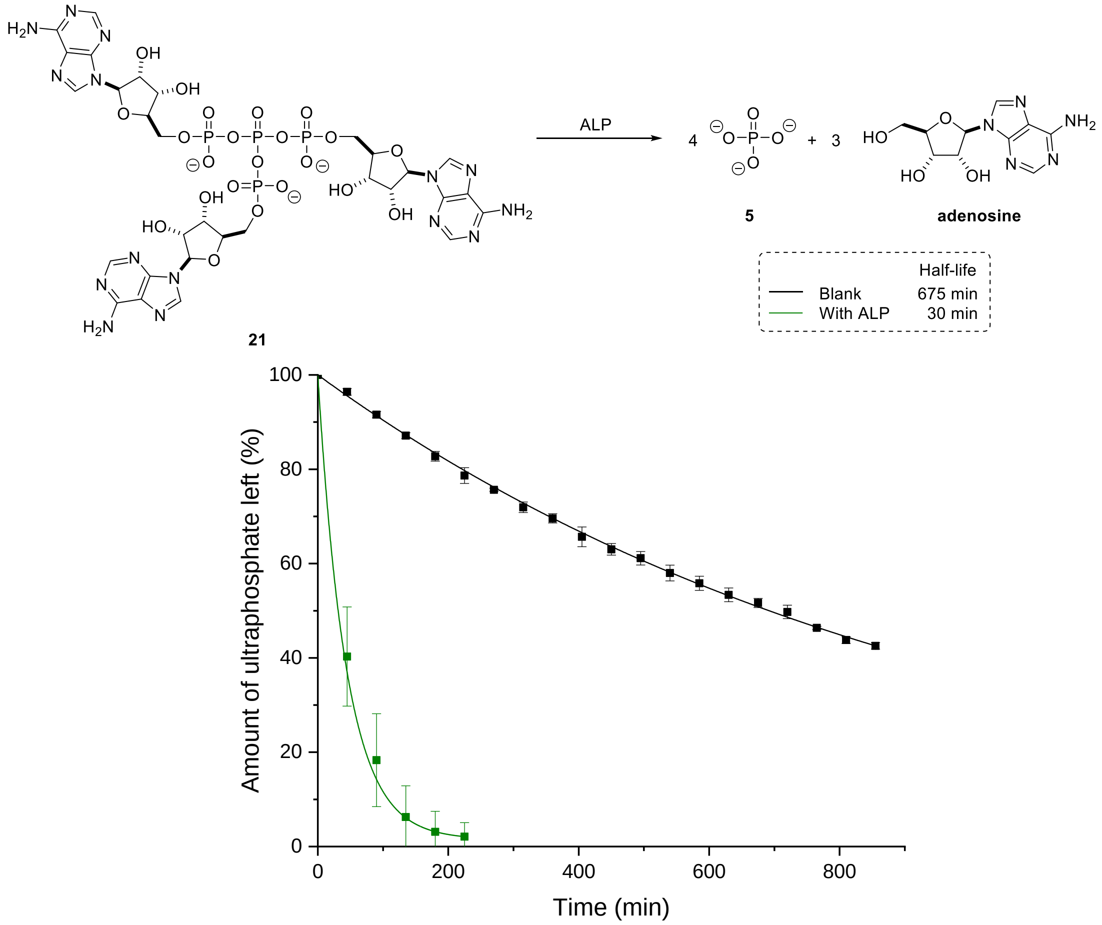

Supplementary Fig. 9│ Enzymatic digestion of trisadenosine ultraphosphate (21) by alkaline phosphatase from bovine intestinal mucosa. The results are means ± standard deviation from experiments performed in triplicates. Half-lives were calculated assuming pseudo-first order reaction kinetics.

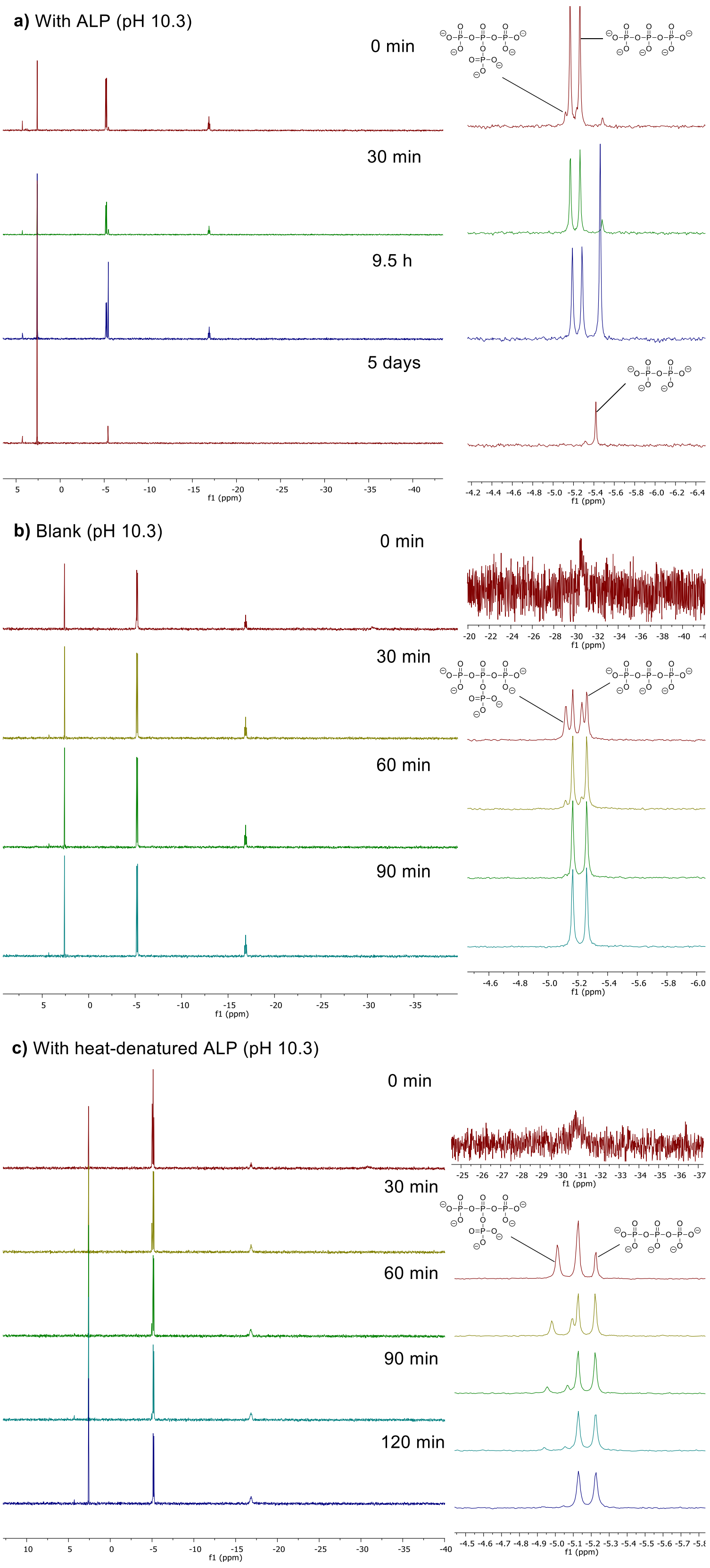

Supplementary Fig. 10│ ^{31}P-NMR spectra on the enzymatic digestion of uP_4 by alkaline phosphatase from bovine intestinal mucosa at pH 10.3. a, Sample with ALP. In the initial spectrum, mostly the decomposition products – inorganic mono- and triphosphate – but also a small amount of residual uP_4 were present. Digestion stops with phosphoric acid. **b**, The blank allowed detection of uP_4 for 60 min and followed the standard decomposition to inorganic mono- and triphosphate. **c**, The sample with heat-denatured ALP allowed detection of uP_4 for 90 min and followed the standard decomposition to inorganic mono- and triphosphate.

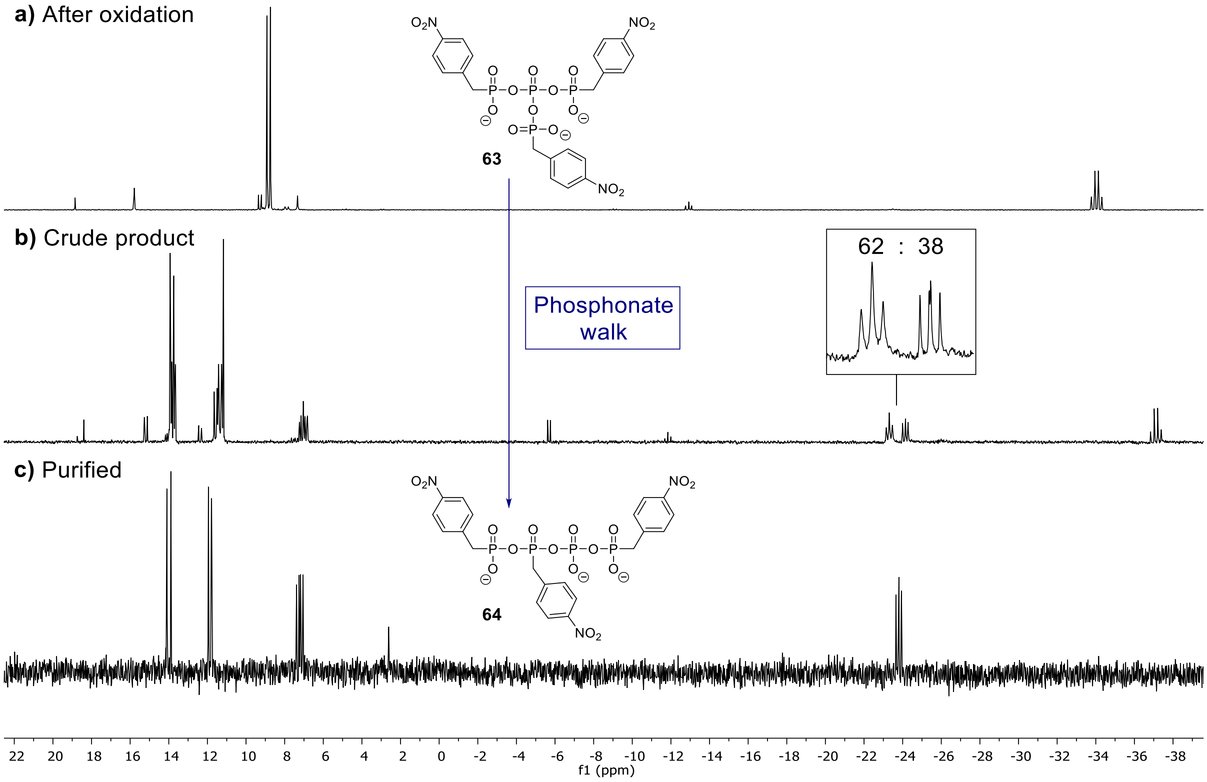

Supplementary Fig. 11 | $^{31}P\{^{1}H\}$-NMR spectra of the attempted synthesis of tris(*para*-nitrobenzyl phosphonyl) ultraphosphate (63). **a**, After oxidation, reaction control indicated a clean reaction. **b**, The crude product showed additional decomposition products which were not observed in the standard decay. **c**, Purification by SAX chromatography allowed the isolation of the linearized product **64** arising from the phosphonate walk.

Supplementary Note 9. Ultraphosphate rearrangement

Supplementary Note 9.1. Experimental results

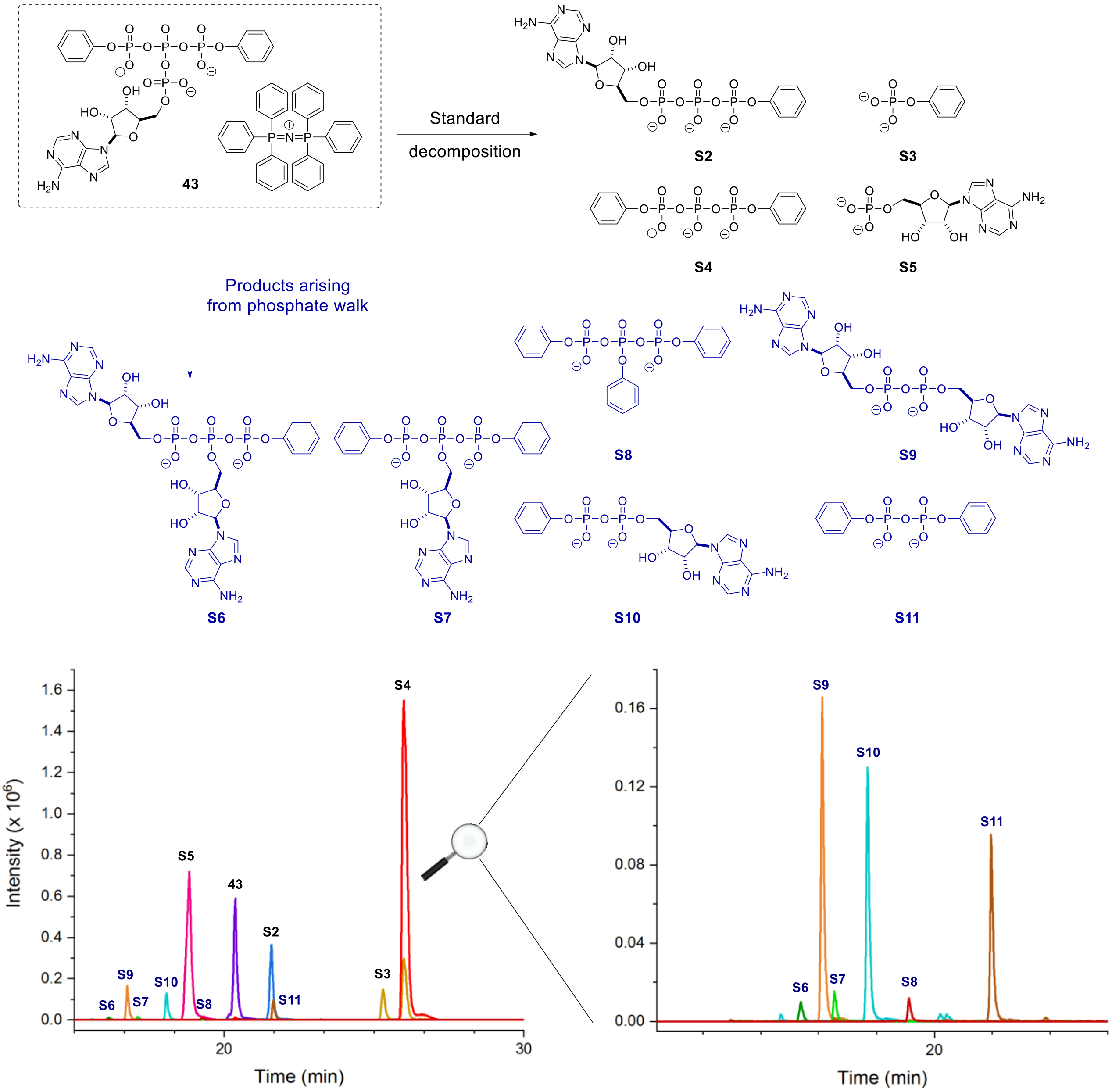

Supplementary Fig. 12│ Decomposition of bisphenyladenosine ultraphosphate (43) [PPN] salt in DMF. CE-MS analysis of the product mixture after 25 days and proposed structures.

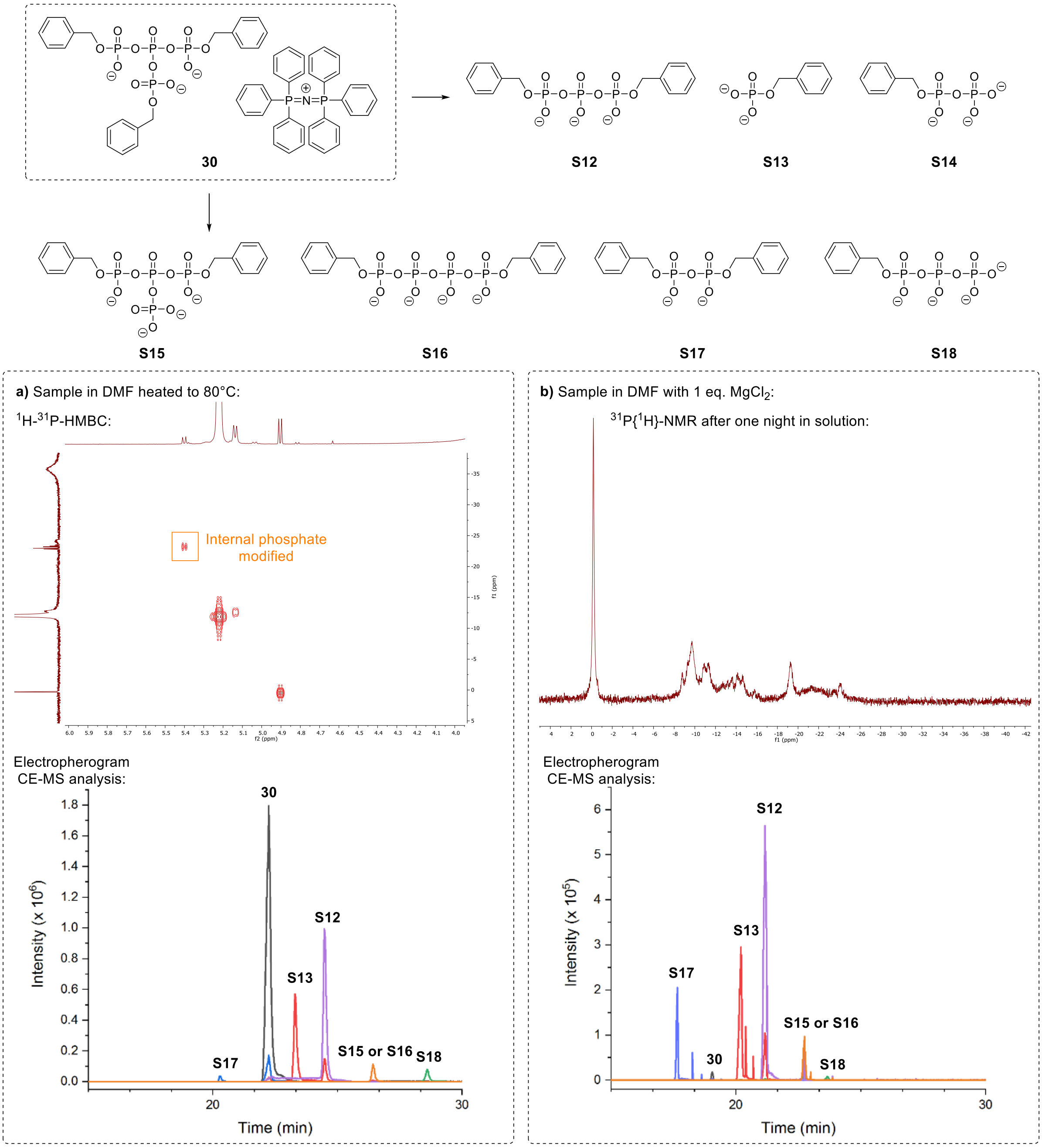

Supplementary Fig. 13│ Decomposition of tribenzyl ultraphosphate (30) [PPN] salt in DMF. a, Sample heated to 80°C for 5 h. ^{1}H-^{31}P-HMBC with cross-peak for internally modified oligophosphate and CE-MS analysis of the product mixture. **b**, Sample with 1 eq. $MgCl_2$. $^{31}P\{^{1}H\}$-NMR after one night in solution and CE-MS analysis of the product mixture.

Supplementary Note 9.2. Computational results

a)

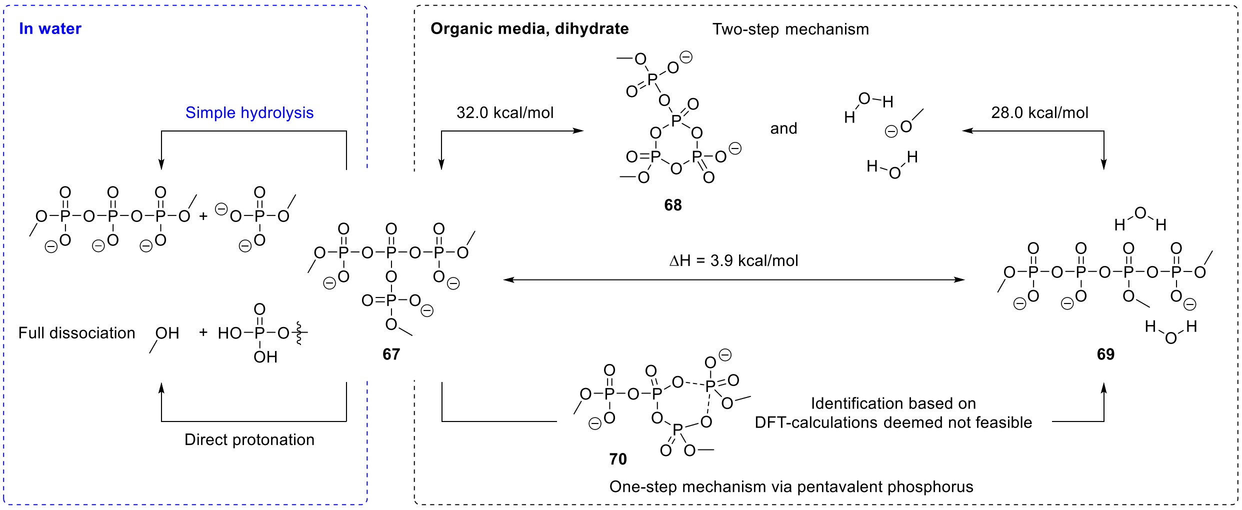

b)

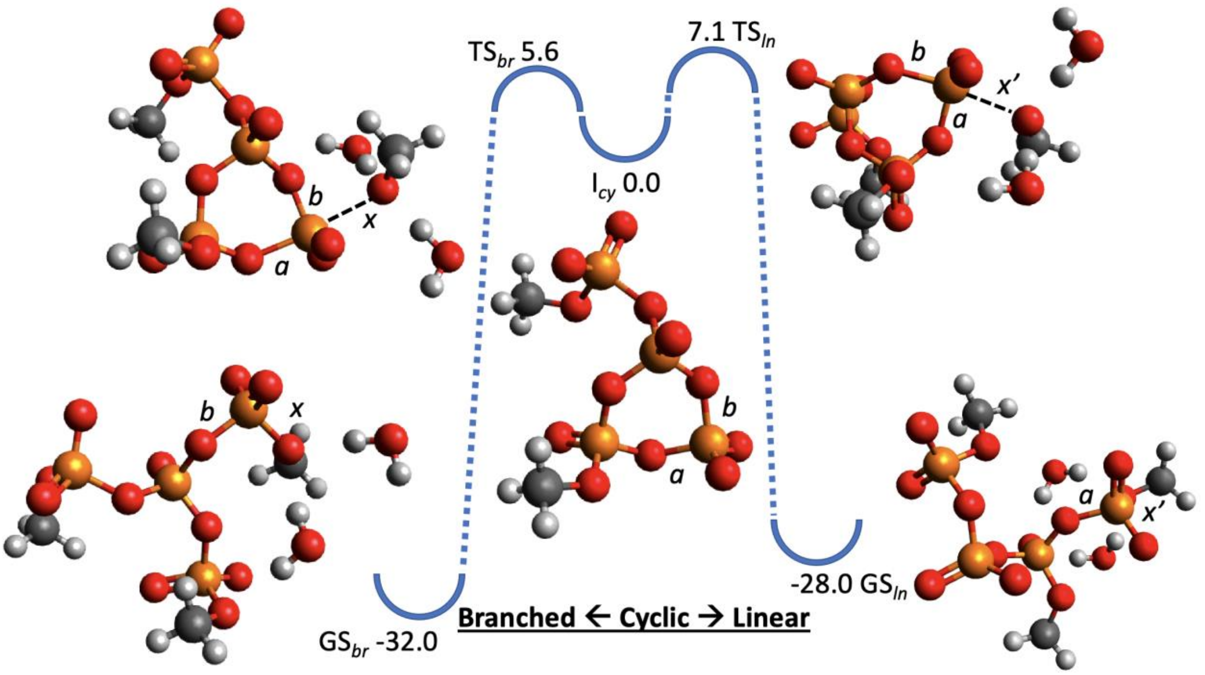

	GS_{br}	TS_{br}	I_{cy}	TS_{ln}	GS_{ln}
**A**		1.888	1.691	1.684	1.675
**B**	1.682	1.668	1.676	1.870	
**X/X′**	1.642	2.409		2.392	1.645
**Energy**	−32.0	+5.6	0.0	+7.1	−28.0

Supplementary Fig. 14 | DFT-calculations on the phosphate walk rearrangement of trimethyl ultraphosphate. a, Reactivity in water and mechanistic pathways of the phosphate walk rearrangement for trimethyl ultraphosphate (**67**) based on DFT calculations. **b**, Energy diagram for the phosphate walk rearrangement of the dihydrate of **67** and selected distances in the calculated structures in Å. Calculations were performed at the B97D/Def2-TZVPD(water) level of theory; ΔH is given in kcal/mol. All phosphates are tetrahedral in the minimum structures.

The structural and energetic analyses of the molecular systems for all compounds described in this study were carried out using GAMESS[4] and Gaussian09[5] software packages, as both are developed in this group. The B97-D dispersion enabled density functional method[6,7] was employed using an ultrafine grid, together with the Def2-TZVPD basis set.[8] Full geometry optimizations were performed and uniquely characterized via second derivatives (Hessian) analysis to establish stationary points and effects of zero point and thermal energy contributions. Calculations were carried out with zero, one and two explicit solvent molecules to track trends in energetics. Transition state modes clearly show connection to reactants and products in each case, however in addition, Intrinsic Reaction Coordinate analyses in solvent were carried out to ensure the reaction is as represented. A predictor-corrector method utilizing second derivatives[9,10] was employed using 0.05 bohr stepsize. Effects of solvent employed the COSMO:*ab initio* continuum method using a dielectric as in experiment.[11,12] Visualization and analysis of structural and property results were obtained using Avogadro[13] and WEBMO.[14]

B97-D/Def2-TZVPD(water) Energetic Comparison with explicit solvent

continuum → continuum + 1 h2o → continuum + 2 h2o	continuum	1 h2o	2 h2o
**straight chain**			
ΔG	−49.0	−42.0	−36.5
ΔH	−47.6	−38.0	−35.1
**branched chain**			
ΔG	−49.1	−43.7	−38.7
ΔH	−48.0	−43.3	−37.5

Ring_Me3_TSb_h2o_5_2h2o_3_b97dDef2tzvpd_water
Transition State with imaginary mode -229.0

35

P 0.810137911 1.8329821709 1.07840401
O -0.3352779355 1.3175238695 0.060011486
O 1.5278880767 2.9969716012 0.3011802866
O 1.9435356271 0.7150327298 1.0004312647
O 0.2892710859 2.1445076695 2.4246296037
P -0.7971087782 -0.2373982866 -0.2096326793
P 0.9593055073 4.5610175733 -0.1024913793
P 1.8552948234 -0.9252667619 1.2872622002
O -1.7745980844 -0.5669447147 1.0122912494
O 1.2669849368 4.5197881306 -1.691518107
O 3.7234424681 -0.3272861821 2.685596138
O -1.4317264814 -0.3144015409 -1.5464119818
O -0.5074619696 4.5967171285 0.1921642417
O 1.9269589677 5.5211863604 0.5064848339
O 2.8585193403 -1.684644543 0.4958091691
O 1.1101558633 -1.3113734964 2.5161549233
C -3.056582955 0.1199685595 1.1103316942
C 0.4987225405 3.6329934101 -2.5453142556
C 3.3976217445 0.5131449746 3.7658680215
H -3.5457456831 -0.2852690165 1.9996657424
H -2.8941274725 1.19810828 1.2271198497
H -3.6614676519 -0.0809052105 0.218725796
H 0.8387053398 3.820829936 -3.5680165385
H 0.6891770698 2.5874004951 -2.2734191314

H -0.5729165755 3.8500205164 -2.4589253245
H 3.0972056504 1.5220021353 3.4216257399
H 2.5557632493 0.1088825906 4.3604547448
H 4.2559865821 0.6443440294 4.4550951999
O 0.4560932473 -1.1067876147 0.0322446364
O 5.1361527247 1.0729715515 1.0000304364
H 4.4718229625 1.5025447606 0.4440342713
H 4.5787548678 0.5098043301 1.6534371533
O 4.5041593597 -2.6458438807 3.5536962469
H 3.8345812358 -3.2735467489 3.2493527934
H 4.1790324052 -1.7344548058 3.1998376646

Ring_Me3_TSf_protTS_10_2h2o_b97dDef2tzvpd_water
Transition State with imaginary mode -225.4

35

P 0.8905536071 -0.1025032359 1.5169649034
O 0.5951587951 1.2004468443 0.5555809433
O 1.7964409939 -1.0560896724 0.6135353241
O -0.4695513847 -0.830284693 1.6594356298
O 1.5278448121 0.3403615599 2.7763112322
P -0.6642081953 1.3629204733 -0.4452671161
P 3.1219014472 -0.7254102676 -0.3600768591
P -1.7258689745 -1.3232737468 0.3651121307
O -1.8085330024 2.0532213666 0.4165420266
O 2.2563793879 -0.4176146798 -1.7080793393
O -0.2760691144 2.0615942504 -1.6849867063
O 3.8194749544 0.5087950691 0.1291803621
O 3.859650902 -2.0243552246 -0.481364836
O -1.1453195384 -2.6333416828 -0.0349409172
O -2.9231608314 -1.0218116592 1.1952865837
C -1.6873574984 3.4601800621 0.7952354212
C 2.9480256328 0.1508621308 -2.8433186846
H -2.6006007338 3.695130639 1.3463236026
H -0.8088341352 3.595239231 1.4363998839
H -1.6100978858 4.0817934416 -0.1032463643
H 2.1988236039 0.2745791833 -3.6312784421
H 3.3772599399 1.1275016096 -2.5833899531
H 3.7430195091 -0.52245257 -3.1934389842
O -1.2274792406 -0.0940862757 -0.6726149353
O -3.2896151079 -1.5442549705 -1.4318684478
C -2.7266118218 -1.8000965865 -2.6953364243
H -2.1691894127 -0.9239127362 -3.0811692258
H -3.5039300585 -2.0499652242 -3.445101218
H -2.0162874449 -2.6492584408 -2.6633553342
O -4.3806940456 0.8292815988 -1.3931760031
H -3.9468156222 -0.1002732552 -1.3964983427
H -3.809142043 1.3537858425 -0.8149060166
O -4.6708279027 -3.5945052287 -0.6333107604
H -4.1094324946 -2.786174893 -0.9364183034
H -4.2037070966 -3.9316882593 0.1432351703

UP_Cy_Me2_ring_nomethoxy_b97dDef2tzvpd_water
Positive Definite Stationary Point

24

P 0.1570224668 2.2523563365 1.6213492285
O -0.7461386706 1.5157079382 0.494636451
O 0.9028373302 3.4206360204 0.8712037698
O 1.3667692396 1.2539127402 1.9020061089
O -0.6434943667 2.6377138085 2.7970204466
P -0.6499739256 0.0772478905 -0.2537633722
P 0.2878102493 4.6161034139 -0.1667285923
P 1.3974966056 -0.4196474435 1.8108768038
O -1.7894003224 -0.7927612457 0.4176759727
O 0.4864505023 3.7511559975 -1.530495689
O -0.7106716953 0.222505086 -1.7188165303
O -1.1621119757 4.8312236549 0.1391791034
O 1.2784574664 5.7336742838 -0.084311467
O 2.818972686 -0.8116618171 1.6464591282
O 0.4840060108 -0.9834207622 2.8399166164
C -3.195503788 -0.5065916922 0.1225915657
C -0.224521486 4.1720036903 -2.7198983142
H -3.7667893043 -1.2605362331 0.6681714732
H -3.4481253782 0.4980807738 0.4795769794
H -3.3737244512 -0.5922368903 -0.954500512
H 0.0456478821 3.4604798319 -3.5058977077
H -1.3080170074 4.1446487134 -2.5479688081
H 0.0799829724 5.1847815951 -3.0179533647
O 0.6830589598 -0.5860656909 0.28766071

Ring_Me3_TSb_h2o_5fullopt_b97dDef2tzvpd_water
Positive Definite Stationary Point

32

P 0.3896243705 0.5989003267 1.2440219229
O -0.4477354714 0.4483533634 -0.0990342823
O 1.2535267964 1.9063541268 0.9973940738
O 1.4823671577 -0.5463516093 1.0953846772
O -0.3949798203 0.5883611002 2.5009156387
P -1.7195527554 -0.6097519418 -0.4622778339
P 0.8659284491 3.5459265901 0.9023747302
P 2.7217868532 -1.1224220717 2.0835696895
O -2.9447567509 0.0666940093 0.3592176099
O 1.664865472 3.8796312801 -0.4777209413
O 3.6558989269 0.2073727571 2.2494043663
O -1.8926270817 -0.4593093444 -1.9419883039
O -0.6160814258 3.6794838783 0.7405707876
O 1.6133557216 4.2289634996 2.00162375
O 3.4688220581 -2.0711768326 1.202859611
O 2.1333662908 -1.5338766821 3.3961551945
C -3.407725251 1.3893523007 -0.0155681162
C 1.1478944935 3.363439638 -1.7321292632
C 3.444650441 1.1234800197 3.3576155684
H -4.2478642992 1.6201386623 0.6468439098

H -2.6064412528 2.1249720841 0.1271724335
H -3.7432765499 1.3958406131 -1.0602979173
H 1.8215955982 3.7257723982 -2.5142553672
H 1.1470322585 2.2662127405 -1.7184822369
H 0.1310426244 3.7341569237 -1.907546075
H 2.4146316494 1.4939711646 3.3668637643
H 3.6717976453 0.6210824666 4.3043700746
H 4.1338371235 1.9572212976 3.1944533317
O -1.4501230662 -1.9339039412 0.1798639673
O 4.2190096851 1.8210377433 -0.1652427931
H 3.449459051 2.407256726 -0.2351854424
H 4.0333610582 1.2886767132 0.6312234713

Ring_Me3_TSf_protTS_4opt_b97dDef2tzvpd_water
Stationary Point with zero negative eigenvalues

32

P -1.4277894311 2.8437585208 1.6873795373
O -1.6026044151 1.6459832998 0.5120522002
O -0.0749480734 3.5858696818 1.157568311
O -1.0881861551 2.2155205728 3.0009368918
O -2.6310652568 3.7162583389 1.5270506072
P -1.1366445454 0.1274946846 0.4363856055
P 0.3729612072 4.2311820236 -0.295819687
P 1.5005864033 -0.9649014374 1.4608711061
O -1.8769355205 -0.6596409164 1.6098512118
O 1.0558133095 2.8717756617 -0.9044483771
O -1.3712737193 -0.4101238256 -0.9241728685
O -0.8206941093 4.6283811314 -1.1186943221
O 1.4331397923 5.2516492174 0.0148824026
O 2.8303833476 -0.3085117972 1.2579372881
O 1.0925143773 -1.471534888 2.8092234055
C -3.3152083179 -0.8775870807 1.5214354486
C 1.5109599818 2.9018307812 -2.2731915842
H -3.5833406913 -1.4582744679 2.4074146946
H -3.8389046737 0.0858931644 1.5276147818
H -3.5567862773 -1.4389528535 0.6118198319
H 1.9352157216 1.9144272886 -2.4828772615
H 0.6732168454 3.0956688381 -2.9571609098
H 2.2851651059 3.670032957 -2.4131079013
O 0.3679747112 0.1462089073 0.9288325166
O 1.2310034749 -2.1035798258 0.3104358049
C 2.1949565201 -3.1842642594 0.209545373
H 3.1810618981 -2.7878967144 -0.0617746136
H 1.8277893946 -3.8487037676 -0.5780459555
H 2.2588027377 -3.7350369466 1.157581419
O -1.1710802327 -3.8861969996 0.6191341253
H -0.4876899415 -3.1935564746 0.5567680817
H -0.6810134682 -4.7085428146 0.475502836

The energy of highly charged species, with variable counter ions in high vs. moderate dielectrics involving hydrogen bond donors, are sensitive to intricate changes in speciation, constitutional and conformation isomerism, and explicit viz continuum environmental effects.[15-17] This issue has been discussed previously in a study that systematically looked at monophosphate ester hydrolysis as a function of protonation state and medium effects.[18]

Mechanistically, cyclic species **68** could arise from displacement of XOR, where X could be a lone pair as in :OR- , a proton (HOR), or a metal ion. Studies on the displacement of :OR- and HOR found transition geometries between cyclic and either branched or linear forms. Energies in water with :OR- as leaving group are substantially higher than one might expect from the experimental reaction times observed. Studies in which the OR in the ester is directly protonated as HOR lead to full dissociation of HOR. Therefore, explicit mono- and dihydrates were considered, where the explicit waters were intentionally placed in positions consistent with prevailing mechanistic models.[15]

Supplementary Note 10. Reactivity of uP_4 as phosphorylating agent

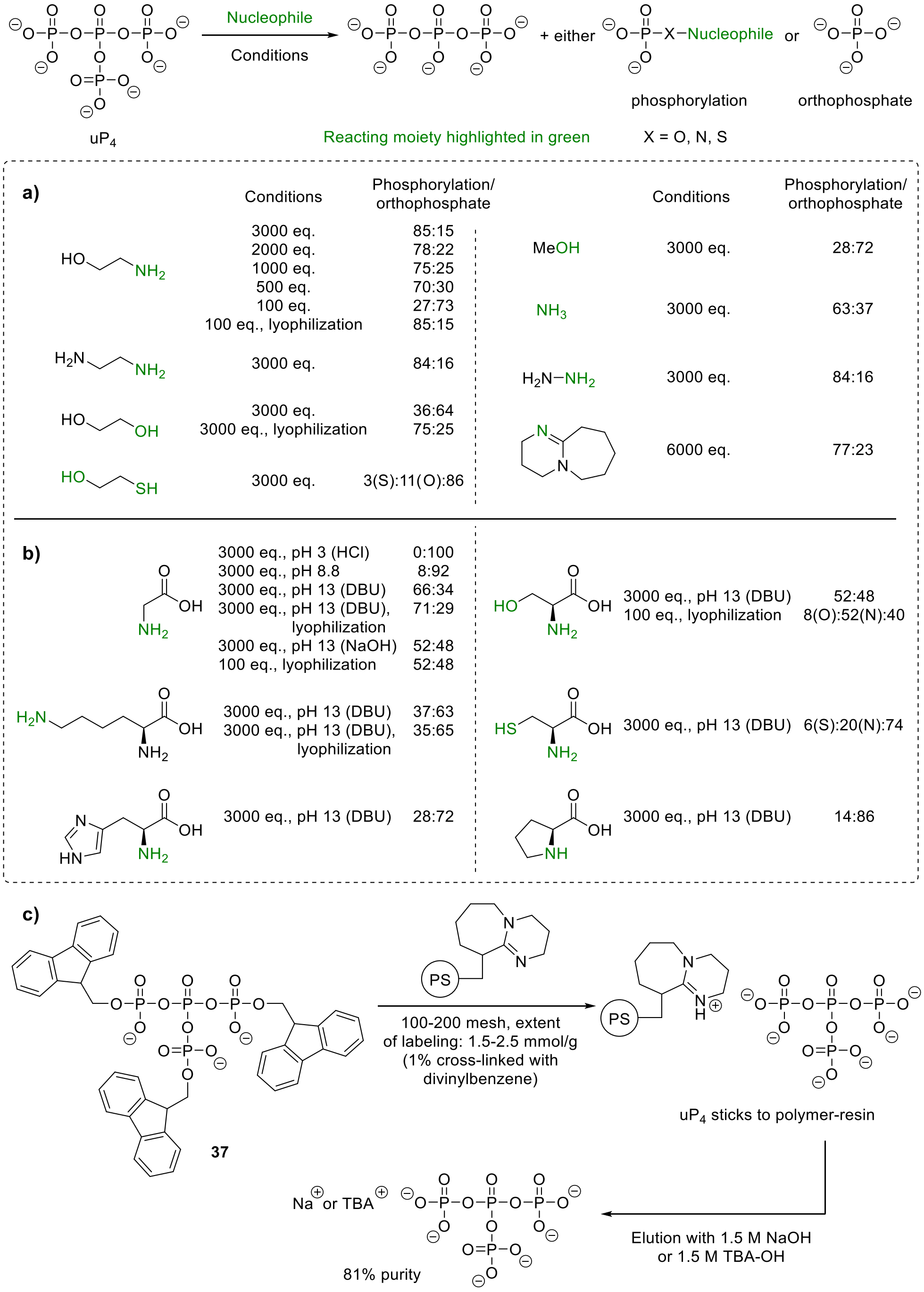

Supplementary Fig. 15│ Phosphorylation of different nucleophiles by uP_4. The reacting moiety of the nucleophile is marked in green. If the pH was adjusted, the applied acid or base is indicated in brackets. Phosphorylation rates were calculated without consideration of the phosphoramidate by-product arising from the reaction of DBU with uP_4. **a**, Phosphorylation of aliphatic and amine nucleophiles. **b**, Phosphorylation of amino acids. **c**, Procedure for the deprotection of **37** using polymer-bound DBU to avoid side-reactions of uP_4 with DBU. After full deprotection, uP_4 sticks to the polymer-resin and must be eluted using a 1.5 M solution of either NaOH or TBA-OH.

Supplementary Methods

Supplementary Methods 1. Monophosphate syntheses

2-Nitrobenzyl dihydrogen phosphate (S25)

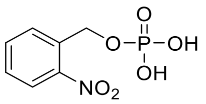

Chemical Formula: $C_7H_8NO_6P$
Exact Mass: 233.0089

To a stirred solution of 2-nitrobenzyl alcohol (0.20 g, 1.33 mmol, 1.0 eq.) in THF (3 ml) at –40°C was added diphosphoryl chloride (0.48 ml, 0.83 g, 3.32 mmol, 2.5 eq.) and the resulting mixture stirred at –40°C for 2 h. The reaction was quenched with water and treated with sat. $NaHCO_3$ solution until pH ≈ 8. The solution was made acidic using a 1 M HCl solution and extracted using AcOEt. The combined org. layers were washed with brine, dried over Na_2SO_4 and the solvent removed under reduced pressure to give the product [154 mg, 661 µmol, 50%] as a white solid.

Analytical data were in accordance with literature.[19]

1-(2-Nitrophenyl)ethyl dihydrogen phosphate (S26)

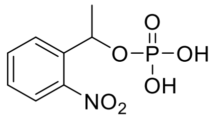

Chemical Formula: $C_8H_{10}NO_6P$
Exact Mass: 247.0246

To a stirred solution of 1-(2-nitrophenyl)ethanol (1.00 g, 5.99 mmol, 1.0 eq.) in THF (15 ml) at –40°C was added diphosphoryl chloride (2.2 ml, 3.77 g, 15.0 mmol, 2.5 eq.) and the resulting mixture stirred at –40°C for 2 h. The reaction was quenched with water and treated with sat. $NaHCO_3$ solution until pH ≈ 8. The solution was made acidic using a 1 M HCl solution and extracted using AcOEt. The combined org. layers were washed with brine, dried over Na_2SO_4 and the solvent removed under reduced pressure. The product was purified by reversed-phase chromatography (H_2O/MeOH 1:1) to give the product [637 mg, 2.58 mmol, 45%] as a brownish oil which solidified under cooling.

Analytical data were in accordance with literature.[19]

DEACM phosphate (S27)

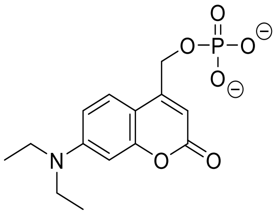

Chemical Formula: $C_{14}H_{16}NO_6P^{2-}$
Exact Mass: 325.0726

DEACM-OH (200 mg, 0.73 mmol, 1.0 eq.) and bis-(fluorenylmethyl) phosphoramidite (about 75%, 608 mg, 0.87 mmol, 1.2 eq.) were coevaporated using MeCN (3 x 3 ml) and dissolved in DMF (3 ml). A solution of ETT (133 mg, 1.02 mmol, 1.4 eq.) in DMF (2 ml) was added and stirred for 30 min. At 0°C, *m*CPBA (≤ 77%, 245 mg, 1.09 mmol, 1.5 eq.) was added and stirred for 10 min. Piperidine (0.6 ml, 516 mg, 6.06 mmol, 8.3 eq.) was added and stirred for 25 min. The reaction mixture become solid, was resolved with Et_2O and precipitated using a mixture of Et_2O/acetone. The resulting oil was washed with Et_2O/acetone. Drying *in vacuo* and purification by AIEX chromatography (Q Sepharose® Fast Flow, increasing concentrations of NH_4HCO_3) gave the product [eluting at 0.3-0.5 M buffer concentration, 112 mg, 0.31 mmol, 42%] as a yellow solid. Residues of *m*CPBA and ETT in the product were removed by several washings with acetone.

Analytical data were in accordance with literature.[20]

(9*H*-Fluoren-9-yl)methyl dihydrogen phosphate (S28)

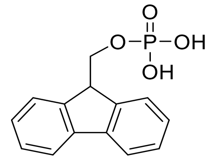

Chemical Formula: $C_{14}H_{13}O_4P$
Exact Mass: 276.0551

To a stirred solution of 9-fluorenylmethanol (5.50 g, 28.1 mmol, 1.0 eq.) in THF (55 ml) at –40°C was added diphosphoryl chloride (10.1 ml, 17.7 g, 70.1 mmol, 2.5 eq.) and the resulting mixture stirred at –40°C for 2 h. The reaction was quenched with water (30 ml) and treated with sat. $NaHCO_3$ solution (about 800 ml) until pH ≈ 8. The solution was made acidic using a 1 M HCl solution and extracted using AcOEt. The combined org. layers were washed with brine, dried over Na_2SO_4 and the solvent removed under reduced pressure to give the product [7.73 g, 28.0 mmol, quantitative] as a white solid.

The compound was synthesized as previously reported; analytical data were in accordance with literature.[21]

Supplementary Methods 2. Determination of purities and yields of ultraphosphates

The inherent instability of ultraphosphates complicates the isolation procedures and determination of yields of purified products. Depending on the modifications of the terminating phosphates, purified ultraphosphates may be precipitated from aqueous solutions. However, subsequent drying *in vacuo* leads to decomposition products again. For products, which do not readily precipitate, the molarity of fractions after purification must be determined by addition of PMe_4Br as a standard. Unclear salt compositions, additionally, impede the determination of molecular masses.
Therefore, exemplary yields are given to illustrate the efficiency of the syntheses protocols:

Yield for the synthesis of trisadenosine ultraphosphate (**21**) after precipitation: 55%.
Yield for the salt metathesis of trisadenosine ultraphosphate (**21**) from sodium to [PPN] salt: 48%.
Yield for the synthesis of the unsymmetrically modified ultraphosphate bis((9*H*-fluoren-9-yl)methyl)phenyl ultraphosphate (**46**) determined by addition of PMe_4Br to pure fractions after reversed-phase purification: 22%.

Purities according to $^{31}P\{^1H\}$-NMR thus are well suited for the comparison of ultraphosphate syntheses. If not stated otherwise in the synthetic procedure, the pure product (according to $^{31}P\{^1H\}$-NMR) could be obtained in solution or in solid form as [PPN] salt.

Supplementary Methods 3. Ultraphosphate syntheses

Triphenyl ultraphosphate (20)

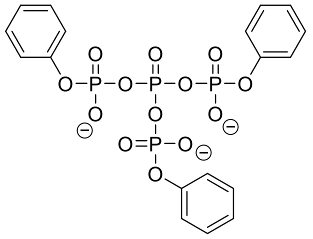

Chemical Formula: $C_{18}H_{15}O_{13}P_4^{3-}$
Exact Mass: 562.9480

Phenyl phosphate · 1.1 TBA (810 mg, 1.81 mmol, 3.0 eq.) and ETT (235 mg, 1.81 mmol, 3.0 eq.) were coevaporated using MeCN (3 x 6 ml) and dissolved in DMF (6 ml). $P(NEt_2)_3$ (165 µl, 149 mg, 0.60 mmol, 1.0 eq.) was added and stirred for 10 min. *m*CPBA (≤ 77%, 203 mg, 0.90 mmol, 1.5 eq.) was added at 0°C and stirred for 10 min. The product was precipitated using ice-cooled Et_2O/pentane (5:1, 200 ml), the suspension centrifuged and the pellet washed with ice-cooled Et_2O (200 ml). Drying *in vacuo* gave a colorless oil (m_{crude} = 1055 mg, purity according to $^{31}P\{^1H\}$-NMR: 78%), which was purified by AIEX chromatography (Q Sepharose® Fast Flow, increasing concentrations of NaCl, eluting at 0.7 M buffer concentration). The ultraphosphate was converted into its [PPN] salt according to the general procedure.

Signals for [PPN] not indicated: **^{1}H-NMR** (400 MHz, CD_3CN): δ = 6.93 (m_c, 3 H), 7.15 (m_c, 6 H), 7.26 ppm (m, 6 H). **^{31}P{^{1}H}-NMR** (162 MHz, CD_3CN): δ = –37.14 (q, *J* = 20.4 Hz, 1 P), –18.15 ppm (d, *J* = 20.2 Hz, 3 P). **^{13}C-NMR** (101 MHz, CD_3CN): δ = 122.0 (d, *J* = 4.9 Hz), 123.1, 129.7, 154.9 ppm (d, *J* = 7.2 Hz). **HRMS (ESI)**: *m/z* calcd for $C_{18}H_{18}O_{13}N_aP_4$ [M + Na^+]$^+$: 588.9590, found: 588.9590. **Raman:** $\tilde{\nu}$ = 3174 (vw), 3143 (vw), 3062 (s), 3012 (vw), 2993 (vw), 2962 (vw), 1589 (m), 1575 (vw), 1484 (vw), 1442 (vw), 1234 (vw), 1187 (vw), 1164 (vw), 1110 (w), 1029 (w), 1002 (vs), 727 (vw), 663 (w), 619 (w), 532 (vw), 485 (vw), 366 (vw), 337 (vw), 316 (vw), 283 (vw), 266 (vw), 256 (vw), 231 cm^{-1} (w).

Trisadenosine ultraphosphite (19)

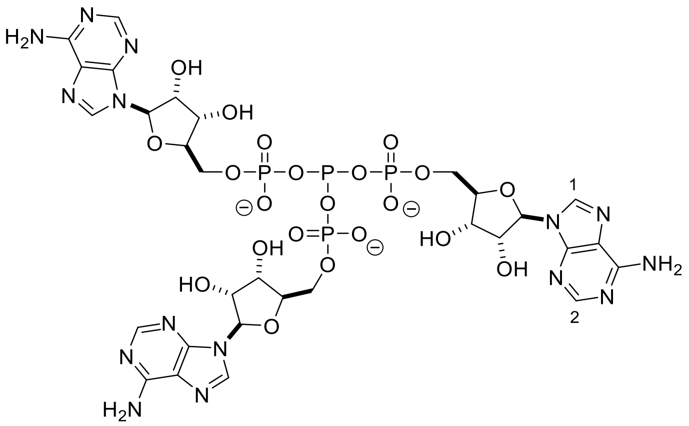

Chemical Formula: $C_{30}H_{36}N_{15}O_{21}P_4^{3-}$
Exact Mass: 1066.1177

AMP · 1.1 TBA (135 mg, 220 µmol, 3.0 eq.) and DCI (26 mg, 220 µmol, 3.0 eq.) were coevaporated using MeCN (3 x 2 ml) and dissolved in DMF (2 ml). $P(NEt_2)_3$ (20 µl, 18 mg, 73 µmol, 1.0 eq.) was added and stirred for 10 min. The product was precipitated using ice-cooled Et_2O (50 ml), the suspension centrifuged and the pellet washed with ice-cooled Et_2O (50 ml). Drying *in vacuo* gave a white solid [1.1 eq. TBA, 2.6 eq. diethylamine, resulting weight: 145 mg, 95 µmol, purity according to ^{31}P{^{1}H}-NMR: 95%, calculated yield over 100% due to residual DCI].

Signals for TBA and diethylamine not indicated: **^{1}H-NMR** (400 MHz, DMF-d_7): δ = 4.19-4.26 (m, 9 H, 3 x 4'-H + 3 x 5'-H_2), 4.56 (dd, *J* = 4.9 Hz, *J* = 3.3 Hz, 3 H, 3 x 3'-H), 4.84 (dd, *J* = 5.3 Hz, 3 H, 3 x 2'-H), 6.14 (d, *J* = 5.6 Hz, 3 H, 3 x 1'-H), 7.28 (s, 6 H, 3 x NH_2), 8.20 (s, 3 H, 3 x 1 H), 8.66 ppm (s, 3 H, 3 x 2-H). **^{31}P{^{1}H}-NMR** (162 MHz, DMF-d_7): δ = –10.09 (s, 3 P), 120.16 ppm (s, 1 P). **^{31}P-NMR** (162 MHz, DMF-d_7): δ = –10.09 (t, *J* = 6.3 Hz, 3 P), 120.16 ppm (s, 1 P).

Trisadenosine ultraphosphate (21)

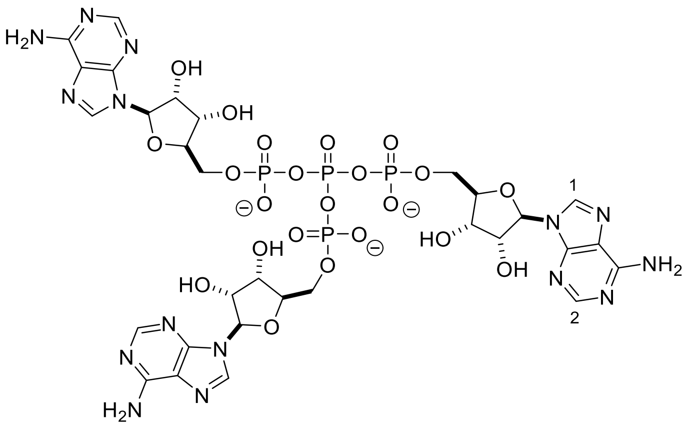

Chemical Formula: $C_{30}H_{36}N_{15}O_{22}P_4^{3-}$
Exact Mass: 1082.1126

AMP · 1.1 TBA (510 mg, 0.84 mmol, 3.0 eq.) and DCI (100 mg, 0.84 mmol, 3.0 eq.) were coevaporated using MeCN (3 x 4 ml) and dissolved in DMF (5 ml). $P(NEt_2)_3$ (77 µl, 70 mg, 0.28 mmol, 1.0 eq.) was added and stirred for 10 min. *m*CPBA (≤77 %, 95 mg, 0.42 µmol, 1.5 eq.) was added at 0°C and stirred for 10 min. The product was precipitated using Et_2O (100 ml), the suspension centrifuged and the pellet washed with Et_2O (100 ml). Drying *in vacuo* gave a white solid (m_{crude} = 462 mg, purity according to $^{31}P\{^1H\}$-NMR: 88%), which was purified by AIEX chromatography (Q Sepharose® Fast Flow, increasing concentrations of NaCl, eluting at 0.22 M buffer concentration, 93.6 µmol, 33%). The ultraphosphate was converted into its [PPN] salt (146 mg, 45.1 µmol, 16% overall yield) according to the general procedure.

Signals for [PPN] not indicated: **^{1}H-NMR** (400 MHz, DMF-d_7): δ = 4.14-4.29 (m, 9 H, 3 x 4'-H + 3 x 5'-H_2), 4.88-4.96 (m, 6 H, 3 x 3'-H + 3 x 2'-H), 5.91 (d, *J* = 3.9 Hz, 3 H, 3 x OH), 6.14 (d, *J* = 5.2 Hz, 3 H, 3 x 1'-H), 6.53 (d, *J* = 3.8 Hz, 3 H, 3 x OH), 7.16 (br. s, 6 H, 3 x NH_2), 8.15 (s, 3 H, 3 x 1-H), 8.98 ppm (s, 3 H, 3 x 2-H). **$^{31}P\{^1H\}$-NMR** (162 MHz, DMF-d_7): δ = –34.82 (q, *J* = 23.1 Hz, 1P), –13.03 ppm (d, *J* = 22.7 Hz, 3 P). **^{31}P-NMR** (162 MHz, DMF-d_7): δ = –34.92 (q, *J* = 23.0 Hz), –13.05 ppm (dt, *J* = 23.0 Hz, *J* = 6.0 Hz, 3 P). **^{13}C-NMR** (101 MHz, DMF-d_7): δ = 65.1 (d, *J* = 6.3 Hz), 70.7, 75.3, 84.8 (d, *J* = 9.0 Hz), 87.0, 119.4, 140.9, 150.7, 153.0, 156.6 ppm. **HRMS (ESI)**: *m/z* calcd for $C_{30}H_{37}N_{15}O_{22}P_4$ $[M - 2 H^+]^{2-}$: 541.5600, found: 541.5598. **Raman:** $\tilde{\nu}$ = 3176 (vw), 3151 (vw), 3141 (vw), 3060 (s), 3012 (vw), 2996 (vw), 2956 (vw), 2946 (vw), 2927 (vw), 2890 (vw), 1589 (s), 1575 (w), 1484 (vw), 1442 (vw), 1378 (vw), 1334 (vw), 1309 (vw), 1295 (vw), 1280 (vw), 1191 (vw), 1164 (vw), 1110 (w), 1083 (vw), 1029 (w), 1000 (vs), 727 (vw), 663 (w), 619 (w), 366 (vw), 335 (vw), 322 (vw), 287 (vw), 268 (vw), 252 (vw), 235 cm^{-1} (w).

Trisadenosine thioultraphosphate (22)

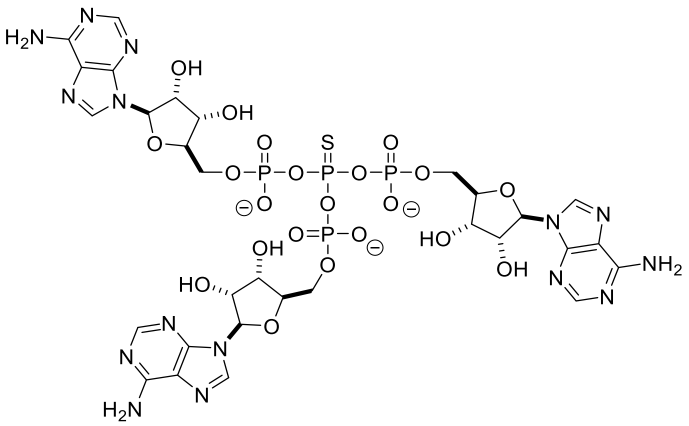

Chemical Formula: $C_{30}H_{36}N_{15}O_{21}P_4S^{3-}$
Exact Mass: 1098.0898

AMP · 1.1 TBA (229 mg, 378 µmol, 3.0 eq.) and DCI (45 mg, 378 µmol, 3.0 eq.) were coevaporated using MeCN (3 x 2 ml) and dissolved in DMF (2.5 ml). $P(NEt_2)_3$ (35 µl, 31 mg, 126 µmol, 1.0 eq.) was added and stirred for 10 min. Sulfur (6 mg, 189 µmol, 1.5 eq.) was added and stirred for 30 min. The product was precipitated using Et_2O (50 ml), the suspension centrifuged and the pellet washed with Et_2O (50 ml). Drying *in vacuo* gave a white solid (m_{crude} = 194 mg, purity according to $^{31}P\{^1H\}$-NMR: 64%), which was purified by AIEX chromatography (Q Sepharose® Fast Flow, increasing concentrations of $NaClO_4$, eluting at 0.11 M buffer concentration).

^{1}H-NMR (400 MHz, D_2O, presat): δ = 4.23-4.29 (m, ambiguous integration due to presat), 4.46-4.55 (m, ambiguous integration due to presat), 5.91 (d, *J* = 5.3 Hz, 3 H), 7.99 (s, 3 H), 8.15 ppm (s, 3 H). **^{31}P{^{1}H}-NMR** (162 MHz, D_2O): δ = −12.57 (d, *J* = 21.0 Hz, 3 P), 23.68 ppm (q, *J* = 21.0 Hz, 1 P). **HRMS (ESI)**: *m/z* calcd for $C_{30}H_{38}O_{21}N_{15}P_4S$ $[M - H^+]^-$: 1100.1043, found: 1100.1052.

Trisadenosine selenoultraphosphate (23)

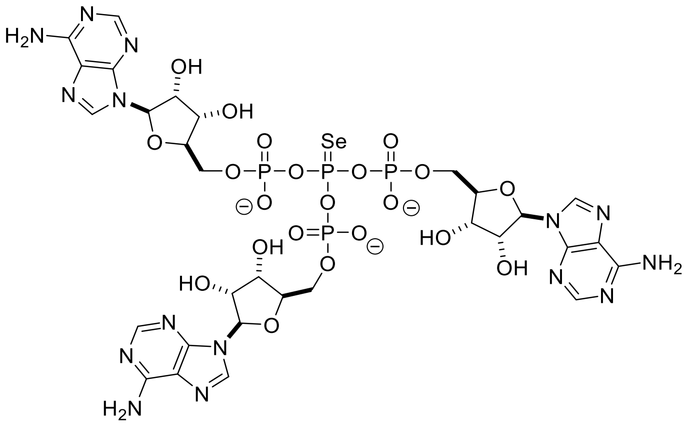

Chemical Formula: $C_{30}H_{36}N_{15}O_{21}P_4Se^{3-}$
Exact Mass: 1146.0342

AMP · 1.1 TBA (125 mg, 204 µmol, 3.0 eq.) and DCI (24 mg, 204 µmol, 3.0 eq.) were coevaporated using MeCN (3 x 2 ml) and dissolved in DMF (2 ml). $P(NEt_2)_3$ (19 µl, 17 mg, 68 µmol, 1.0 eq.) was added and stirred for 10 min. A solution of KSeCN in DMF (100 mg/ml, 2.45 ml, 245 mg, 1.70 mmol, 25.0 eq.) was added,

immediately a solid precipitated and it was stirred at room temp. 2.5 h. Due to precipitation, no full conversion could be reached. The product was precipitated using ice-cooled Et_2O (50 ml), the suspension centrifuged and the pellet washed with ice-cooled Et_2O (50 ml). The crude product (purity according to $^{31}P\{^1H\}$-NMR: 55%, calculated with regard to oxidized products) was dried *in vacuo* and purified by AIEX chromatography (Q Sepharose® Fast Flow, increasing concentrations of LiCl). The product [eluting at 0.25 M buffer concentration] was precipitated using ice-cooled acetone as a white solid.

^{1}H-NMR (400 MHz, D_2O, presat): δ = 4.23-4.31 (m, 9 H, 3 x 4'-H + 3 x 5'-H_2), 4.46 (dd, *J* = 5.2 Hz, 3 H, 3 x 3'-H), 4.57 (dd, *J* = 5.2 Hz, 3 H, 3 x 2'-H), 5.94 (d, *J* = 5.3 Hz, 3 H), 8.03 (s, 3 H), 8.22 ppm (s, 3 H). **^{31}P{^{1}H}-NMR** (162 MHz, D_2O): δ = −12.98 (d, *J* = 24.2 Hz, 3 P), 19.05 ppm (q, *J* = 24.2 Hz, 1 P). **^{31}P-NMR** (162 MHz, D_2O): δ = −12.97 (m, 3 P), 19.04 ppm (q, *J* = 24.2 Hz, 1 P). **HRMS (ESI)**: *m/z* calcd for $C_{30}H_{38}O_{21}N_{15}P_4Se$ [M − H^+]$^-$: 1148.0488, found: 1148.0486.

Trispentynyle ultraphosphate (24)

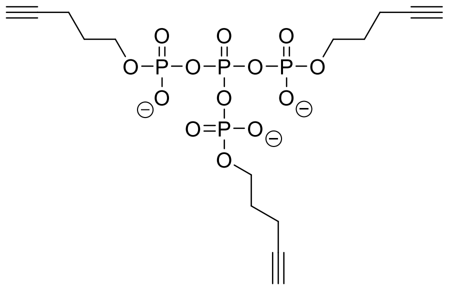

Pentynyle phosphate · 1.2 TBA (239 mg, 533 µmol, 3.0 eq.) and DCI (63 mg, 533 µmol, 3.0 eq.) were coevaporated using MeCN (3 x 3 ml) and dissolved in DMF (2 ml). $P(NEt_2)_3$ (49 µl, 44 mg, 178 µmol, 1.0 eq.) was added and stirred for 10 min. *m*CPBA (≤ 77%, 60 mg, 266 µmol, 1.5 eq.) was added at 0°C and stirred for 10 min. The product was precipitated using ice-cooled Et_2O (50 ml), the suspension centrifuged and the pellet washed with ice-cooled Et_2O (50 ml). Drying *in vacuo* gave a yellowish oil (m_{crude} = 183 mg, purity according to $^{31}P\{^1H\}$-NMR: 60%), which was purified by AIEX chromatography (Q Sepharose® Fast Flow, increasing concentrations of $NaClO_4$, eluting at 0.10 M buffer concentration). The purified product had a purity of 83% according to $^{31}P\{^1H\}$-NMR.

^{1}H-NMR (400 MHz, D_2O, presat): δ = 1.82 (ddt, *J* = 3 x 6.7 Hz, 6 H), 2.25-2.30 (m, 9 H), 4.04 ppm (ddd, *J* = 2 x 6.2 Hz, *J* = 7.5 Hz, 6 H). **^{31}P{^{1}H}-NMR** (162 MHz, D_2O): δ = −36.96 (q, *J* = 19.2 Hz, 1P), −11.63 ppm (d, *J* = 19.9 Hz, 3P). **^{31}P-NMR** (162 MHz, D_2O): δ = −36.36 (q, *J* = 19.2 Hz, 1P), −11.63 ppm (dt, *J* = 19.4 Hz, *J* = 7.2 Hz, 3 P). **HRMS (ESI):** *m/z* calcd for $C_{15}H_{23}O_{13}P_4$ [M − H^+]$^-$: 535.0095, found: 535.0094.

Trispropargyl ultraphosphate (25)

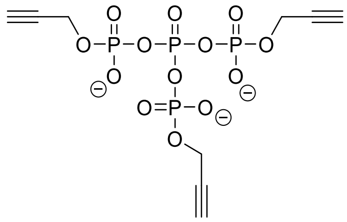

Chemical Formula: $C_9H_9O_{13}P_4^{3-}$
Exact Mass: 448.9010

Propargyl phosphate · 1.0 TBA (200 mg, 532 µmol, 3.0 eq.) and ETT (69 mg, 532 µmol, 3.0 eq.) were coevaporated using MeCN (3 x 3 ml) and dissolved in DMF (3.0 ml). $P(NEt_2)_3$ (49 µl, 44 mg, 177 µmol, 1.0 eq.) was added and stirred for 10 min. *m*CPBA (≤ 77%, 60 mg, 266 µmol, 1.5 eq.) was added at 0°C and stirred for 10 min. The product was precipitated using ice-cooled Et_2O/pentane (5:1, 100 ml), the suspension centrifuged and the pellet washed with ice-cooled Et_2O (100 ml). Drying *in vacuo* gave a yellowish oil (m_{crude} = 212 mg, purity according to $^{31}P\{^1H\}$-NMR: 68%), which was purified by AIEX chromatography (Q Sepharose® Fast Flow, increasing concentrations of NaCl, eluting at 0.4 M buffer concentration). The ultraphosphate was converted into its [PPN] salt according to the general procedure.

Signals for [PPN] not indicated: **^{1}H-NMR** (400 MHz, CD_3CN): δ = 4.59 and 4.61 ppm (each d, *J* = 2.5 Hz, 6 H, 3 x CH_2). **$^{31}P\{^1H\}$-NMR** (162 MHz, CD_3CN): δ = −36.02 (q, *J* = 21.5 Hz, 1 P), −13.09 ppm (d, *J* = 22.7 Hz, 3 P). **^{31}P-NMR** (162 MHz, CD_3CN): δ = −36.02 (q, *J* = 22.2 Hz, 1 P), −13.09 ppm (dt, *J* = 22.2 Hz, *J* = 6.6 Hz, 3 P). **^{13}C-NMR** (101 MHz, CD_3CN): δ = 54.1 (d, *J* = 4.6 Hz), 74.1, 82.5 ppm. **HRMS (ESI)**: *m/z* calcd for $C_9H_{10}O_{13}P_4$ $[M - H^+]^-$: 224.9541, found: 224.9543.

Tris(Fmoc-tyrosine) ultraphosphate (26)

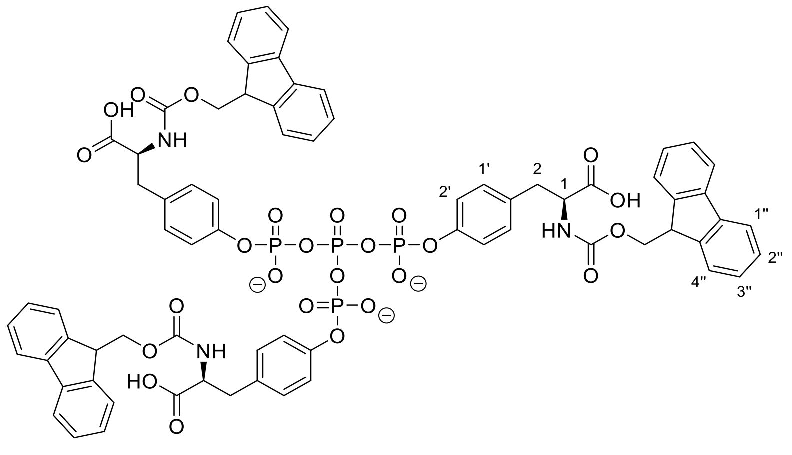

Chemical Formula: $C_{72}H_{60}N_3O_{25}P_4^{3-}$
Exact Mass: 1490.2483

Fmoc-tyrosine phosphate · 1.2 TBA (435 mg, 571 µmol, 3.0 eq.) and DCI (67 mg, 571 µmol, 3.0 eq.) were coevaporated using MeCN (3 x 5 ml) and dissolved in DMF (2 ml). $P(NEt_2)_3$ (52 µl, 47 mg, 190 µmol, 1.0 eq.) was added and stirred for 10 min. *m*CPBA (≤ 77%, 64 mg, 286 µmol, 1.5 eq.) was added at 0°C and stirred for 10 min. The product was precipitated using Et_2O (100 ml), the suspension centrifuged and the pellet washed with Et_2O (100 ml). Drying *in vacuo* gave a white foam (m_{crude} = 329 mg, purity according to $^{31}P\{^1H\}$-NMR: 79%).

Signals for TBA and diethylamine not indicated: **^{1}H-NMR** (300 MHz, DMF-d$_7$): δ = 3.02-3.22 (m, 6 H, 3 x 2-H$_2$), 4.24 (br. s, 6 H, 3 x Fmoc-CH$_2$), 4.34-4.42 (m, 3 H, 3 x 1-H), 7.20-7.27 (m, 12 H, 6 x 2''-H + 6 x 3''-H), 7.31-7.45 (m, 12 H, 6 x 1'-H + 6 x 2'-H), 7.74 (d, *J* = 7.4 Hz, 6 H, 6 x 4''-H), 7.90 (d, *J* = 7.5 Hz, 6 H, 6 x 1''-H) ppm. **^{31}P{^{1}H}-NMR** (121 MHz, DMF-d$_7$): δ = −33.51 (q, *J* = 16.0 Hz, 1 P, 1-P), −17.99 ppm (d, *J* = 16.5 Hz, 3 P, 3 x 2-P). **HRMS (ESI):** *m/z* calcd for $C_{72}H_{63}N_3NaO_{25}P_4$ [M + Na$^+$]$^+$: 1516.25933, found: 1516.26442.

Tristyrosine ultraphosphate (27)

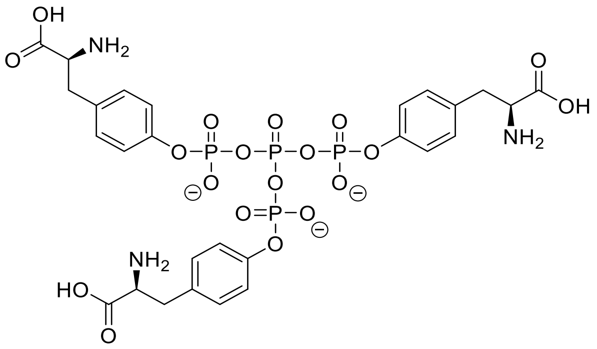

Chemical Formula: $C_{27}H_{30}N_3O_{19}P_4^{3-}$
Exact Mass: 824.0440

Fmoc-Tyr-ultraphosphate · 1.8 TBA · 2.7 $HNEt_2$ (160 mg, 75 µmol, 1.0 eq.) was dissolved in a DBU solution in DMF (5%, 3.0 ml, 1.00 mmol, 13.3 eq.) and stirred for 15 min. The solution became yellowish and the product was precipitated using ice-cooled Et_2O (50 ml) and washed with ice-cooled Et_2O (50 ml). Drying *in vacuo* gave a yellowish solid (purity according to ^{31}P{^{1}H}-NMR: 62%) which was purified by AIEX chromatography (Q Sepharose® Fast Flow, increasing concentrations of $NaClO_4$, eluting at 0.15 M buffer concentration).

^{1}H-NMR (400 MHz, D_2O, presat): δ = 2.72 (dd, *J* = 14.0 Hz, *J* = 8.4 Hz, 3 H), 2.99 (dd, *J* = 14.0 Hz, *J* = 5.0 Hz, 3 H), 3.51 (dd, *J* = 8.4 Hz, *J* = 4.9 Hz, 3 H), 7.05-7.14 ppm (m, 12 H, aromatic). **^{31}P{^{1}H}-NMR** (162 MHz, D_2O): δ = −37.31 (q, *J* = 19.4 Hz, 1 P), −17.05 ppm (d, *J* = 19.2 Hz, 3 P). **HRMS (ESI):** *m/z* calcd for $C_{27}H_{32}N_3O_{19}P_4$ [M − H$^+$]$^-$: 826.0586, found: 826.0632.

Tris(D-glucose-6) ultraphosphate (28)

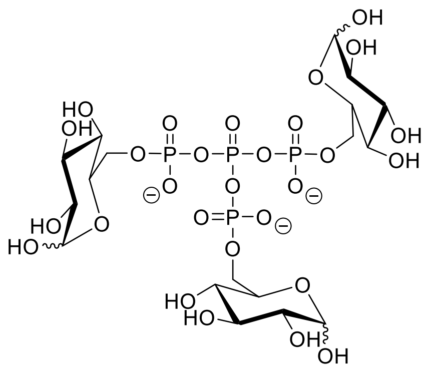

Chemical Formula: $C_{18}H_{33}O_{28}P_4^{3-}$
Exact Mass: 821.0125

D-Glucose-6-phosphate · 1.1 TBA (195 mg, 370 µmol, 3.0 eq.) and DCI (44 mg, 370 µmol, 3.0 eq.) were coevaporated using MeCN (3 x 3 ml) and dissolved in DMF (3 ml). $P(NEt_2)_3$ (34 µl, 31 mg, 123 µmol, 1.0 eq.)

was added and stirred for 10 min. *m*CPBA (≤ 77%, 42 mg, 185 µmol, 1.5 eq.) was added at 0°C and stirred for 10 min. The product was precipitated using ice-cooled Et_2O (50 ml), the suspension centrifuged and the pellet washed with ice-cooled Et_2O (50 ml). Drying *in vacuo* gave a white solid (m_{crude} = 144 mg, purity according to $^{31}P\{^1H\}$-NMR: 61%), which was purified by AIEX chromatography (Q Sepharose® Fast Flow, increasing concentrations of $NaClO_4$). The product [eluting at 0.07 M buffer concentration] was precipitated using ice-cooled $NaClO_4$ solution in acetone (0.5 M, product fraction/$NaClO_4$ solution: 1:9 v/v) as a white solid. The purified product had a purity of 90% according to $^{31}P\{^1H\}$-NMR.

^{1}H-NMR (400 MHz, D_2O, presat): δ = signals in the range of 3.18-4.27, 4.60 and 5.16 ppm. **$^{31}P\{^1H\}$-NMR** (162 MHz, D_2O): δ = −36.31 (q, *J* = 18.7 Hz, 1P), −11.68 and −11.63 ppm (each d, *J* = 18.8 Hz, together 3 P). **^{31}P-NMR** (162 MHz, D_2O): δ = −36.31 (q, *J* = 18.7 Hz), −11.68 and −11.63 ppm (each dt, *J* = 18.6 Hz, *J* = 7.3 Hz, together 3 P). **HRMS (ESI):** *m/z* calcd for $C_{18}H_{35}O_{28}P_4$ $[M - H^+]^-$: 823.0271, found: 823.0251.

Tristhiamine ultraphosphate BArF salt (29)

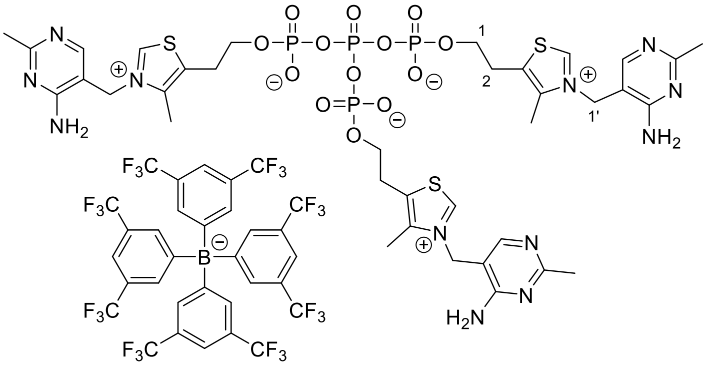

Thiamine monophosphate · 1.4 BArF (350 mg, 218 µmol, 3.0 eq.) and DCI (26 mg, 220 µmol, 3.0 eq.) were coevaporated using MeCN (3 x 2 ml) and dissolved in DMF (2 ml). $P(NEt_2)_3$ (20 µl, 18 mg, 73 µmol, 1.0 eq.) was added and stirred for 10 min. *m*CPBA (≤ 77%, 25 mg, 112 µmol, 1.5 eq.) was added at 0°C and stirred for 10 min. The product was precipitated using Et_2O (50 ml), the suspension centrifuged and the pellet washed with Et_2O (50 ml). Drying *in vacuo* gave a white solid (0.7 eq. BArF, 0.5 eq. diethylamine, m_{crude} = 151 mg, purity according to $^{31}P\{^1H\}$-NMR: 72%, 63 µmol, 86%).

Signals for BArF and diethylamine not indicated: **^{1}H-NMR** (500 MHz, CD_3CN): δ = 2.44 (s, 9 H, 3 x CH_3), 2.45 (s, 9 H, 3 x CH_3), 3.22 (t, $J_{2,1}$ = 5.6 Hz, 6 H, 3 x 2-CH_2), 4.13 (dt, $J_{1,2}$ = 5.9 Hz, $J_{1,P}$ = 6.1 Hz, 6 H, 3 x 1-CH_2), 5.34 (s, 6 H, 3 x 1'-CH_2), 7.50 (s, 3 H, 3 x pyrimidine-H), 7.91 ppm (s, 3 H, 3 x thiazole-H). **$^{31}P\{^1H\}$-NMR** (202 MHz, CD_3CN): δ = −37.16 (q, *J* = 19.9 Hz, 1 P), −12.80 ppm (d, *J*= 19.9 Hz, 3 P). **^{31}P-NMR** (202 MHz, CD_3CN): δ = −37.15 (q, *J* = 19.9 Hz, 1 P), −12.80 ppm (dt, *J*= 20.3 Hz, *J*= 6.9 Hz, 3 P). **HRMS (ESI):** *m/z* calcd for $C_{36}H_{49}N_{12}O_{13}P_4S_3$ $[M - 2 H^+]^+$: 1077.16491, found: 1077.16134.

Trisbenzyl ultraphosphate (30)

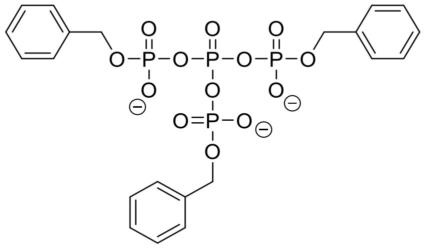

Chemical Formula: $C_{21}H_{21}O_{13}P_4^{3-}$
Exact Mass: 604.9949

Benzyl phosphate · 1.2 TBA (500 mg, 1.05 mmol, 3.0 eq.) and ETT (136 mg, 1.05 mmol, 3.0 eq.) were coevaporated using MeCN (3 x 3 ml) and dissolved in DMF (4 ml). $P(NEt_2)_3$ (96 µl, 86 mg, 349 µmol, 1.0 eq.) was added and stirred for 10 min. *m*CPBA (≤ 77%, 117 mg, 524 µmol, 1.5 eq.) was added at 0°C and stirred for 10 min. The product was precipitated using ice-cooled Et_2O/pentane (5:1, 100 ml), the suspension centrifuged and the pellet washed with ice-cooled Et_2O (2 x 100 ml). Drying *in vacuo* gave a colourless oil (m_{crude} = 593 mg, purity according to $^{31}P\{^1H\}$-NMR: 63%), which was purified by AIEX chromatography (Q Sepharose® Fast Flow, increasing concentrations of NaCl, eluting at 0.7 M buffer concentration). The ultraphosphate was converted into its [PPN] salt according to the general procedure.

Signals for [PPN] not indicated: **^{1}H-NMR** (400 MHz, CD_3CN): δ = 4.97 (d, *J* = 6.4 Hz, 6 H, 3 x CH_2), 7.18 (m, 3 H), 7.25 (m, 6 H), 7.39 ppm (m, 6 H). **$^{31}P\{^1H\}$-NMR** (162 MHz, CD_3CN): δ = −36.25 (q, *J* = 23.0 Hz, 1 P), −12.24 ppm (d, *J* = 23.0 Hz, 3 P). **^{31}P-NMR** (162 MHz, CD_3CN): δ = −36.25 (q, *J* = 23.0 Hz, 1 P), −12.24 ppm (dt, *J* = 23.0 Hz, *J* = 6.5 Hz, 3 P). **^{13}C-NMR** (101 MHz, CD_3CN): δ = 67.8 (d, *J* = 5.8 Hz), 127.6, 128.5, 128.9, 141.4 ppm (d, *J* = 9.2 Hz). **HRMS (ESI)**: *m/z* calcd for $C_{21}H_{23}O_{13}P_4$ $[M - H^+]^-$: 607.0095, found: 607.0099.

Tris(*para*-nitrophenyl) ultraphosphate (31)

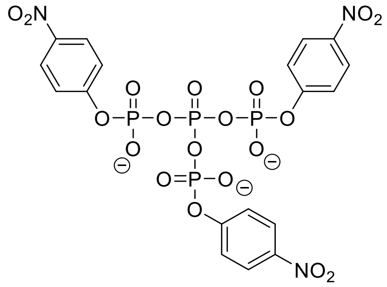

Chemical Formula: $C_{18}H_{12}N_3O_{19}P_4^{3-}$
Exact Mass: 697.9032

para-Nitrophenyl phosphate · 1.2 TBA (650 mg, 1.30 mmol, 3.0 eq.) and ETT (169 mg, 1.30 mmol, 3.0 eq.) were coevaporated using MeCN (3 x 6 ml) and dissolved in DMF (6 ml). $P(NEt_2)_3$ (119 µl, 107 mg, 0.43 mmol, 1.0 eq.) was added and stirred for 10 min. *m*CPBA (≤ 77%, 146 mg, 0.65 mmol, 1.5 eq.) was added at 0°C and stirred for 10 min. The product was precipitated using ice-cooled Et_2O/pentane (5:1, 200 ml), the suspension centrifuged and the pellet washed with ice-cooled Et_2O (200 ml). Drying *in vacuo* gave a yellowish oil (m_{crude} = 779 mg, purity according to $^{31}P\{^1H\}$-NMR: 45%), which was purified by AIEX chromatography (Q Sepharose® Fast Flow, increasing concentrations of NaCl, eluting at 0.85 M buffer concentration). The ultraphosphate was converted into its [PPN] salt according to the general procedure.

Signals for [PPN] not indicated: **^{1}H-NMR** (400 MHz, CD_3CN): δ = 7.45 (m_c, 6 H), 7.96 ppm (m_c, 6 H). **^{31}P{^{1}H}-NMR** (162 MHz, CD_3CN): δ = −37.51 (q, *J* = 21.9 Hz, 1 P), −19.52 ppm (d, *J* = 21.9 Hz, 3 P). **^{13}C-NMR** (101 MHz, CD_3CN): δ = 122.0 (d, *J* = 5.6 Hz), 125.7, 143.3, 160.7 ppm (d, *J* = 6.8 Hz). **HRMS (ESI)**: *m/z* calcd for $C_{18}H_{13}O_{19}N_3P_4$ [M − 2 H$^+$]$^{2-}$: 349.4552, found: 349.4552.

1,2,4-Tris(*para*-nitrobenzyl)-3-oxo-tetraphosphonate (64)

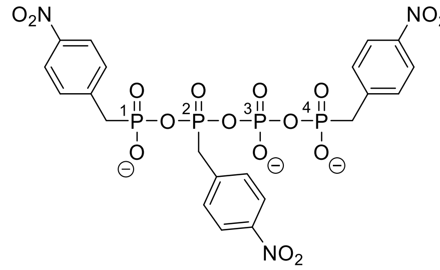

Chemical Formula: $C_{21}H_{18}N_3O_{16}P_4^{3-}$
Exact Mass: 691.9654

The product was isolated as a side-product of the attempted synthesis of tris(*para*-nitrobenzyl phosphonyl) ultraphosphate (**S6**) and purified by AIEX chromatography (Q Sepharose® Fast Flow, increasing concentrations of NaCl, eluting at 0.45 M buffer concentration).

^{1}H-NMR (400 MHz, D_2O, presat): δ = 3.15 (d, *J* = 22.2 Hz, 2 H), 3.18 (d, *J* = 22.2 Hz, 2 H), 3.44 (two d overlaying, each *J* = 23.4 Hz, 2 H), 7.26 (m, 2 H), 7.37 (m, 4 H) 7.96 ppm (m, 6 H). **^{31}P{^{1}H}-NMR** (162 MHz, D_2O): δ = −23.79 (dd, *J* = 25.3 Hz, *J* = 20.7 Hz, 1 P, 3-P), 7.21 (dd, *J* = 32.9 Hz, *J* = 20.8 Hz, 1 P, 2-P), 11.87 (d, *J* = 25.2 Hz, 1 P, 4-P), 13.99 ppm (d, *J* = 32.9 Hz, 1 P, 1-P). **^{31}P-NMR** (162 MHz, D_2O): δ = −23.80 (dd, *J* = 25.4 Hz, *J* = 20.8 Hz, 1 P, 3-P), 7.15 (m, 1 P, 2-P), 11.87 (dt, *J* = 25.2 Hz, *J* = 23.4 Hz, 1 P, 4-P), 13.99 ppm (dt, *J* = 32.9 Hz, *J* = 23.4 Hz, 1 P, 1-P). **HRMS (ESI)**: *m/z* calcd for $C_{21}H_{18}O_{16}N_3P_4Na_2$ [M − 3 H$^+$, + 2 Na$^+$]$^-$: 737.9438, found: 737.9442.

Tris(2-nitrobenzyl) ultraphosphate (32)

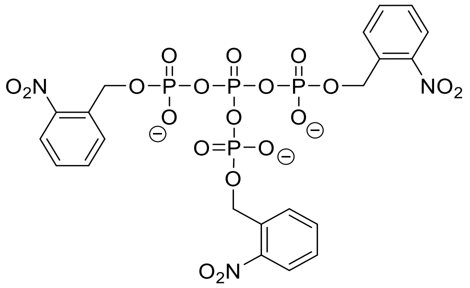

Chemical Formula: $C_{21}H_{18}N_3O_{19}P_4^{3-}$
Exact Mass: 739.9501

2-Nitrobenzyl phosphate (134 mg, 575 µmol, 3.0 eq.) and ETT (75 mg, 575 µmol, 3.0 eq.) were coevaporated using MeCN (3 x 2.5 ml) and dissolved in DMF (2.5 ml). $P(NEt_2)_3$ (53 µl, 48 mg, 192 µmol, 1.0 eq.) was added and stirred for 15 min. *m*CPBA (≤ 77%, 50 mg, 288 µmol, 1.5 eq.) was added at 0°C and stirred for 10 min. The product was precipitated using ice-cooled Et_2O (50 ml), the suspension centrifuged and the pellet washed with ice-cooled Et_2O (50 ml). Drying *in vacuo* gave a brownish oil (m_{crude} = 156 mg, purity according to ^{31}P{^{1}H}-NMR: 70%), which was purified by AIEX chromatography (Q Sepharose® Fast Flow, increasing concentrations of $NaClO_4$). The product [eluting at 0.55 M buffer concentration] was precipitated using ice-cooled $NaClO_4$ solution in acetone (0.5 M, product fraction/$NaClO_4$ solution: 1:9 v/v) as a white solid.

^{1}H-NMR (400 MHz, D_2O, presat): δ = 5.22 (d, *J* = 6.5 Hz, 6 H,3 x CH_2), 7.35 (m, 3 H), 7.57 (m, 3 H), 7.79 (dd, *J* = 7.9 Hz, 1.3 Hz, 3 H), 7.93 ppm (dd, *J* = 8.2 Hz, 1.2 Hz, 3 H). **^{31}P{^{1}H}-NMR** (162 MHz, D_2O): δ = −36.30 (q, *J* = 19.1 Hz, 1P), −12.12 ppm (d, *J* = 19.1 Hz, 3 P). **^{31}P-NMR** (162 MHz, D_2O): δ = −36.30 (q, *J* = 19.0 Hz, 1 P), −12.13 ppm (dt, *J* = 19.1 Hz, *J* = 6.6 Hz, 3 P). **HRMS (ESI)**: *m/z* calcd for $C_{21}H_{20}O_{19}N_3P_4$ [M − H^+]$^-$: 741.9647, found: 741.9652.

Tris(2-nitrobenzyl) thioultraphosphate (33)

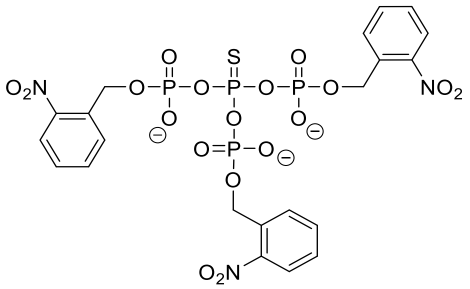

Chemical Formula: $C_{21}H_{18}N_3O_{18}P_4S^{3-}$
Exact Mass: 755.9273

2-Nitrobenzyl phosphate (121 mg, 519 µmol, 3.0 eq.) and DCI (61 mg, 519 µmol, 3.0 eq.) were coevaporated using MeCN (3 x 4 ml) and dissolved in DMF (3 ml). $P(NEt_2)_3$ (47 µl, 43 mg, 173 µmol, 1.0 eq.) was added and stirred for 10 min. Sulfur (8.5 mg, 260 µmol, 1.5 eq.) was added and stirred for 30 min. The product was precipitated using ice-cooled Et_2O (100 ml), the suspension centrifuged and the pellet washed with ice-cooled Et_2O (100 ml). Drying *in vacuo* gave a brownish oil (m_{crude} = 128 mg, purity according to ^{31}P{^{1}H}-NMR: 26%), which was purified by AIEX chromatography (Q Sepharose® Fast Flow, increasing concentrations of $NaClO_4$, eluting at 0.65 M buffer concentration). The purified product had a purity of 81% according to ^{31}P{^{1}H}-NMR.

^{1}H-NMR (400 MHz, D_2O, presat): δ = 5.22 (d, *J* = 6.4 Hz, 6 H, 3 x CH_2), 7.35 (m, 3 H), 7.56 (m, 3 H), 7.68 (m, 3 H), 7.94 ppm (m, 3 H). **^{31}P{^{1}H}-NMR** (162 MHz, D_2O): δ = −12.57 (d, *J* = 22.5 Hz, 3 P), 23.62 ppm (q, *J* = 22.2 Hz, 1 P). **^{31}P-NMR** (162 MHz, D_2O): δ = −12.58 (dt, *J* = 22.4 H, *J* = 6.5 Hz, 3 P), 23.62 ppm (q, *J* = 22.2 Hz, 1 P). **HRMS (ESI)**: *m/z* calcd for $C_{21}H_{20}O_{18}N_3P_4S$ [M − H^+]$^-$: 757.9419, found: 757.9426.

Tris(1-(2-nitrophenyl)ethyl) ultraphosphate (34)

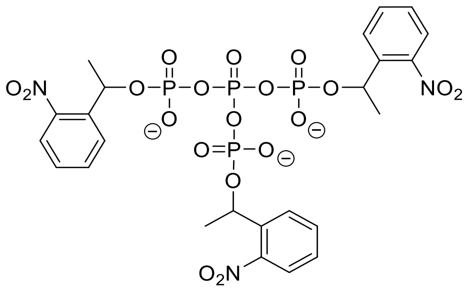

Chemical Formula: $C_{24}H_{24}N_3O_{19}P_4^{3-}$
Exact Mass: 781.9971

1-(2-Nitrophenyl)ethyl phosphate (805 mg, 3.26 mmol, 3.0 eq.) and ETT (425 mg, 3.26 mmol, 3.0 eq.) were coevaporated using MeCN (3 x 7 ml) and dissolved in DMF (7 ml). $P(NEt_2)_3$ (297 µl, 269 mg, 1.09 mmol,

1.0 eq.) was added and stirred for 10 min. *m*CPBA (≤ 77%, 281 mg, 1.63 mmol, 1.5 eq.) was added at 0°C and stirred for 10 min. The product was precipitated using ice-cooled Et_2O (200 ml), the suspension centrifuged and the pellet washed with ice-cooled Et_2O (200 ml). Drying *in vacuo* gave a brownish solid (m_{crude} = 669 mg, purity according to $^{31}P\{^1H\}$-NMR: 45%), which was purified by AIEX chromatography (Q Sepharose® Fast Flow, increasing concentrations of $NaClO_4$, eluting at 0.6 M buffer concentration).

^{1}H-NMR (400 MHz, D_2O, presat): δ = 1.47-1.53 (m, 9 H, 3 x CH_3), 5.87-5.96 (m, 3 H,3 x CH), 7.43-7.49 (m, 3 H), 7.67-7.74 (m, 3 H), 7.77-7.83 (m, 3 H), 7.93-8.00 ppm (m 1.2 Hz, 3 H). **^{31}P{^{1}H}-NMR** (162 MHz, D_2O): δ = −36.26 (q, *J* = 17.3 Hz, 1P), −13.08 and −13.10 ppm (each d, *J* = 17.8 Hz, together 3 P). **^{31}P-NMR** (162 MHz, D_2O): δ = −36.27 (q, *J* = 17.8 Hz,1 P), −13.09 and −13.11 ppm (each dd, *J* = 17.3 Hz, *J* = 8.3 Hz, together 3 P). **HRMS (ESI)**: *m/z* calcd for $C_{24}H_{26}O_{19}N_3P_4$ $[M - H^+]^-$: 784.0116, found: 784.0125.

Tris(1-(2-nitrophenyl)ethyl) thioultraphosphate (35)

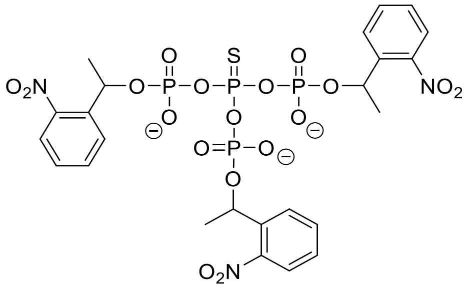

Chemical Formula: $C_{24}H_{24}N_3O_{18}P_4S^{3-}$
Exact Mass: 797.9742

1-(2-Nitrophenyl)ethyl phosphate (144 mg, 583 µmol, 3.0 eq.) and DCI (69 mg, 583 µmol, 3.0 eq.) were coevaporated using MeCN (3 x 3 ml) and dissolved in DMF (2 ml). $P(NEt_2)_3$ (53 µl, 48 mg, 194 µmol, 1.0 eq.) was added and stirred for 10 min. Sulfur (9.0 mg, 291 µmol, 1.5 eq.) was added and stirred for 10 min. The product was precipitated using ice-cooled Et_2O (100 ml), the suspension centrifuged and the pellet washed with ice-cooled Et_2O (100 ml). Drying *in vacuo* gave a brownish solid (m_{crude} = 72 mg, purity according to $^{31}P\{^1H\}$-NMR: 28%), which was purified by AIEX chromatography (Q Sepharose® Fast Flow, increasing concentrations of $NaClO_4$, eluting at 0.7 M buffer concentration). The purified product had a purity of 85% according to $^{31}P\{^1H\}$-NMR.

^{1}H-NMR (500 MHz, D_2O, presat): δ = 1.53 (m, 9 H, 3 x CH_3), 5.91-6.00 (m, 3 H, 3 x CH), 7.48 (m, 3 H), 7.73 (m, 3 H), 7.85 (m, 3 H), 8.00 ppm (m, 3 H). **^{31}P{^{1}H}-NMR** (202 MHz, D_2O): δ = −13.72 (m, 3 P), 23.02 ppm (q, *J* = 21.0 Hz, 1 P). **^{31}P-NMR** (202 MHz, D_2O): δ = −13.74 (m, 3 P), 23.02 ppm (q, *J* = 20.7 Hz, 1 P). **HRMS (ESI)**: *m/z* calcd for $C_{24}H_{26}O_{18}N_3P_4S$ $[M - H^+]^-$: 799.9888, found: 799.9897.

Tris-DEACM ultraphosphate (36)

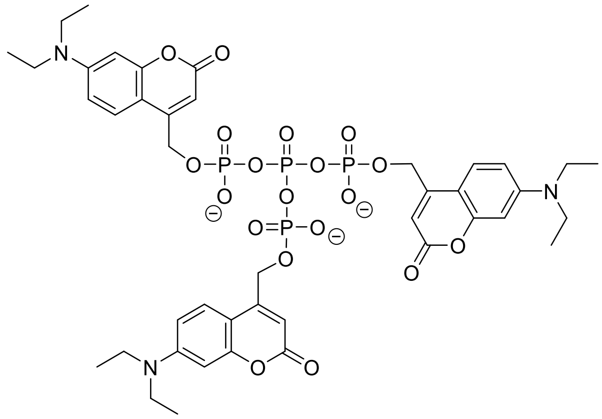

Chemical Formula: $C_{42}H_{48}N_3O_{19}P_4^{3-}$
Exact Mass: 1022.1849

DEACM-phosphate · 1.2 TBA (45% with unclear salt composition, 665 mg, 485 µmol, 3.0 eq.) and DCI (57 mg, 485 µmol, 3.0 eq.) were coevaporated using MeCN (3 x 5 ml) and dissolved in DMF (5 ml). $P(NEt_2)_3$ (44 µl, 40 mg, 162 µmol, 1.0 eq.) was added and stirred for 10 min. *m*CPBA (≤ 77%, 54 mg, 242 µmol, 1.5 eq.) was added at 0°C and stirred for 10 min. The product was precipitated using ice-cooled Et_2O (100 ml), the suspension centrifuged and the pellet washed with ice-cooled Et_2O (100 ml). Drying *in vacuo* gave a yellow solid (m_{crude} = 398 mg, purity according to $^{31}P\{^1H\}$-NMR: 55%), which was purified by AIEX chromatography (Q Sepharose® Fast Flow, increasing concentrations of $NaClO_4$, eluting at 0.8 M buffer concentration).

^{1}H-NMR (400 MHz, D_2O, presat): δ = 1.08 (t, *J* = 6.9 Hz, 18 H, 6 x CH_3), 3.24 (q, *J* = 7.1 Hz, 12 H, 6 x CH_2), P-CH_2- signal not resolved due to low product and high water concentration, 5.92 (d, *J* = 4.8 Hz, 3 H), 6.06 (dd, *J* = 6.4 Hz, 2.5 Hz, 3 H), 6.34 (d, *J* = 9.5 Hz, 3 H), 6.90 ppm (dd, *J* = 9.1 Hz, 2.7 Hz, 3 H). **$^{31}P\{^1H\}$-NMR** (162 MHz, D_2O): δ = −35.93 (m, 1P), −12.08 ppm (m, 3 P). **^{31}P-NMR** (162 MHz, D_2O): δ = −35.93 (m, 1P), −12.08 ppm (m, 3 P). **HRMS (ESI)**: *m/z* calcd for $C_{42}H_{50}O_{19}N_3P_4$ $[M - H^+]^-$: 1024.1994, found: 1024.1995.

Tris(9*H*-fluoren-9-yl)methyl ultraphosphate (37)

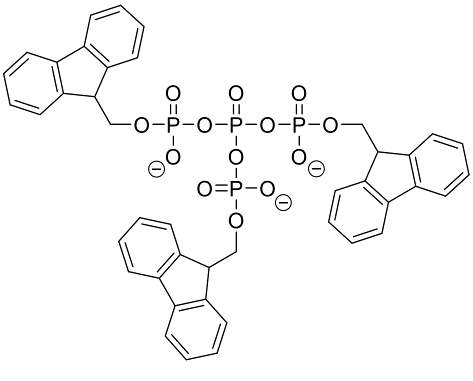

Chemical Formula: $C_{42}H_{33}O_{13}P_4^{3-}$
Exact Mass: 869.0888

(9*H*-Fluoren-9-yl)methyl dihydrogen phosphate (1.26 g, 4.55 mmol, 3.0 eq.) and DCI (537 mg, 4.55 mmol, 3.0 eq.) were coevaporated using MeCN (3 x 8 ml) and dissolved in DMF (12 ml). $P(NEt_2)_3$ (415 µl, 375 mg,

1.52 mmol, 1.0 eq.) was added and stirred for 10 min. *m*CPBA (≤77%, 509 mg, 2.27 mmol, 1.5 eq.) was added at 0°C and stirred for 10 min. The product was precipitated using ice-cooled Et_2O (200 ml), the suspension centrifuged and the pellet washed with ice-cooled Et_2O (200 ml). Drying *in vacuo* gave a colourless, sticky oil (m_{crude} = 1.49 g, purity according to $^{31}P\{^1H\}$-NMR: 73%), which was purified using a PuriFlash Column (30µ C18 AQ; water, MeCN gradient (0-45%), 10% TEAA (100 mM, pH 7.0)).

^{1}H-NMR (400 MHz, D_2O, presat): δ = 4.12 (m, 9 H, 3 x CH + 6 x CH_2), 7.22 (ddd, *J* = 7.5 Hz, *J* = 7.5 Hz, *J* = 1.1 Hz, 6 H), 7.40 (ddd, *J* = 7.5 Hz, *J* = 7.5 Hz, *J* = 1.0 Hz, 6 H), 7.60 (d, *J* = 7.5 Hz, 6 H), 7.80 ppm (d, *J* = 7.6 Hz, 6 H). **^{31}P{^{1}H}-NMR** (162 MHz, D_2O): δ = −35.87 (q, *J* = 18.3 Hz, 1 P), −11.91 ppm (d, *J* = 18.3 Hz, 3 P). **^{31}P-NMR** (162 MHz, D_2O): δ = −35.86 (q, *J* = 18.6 Hz, 1 P), −11.92 ppm (dt, *J* = 18.0 Hz, *J* = 7.0 Hz, 3 P). **^{13}C-NMR** (101 MHz, D_2O): δ = 47.9 (d, *J* = 7.3 Hz, CH_2), 68.7 (d, *J* = 6.6 Hz, CH), 120.0, 125.6, 127.4, 127.8, 140.9, 143.8 ppm. **HRMS (ESI)**: *m/z* calcd for $C_{42}H_{35}O_{13}P_4$ [M − H$^+$]$^-$: 871.1034, found: 871.1038. **Raman:** $\tilde{\nu}$ =3095 (vw), 3062 (m), 2966 (vw), 2919 (w), 2892 (vw), 2765 (vw), 2753 (vw), 1704 (vw), 1612 (s), 1589 (m), 1577 (w), 1483 (vw), 1344 (vw), 1317 (vw), 1297 (w), 1236 (vw), 1224 (vw), 1186 (vw), 1159 (vw), 1110 (w), 1074 (vw), 1027 (m), 1000 (vs), 792 (vw), 744 (vw), 728 (vw), 665 (w), 617 (vw), 416 (vw), 302 (vw), 266 (vw), 235 cm^{-1} (w).

Ultraphosphate (2)

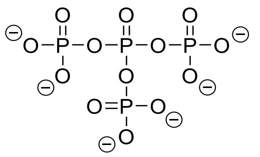

Chemical Formula: $O_{13}P_4^{6-}$
Exact Mass: 331.8322

A 10 % solution of DBU in water (270 µl) was added to tris(9*H*-fluoren-9-yl)methyl ultraphosphate (6.0 mM in water/MeCN, 10 mM TEAA; 270 µl, 1.62 µmol, 1.0 eq.) and stirred for 30-40 min. The product (purity according to $^{31}P\{^1H\}$-NMR: 98%) could not be isolated.

^{31}P-NMR (162 MHz, D_2O): δ = −35.50 (q, *J* = 22.1 Hz, 1 P), −5.31 ppm (d, *J* = 22.1 Hz, 3 P). **HRMS (ESI)**: *m/z* calcd for $H_5O_{13}P_4$ [M − H$^+$]$^-$: 336.8686, found: 336.8687.

Ultraphosphate [PPN] salt (2 $^{[PPN]}$)

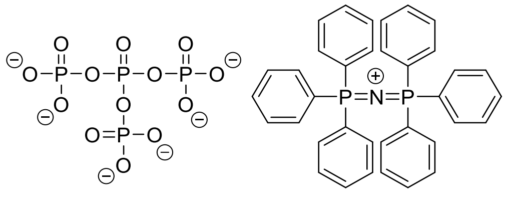

A 10 % solution of DBU in water (1.0 ml) was added to tris(9*H*-fluoren-9-yl)methyl ultraphosphate (4.9 mM in water/MeCN, 10 mM TEAA; 1.0 ml, 4.90 µmol, 1.0 eq.) and stirred for 40 min. The mixture was diluted with water (4 ml) and centrifuged to remove the deprotection product. A solution of [PPN]-Cl (16.9 mg, 29.4 µmol, 6.0 eq.) in a mixture of acetone and water (9:1, 200 µl) was added. A solid/oil formed which dissolved again upon rigorous stirring (vortex centrifuge tube). The product was allowed to precipitate from the supernatant by diluting the mixture with water (6 ml) and cooling in an ice-bath. The solid was collected by centrifugation and washed with water twice. Purity according to $^{31}P\{^1H\}$-NMR: 98%.

Signals for [PPN] counter-ion are not indicated: **^{1}H-NMR** (400 MHz, CD_3CN): Only solvent signals and small impurities of dibenzofulvene at δ = 7.34, 7.41, 7.77, 7.82 ppm. **^{31}P-NMR** (162 MHz, CD_3CN): δ = –35.54 (q, *J* = 22.4 Hz, 1 P), –5.42 ppm (d, *J* = 22.0 Hz, 3 P).

Tris(9*H*-fluoren-9-yl)methyl thioultraphosphate (38)

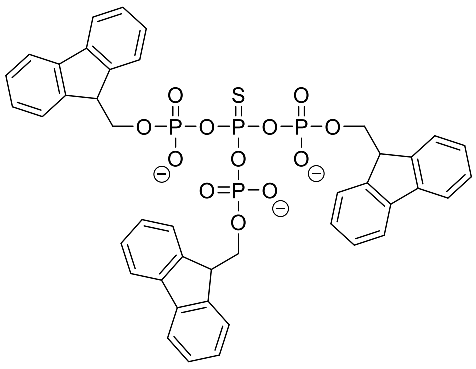

Chemical Formula: $C_{42}H_{33}O_{12}P_4S^{3-}$
Exact Mass: 885.0660

(9*H*-Fluoren-9-yl)methyl dihydrogen phosphate (205 mg, 743 µmol, 3.0 eq.) and DCI (88 mg, 743 µmol, 3.0 eq.) were coevaporated using MeCN (3 x 3 ml) and dissolved in DMF (2.5 ml). $P(NEt_2)_3$ (68 µl, 61 mg, 248 µmol, 1.0 eq.) was added and stirred for 10 min. Sulfur (12 mg, 371 µmol, 1.5 eq.) was added and stirred for 30 min. The product was precipitated using ice-cooled Et_2O (100 ml), the suspension centrifuged and the pellet washed with ice-cooled Et_2O (100 ml). Drying *in vacuo* gave a white solid (purity according to ^{31}P{^{1}H}-NMR: 50%), which was purified using a PuriFlash Column (30µ C18 AQ; water, MeCN gradient (0-45%), 10% TEAA (100 mM, pH 7.0)).

^{1}H-NMR (400 MHz, D_2O, presat): δ = 4.14 (m, 9 H, 3 x CH + 6 x CH_2), 7.20 (ddd, *J* = 7.6 Hz, *J* = 7.6 Hz, *J* = 1.0 Hz, 6 H), 7.39 (ddd, *J* = 7.6 Hz, *J* = 7.6 Hz, *J* = 1.0 Hz, 6 H), 7.60 (d, *J* = 7.5 Hz, 6 H), 7.79 ppm (d, *J* = 7.6 Hz, 6 H). **^{31}P{^{1}H}-NMR** (162 MHz, D_2O): δ = –12.36 (d, *J* = 20.1 Hz, 1 P), 23.74 ppm (q, *J* = 20.1 Hz, 3 P). **^{31}P-NMR** (162 MHz, D_2O): δ = –12.36 (dt, *J* = 21.4 Hz, *J* = 7.2 Hz, 1 P), 23.74 ppm (q, *J* = 20.2 Hz, 3 P). **HRMS (ESI)**: *m/z* calcd for $C_{42}H_{35}O_{12}P_4S$ $[M - H^+]^-$: 887.0805, found: 887.0799.

Thioultraphosphate (15)

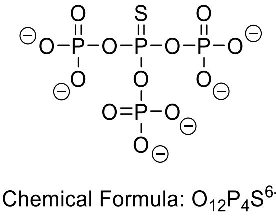

Chemical Formula: $O_{12}P_4S^{6-}$
Exact Mass: 347.8094

A 10 % solution of DBU in water (270 µl) was added to tris(9*H*-fluoren-9-yl)methyl thioultraphosphate (6.0 mM in water/MeCN, 10 mM TEAA; 270 µl, 1.62 µmol, 1.0 eq.) and stirred for 30-40 min. The product (purity according to ^{31}P{^{1}H}-NMR: 90%) could not be isolated.

^{31}P-NMR (162 MHz, D_2O): δ = –5.71 (d, *J* = 26.3 Hz, 3 P), 22.44 ppm (q, *J* = 26.8 Hz, 1 P). **HRMS (ESI)**: *m/z* calcd for $H_5O_{12}P_4S$ $[M - H^+]^-$: 352.8458, found: 352.8458.

Bisphenyladenosine ultraphosphate (43)

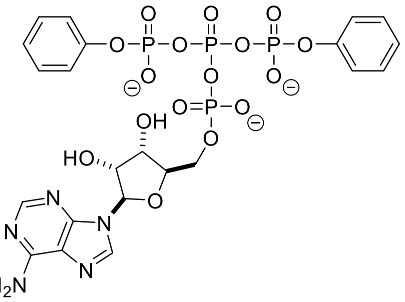

Chemical Formula: $C_{22}H_{22}N_5O_{16}P_4^{3-}$
Exact Mass: 736.0028

Phenyl phosphate · 1.0 TBA (219 mg, 529 µmol, 2.0 eq.) was coevaporated using MeCN (3 x 4 ml) and then dissolved in DMF (4 ml). DIPEA (135 µl, 103 mg, 793 µmol, 3.0 eq.) was added and the mixture cooled to 0°C. (*i*Pr)$_2$N-PCl$_2$ (48 µl, 53 mg, 264 µmol, 1.0 eq.) was slowly added and stirred for 15 min. In another flask, AMP · 1.0 TBA (155 mg, 264 µmol, 1.0 eq.) and DCI (125 mg, 1.06 mmol, 4.0 eq.) were coevaporated using MeCN (3 x 2 ml), dissolved in DMF (2.5 ml) and added to the reaction mixture at 0°C. It was stirred 15 min at room temp. and then cooled to 0°C again. *m*CPBA (≤77 %, 89 mg, 396 µmol, 1.5 eq.) was added and stirred for 10 min. The product was precipitated using ice-cooled Et$_2$O (100 ml), the suspension centrifuged and the pellet washed with ice-cooled Et$_2$O (100 ml). Drying *in vacuo* gave a white solid (m_{crude} = 238 mg), which was purified by AIEX chromatography (Q Sepharose® Fast Flow, increasing concentrations of NaClO$_4$, eluting at 0.15 M buffer concentration). The purified product had a purity of 93% according to $^{31}P\{^1H\}$-NMR.

^{1}H-NMR (400 MHz, D$_2$O, presat): δ = 4.06-4.16 (m, 2 H), 4.21 (m, 1 H), 4.31 (m, 1 H), 4.51 (m, 1 H), 5.93 (d, *J* = 5.5 Hz, 1 H), 6.93-7.19 (m, 10 H, phenyl-H), 8.11 (s, 1 H), 8.25 ppm (s, 1 H). **^{31}P{^{1}H}-NMR** (162 MHz, D$_2$O): δ = −37.02 (q, *J* = 18.9 Hz, 1 P), −17.04 (d, *J* = 19.1 Hz, 1 P_{Ph}), −17.01 (d, *J* = 19.1 Hz, 1 P_{Ph}), −12.27 ppm (d, *J* = 18.4 Hz, 1 P_A). **^{31}P-NMR** (162 MHz, D$_2$O): δ = −37.02 (q, *J* = 18.9 Hz, 1 P), −17.04 (d, *J* = 19.0 Hz, 1 P_{Ph}), −17.01 (d, *J* = 18.9 Hz, 1 P_{Ph}), −12.27 ppm (m, 1 P_A). **HRMS (ESI)**: *m/z* calcd for $C_{22}H_{26}N_5O_{16}P_4$ [M + H$^+$]$^+$: 740.0320, found: 740.0320.

Bisphenyl-(9*H*-fluoren-9-yl)methyl ultraphosphate (44)

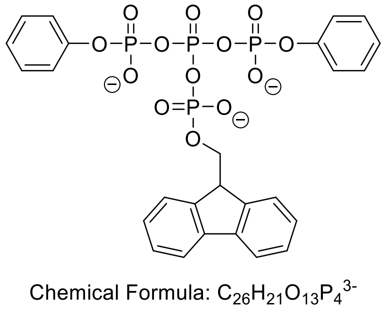

Chemical Formula: $C_{26}H_{21}O_{13}P_4^{3-}$
Exact Mass: 664.9949

Phenyl phosphate · 1.0 TBA (220 mg, 531 µmol, 2.0 eq.) was coevaporated using MeCN (3 x 3 ml) and then dissolved in DMF (3 ml). DIPEA (99 µl, 175 mg, 584 µmol, 2.2 eq.) was added and the mixture cooled to 0°C. (*i*Pr)$_2$N-PCl$_2$ (49 µl, 54 mg, 265 µmol, 1.0 eq.) was slowly added and stirred for 15 min. (9*H*-Fluoren-9-yl)methyl dihydrogen phosphate (73 mg, 265 µmol, 1.0 eq.) and DCI (125 mg, 1.06 mmol, 4.0 eq.) were

coevaporated using MeCN (3 x 2 ml), dissolved in DMF (2.5 ml) and added to the reaction mixture at 0°C. It was stirred 15 min at room temp. and then cooled to 0°C again. *m*CPBA (≤77 %, 89 mg, 398 µmol, 1.5 eq.) was added and stirred for 10 min. The product was precipitated using ice-cooled Et_2O (100 ml), the suspension centrifuged and the pellet washed with ice-cooled Et_2O (100 ml). Drying *in vacuo* gave a white solid (m_{crude} = 236 mg), which was purified using a PuriFlash Column (30µ C18 AQ; water, MeCN gradient (0-45%), 10% TEAA (100 mM, pH 7.0)).

^{1}H-NMR (400 MHz, D_2O, presat): δ = 4.27 (dd, 2 H, *J* = 7.1, 7.1 Hz, CH_2), 4.37 (t, 1 H, *J* = 7.3 Hz, CH), 7.18-7.24 (m, 2 H, 2 x Ph-H), 7.29-7.37 (m, 8 H, 8 x Ph-H), 7.53 (m, 2 H), 7.65 (m, 2 H), 7.84 (m, 2 H), 8.06 ppm (m, 2 H). **^{31}P{^{1}H}-NMR** (162 MHz, D_2O): δ = –36.99 (q, *J* = 18.8 Hz, 1 P), –17.50 (d, *J* = 18.9 Hz, 2 P_{Ph}), –12.17 ppm (d, *J* = 19.0 Hz, 1 P_{Fm}). **^{31}P-NMR** (162 MHz, D_2O): δ = –36.99 (q, *J* = 19.0 Hz, 1 P), –17.50 (d, *J* = 18.9 Hz, 2 P_{Ph}), –12.17 ppm (dt, *J* = 18.6 Hz, *J* = 6.3 Hz, 1 P_{Fm}). **HRMS (ESI)**: *m/z* calcd for $C_{26}H_{23}O_{13}P_4$ [M – H$^+$]$^-$: 667.0095, found: 667.0100.

Bisphenyl ultraphosphate (53)

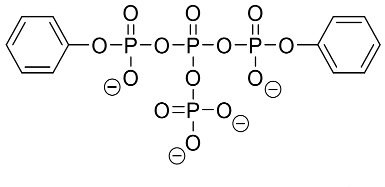

Equal volumes of a solution of bisphenyl-(9*H*-fluoren-9-yl)methyl ultraphosphate (water/MeCN, 10 mM TEAA) and a 10% solution of DBU in water were mixed. The reaction is finished within 15 min. The product (purity according to ^{31}P{^{1}H}-NMR: 71%) could not be isolated.

^{31}P-NMR (162 MHz, D_2O): δ = –36.54 (dt, *J* = 22.2 Hz, *J* = 19.5 Hz, 1 P), –17.17 (d, *J* = 19.2 Hz, 2 P_{Ph}), –4.01 ppm (d, *J* = 22.1 Hz, 1 P).

Bisadenosine(9*H*-fluoren-9-yl)methyl ultraphosphate (45)

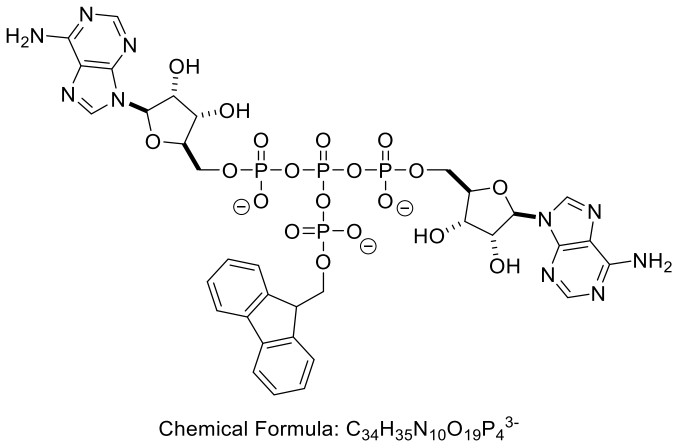

Bisadenosine(9*H*-fluoren-9-yl)methyl ultraphosphate was isolated as a byproduct in the synthesis of bis((9*H*-fluoren-9-yl)methyl)adenosine ultraphosphate. The ultraphosphate was converted into its [PPN] salt according to the general procedure.

^{1}H-NMR (400 MHz, CD_3CN): δ = 3.98 (dt, *J* = 11.5 Hz, *J* = 3.5 Hz, 1 H, Fm-CH), 4.05 (m, 3 H), 4.24 (m 2 H, Fm-CH_2), 4.29 (m, 1 H), 4.37 (m, 2 H), 4.72-4.85 (m, 4 H, 2 x 2'-H + 2 x 3'-H), 5.98 (d, *J* = 4.1 Hz, 1 H, 1'-H), 5.99 (d, *J* = 3.8 Hz, 1 H, 1'-H), 6.62 (br. s, 4 H, 2 x NH_2), 7.21-7.33 (m, 4 H), 7.74 (m, 2 H), 7.85 (m, 2 H), 7.93 (s, 1 H, 1-H), 7.94 (s, 1 H, 1-H), 8.54 (s, 1 H, 2-H), 8.57 ppm (s, 1 H, 2-H). **^{31}P{^{1}H}-NMR** (162 MHz, CD_3CN): δ = −35.69 (q, *J* = 23.0 Hz, 1 P), −13.19 (d, *J* = 23.9 Hz, 1 P_{Fm}), −12.86 (d, *J* = 18.3 Hz, 1 P_A), −12.73 ppm (d, *J* = 18.1 Hz, 1 P_A). **^{31}P-NMR** (162 MHz, CD_3CN): δ = −35.69 (q, *J* = 22.7 Hz, 1 P), −13.19 (br. d, *J* = 23.1 Hz, 1 P_{Fm}), −12.97 to −12.65 ppm (m, 2 P_A). **^{13}C-NMR** (101 MHz, CD_3CN): δ = 49.5 (d, *J* = 7.5 Hz), 65.6 (m), 68.5 (d, *J* = 6.4 Hz), 70.8, 71.3, 76.3, 76.6, 84.6 (d, *J* = 8.2 Hz), 85.1 (d, *J* = 9.5 Hz), 87.9, 88.2, 119.6, 120.4, 127.0, 127.1, 127.9, 128.1, 140.4, 140.5, 142.0, 146.3, 150.4, 150.6, 153.4, 156.6 ppm. **HRMS (ESI)**: *m/z* calcd for $C_{34}H_{37}N_{10}O_{19}P_4$ $[M - H^+]^-$: 1013.1192, found: 1013.1203. **Raman:** $\tilde{\nu}$ =3174 (vw), 3147 (vw), 3062 (s), 3012 (vw), 2994 (vw), 2960 (vw), 2939 (vw), 2919 (vw), 2888 (vw), 1610 (w), 1589 (s), 1577 (w), 1508 (vw), 1483 (vw), 1440 (vw), 1421 (vw), 1371 (vw), 1344 (vw), 1297 (vw), 1238 (vw), 1224 (vw), 1186 (vw), 1164 (vw), 1110 (w), 1074 (vw), 1029 (m), 1000 (vs), 792 (vw), 744 (vw), 727 (vw), 663 (w), 617 (w), 530 (vw), 416 (vw), 364 (vw), 356 (vw), 322 (vw), 302 (vw), 266 (vw), 252 (vw), 237 cm^{-1} (w).

Bisadenosine ultraphosphate (54)

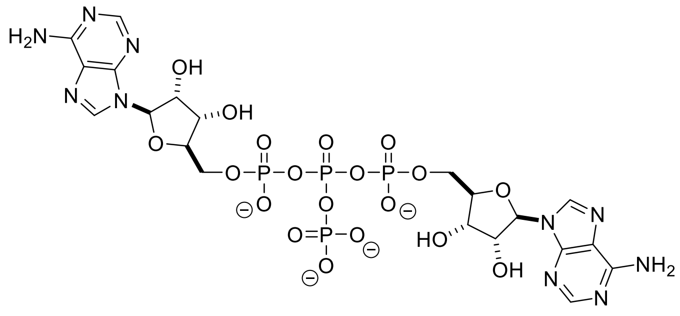

Chemical Formula: $C_{20}H_{24}N_{10}O_{19}P_4^{4-}$
Exact Mass: 832.0192

Equal volumes of a solution of bisadenosine(9*H*-fluoren-9-yl)methyl ultraphosphate (water/MeCN, 10 mM TEAA) and a 10% solution of DBU in water were mixed. The reaction is finished within 15 min. The product (purity according to ^{31}P{^{1}H}-NMR: 66%) could not be isolated.

^{31}P{^{1}H}-NMR (162 MHz, D_2O): δ = −36.11 (q, *J* = 22.5 Hz, 1 P), −12.23 (d, *J* = 19.7 Hz, 1 P_A), −12.16 (d, *J* = 19.4 Hz, 1 P_A), −4.58 ppm (d, *J* = 22.6 Hz, 1 P). **^{31}P-NMR** (162 MHz, D_2O): δ = −36.11 (q, *J* = 22.4 Hz, 1 P), −12.32 to −12.04 (m, 2 P_A), −4.59 ppm (d, *J* = 22.6 Hz, 1 P). **HRMS (ESI)**: *m/z* calcd for $C_{20}H_{27}O_{19}N_{10}P_4$ $[M - H^+]^-$: 835.0410, found: 835.0413.

Bis((9*H*-fluoren-9-yl)methyl)phenyl ultraphosphate (46)

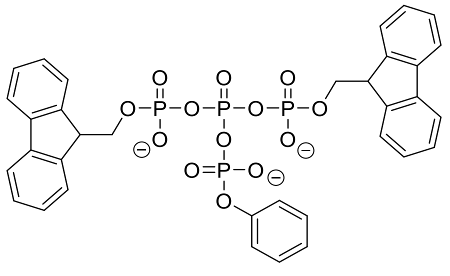

Chemical Formula: $C_{34}H_{27}O_{13}P_4^{3-}$
Exact Mass: 767.0419

(9*H*-Fluoren-9-yl)methyl dihydrogen phosphate (306 mg, 1.11 mmol, 2.0 eq.) and DCI (524 mg, 4.44 mmol, 8.0 eq.) were coevaporated using MeCN (3 x 5 ml) and dissolved in DMF (5 ml). In another flask, phenyl phosphate · 1.0 TBA (230 mg, 555 µmol, 1.0 eq.) was coevaporated using MeCN (3 x 3 ml) and then dissolved in DMF (3.5 ml). DIPEA (104 µl, 79 mg, 611 µmol, 1.1 eq.) was added and the mixture cooled to 0°C. $((i\mathrm{Pr})_2\mathrm{N})_2$-PCl (148 mg, 555 µmol, 1.0 eq.) was added and stirred for 15 min. This mixture was added to the solution of (9*H*-fluoren-9-yl)methyl dihydrogen phosphate and DCI in DMF at 0°C. It was stirred 15 min at room temp. and then cooled to 0°C again. *m*CPBA (≤77 %, 187 mg, 832 µmol, 1.5 eq.) was added and stirred for 10 min. The product was precipitated using ice-cooled Et_2O (200 ml), the suspension centrifuged and the pellet washed with ice-cooled Et_2O (200 ml). Drying *in vacuo* gave a white solid (m_{crude} = 453 mg), which was purified using a PuriFlash Column (30µ C18 AQ; water, MeCN gradient (0-45%), 10% TEAA (100 mM, pH 7.0)).

^{1}H-NMR (400 MHz, MeCN, presat): δ = 4.08 (m, 6 H, 2 x CH + 4 x CH_2), 6.86 (m, 2 H, 2 x Ph-H), 7.00-7.09 (m, 3 H, 3 x Ph-H), 7.19 (m, 4 H), 7.31 (m, 4 H), 7.57 (m, 4 H), 7.73 ppm (m, 4 H). **^{31}P{^{1}H}-NMR** (162 MHz, MeCN): δ = –36.28 (q, *J* = 18.4 Hz, 1 P), –17.99 (d, *J* = 18.4 Hz, 1 P_{Ph}), –12.37 ppm (d, *J* = 18.2 Hz, 2 P_{Fm}). **^{31}P-NMR** (162 MHz, MeCN): δ = –36.27 (q, *J* = 18.3 Hz, 1 P), –17.99 (d, *J* = 18.5 Hz, 1 P_{Ph}), –12.37 ppm (m, 2 P_{Fm}). **HRMS (ESI)**: *m/z* calcd for $C_{34}H_{29}O_{13}P_4$ [M – H^+]$^-$: 769.0564, found: 769.0566.

Phenyl ultraphosphate (50)

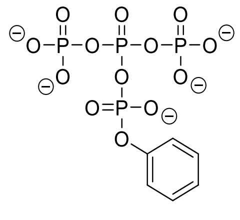

Chemical Formula: $C_6H_5O_{13}P_4^{5-}$
Exact Mass: 408.8708

Equal volumes of a solution of bis((9*H*-fluoren-9-yl)methyl)phenyl ultraphosphate (water/MeCN, 10 mM TEAA) and a 10% solution of DBU in water were mixed. The reaction is finished within 15 min. The product (purity according to ^{31}P{^{1}H}-NMR: 77%) could not be isolated.

^{31}P-NMR (162 MHz, MeCN): δ = –35.90 (q, *J* = 20.3 Hz, 1 P), –16.70 (d, *J* = 18.4 Hz, 1 P_{Ph}), –4.83 ppm (d, *J* = 22.5 Hz, 2 P). **HRMS (ESI)**: *m/z* calcd for $C_6H_9O_{13}P_4$ [M – H^+]$^-$: 412.8999, found: 412.8994.

Bis((9*H*-fluoren-9-yl)methyl)benzyl ultraphosphate (47)

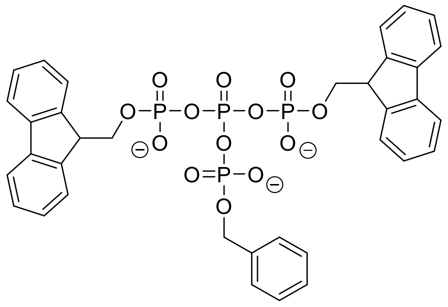

Chemical Formula: $C_{35}H_{29}O_{13}P_4^{3-}$
Exact Mass: 781.0575

(9*H*-Fluoren-9-yl)methyl dihydrogen phosphate (301 mg, 1.09 mmol, 2.0 eq.) and DCI (516 mg, 4.37 mmol, 8.0 eq.) were coevaporated using MeCN (3 x 4 ml) and dissolved in DMF (5 ml). In another flask, benzyl phosphate · 1.2 TBA (263 mg, 546 µmol, 1.0 eq.) was coevaporated using MeCN (3 x 3 ml) and then dissolved in DMF (3.5 ml). DIPEA (102 µl, 78 mg, 601 µmol, 1.1 eq.) was added and the mixture cooled to 0°C. $((iPr)_2N)_2$-PCl (145 mg, 546 µmol, 1.0 eq.) was added and stirred for 15 min. This mixture was added to the solution of (9*H*-fluoren-9-yl)methyl dihydrogen phosphate and DCI in DMF at 0°C. It was stirred 15 min at room temp. and then cooled to 0°C again. *m*CPBA (≤77 %, 184 mg, 820 µmol, 1.5 eq.) was added and stirred for 10 min. The product was precipitated using ice-cooled Et_2O (200 ml), the suspension centrifuged and the pellet washed with ice-cooled Et_2O (200 ml). Drying *in vacuo* gave a white solid (m_{crude} = 399 mg), which was purified using a PuriFlash Column (30µ C18 AQ; water, MeCN gradient (0-45%), 10% TEAA (100 mM, pH 7.0)).

^{1}H-NMR (400 MHz, D_2O, presat): δ = 4.20 (m, 6 H, 2 x CH + 2 x CH_2), 4.91 (d, *J* = 6.6 Hz, 2 H, benzyl-CH_2), 7.24 (m, 5 H, 5 x benzyl-H), 7.31 (m, 4 H), 7.46 (m, 4 H), 7.69 (m, 4 H), 7.85 ppm (m, 4 H). **^{31}P{^{1}H}-NMR** (162 MHz, D_2O): δ = −36.15 (q, *J* = 19.2 Hz, 1 P), −11.98 ppm (d, *J* = 18.7 Hz, 3 P). **^{31}P-NMR** (162 MHz, D_2O): δ = −36.15 (q, *J* = 18.7 Hz, 1 P), −11.97 ppm (dt, *J* = 17.8 Hz, 5.6 Hz, 3 P). **HRMS (ESI)**: *m/z* calcd for $C_{35}H_{33}O_{13}P_4$ [M + H$^+$]$^+$: 785.0866, found: 785.0861.

Benzyl ultraphosphate (51)

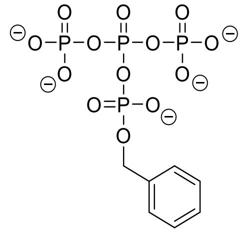

Chemical Formula: $C_7H_7O_{13}P_4^{5-}$
Exact Mass: 422.8865

Equal volumes of a solution of bis((9*H*-fluoren-9-yl)methyl)benzyl ultraphosphate (water/MeCN, 10 mM TEAA) and a 10% solution of DBU in water were mixed. The reaction is finished within 15 min. The product (purity according to ^{31}P{^{1}H}-NMR: 69%) could not be isolated.

^{31}P{^{1}H}-NMR (162 MHz, D_2O): δ = −35.80 (td, *J* = 22.9, *J* = 19.2 Hz 1 P), −11.69 (d, *J* = 19.2 Hz, 1 P_{Bn}), −4.93 ppm (d, *J* = 22.3 Hz, 2 P).

Bis((9*H*-fluoren-9-yl)methyl)adenosine ultraphosphate (48)

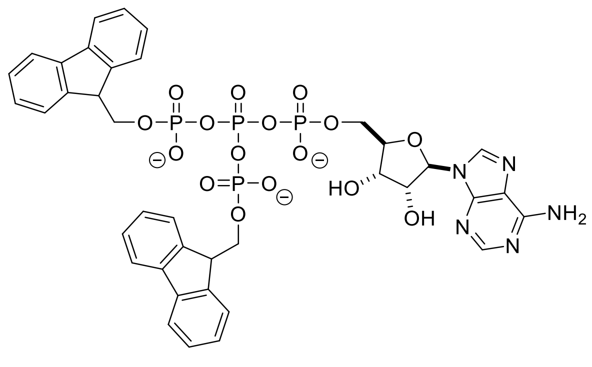

(9*H*-Fluoren-9-yl)methyl dihydrogen phosphate (390 mg, 1.41 mmol, 2.0 eq.) and DCI (668 mg, 5.66 mmol, 8.0 eq.) were coevaporated using MeCN (3 x 5 ml) and dissolved in DMF (10 ml). In another flask, AMP · 1.1 TBA (600 mg, 0.99 mmol, 1.4 eq.) was coevaporated using MeCN (3 x 5 ml), dissolved in DMF (8 ml) and cooled to 0°C. DIPEA (132 µl, 101 mg, 0.78 mmol, 1.1 eq.) and $((iPr)_2N)_2$-PCl (188 mg, 0.71 mmol, 1.0 eq.) were added, shortly ultrasonified and stirred for 15 min. This mixture was added to the solution of (9*H*-fluoren-9-yl)methyl dihydrogen phosphate and DCI in DMF at 0°C, warmed to room temp. and stirred for 15 min. *m*CPBA (≤77 %, 238 mg, 1.06 µmol, 1.5 eq.) was added and stirred for 10 min. The product was precipitated using ice-cooled Et_2O (300 ml), the suspension centrifuged and the pellet washed with ice-cooled Et_2O (300 ml). Drying *in vacuo* gave a white solid (m_{crude} = 766 mg), which was purified using a PuriFlash Column (30µ C18 AQ; water, MeCN gradient (0-45%), 10% TEAA (100 mM, pH 7.0)).

^{1}H-NMR (400 MHz, D_2O, presat): δ = 4.16-4.28 (m, 9 H, 2 x Fm-CH + 2 x Fm-CH_2 + 4'-H + 5'-H_2), further sugar signals not resolved due to presat, 5.97 (d, *J* = 5.4 Hz, 1 H), 7.24-7.83 (m, 16 H), 8.22 (s, 1 H), 8.39 ppm (s, 1 H). **^{31}P{^{1}H}-NMR** (162 MHz, D_2O): δ = –36.34 (q, *J* = 20.0 Hz, 1 P), –12.08 ppm (m, 3 P). **^{31}P-NMR** (162 MHz, D_2O): δ = –36.34 (q, *J* = 19.9 Hz, 1 P), –12.09 ppm (m, 3 P). **HRMS (ESI)**: *m/z* calcd for $C_{38}H_{37}N_5O_{16}P_4$ $[M + H^+]^+$: 944.1259, found: 944.1259.

Adenosine ultraphosphate (52)

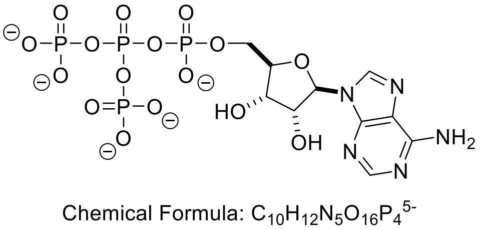

Equal volumes of a solution of bis((9*H*-fluoren-9-yl)methyl)adenosine ultraphosphate (water/MeCN, 10 mM TEAA) and a 10% solution of DBU in water were mixed. The reaction is finished within 15 min. The product (purity according to ^{31}P{^{1}H}-NMR: 54%) could not be isolated.

^{31}P{^{1}H}-NMR (162 MHz, D_2O): δ = –35.80 (td, *J* = 22.2, *J* = 19.3 Hz, 1 P), –12.02 (d, *J* = 19.9 Hz, 1 P_A), –5.16 ppm (d, *J* = 22.8 Hz, 2 P). **^{31}P-NMR** (162 MHz, D_2O): δ = –35.81 (td, *J* = 22.6, *J* = 19.4 Hz, 1 P), –12.02 (m, 1 P_A), –5.17 ppm (d, *J* = 22.6 Hz, 2 P). **HRMS (ESI)**: *m/z* calcd for $C_{10}H_{17}O_{16}N_5P_4$ $[M - H^+]^-$: 585.9548, found: 585.9551.

Bis((9*H*-fluoren-9-yl)methyl)pentynyle ultraphosphate (49)

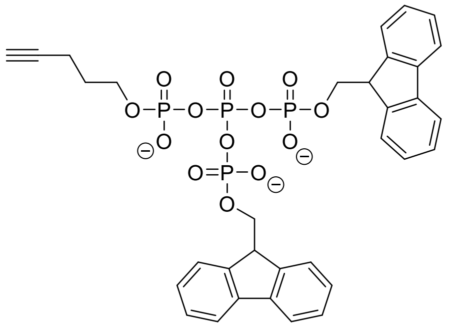

Chemical Formula: $C_{33}H_{29}O_{13}P_4^{3-}$
Exact Mass: 757.0575

(9*H*-Fluoren-9-yl)methyl dihydrogen phosphate (175 mg, 633 µmol, 2.0 eq.) and DCI (299 mg, 2.53 mmol, 8.0 eq.) were coevaporated using MeCN (3 x 3 ml) and dissolved in DMF (2.5 ml). In another flask, penynyle phosphate · 1.2 TBA (142 mg, 317 µmol, 1.0 eq.) was coevaporated using MeCN (3 x 2 ml), dissolved in DMF (2.5 ml) and cooled to 0°C. DIPEA (59 µl, 45 mg, 348 µmol, 1.1 eq.) and ((*i*Pr)$_2$N)$_2$-PCl (84 mg, 317 µmol, 1.0 eq.) were added and stirred for 15 min. This mixture was added to the solution of (9*H*-fluoren-9-yl)methyl dihydrogen phosphate and DCI in DMF at 0°C. It was stirred 15 min at room temp. and then cooled to 0°C again. *m*CPBA (≤77 %, 106 mg, 475 µmol, 1.5 eq.) was added and stirred for 10 min. The product was precipitated using ice-cooled Et_2O (200 ml), the suspension centrifuged and the pellet washed with ice-cooled Et_2O (200 ml). Drying *in vacuo* gave a white solid (m_{crude} = 273 mg), which was purified using a PuriFlash Column (30µ C18 AQ; water, MeCN gradient (0-45%), 10% TEAA (100 mM, pH 7.0)).

^{1}H-NMR (400 MHz, D_2O, presat, 2 H of pentynyl residue not resolved due to solvent signal): δ = 1.82 (ddt, *J* = 3 x 6.3 Hz, 2 H, pentynyl-CH$_2$), 4.04 (m, 2 H, pentynyl-CH$_2$), 4.17-4.24 (m, 6 H, 2 x Fm-CH + 2 x Fm-CH$_2$), 7.36 (m, 4 H), 7.47 (m, 4 H), 7.71 (m, 4 H), 7.86 ppm (d, *J* = 7.6 Hz, 4 H). **^{31}P{^{1}H}-NMR** (162 MHz, D_2O): δ = −36.34 (q, *J* = 19.2 Hz, 1 P), −12.05 (d, *J* = 18.5 Hz, 2 P_{Fm}), −11.68 ppm (d, *J* = 18.6 Hz, 1 P_{Pent}). **^{31}P-NMR** (162 MHz, D_2O): δ = −36.34 (q, *J* = 19.2 Hz, 1 P), −12.05 (m, 2 P_{Fm}), −11.68 ppm (m, 1 P_{Pent}). **HRMS (ESI)**: *m/z* calcd for $C_{33}H_{31}O_{13}P_4$ [M − H$^+$]$^-$: 759.0721, found: 759.0724.

Bis((9*H*-fluoren-9-yl)methyl)-3-(1-(PEG$_3$-5-FAM)-1*H*-1,2,3-triazol-4-yl)propyl ultraphosphate (55)

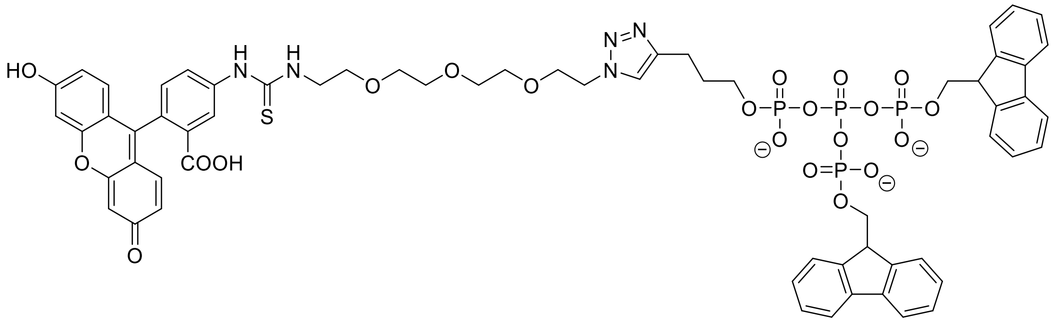

Chemical Formula: $C_{62}H_{58}N_5O_{21}P_4S^{3-}$
Exact Mass: 1364.2312

A solution of TEAA (1 M, pH 7.0, 200 µl) was added to a solution of bis((9*H*-fluoren-9-yl)methyl)pentynyle ultraphosphate (3.1 mM in water/MeCN, 10 mM TEAA; 1.00 ml, 3.12 µmol, 1.0 eq.) to yield approximately a 100 mM solution of TEEA in the final reaction mixture. The mixture was degased by bubbling a stream of argon through the solution for 10 min. Sodium ascorbate (10 mg/ml in water, 309 µl, 3.09 mg, 15.6 µmol, 5.0 eq.), $CuSO_4 \cdot 5\ H_2O$ (10 mg/ml in water, 78 µl, 0.78 mg, 3.12 µmol, 1.0 eq.) and 5-FAM-PEG$_3$-azide (10 mg/ml in water/MeCN 1:1; 189 µl, 1.89 mg, 3.12 µmol, 1.0 eq.) were added. MeCN (0.8 ml) was added to increase the solubility and the mixture stirred for 2 h.

HRMS (ESI): *m/z* calcd for $C_{62}H_{62}O_{21}N_5P_4S$ [M + H$^+$]$^+$: 1368.2603, found: 1368.2594.

Bis((9*H*-fluoren-9-yl)methyl)-3-(1-(PEG$_3$-biotin)-1*H*-1,2,3-triazol-4-yl)propyl ultraphosphate (56)

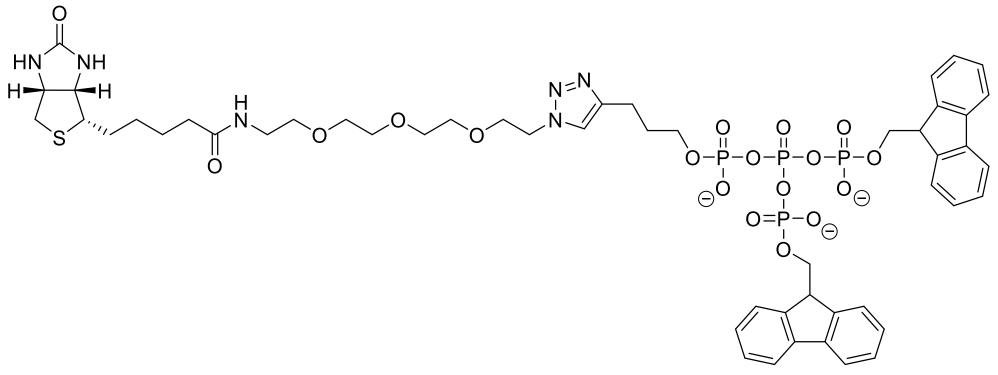

Chemical Formula: $C_{51}H_{61}N_6O_{18}P_4S^{3-}$
Exact Mass: 1201.2730

A solution of TEAA (1 M, pH 7.0, 165 µl) was added to a solution of bis((9*H*-fluoren-9-yl)methyl)pentynyle ultraphosphate (0.65 mM in water/MeCN, 10 mM TEAA; 1.50 ml, 0.97 µmol, 1.0 eq.) to yield approximately a 100 mM solution of TEEA in the final reaction mixture. The mixture was degased by bubbling a stream of argon through the solution for 10 min. Sodium ascorbate (10 mg/ml in water, 96 µl, 0.96 mg, 4.86 µmol, 5.0 eq.), $CuSO_4 \cdot 5\ H_2O$ (10 mg/ml in water, 24 µl, 0.24 mg, 0.97 µmol, 1.0 eq.) and biotin-PEG$_3$-azide (10 mg/ml in water/MeCN; 1:1, 43 µl, 0.43 mg, 0.97 µmol, 1.0 eq.) were added. The mixture was stirred for 2.5 h.

HRMS (ESI): *m/z* calcd for $C_{51}H_{63}O_{18}N_6P_4S$ [M + H$^+$]$^+$: 1203.2875, found: 1203.2872.

X-ray crystallography data

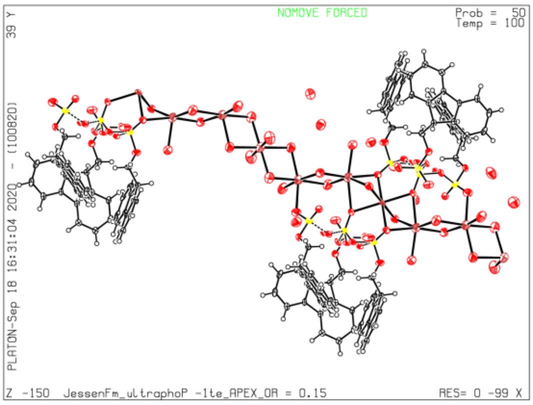

The compound was crystallized from water at 25 °C. The data for JessenFm_ultraphosphate_APEX_0m were collected from a shock-cooled single crystal at 100(2) K on a Bruker APEX2 QUAZAR three-circle diffractometer with a microfocus sealed X-ray tube using mirror optics as monochromator and a Bruker APEXII detector. The diffractometer was equipped with an Oxford Cryostream 800 low temperature device and used MoK_α radiation (λ = 0.71073 Å). All data were integrated with SAINT and a multi-scan absorption correction using SADABS was applied.[22,23] The structure were solved by direct methods using SHELXT and refined by full-matrix least-squares methods against F^2 by SHELXL-2018/3.[24,25] All non-hydrogen atoms were refined with anisotropic displacement parameters. The hydrogen atoms were refined isotropically on calculated positions using a riding model with their U_{iso} values constrained to 1.5 times the U_{eq} of their pivot atoms for terminal sp^3 carbon atoms and 1.2 times for all other carbon atoms. Disordered moieties were refined using bond lengths restraints and displacement parameter restraints. Crystallographic data (including structure factors) for the structures reported in this paper have been deposited with the Cambridge Crystallographic Data Centre.[26] CCDC 2032762 contain the supplementary crystallographic data for this paper. Copies of the data can be obtained free of charge from the Cambridge Crystallographic Data Centre via www.ccdc.cam.ac.uk/structures. This report and the CIF file were generated using FinalCif.[27]

Supplementary Table 1: Crystal data and structure refinement for JessenFm_ultraphosphate_APEX_0m

CCDC number	2032762
Empirical formula	$C_{42}H_{33}Na_3O_{28}P_4$
Formula weight	1178.53
Temperature [K]	100(2)
Crystal system	triclinic
Space group (number)	$P\bar{1}$ (2)
a [Å]	10.684
b [Å]	12.958
c [Å]	21.768
α [Å]	96.93
β [Å]	100.79
γ [Å]	109.43
Volume [Å^3]	2737.0
Z	2
ρ_{calc} [g/cm^3]	1.430
μ [mm^{-1}]	0.249
F(000)	1204
Crystal size [mm^3]	0.190×0.150×0.140
Crystal colour	colourless
Crystal shape	block
Radiation	MoK_α (λ=0.71073 Å)
2Θ range [°]	3.40 to 53.37 (0.79 Å)
Index ranges	$-13 \le h \le 13$<br>$-16 \le k \le 16$<br>$-27 \le l \le 27$
Reflections collected	45668
Independent reflections	11438<br>R_{int} = 0.0939<br>R_{sigma} = 0.0893
Completeness to θ = 25.242°	99.9 %
Data / Restraints / Parameters	11438/1059/725
Goodness-of-fit on F^2	1.158
Final R indexes [$I \geq 2\sigma(I)$]	R_1 = 0.1463<br>wR_2 = 0.3802
Final R indexes [all data]	R_1 = 0.1868<br>wR_2 = 0.4011
Largest peak/hole [eÅ^3]	1.43/-1.39

Supplementary Table 2: Atomic coordinates and U_{eq} [Å^2] for JessenFm_ultraphosphate_APEX_0m

Atom	*x*	*y*	*z*	U_{eq}
P1	0.5204(2)	0.65724(19)	0.64101(11)	0.0198(5)
P2	0.7803(2)	0.8218(2)	0.64292(11)	0.0235(5)
P3	0.4804(3)	0.4248(2)	0.64588(11)	0.0240(5)
P4	0.2986(3)	0.7288(2)	0.66683(12)	0.0253(6)
Na1	0.500000	0.500000	0.500000	0.0236(10)
Na2	0.6719(5)	0.3565(4)	0.5310(2)	0.0428(11)
Na3	0.7337(5)	0.1166(4)	0.4557(2)	0.0437(11)
Na4	1.000000	0.000000	0.500000	0.0452(15)
O1	0.2757(7)	0.7258(5)	0.7364(3)	0.0230(13)
O2	0.1991(7)	0.6289(6)	0.6214(3)	0.0282(14)
O5	0.4774(6)	0.6378(5)	0.5722(3)	0.0219(12)
O7	0.5234(9)	0.4134(7)	0.5867(3)	0.0384(17)
O8	0.7401(7)	0.5570(6)	0.5226(3)	0.0350(15)
O9	0.7995(9)	0.3870(7)	0.6380(4)	0.0469(19)
O10	0.8551(8)	0.3158(7)	0.4986(4)	0.0403(16)
O11	0.5457(9)	0.1635(7)	0.4898(4)	0.0490(19)
O12	0.5749(9)	-0.0692(7)	0.4185(5)	0.054(2)
O13	0.8402(9)	0.0617(7)	0.5484(4)	0.0486(19)
O14	0.9122(9)	0.0731(7)	0.4149(4)	0.0500(19)
O15	1.1947(11)	0.1736(8)	0.5307(5)	0.067(3)
O16	0.8586(7)	0.7635(6)	0.6146(3)	0.0354(17)
O17	0.7056(8)	0.8771(6)	0.6041(4)	0.0411(18)
O18	0.8707(7)	0.9086(6)	0.7055(3)	0.0330(16)
O19	0.3394(9)	0.3594(8)	0.6468(4)	0.048(2)
O20	0.2674(11)	1.1029(9)	0.6812(5)	0.068(3)
O21	0.4608(11)	1.0308(9)	0.7389(5)	0.067(3)
O22	0.1286(8)	0.4096(6)	0.5774(4)	0.0417(19)
O23	0.3446(9)	-0.0658(8)	0.3374(4)	0.051(2)
O24	0.9527(8)	0.6029(8)	0.6587(4)	0.046(2)
O25	0.9050(9)	0.2225(7)	0.6589(5)	0.053(2)
O26	1.1782(9)	0.2772(7)	0.7313(4)	0.049(2)
O27	0.5888(7)	0.4084(6)	0.7007(3)	0.0322(15)
O28	0.3138(9)	0.8382(7)	0.6531(4)	0.0435(19)
C1	0.2485(9)	0.6219(7)	0.7590(4)	0.0204(17)
H1A	0.291137	0.575266	0.737743	0.024
H1B	0.148652	0.579805	0.749186	0.024
C2	0.3081(8)	0.6498(7)	0.8303(4)	0.0153(14)
H2	0.267080	0.698887	0.851515	0.018
C3	0.4620(8)	0.7034(7)	0.8491(4)	0.0136(14)
C4	0.5473(9)	0.8015(7)	0.8381(4)	0.0182(16)
H4	0.509974	0.847213	0.815683	0.022
C5	0.6882(9)	0.8337(7)	0.8597(4)	0.0210(17)
H5	0.747216	0.900980	0.851534	0.025
C6	0.7423(9)	0.7681(7)	0.8929(4)	0.0201(16)
H6	0.838748	0.790421	0.907320	0.024
C7	0.6586(9)	0.6707(7)	0.9056(4)	0.0209(17)
H7	0.696481	0.626622	0.929258	0.025
C8	0.5172(9)	0.6376(7)	0.8831(4)	0.0146(14)
C9	0.4075(9)	0.5397(7)	0.8875(4)	0.0162(15)
C10	0.4082(9)	0.4484(7)	0.9168(4)	0.0220(17)
H10	0.492017	0.443989	0.937792	0.026
C11	0.2855(10)	0.3660(7)	0.9144(5)	0.0267(19)

H11	0.284811	0.304505	0.934225	0.032
C12	0.1629(9)	0.3715(7)	0.8834(5)	0.0255(19)
H12	0.079475	0.313208	0.882111	0.031
C13	0.1595(9)	0.4611(7)	0.8541(5)	0.0225(18)
H13	0.075309	0.464489	0.832669	0.027
C14	0.2834(9)	0.5451(7)	0.8574(4)	0.0155(14)
C15	0.9617(9)	0.8851(7)	0.7532(4)	0.0232(18)
H15A	1.014837	0.847025	0.733303	0.028
H15B	0.909899	0.835862	0.778438	0.028
C16	1.0580(8)	0.9969(7)	0.7963(4)	0.0160(15)
H16	1.110604	1.046316	0.770667	0.019
C17	1.1553(8)	0.9820(7)	0.8514(4)	0.0152(14)
C18	1.2527(8)	0.9338(7)	0.8499(4)	0.0180(16)
H18	1.267288	0.906448	0.810401	0.022
C19	1.3287(9)	0.9263(7)	0.9073(4)	0.0195(16)
H19	1.394520	0.891831	0.906924	0.023
C20	1.3095(8)	0.9688(6)	0.9656(4)	0.0176(15)
H20	1.362223	0.963315	1.004487	0.021
C21	1.2145(8)	1.0185(7)	0.9666(4)	0.0166(15)
H21	1.201226	1.047491	1.006065	0.020
C22	1.1387(8)	1.0257(6)	0.9098(4)	0.0130(14)
C23	1.0301(8)	1.0717(6)	0.8971(4)	0.0131(14)
C24	0.9722(8)	1.1199(7)	0.9378(4)	0.0180(16)
H24	1.004446	1.130509	0.982668	0.022
C25	0.8666(9)	1.1525(7)	0.9123(5)	0.0237(17)
H25	0.824097	1.183821	0.940115	0.028
C26	0.8218(9)	1.1406(7)	0.8477(5)	0.0246(17)
H26	0.750445	1.165609	0.831406	0.030
C27	0.8795(9)	1.0923(7)	0.8058(5)	0.0218(17)
H27	0.848428	1.084095	0.761016	0.026
C28	0.9837(8)	1.0563(7)	0.8309(4)	0.0164(15)
C29	0.5605(9)	0.3931(8)	0.7612(4)	0.0222(18)
H29A	0.477006	0.326360	0.755655	0.027
H29B	0.545222	0.458857	0.781701	0.027
C30	0.6819(9)	0.3784(7)	0.8026(4)	0.0184(15)
H30	0.710765	0.324730	0.777085	0.022
C31	0.6474(8)	0.3349(7)	0.8608(4)	0.0148(14)
C32	0.5420(9)	0.2423(7)	0.8651(4)	0.0212(17)
H32	0.478563	0.194302	0.827637	0.025
C33	0.5290(9)	0.2195(7)	0.9243(5)	0.0202(16)
H33	0.457090	0.154996	0.927607	0.024
C34	0.6209(9)	0.2906(7)	0.9793(4)	0.0190(16)
H34	0.609915	0.274627	1.019814	0.023
C35	0.7279(8)	0.3841(7)	0.9760(4)	0.0149(15)
H35	0.790987	0.431952	1.013622	0.018
C36	0.7409(8)	0.4064(6)	0.9161(4)	0.0121(14)
C37	0.8395(8)	0.4982(6)	0.8977(4)	0.0123(14)
C38	0.9484(8)	0.5917(7)	0.9345(4)	0.0172(16)
H38	0.972580	0.601594	0.979653	0.021
C39	1.0194(9)	0.6685(7)	0.9037(5)	0.0212(16)
H39	1.095037	0.732094	0.928016	0.025
C40	0.9846(9)	0.6563(7)	0.8393(5)	0.0219(16)
H40	1.034728	0.712519	0.819580	0.026
C41	0.8774(9)	0.5636(8)	0.8015(4)	0.0213(17)

H41	0.855387	0.554962	0.756359	0.026
C42	0.8037(8)	0.4845(7)	0.8310(4)	0.0159(15)
O3	0.6731(9)	0.7234(9)	0.6694(4)	0.0231(19)
O4	0.4538(9)	0.7282(9)	0.6773(4)	0.025(2)
O6	0.4865(11)	0.5515(7)	0.6708(4)	0.027(2)
O3A	0.664(4)	0.771(4)	0.6775(18)	0.026(5)
O4A	0.423(4)	0.670(4)	0.6787(17)	0.027(4)
O6A	0.572(4)	0.560(3)	0.6703(15)	0.025(4)

U_{eq} is defined as 1/3 of the trace of the orthogonalized U_{ij} tensor.

Supplementary Table 3: Bond lengths and angles for JessenFm_ultraphosphate_APEX_0m

Atom–Atom	Length [Å]
P1–O5	1.446(7)
P1–O4A	1.48(4)
P1–O3	1.530(9)
P1–O6	1.550(9)
P1–O4	1.564(9)
P1–O6A	1.68(3)
P1–O3A	1.70(4)
P1–Na1	3.401(2)
P2–O17	1.463(8)
P2–O16	1.467(8)
P2–O18	1.571(7)
P2–O3A	1.57(4)
P2–O3	1.650(9)
P3–O7	1.455(7)
P3–O19	1.467(9)
P3–O27	1.586(7)
P3–O6	1.642(9)
P3–O6A	1.66(4)
P3–Na1	3.455(2)
P4–O28	1.446(8)
P4–O2	1.472(7)
P4–O1	1.583(7)
P4–O4	1.634(9)
P4–O4A	1.73(4)
Na1–O7	2.324(7)
Na1–O7$^{\#1}$	2.324(7)
Na1–O5	2.339(6)
Na1–O5$^{\#1}$	2.339(6)
Na1–O8$^{\#1}$	2.355(7)
Na1–O8	2.355(7)
Na1–Na2	3.066(5)
Na1–Na2$^{\#1}$	3.066(5)
Na2–O11	2.375(10)
Na2–O9	2.381(10)
Na2–O10	2.391(9)
Na2–O7	2.410(9)
Na2–O8	2.494(9)
Na2–O5$^{\#1}$	2.526(8)
Na2–Na3	3.651(7)
Na3–O12	2.372(10)
Na3–O13	2.428(10)
Na3–O10	2.440(9)
Na3–O14	2.445(10)
Na3–O11	2.493(10)
Na3–O28$^{\#1}$	2.509(9)
Na3–Na4	3.673(5)
Na4–O14	2.360(9)
Na4–O14$^{\#2}$	2.360(9)
Na4–O15	2.416(10)
Na4–O15$^{\#2}$	2.416(10)
Na4–O13$^{\#2}$	2.458(9)
Na4–O13	2.458(9)
O1–C1	1.446(11)
O18–C15	1.417(11)
O27–C29	1.427(11)
C1–C2	1.514(12)
C1–H1A	0.9900
C1–H1B	0.9900
C2–C3	1.507(11)
C2–C14	1.508(11)
C2–H2	1.0000
C3–C4	1.374(12)
C3–C8	1.396(12)
C4–C5	1.389(13)
C4–H4	0.9500
C5–C6	1.378(13)
C5–H5	0.9500
C6–C7	1.377(13)
C6–H6	0.9500
C7–C8	1.395(12)
C7–H7	0.9500
C8–C9	1.440(12)
C9–C14	1.392(12)
C9–C10	1.411(12)
C10–C11	1.376(13)
C10–H10	0.9500
C11–C12	1.384(14)
C11–H11	0.9500
C12–C13	1.399(13)
C12–H12	0.9500
C13–C14	1.389(12)
C13–H13	0.9500
C15–C16	1.536(12)
C15–H15A	0.9900
C15–H15B	0.9900

C16–C17	1.509(12)
C16–C28	1.510(12)
C16–H16	1.0000
C17–C18	1.383(11)
C17–C22	1.396(11)
C18–C19	1.390(13)
C18–H18	0.9500
C19–C20	1.401(13)
C19–H19	0.9500
C20–C21	1.372(12)
C20–H20	0.9500
C21–C22	1.377(12)
C21–H21	0.9500
C22–C23	1.470(11)
C23–C24	1.372(11)
C23–C28	1.399(12)
C24–C25	1.378(13)
C24–H24	0.9500
C25–C26	1.370(15)
C25–H25	0.9500
C26–C27	1.391(13)
C26–H26	0.9500
C27–C28	1.392(12)
C27–H27	0.9500
C29–C30	1.516(12)
C29–H29A	0.9900
C29–H29B	0.9900
C30–C31	1.506(12)
C30–C42	1.507(12)
C30–H30	1.0000
C31–C32	1.373(12)
C31–C36	1.404(12)
C32–C33	1.377(13)
C32–H32	0.9500
C33–C34	1.392(13)
C33–H33	0.9500
C34–C35	1.382(12)
C34–H34	0.9500
C35–C36	1.389(11)
C35–H35	0.9500
C36–C37	1.459(11)
C37–C38	1.393(12)
C37–C42	1.404(12)
C38–C39	1.366(12)
C38–H38	0.9500
C39–C40	1.358(14)
C39–H39	0.9500
C40–C41	1.387(13)
C40–H40	0.9500
C41–C42	1.376(12)
C41–H41	0.9500

**Atom–Atom–Atom**	**Angle [°]**
O5–P1–O4A	118.5(15)
O5–P1–O3	115.1(4)
O5–P1–O6	115.7(4)
O3–P1–O6	105.7(5)
O5–P1–O4	114.8(4)
O3–P1–O4	101.8(5)
O6–P1–O4	102.0(5)
O5–P1–O6A	115.3(12)
O4A–P1–O6A	103(2)
O5–P1–O3A	117.1(14)
O4A–P1–O3A	102(2)
O6A–P1–O3A	97.8(19)
O5–P1–Na1	33.6(3)
O4A–P1–Na1	136.1(17)
O3–P1–Na1	105.6(4)
O6–P1–Na1	89.9(3)
O4–P1–Na1	145.8(3)
O6A–P1–Na1	81.9(11)
O3A–P1–Na1	120.8(13)
O17–P2–O16	119.9(5)
O17–P2–O18	107.6(4)
O16–P2–O18	111.5(4)
O17–P2–O3A	95.3(17)
O16–P2–O3A	126.6(17)
O18–P2–O3A	91.3(14)
O17–P2–O3	110.0(5)
O16–P2–O3	103.1(5)
O18–P2–O3	103.3(5)
O7–P3–O19	119.0(5)
O7–P3–O27	108.2(4)
O19–P3–O27	111.9(4)
O7–P3–O6	112.8(5)
O19–P3–O6	100.3(6)
O27–P3–O6	103.5(5)
O7–P3–O6A	95.7(13)
O19–P3–O6A	130.9(15)
O27–P3–O6A	85.3(13)
O7–P3–Na1	30.6(3)
O19–P3–Na1	111.7(3)
O27–P3–Na1	132.4(3)
O6–P3–Na1	86.6(3)
O6A–P3–Na1	80.4(11)
O28–P4–O2	119.5(5)
O28–P4–O1	109.2(4)
O2–P4–O1	110.6(4)
O28–P4–O4	103.3(6)
O2–P4–O4	111.5(5)
O1–P4–O4	101.1(4)
O28–P4–O4A	127.7(17)
O2–P4–O4A	92.9(15)
O1–P4–O4A	93.6(13)
O7–Na1–O7$^{\#1}$	180.0
O7–Na1–O5	84.9(2)
O7$^{\#1}$–Na1–O5	95.1(2)
O7–Na1–O5$^{\#1}$	95.1(2)

O7$^{\#1}$–Na1–O5$^{\#1}$	84.9(2)	O5$^{\#1}$–Na1–P3	112.20(16)
O5–Na1–O5$^{\#1}$	180.0(2)	O8$^{\#1}$–Na1–P3	86.18(19)
O7–Na1–O8$^{\#1}$	96.4(3)	O8–Na1–P3	93.82(19)
O7$^{\#1}$–Na1–O8$^{\#1}$	83.6(3)	Na2–Na1–P3	69.17(10)
O5–Na1–O8$^{\#1}$	79.9(2)	Na2$^{\#1}$–Na1–P3	110.83(10)
O5$^{\#1}$–Na1–O8$^{\#1}$	100.1(2)	P1$^{\#1}$–Na1–P3	129.65(6)
O7–Na1–O8	83.6(3)	P1–Na1–P3	50.35(6)
O7$^{\#1}$–Na1–O8	96.4(3)	P3$^{\#1}$–Na1–P3	180.00(8)
O5–Na1–O8	100.1(2)	O11–Na2–O9	111.2(4)
O5$^{\#1}$–Na1–O8	79.9(2)	O11–Na2–O10	84.1(3)
O8$^{\#1}$–Na1–O8	180.0	O9–Na2–O10	86.9(3)
O7–Na1–Na2	50.9(2)	O11–Na2–O7	103.1(3)
O7$^{\#1}$–Na1–Na2	129.1(2)	O9–Na2–O7	80.6(3)
O5–Na1–Na2	126.29(18)	O10–Na2–O7	167.2(4)
O5$^{\#1}$–Na1–Na2	53.70(18)	O11–Na2–O8	152.3(3)
O8$^{\#1}$–Na1–Na2	127.2(2)	O9–Na2–O8	96.5(3)
O8–Na1–Na2	52.8(2)	O10–Na2–O8	99.7(3)
O7–Na1–Na2$^{\#1}$	129.1(2)	O7–Na2–O8	79.0(3)
O7$^{\#1}$–Na1–Na2$^{\#1}$	50.9(2)	O11–Na2–O5$^{\#1}$	78.6(3)
O5–Na1–Na2$^{\#1}$	53.71(18)	O9–Na2–O5$^{\#1}$	166.5(3)
O5$^{\#1}$–Na1–Na2$^{\#1}$	126.30(18)	O10–Na2–O5$^{\#1}$	103.7(3)
O8$^{\#1}$–Na1–Na2$^{\#1}$	52.8(2)	O7–Na2–O5$^{\#1}$	88.3(3)
O8–Na1–Na2$^{\#1}$	127.2(2)	O8–Na2–O5$^{\#1}$	73.8(2)
Na2–Na1–Na2$^{\#1}$	180.0	O11–Na2–Na1	112.1(3)
O7–Na1–P1$^{\#1}$	113.89(19)	O9–Na2–Na1	118.3(3)
O7$^{\#1}$–Na1–P1$^{\#1}$	66.11(19)	O10–Na2–Na1	138.7(3)
O5–Na1–P1$^{\#1}$	159.97(16)	O7–Na2–Na1	48.42(18)
O5$^{\#1}$–Na1–P1$^{\#1}$	20.03(16)	O8–Na2–Na1	48.80(18)
O8$^{\#1}$–Na1–P1$^{\#1}$	90.77(18)	O5$^{\#1}$–Na2–Na1	48.26(16)
O8–Na1–P1$^{\#1}$	89.23(18)	O11–Na2–Na3	42.7(2)
Na2–Na1–P1$^{\#1}$	73.36(10)	O9–Na2–Na3	101.6(3)
Na2$^{\#1}$–Na1–P1$^{\#1}$	106.64(10)	O10–Na2–Na3	41.4(2)
O7–Na1–P1	66.11(19)	O7–Na2–Na3	144.5(3)
O7$^{\#1}$–Na1–P1	113.89(19)	O8–Na2–Na3	134.9(2)
O5–Na1–P1	20.03(16)	O5$^{\#1}$–Na2–Na3	91.9(2)
O5$^{\#1}$–Na1–P1	159.97(16)	Na1–Na2–Na3	139.89(18)
O8$^{\#1}$–Na1–P1	89.23(18)	O12–Na3–O13	90.1(3)
O8–Na1–P1	90.77(18)	O12–Na3–O10	167.8(4)
Na2–Na1–P1	106.64(10)	O13–Na3–O10	93.7(3)
Na2$^{\#1}$–Na1–P1	73.36(10)	O12–Na3–O14	92.5(3)
P1$^{\#1}$–Na1–P1	180.0	O13–Na3–O14	80.6(3)
O7–Na1–P3$^{\#1}$	161.43(18)	O10–Na3–O14	99.5(3)
O7$^{\#1}$–Na1–P3$^{\#1}$	18.57(18)	O12–Na3–O11	87.3(3)
O5–Na1–P3$^{\#1}$	112.21(16)	O13–Na3–O11	103.5(3)
O5$^{\#1}$–Na1–P3$^{\#1}$	67.79(16)	O10–Na3–O11	80.6(3)
O8$^{\#1}$–Na1–P3$^{\#1}$	93.82(19)	O14–Na3–O11	175.9(4)
O8–Na1–P3$^{\#1}$	86.18(19)	O12–Na3–O28$^{\#1}$	91.6(3)
Na2–Na1–P3$^{\#1}$	110.83(10)	O13–Na3–O28$^{\#1}$	161.1(4)
Na2$^{\#1}$–Na1–P3$^{\#1}$	69.17(10)	O10–Na3–O28$^{\#1}$	88.4(3)
P1$^{\#1}$–Na1–P3$^{\#1}$	50.35(6)	O14–Na3–O28$^{\#1}$	80.6(3)
P1–Na1–P3$^{\#1}$	129.65(6)	O11–Na3–O28$^{\#1}$	95.4(3)
O7–Na1–P3	18.57(18)	O12–Na3–Na2	127.4(3)
O7$^{\#1}$–Na1–P3	161.43(18)	O13–Na3–Na2	101.1(3)
O5–Na1–P3	67.79(16)	O10–Na3–Na2	40.4(2)

O14–Na3–Na2	139.8(3)	O1–C1–H1A	110.2
O11–Na3–Na2	40.2(2)	C2–C1–H1A	110.2
O28$^{\#1}$–Na3–Na2	92.7(2)	O1–C1–H1B	110.2
O12–Na3–Na4	87.9(3)	C2–C1–H1B	110.2
O13–Na3–Na4	41.6(2)	H1A–C1–H1B	108.5
O10–Na3–Na4	102.7(2)	C3–C2–C14	102.1(7)
O14–Na3–Na4	39.3(2)	C3–C2–C1	113.6(7)
O11–Na3–Na4	144.7(3)	C14–C2–C1	110.8(7)
O28$^{\#1}$–Na3–Na4	119.7(3)	C3–C2–H2	110.0
Na2–Na3–Na4	132.66(16)	C14–C2–H2	110.0
O14–Na4–O14$^{\#2}$	180.0	C1–C2–H2	110.0
O14–Na4–O15	87.4(4)	C4–C3–C8	119.8(8)
O14$^{\#2}$–Na4–O15	92.6(4)	C4–C3–C2	130.3(8)
O14–Na4–O15$^{\#2}$	92.6(4)	C8–C3–C2	109.9(7)
O14$^{\#2}$–Na4–O15$^{\#2}$	87.4(4)	C3–C4–C5	120.0(8)
O15–Na4–O15$^{\#2}$	180.0	C3–C4–H4	120.0
O14–Na4–O13$^{\#2}$	98.3(3)	C5–C4–H4	120.0
O14$^{\#2}$–Na4–O13$^{\#2}$	81.7(3)	C6–C5–C4	120.0(8)
O15–Na4–O13$^{\#2}$	81.6(3)	C6–C5–H5	120.0
O15$^{\#2}$–Na4–O13$^{\#2}$	98.4(3)	C4–C5–H5	120.0
O14–Na4–O13	81.7(3)	C7–C6–C5	120.9(8)
O14$^{\#2}$–Na4–O13	98.3(3)	C7–C6–H6	119.5
O15–Na4–O13	98.4(3)	C5–C6–H6	119.5
O15$^{\#2}$–Na4–O13	81.6(3)	C6–C7–C8	119.0(8)
O13$^{\#2}$–Na4–O13	180.0	C6–C7–H7	120.5
O14–Na4–Na3	41.0(2)	C8–C7–H7	120.5
O14$^{\#2}$–Na4–Na3	139.0(2)	C7–C8–C3	120.2(8)
O15–Na4–Na3	97.8(3)	C7–C8–C9	130.8(8)
O15$^{\#2}$–Na4–Na3	82.2(3)	C3–C8–C9	109.0(7)
O13$^{\#2}$–Na4–Na3	139.0(2)	C14–C9–C10	119.6(8)
O13–Na4–Na3	41.0(2)	C14–C9–C8	108.9(7)
O14–Na4–Na3$^{\#2}$	139.0(2)	C10–C9–C8	131.5(8)
O14$^{\#2}$–Na4–Na3$^{\#2}$	41.0(2)	C11–C10–C9	118.9(9)
O15–Na4–Na3$^{\#2}$	82.2(3)	C11–C10–H10	120.5
O15$^{\#2}$–Na4–Na3$^{\#2}$	97.8(3)	C9–C10–H10	120.5
O13$^{\#2}$–Na4–Na3$^{\#2}$	41.0(2)	C10–C11–C12	120.9(9)
O13–Na4–Na3$^{\#2}$	139.0(2)	C10–C11–H11	119.6
Na3–Na4–Na3$^{\#2}$	180.0	C12–C11–H11	119.6
C1–O1–P4	118.9(5)	C11–C12–C13	121.3(8)
P1–O5–Na1	126.3(4)	C11–C12–H12	119.4
P1–O5–Na2$^{\#1}$	153.3(4)	C13–C12–H12	119.4
Na1–O5–Na2$^{\#1}$	78.0(2)	C14–C13–C12	117.7(8)
P3–O7–Na1	130.8(4)	C14–C13–H13	121.1
P3–O7–Na2	147.0(5)	C12–C13–H13	121.1
Na1–O7–Na2	80.7(2)	C13–C14–C9	121.6(8)
Na1–O8–Na2	78.4(2)	C13–C14–C2	128.3(8)
Na2–O10–Na3	98.1(3)	C9–C14–C2	110.1(7)
Na2–O11–Na3	97.1(3)	O18–C15–C16	107.4(7)
Na3–O13–Na4	97.4(3)	O18–C15–H15A	110.2
Na4–O14–Na3	99.7(3)	C16–C15–H15A	110.2
C15–O18–P2	123.1(6)	O18–C15–H15B	110.2
C29–O27–P3	120.9(6)	C16–C15–H15B	110.2
P4–O28–Na3$^{\#1}$	126.1(5)	H15A–C15–H15B	108.5
O1–C1–C2	107.7(7)	C17–C16–C28	101.3(7)

C17–C16–C15	112.3(7)
C28–C16–C15	112.8(7)
C17–C16–H16	110.0
C28–C16–H16	110.0
C15–C16–H16	110.0
C18–C17–C22	119.9(8)
C18–C17–C16	128.8(8)
C22–C17–C16	111.3(7)
C17–C18–C19	118.7(8)
C17–C18–H18	120.6
C19–C18–H18	120.6
C18–C19–C20	120.7(8)
C18–C19–H19	119.6
C20–C19–H19	119.6
C21–C20–C19	120.1(8)
C21–C20–H20	119.9
C19–C20–H20	119.9
C20–C21–C22	119.3(8)
C20–C21–H21	120.4
C22–C21–H21	120.4
C21–C22–C17	121.2(8)
C21–C22–C23	130.6(8)
C17–C22–C23	108.1(7)
C24–C23–C28	121.0(8)
C24–C23–C22	131.0(8)
C28–C23–C22	108.0(7)
C23–C24–C25	118.8(9)
C23–C24–H24	120.6
C25–C24–H24	120.6
C26–C25–C24	121.2(8)
C26–C25–H25	119.4
C24–C25–H25	119.4
C25–C26–C27	120.7(9)
C25–C26–H26	119.6
C27–C26–H26	119.6
C26–C27–C28	118.5(9)
C26–C27–H27	120.7
C28–C27–H27	120.7
C27–C28–C23	119.7(8)
C27–C28–C16	129.0(8)
C23–C28–C16	111.3(7)
O27–C29–C30	108.4(7)
O27–C29–H29A	110.0
C30–C29–H29A	110.0
O27–C29–H29B	110.0
C30–C29–H29B	110.0
H29A–C29–H29B	108.4
C31–C30–C42	102.6(7)
C31–C30–C29	111.5(7)
C42–C30–C29	115.0(7)
C31–C30–H30	109.2
C42–C30–H30	109.2
C29–C30–H30	109.2
C32–C31–C36	120.4(8)
C32–C31–C30	129.6(8)
C36–C31–C30	110.0(7)
C31–C32–C33	119.5(8)
C31–C32–H32	120.3
C33–C32–H32	120.3
C32–C33–C34	120.2(8)
C32–C33–H33	119.9
C34–C33–H33	119.9
C35–C34–C33	121.2(8)
C35–C34–H34	119.4
C33–C34–H34	119.4
C34–C35–C36	118.3(8)
C34–C35–H35	120.9
C36–C35–H35	120.9
C35–C36–C31	120.4(7)
C35–C36–C37	130.8(8)
C31–C36–C37	108.8(7)
C38–C37–C42	120.6(8)
C38–C37–C36	131.0(8)
C42–C37–C36	108.3(7)
C39–C38–C37	118.1(8)
C39–C38–H38	121.0
C37–C38–H38	121.0
C40–C39–C38	121.6(8)
C40–C39–H39	119.2
C38–C39–H39	119.2
C39–C40–C41	121.5(8)
C39–C40–H40	119.3
C41–C40–H40	119.3
C42–C41–C40	118.4(9)
C42–C41–H41	120.8
C40–C41–H41	120.8
C41–C42–C37	119.8(8)
C41–C42–C30	129.9(8)
C37–C42–C30	110.3(7)
P1–O3–P2	129.2(6)
P1–O4–P4	133.4(6)
P1–O6–P3	132.1(6)
P2–O3A–P1	122(2)
P1–O4A–P4	132(3)
P3–O6A–P1	122(2)

Symmetry transformations used to generate equivalent atoms:
#1: 1-X, 1-Y, 1-Z; #2: 2-X, -Y, 1-Z.

NMR-spectra

Supplementary Fig. 16 | **^{1}H-NMR** (400 MHz, CD_3CN), compound **20**:

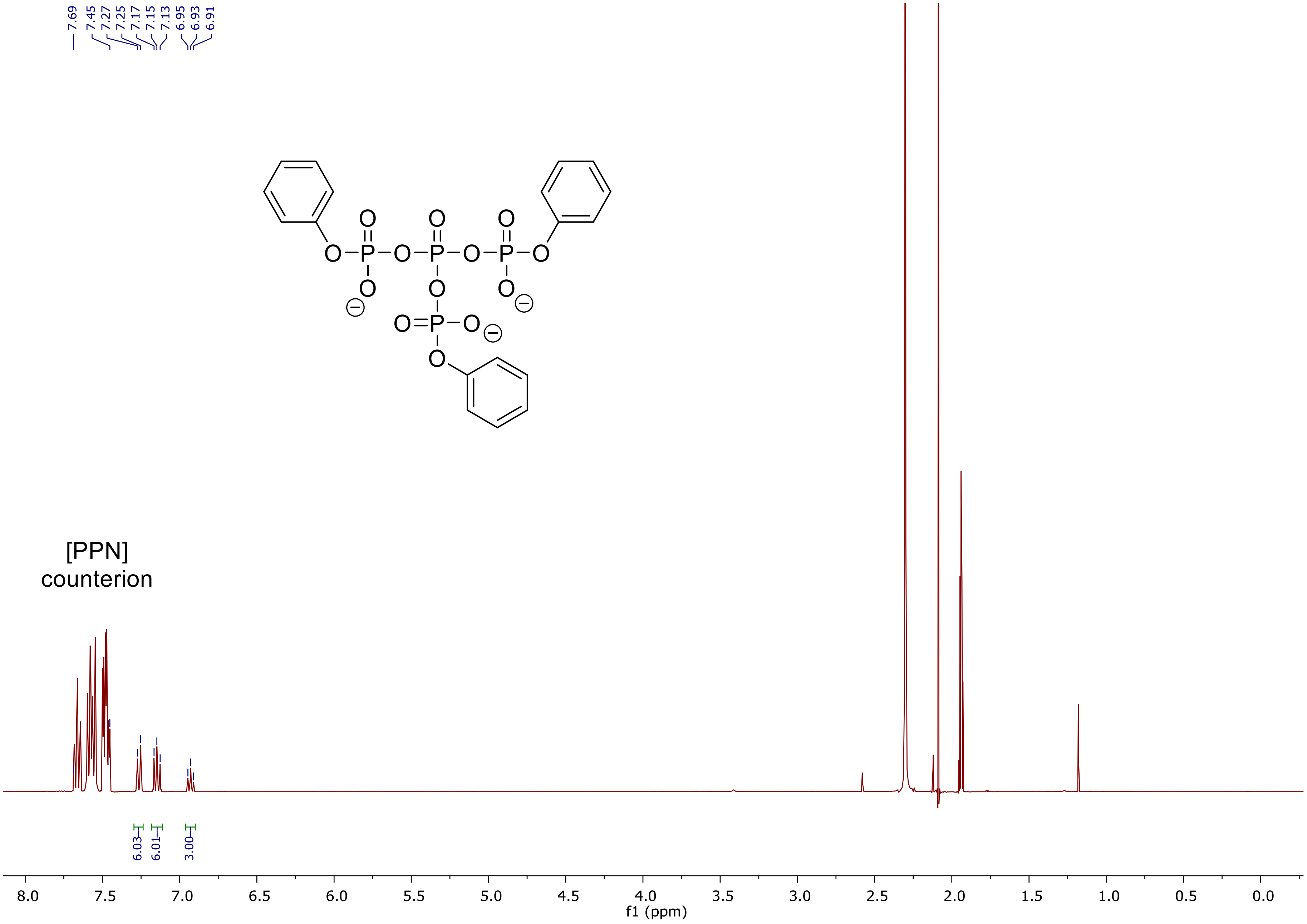

Supplementary Fig. 17| **^{31}P{^{1}H}-NMR** (162 MHz, CD_3CN), compound **20**:

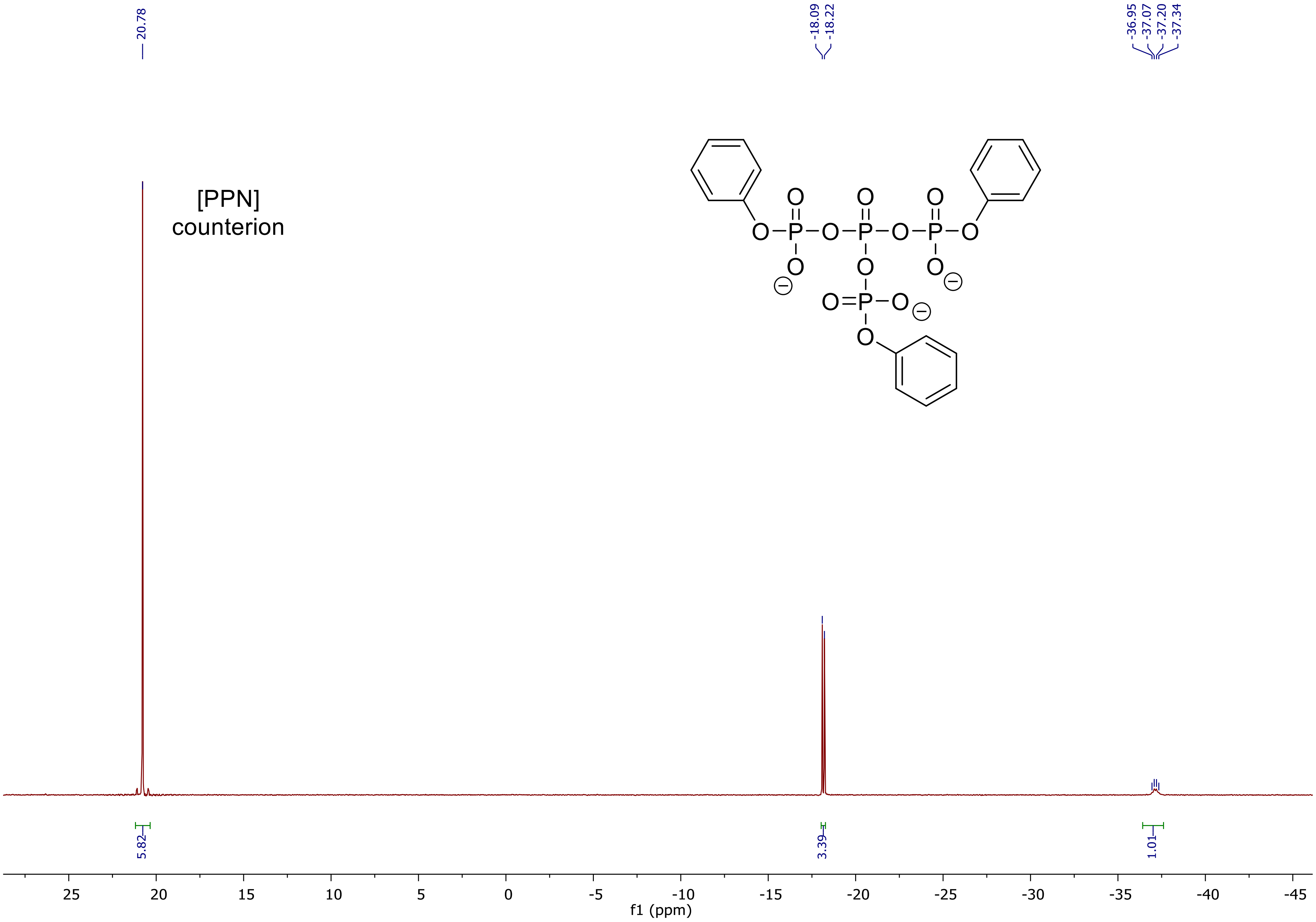

Supplementary Fig. 18│ **^{13}C-NMR** (101 MHz, CD_3CN), compound **20**:

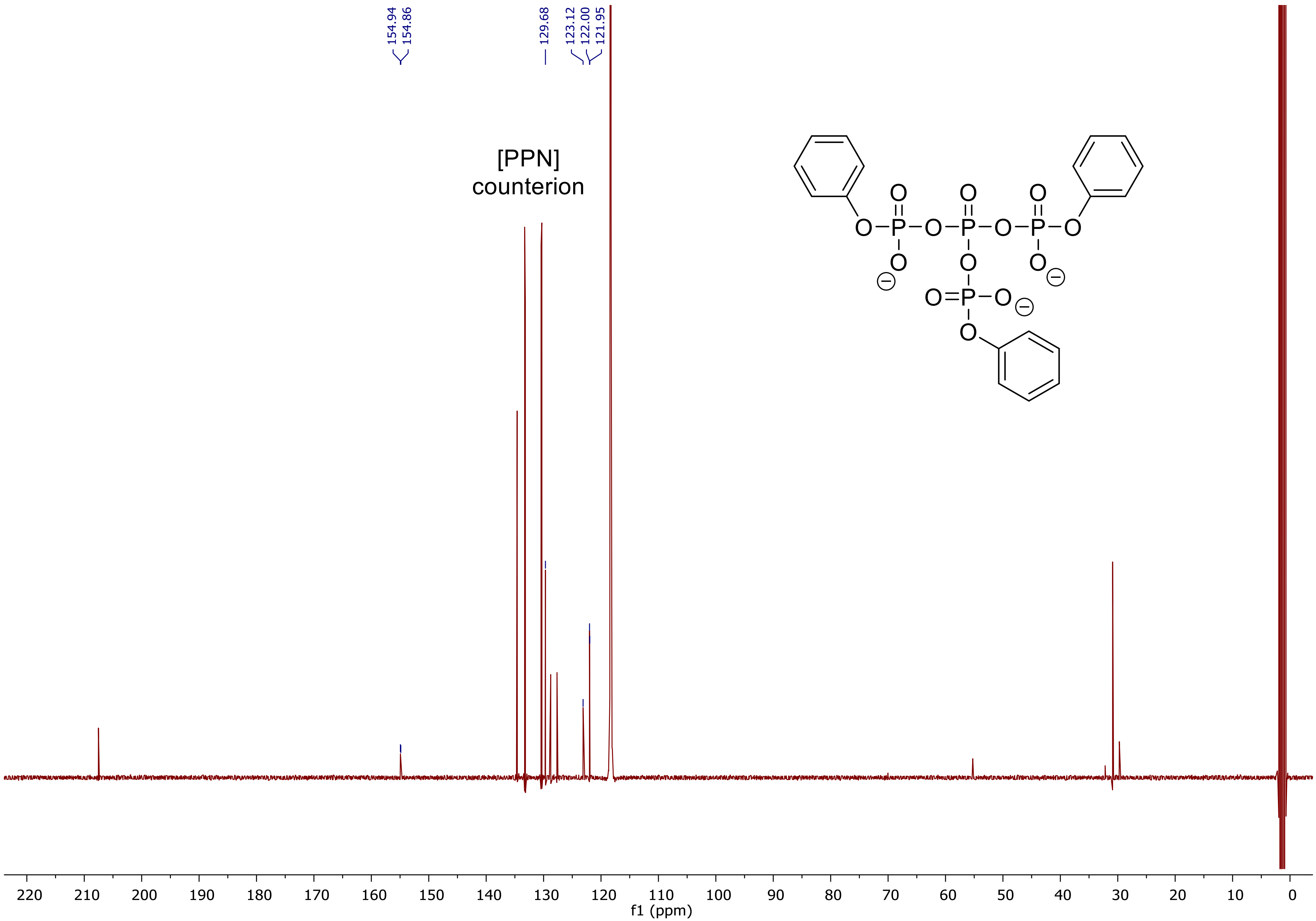

Supplementary Fig. 19 | **^{1}H-NMR** (400 MHz, DMF-d_7), compound **19**:

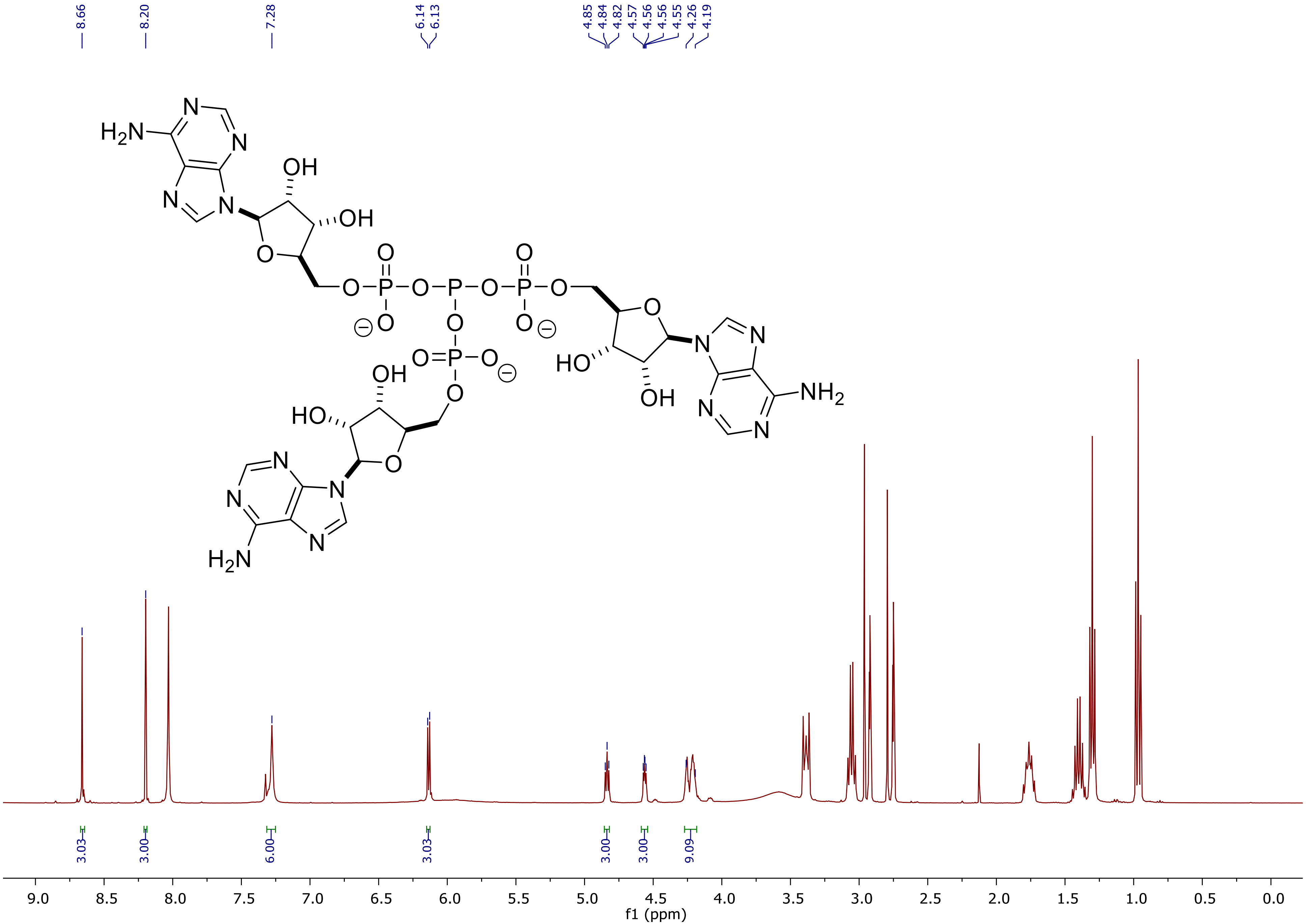

Supplementary Fig. 20| **$^{31}P\{^1H\}$-NMR** (162 MHz, DMF-d$_7$), compound **19**:

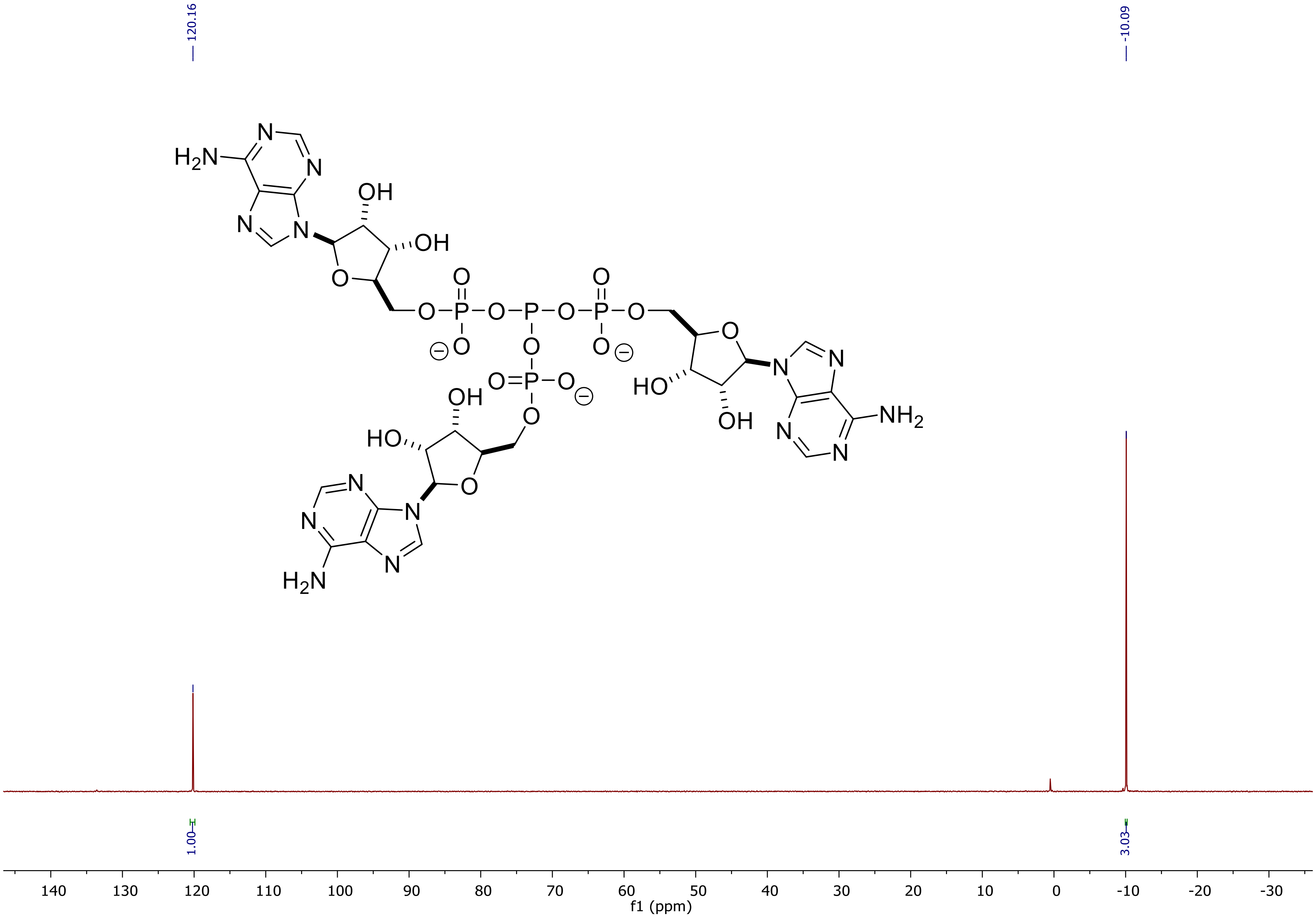

Supplementary Fig. 21 | **^{31}P-NMR** (162 MHz, DMF-d$_7$), compound **19**:

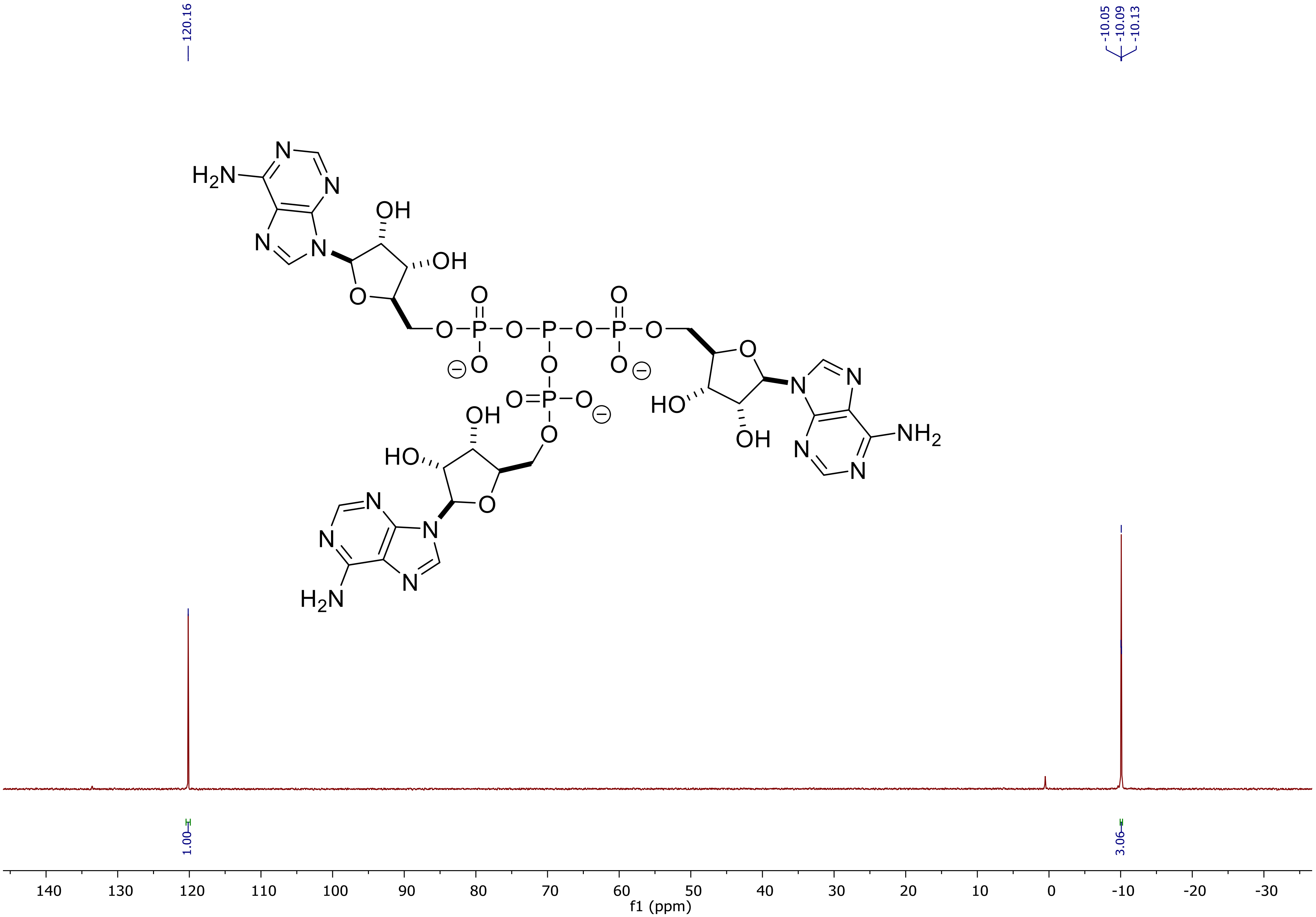

Supplementary Fig. 22| **^{1}H-NMR** (400 MHz, DMF-d$_7$), compound **21**:

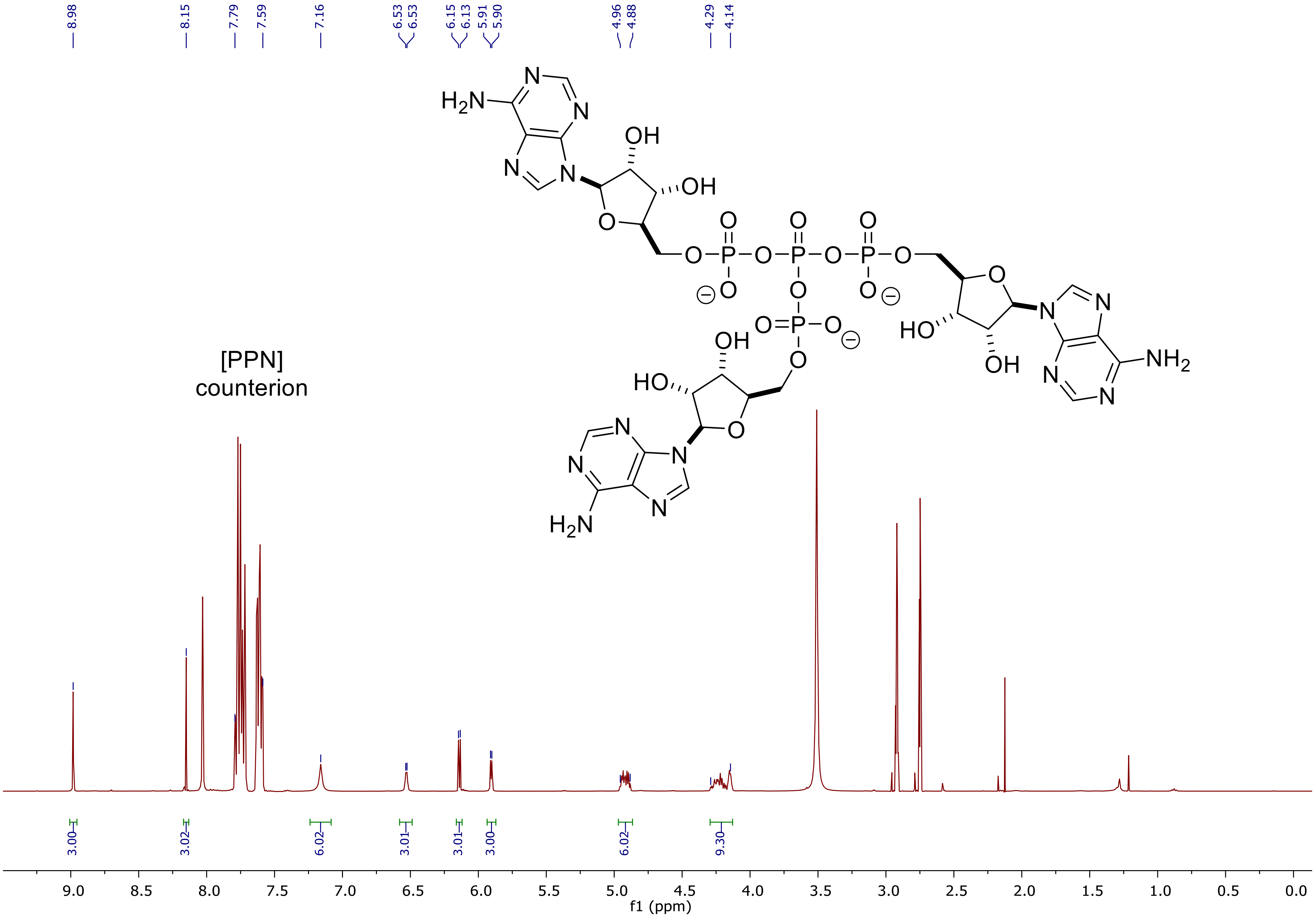

Supplementary Fig. 23 | **$^{31}P\{^{1}H\}$-NMR** (162 MHz, DMF-d_7), compound **21**:

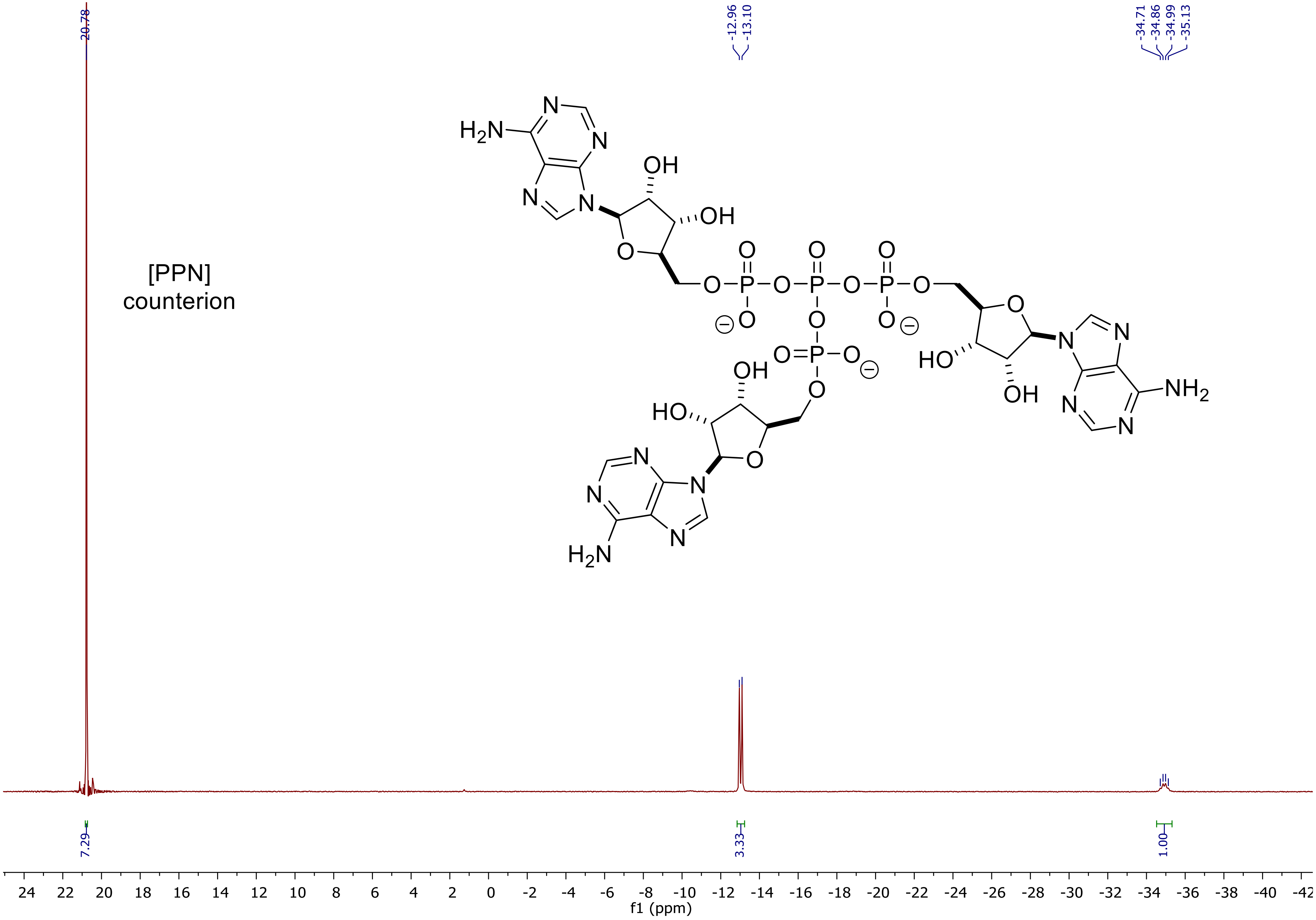

Supplementary Fig. 24 | **^{31}P-NMR** (162 MHz, DMF-d$_7$), compound **21**:

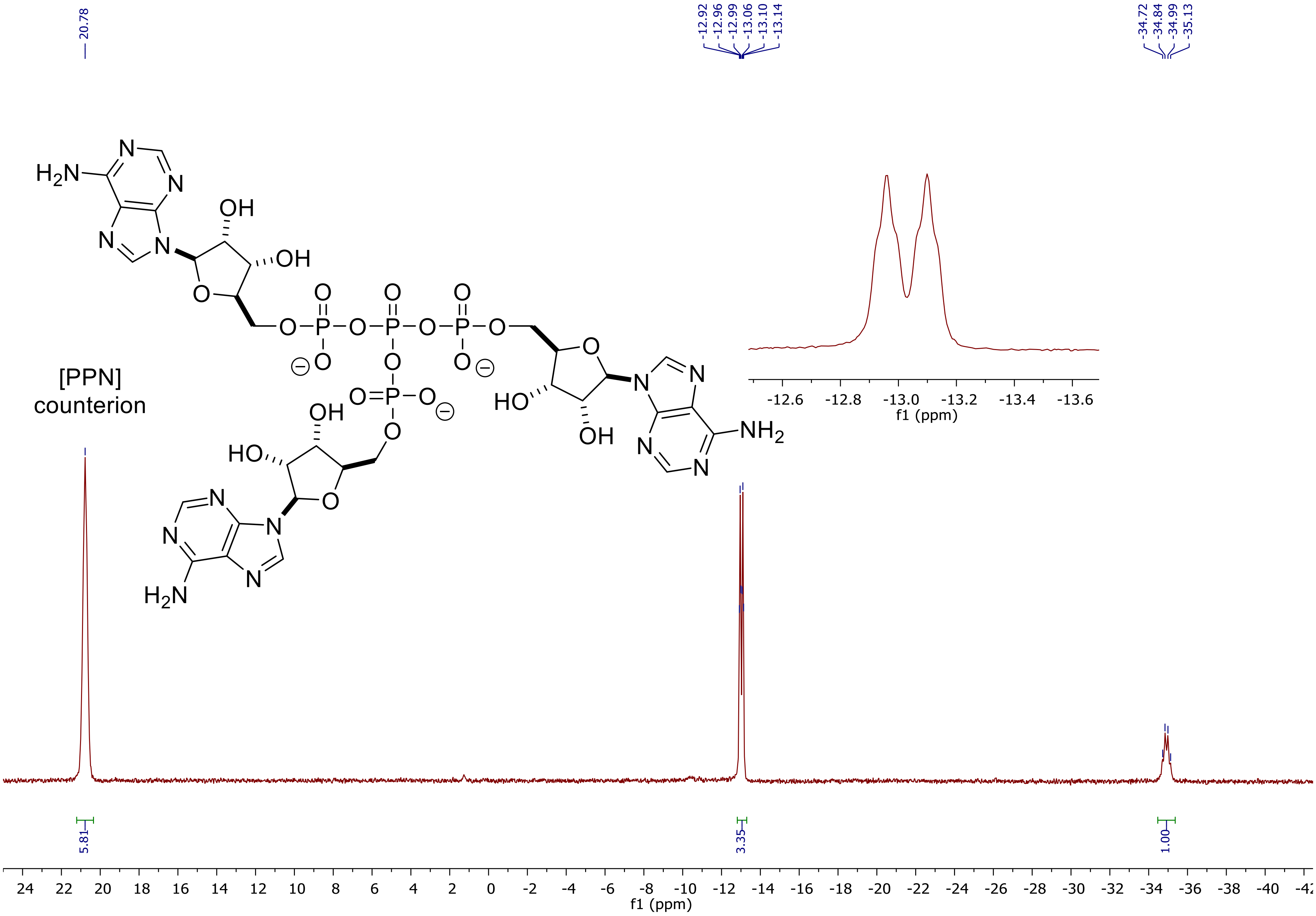

Supplementary Fig. 25 | **^{13}C-NMR** (101 MHz, DMF-d$_7$), compound **21**:

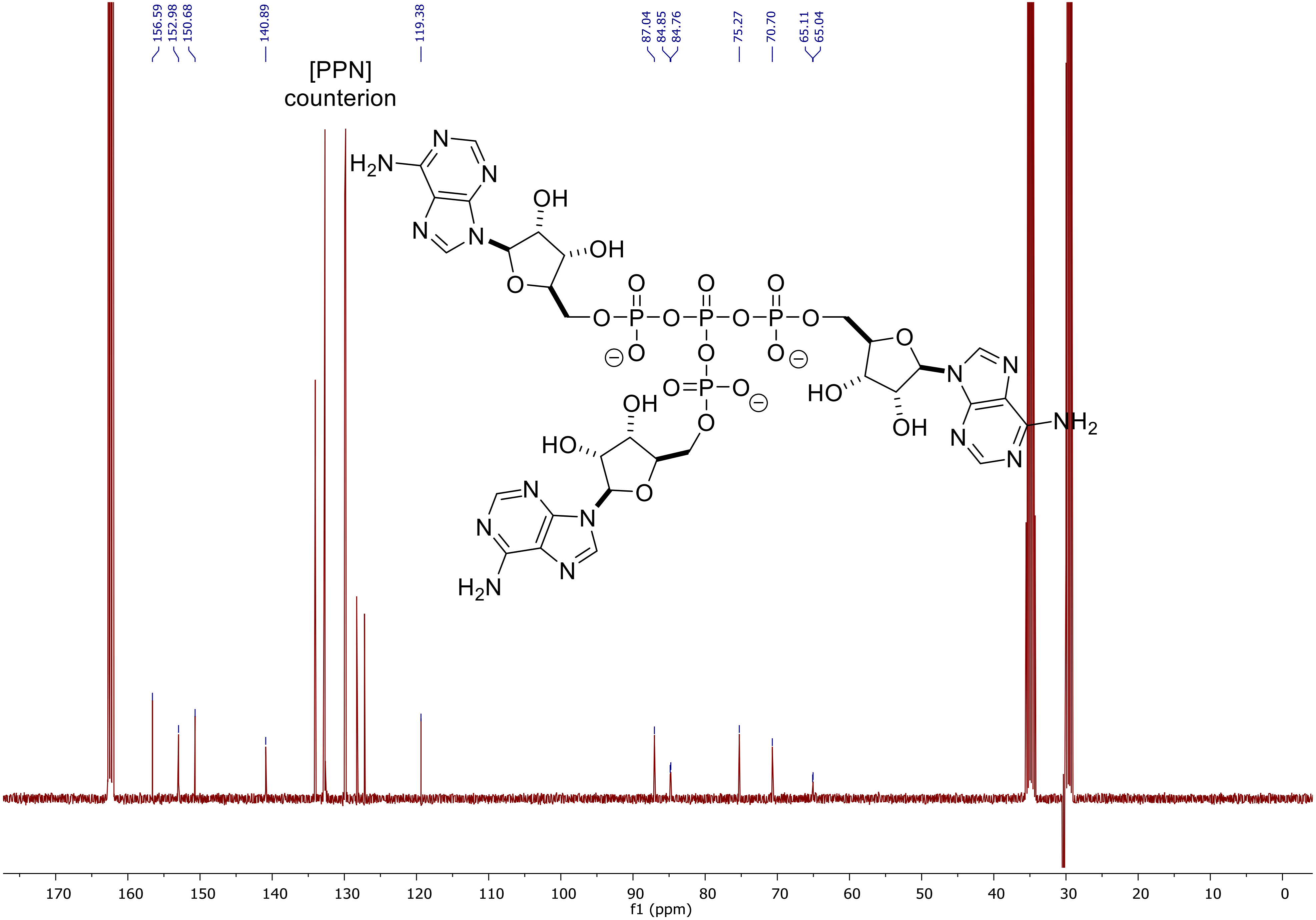

Supplementary Fig. 26| [1]**H-NMR** (400 MHz, D_2O, presat), compound **22**:

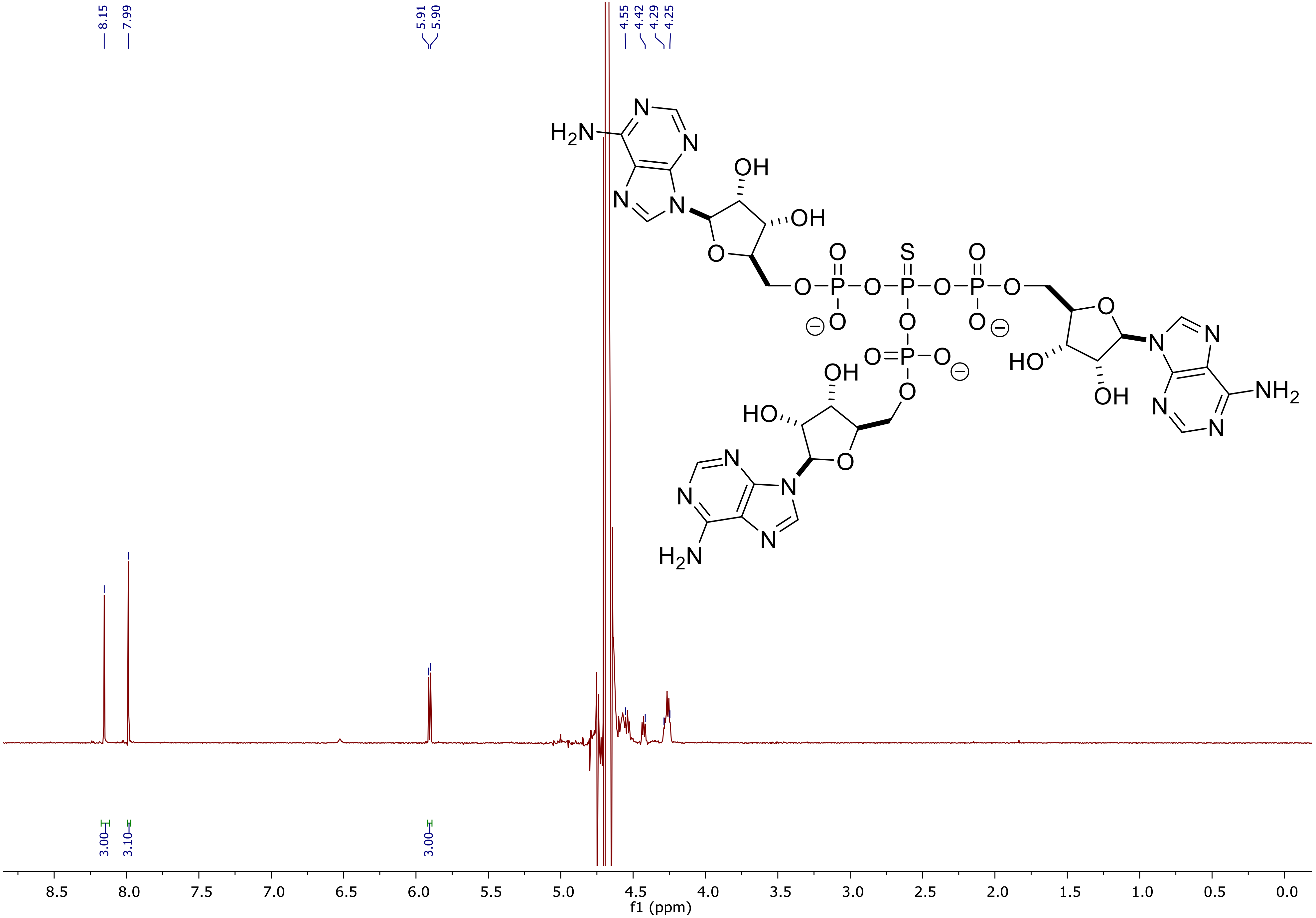

Supplementary Fig. 27| **$^{31}P\{^{1}H\}$-NMR** (162 MHz, D_2O), compound **22**:

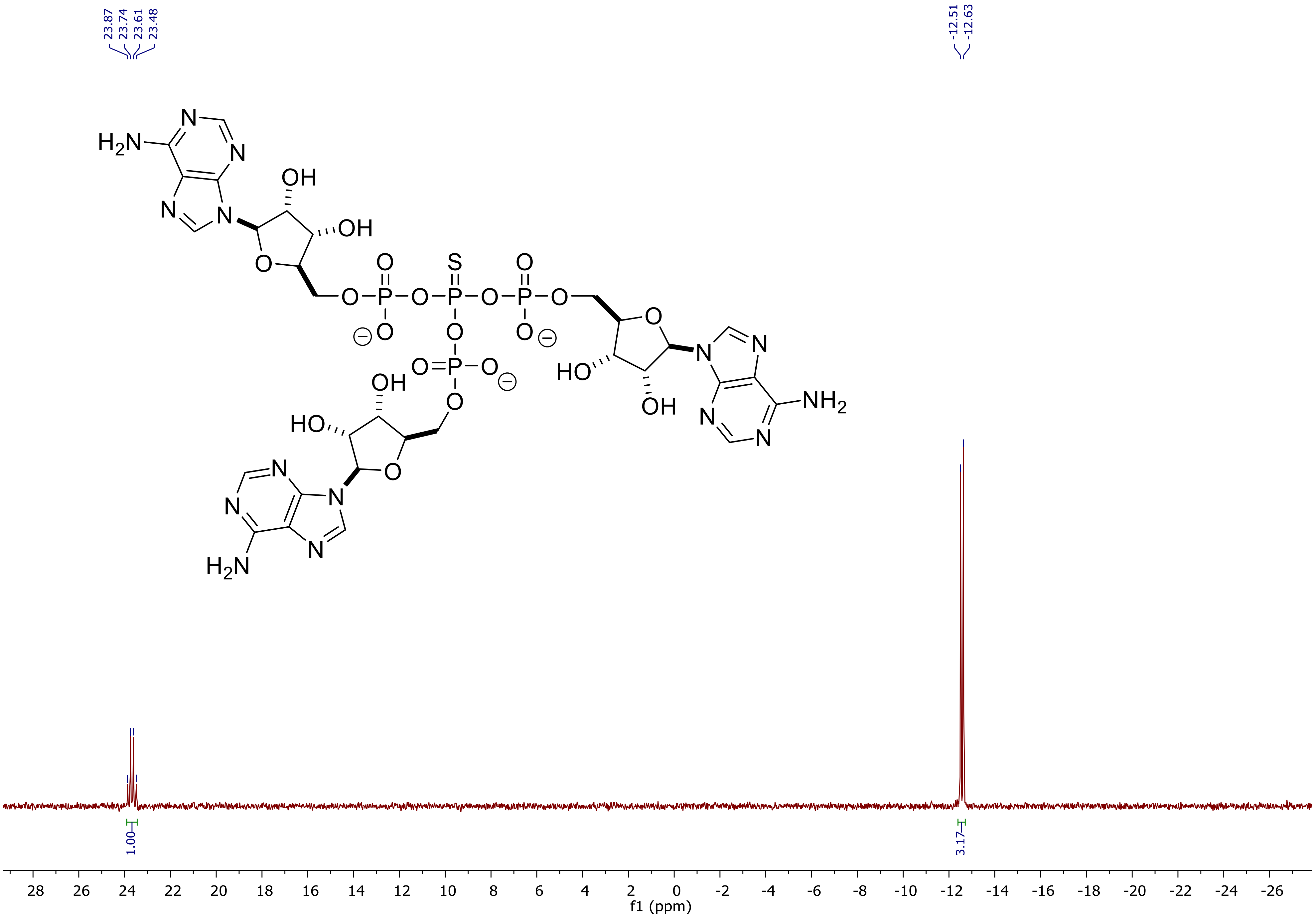

Supplementary Fig. 28 | **^{1}H-NMR** (400 MHz, D_2O, presat), compound **23**:

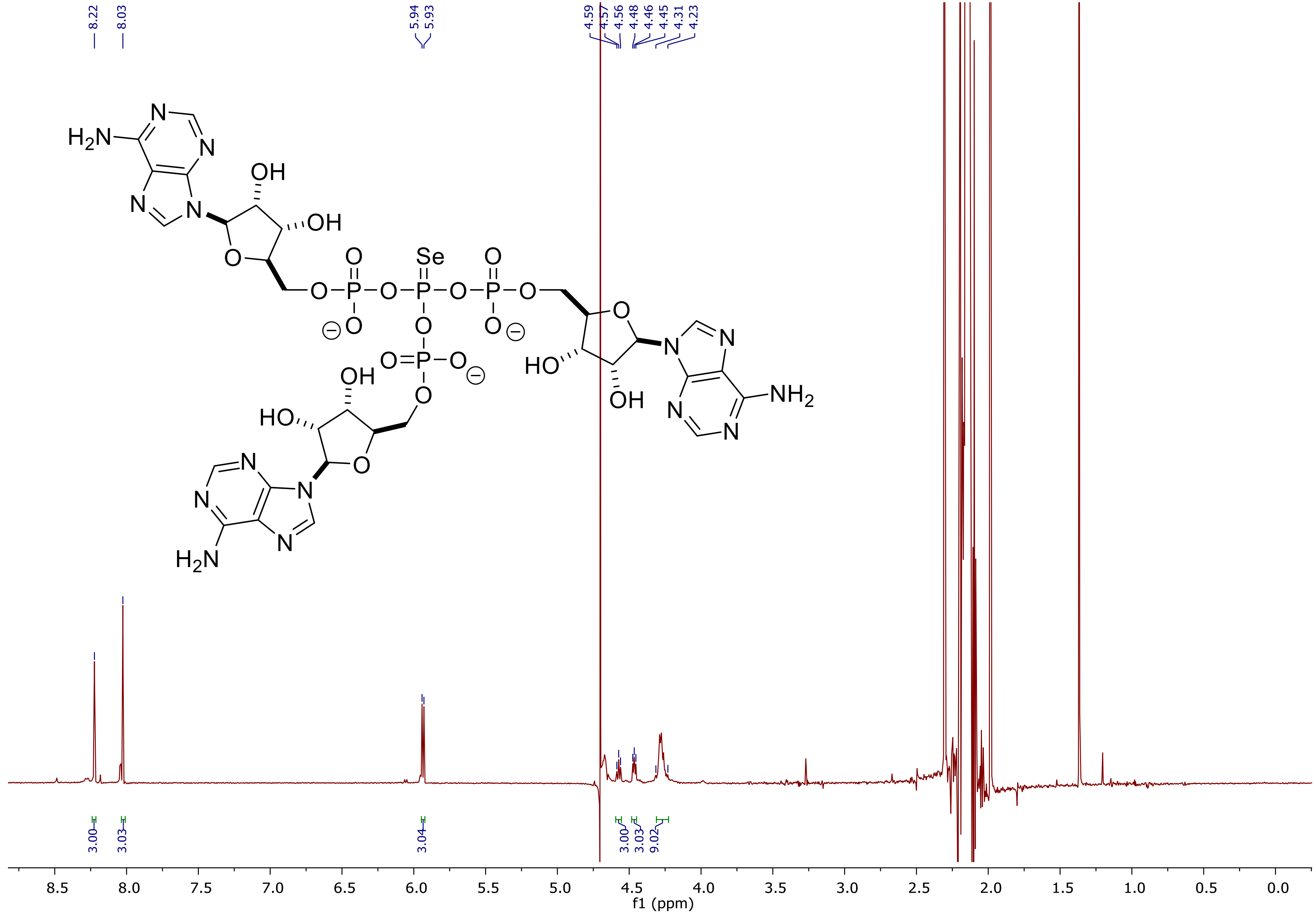

Supplementary Fig. 29 | $^{31}P\{^1H\}$-NMR (162 MHz, D_2O), compound **23**:

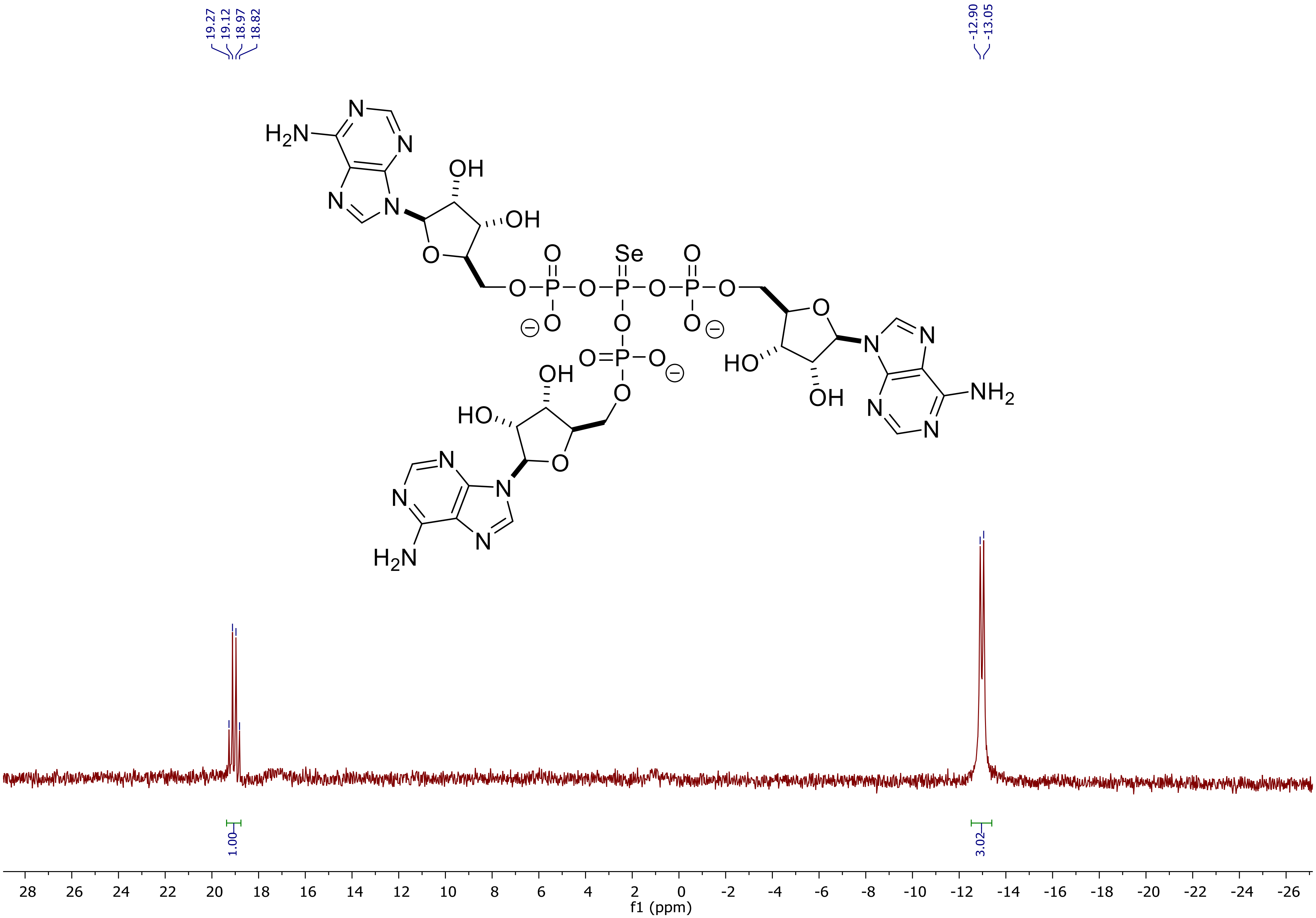

Supplementary Fig. 30| **^{31}P-NMR** (162 MHz, D_2O), compound **23**:

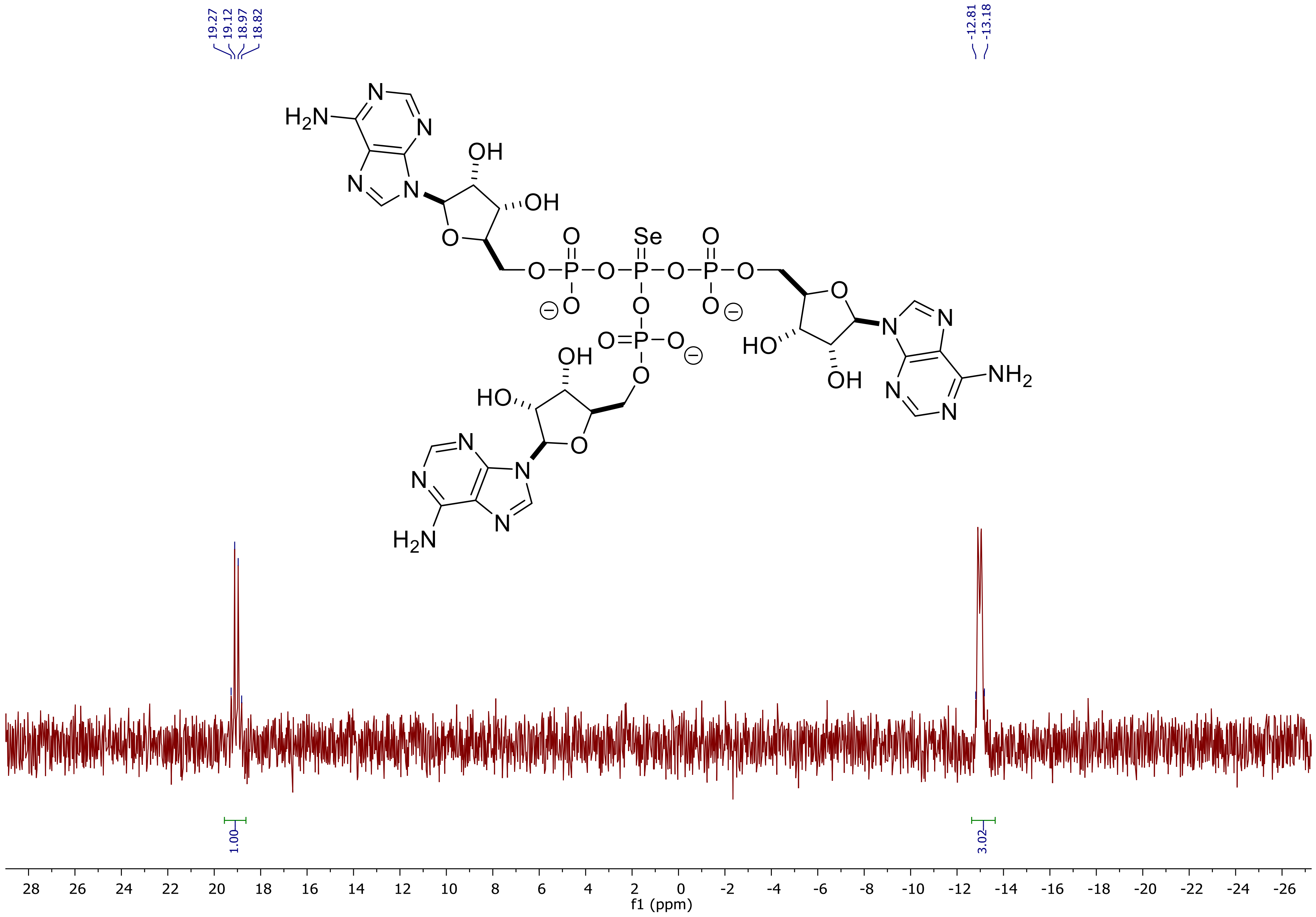

Supplementary Fig. 31| **^{1}H-NMR** (400 MHz, D_2O, presat), compound **24**:

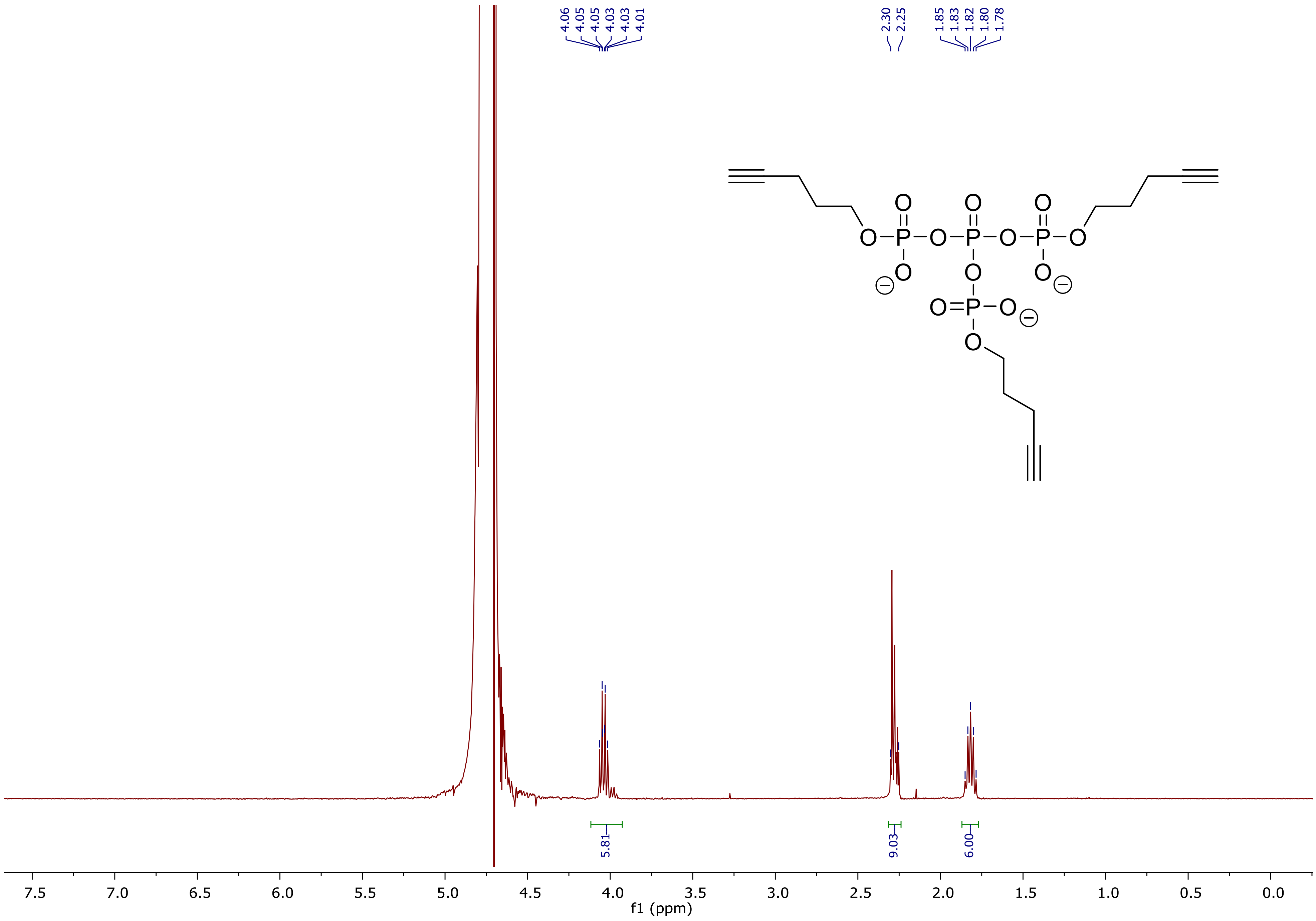

Supplementary Fig. 32 | **^{31}P{^{1}H}-NMR** (162 MHz, D_2O), compound **24**:

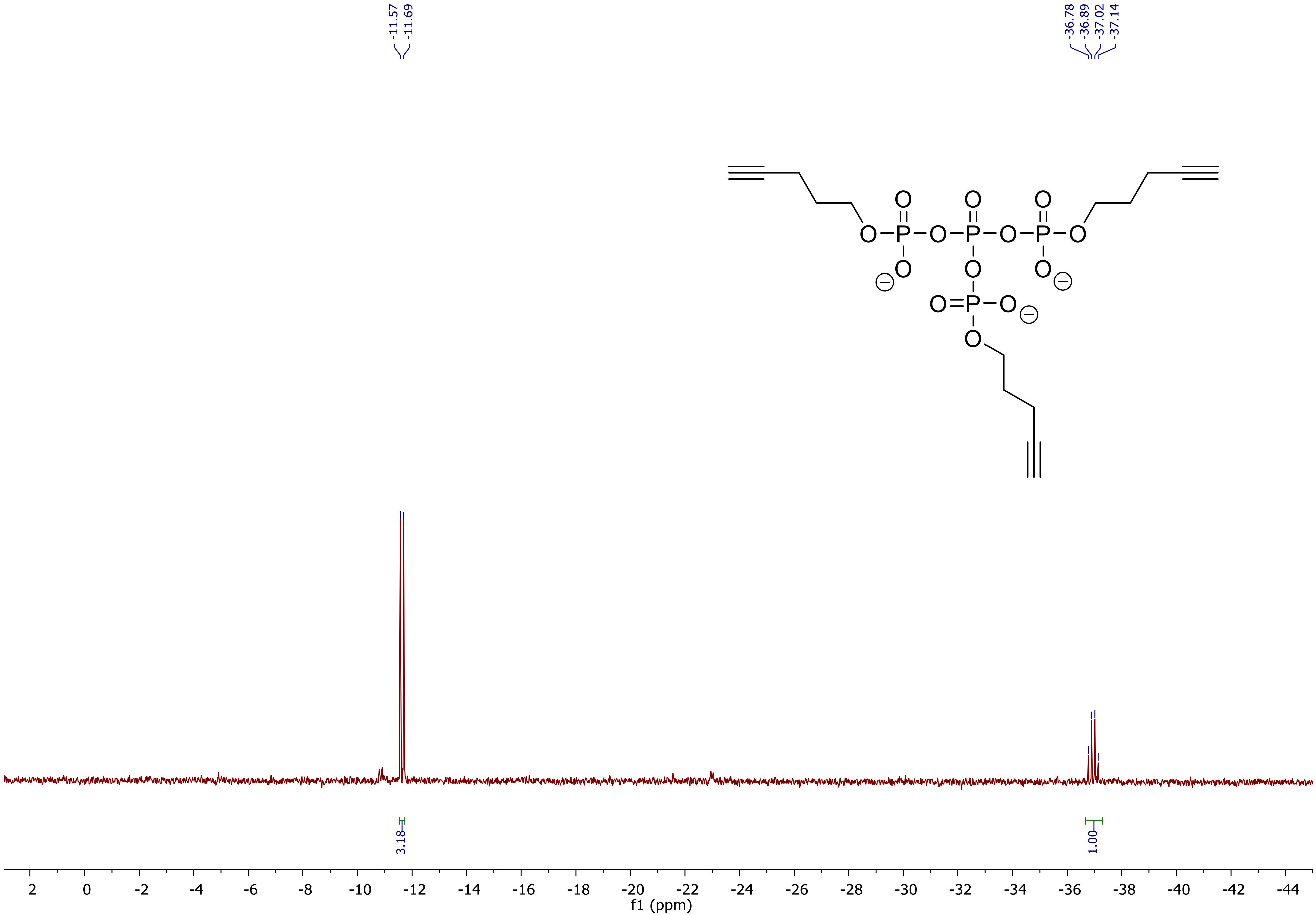

Supplementary Fig. 33 | **^{31}P-NMR** (162 MHz, D_2O), compound **24**:

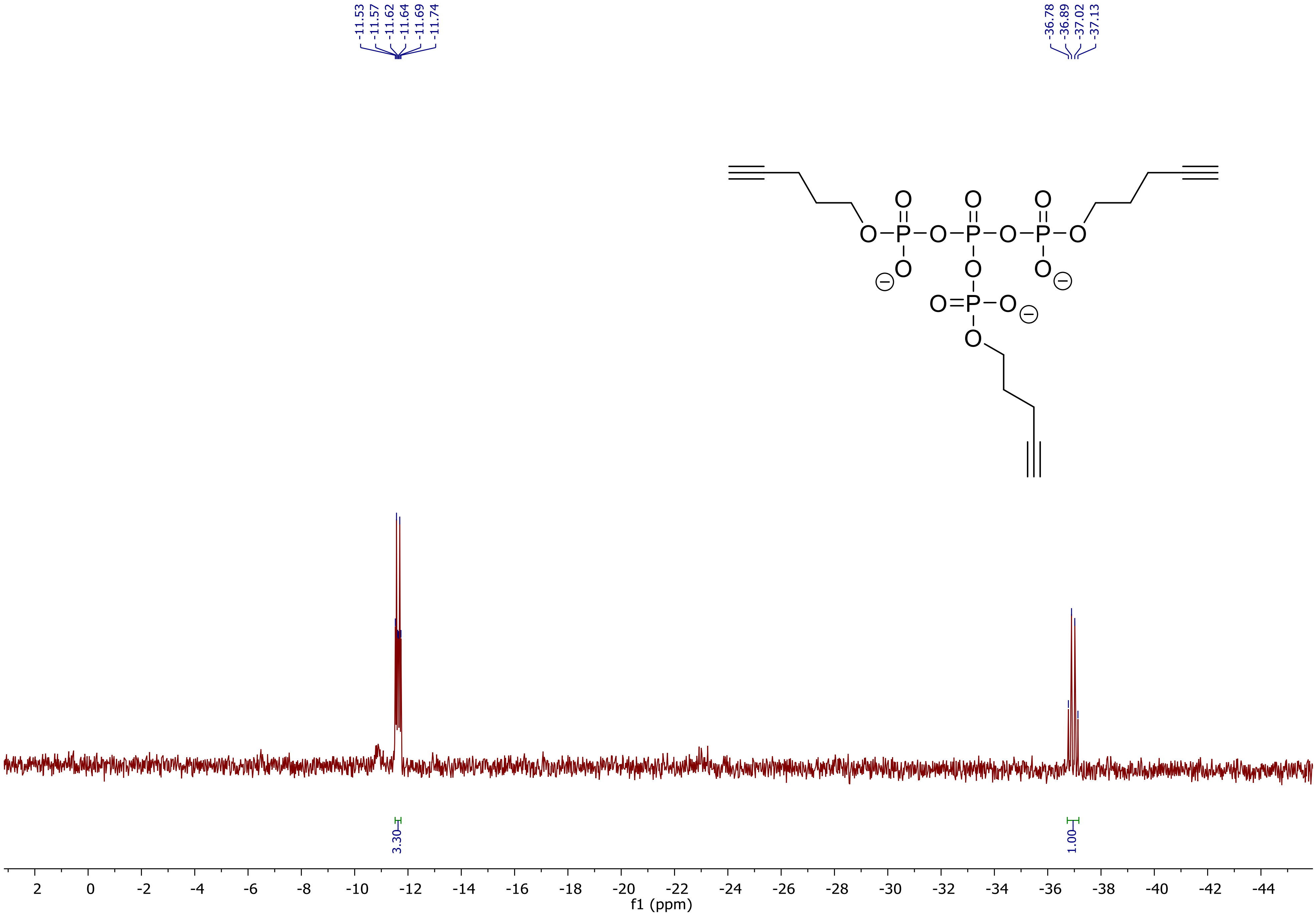

Supplementary Fig. 34| [1]**H-NMR** (400 MHz, CD_3CN), compound **25**:

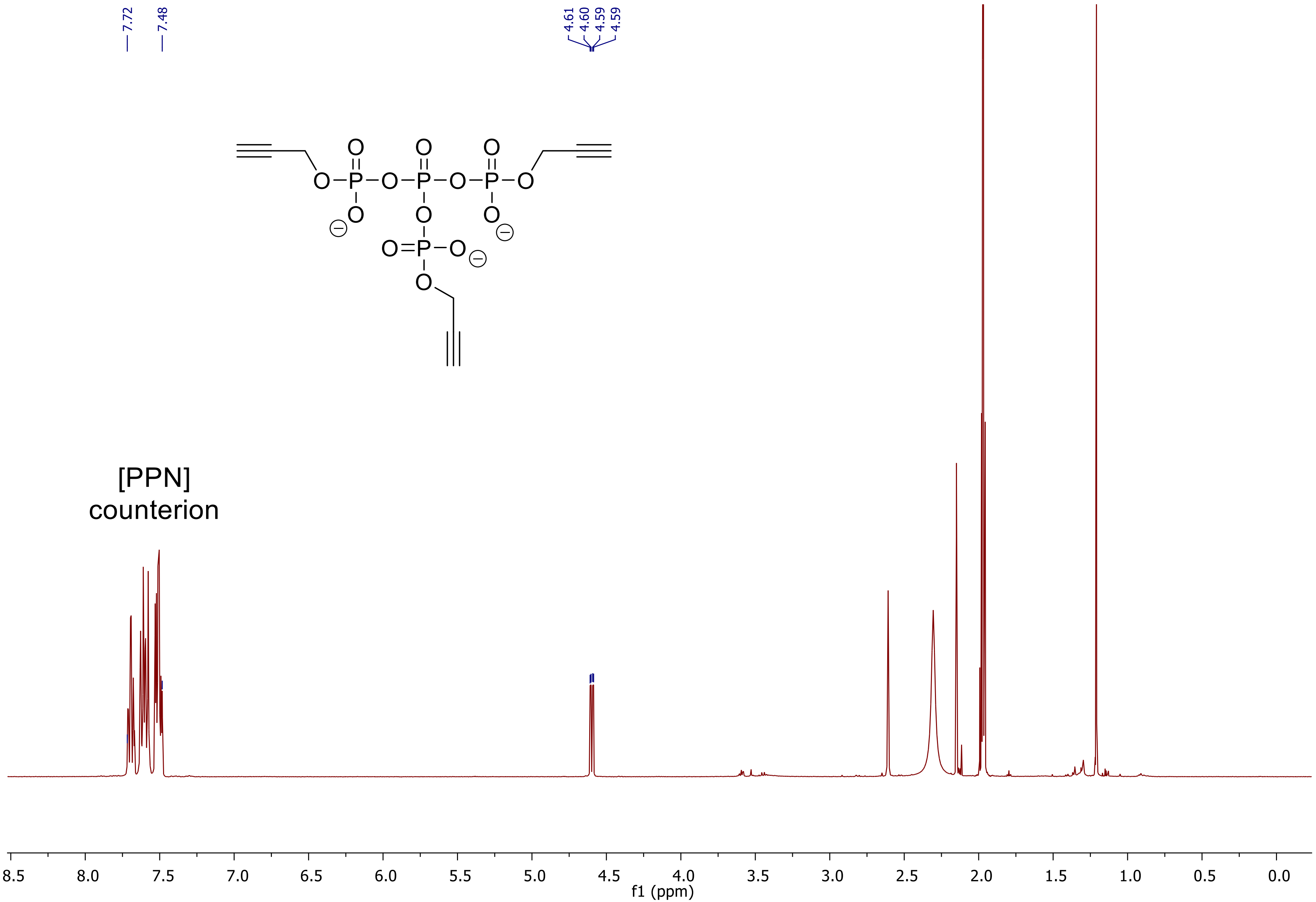

Supplementary Fig. 35 | **^{31}P{^{1}H}-NMR** (162 MHz, CD_3CN), compound **25**:

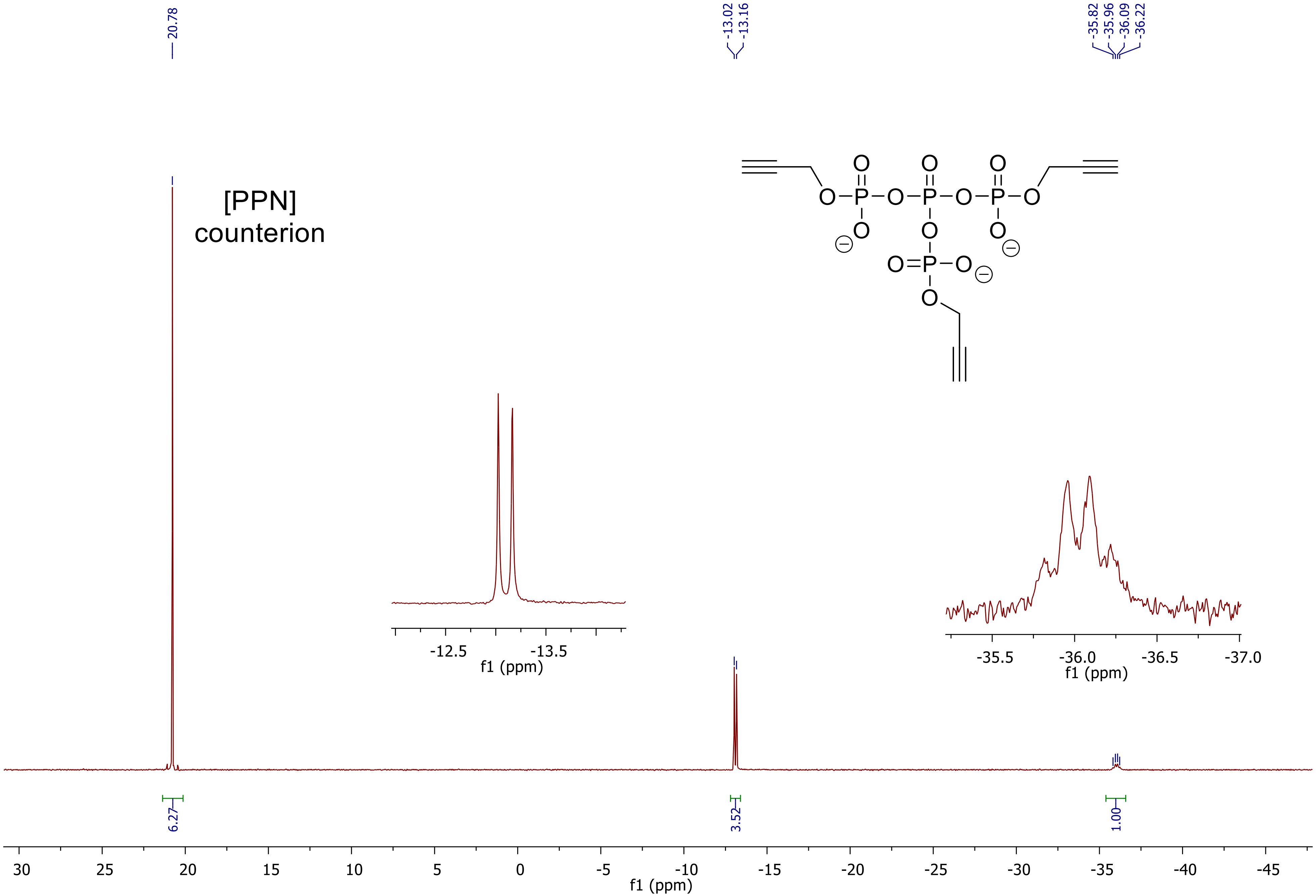

Supplementary Fig. 36| **^{31}P-NMR** (162 MHz, CD_3CN), compound **25**:

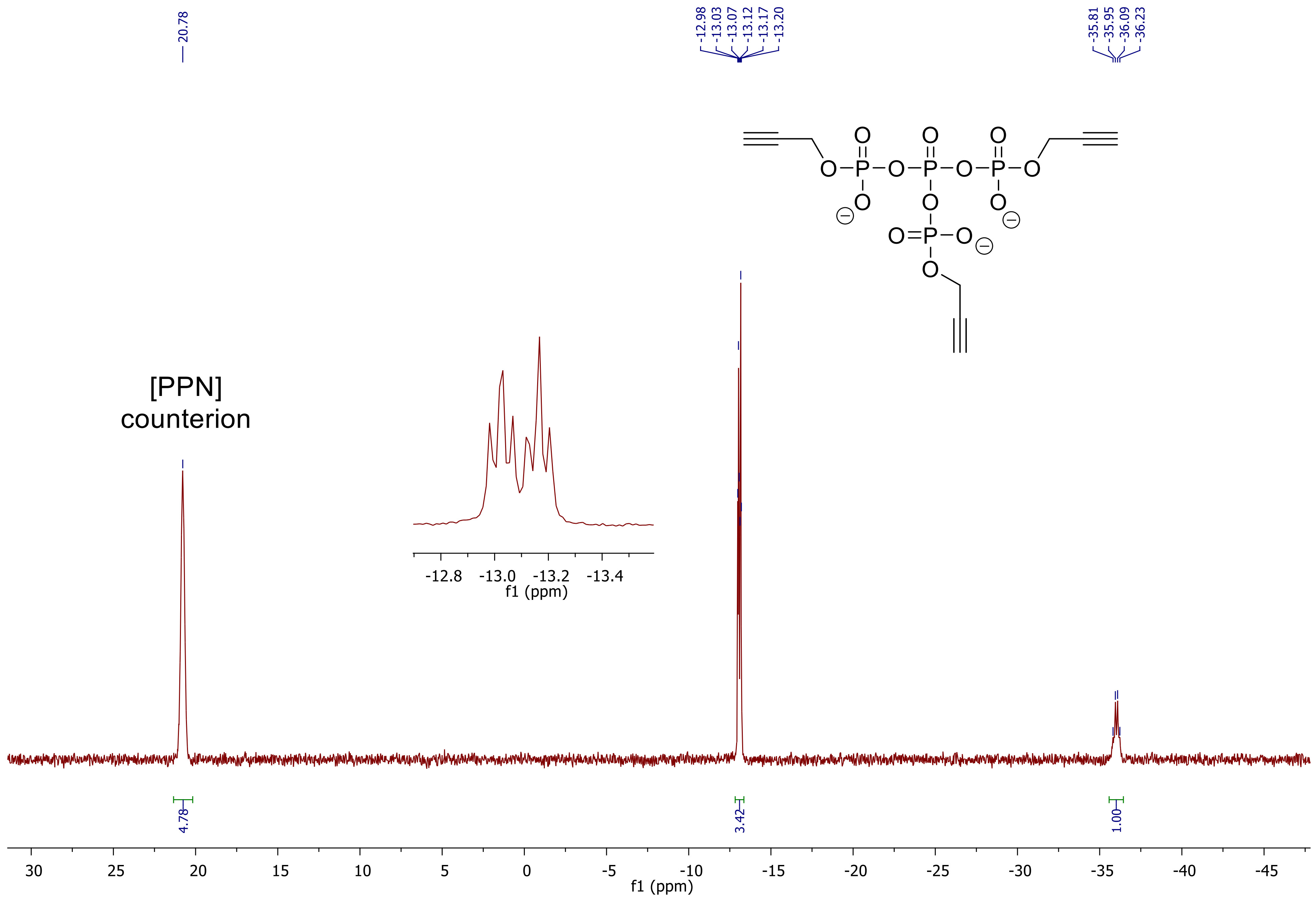

Supplementary Fig. 37 | **^{13}C-NMR** (101 MHz, CD_3CN), compound **25**:

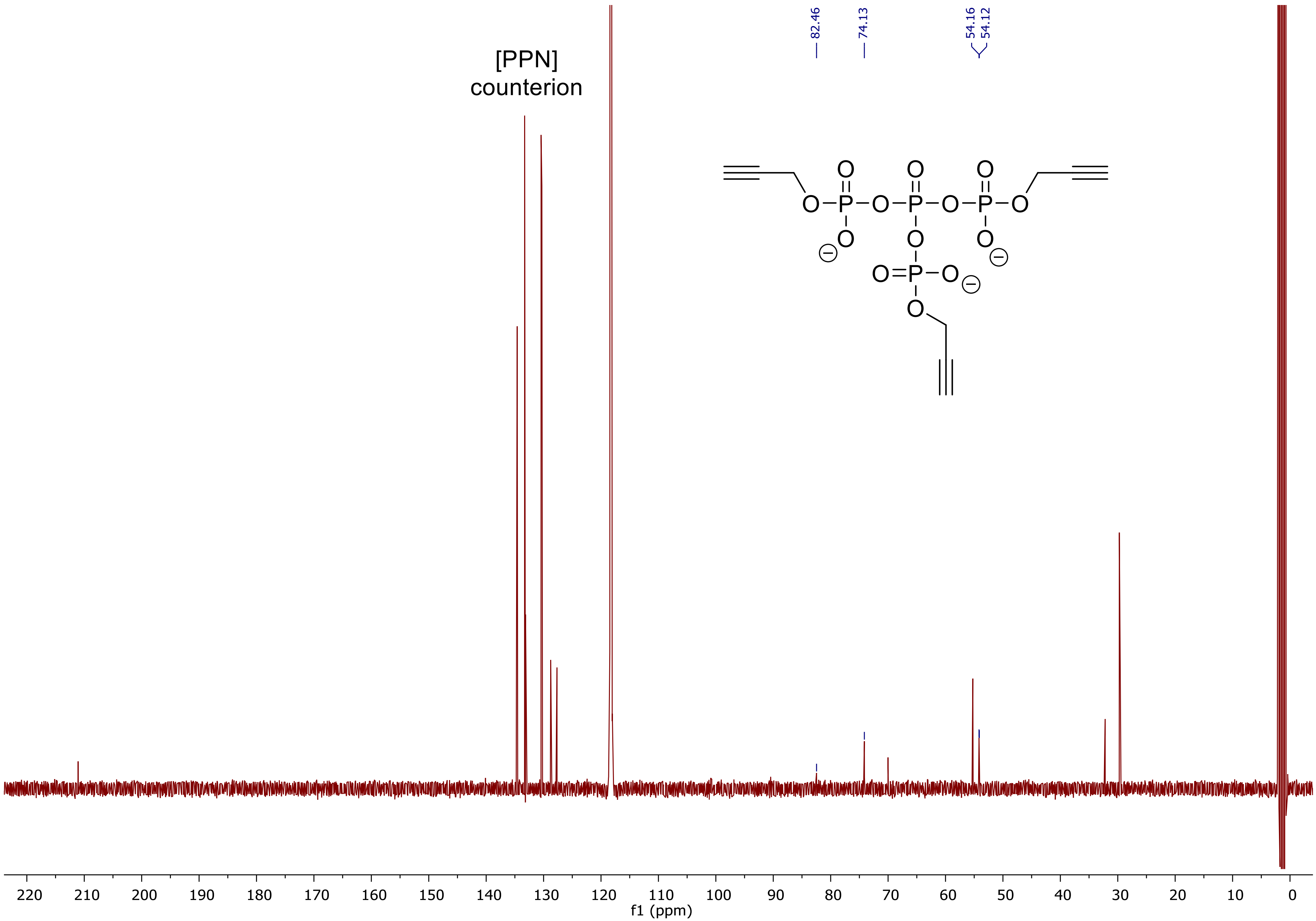

Supplementary Fig. 38| [1]**H-NMR** (300 MHz, DMF-d$_7$), compound **26**:

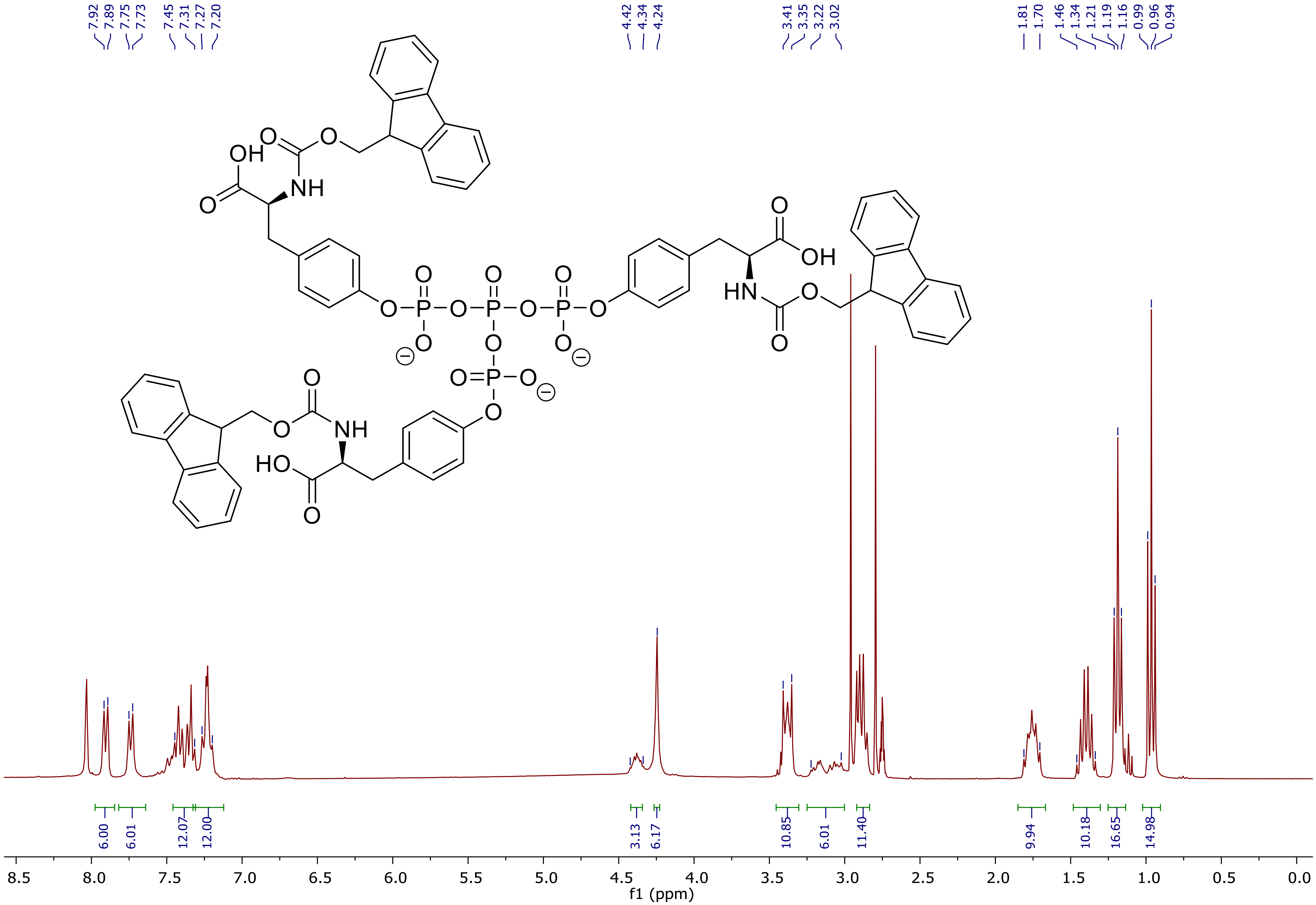

Supplementary Fig. 39 | **$^{31}P\{^{1}H\}$-NMR** (121 MHz, D_2O), compound **26**:

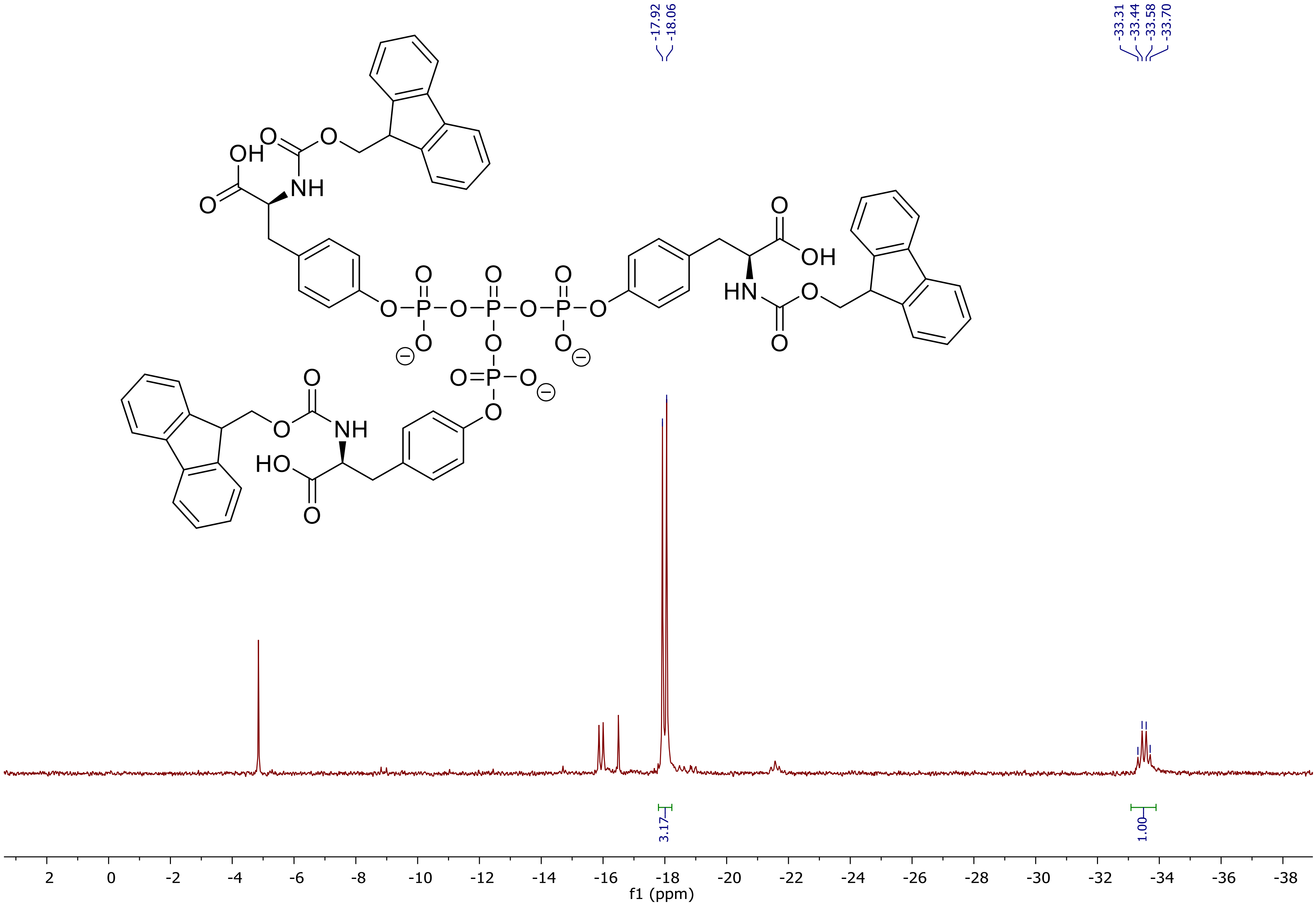

Supplementary Fig. 40 | [1]**H-NMR** (400 MHz, D_2O, presat), compound **27**:

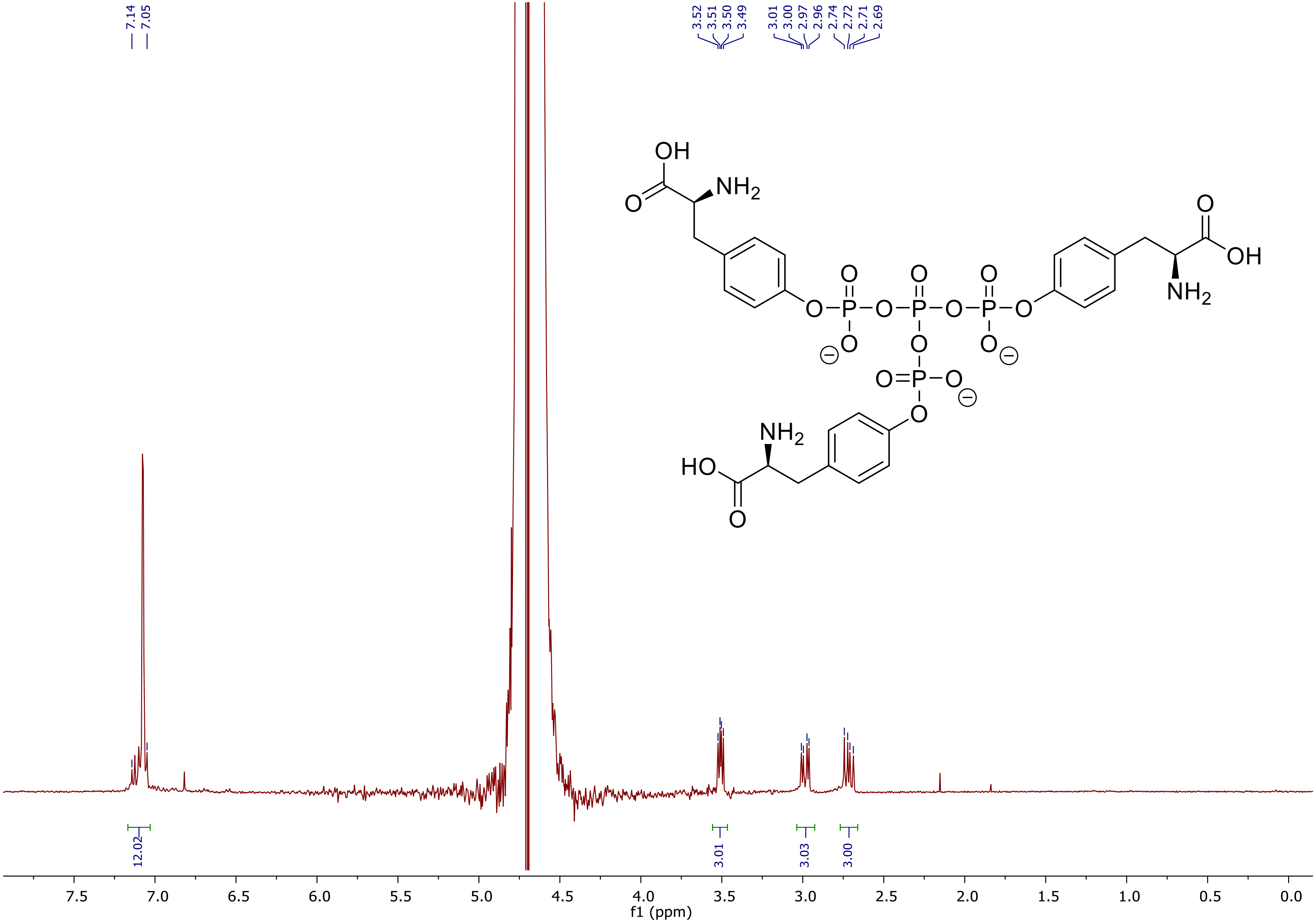

Supplementary Fig. 41| **^{31}P{^{1}H}-NMR** (162 MHz, D_2O), compound **27**:

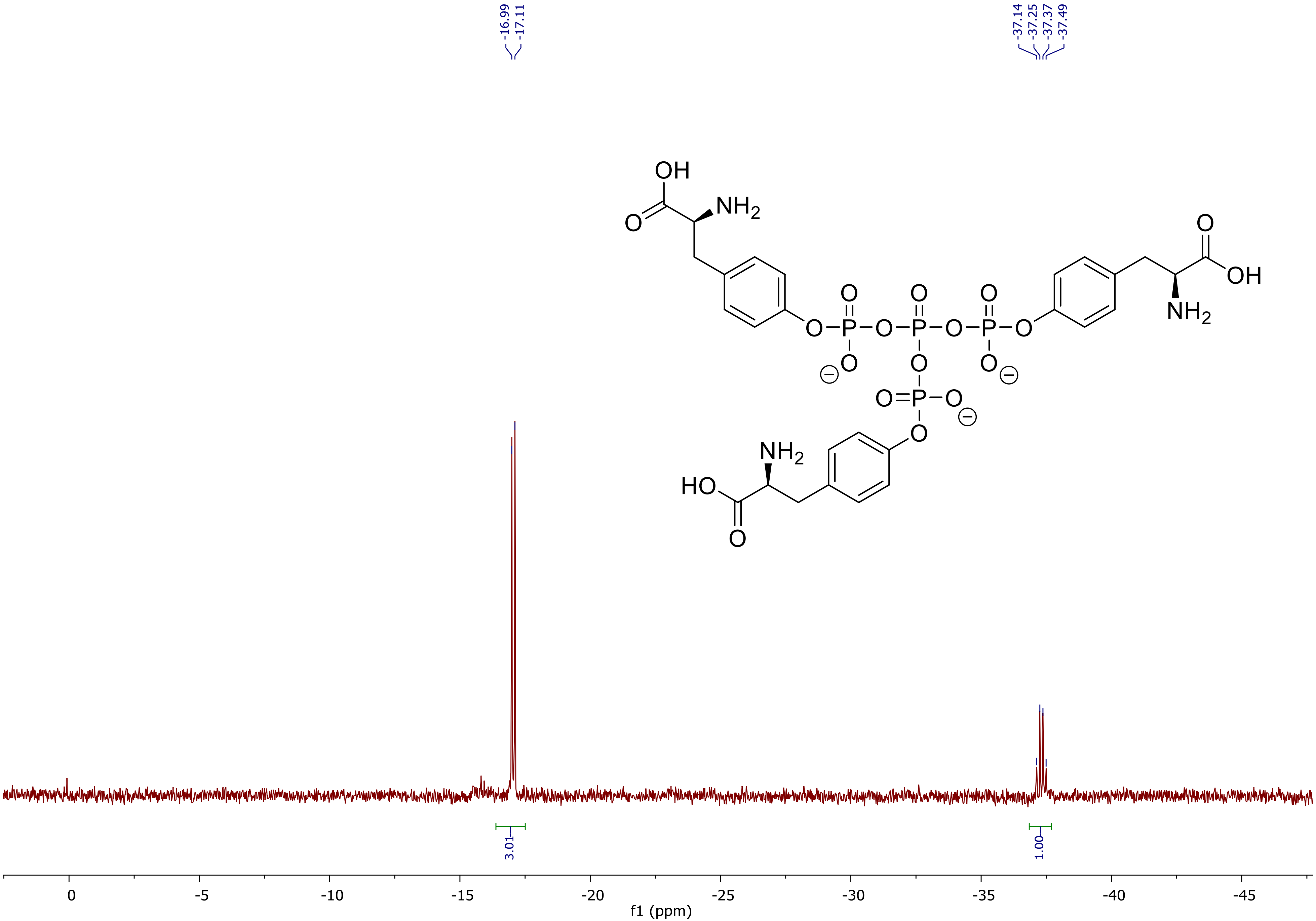

Supplementary Fig. 42| [1]**H-NMR** (400 MHz, D_2O, presat), compound **28**:

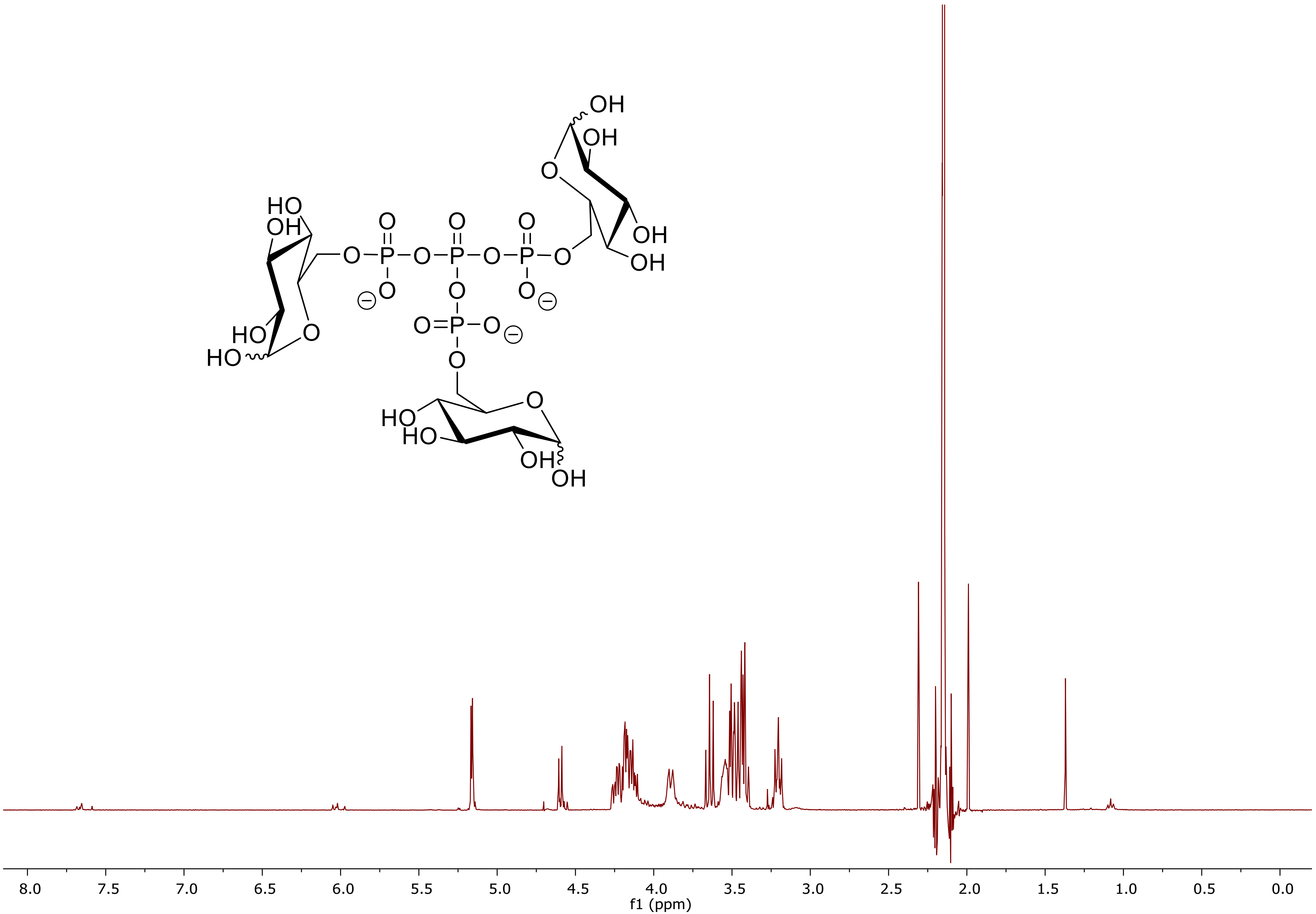

Supplementary Fig. 43 | $^{31}P\{^{1}H\}$-NMR (162 MHz, D_2O), compound **28**:

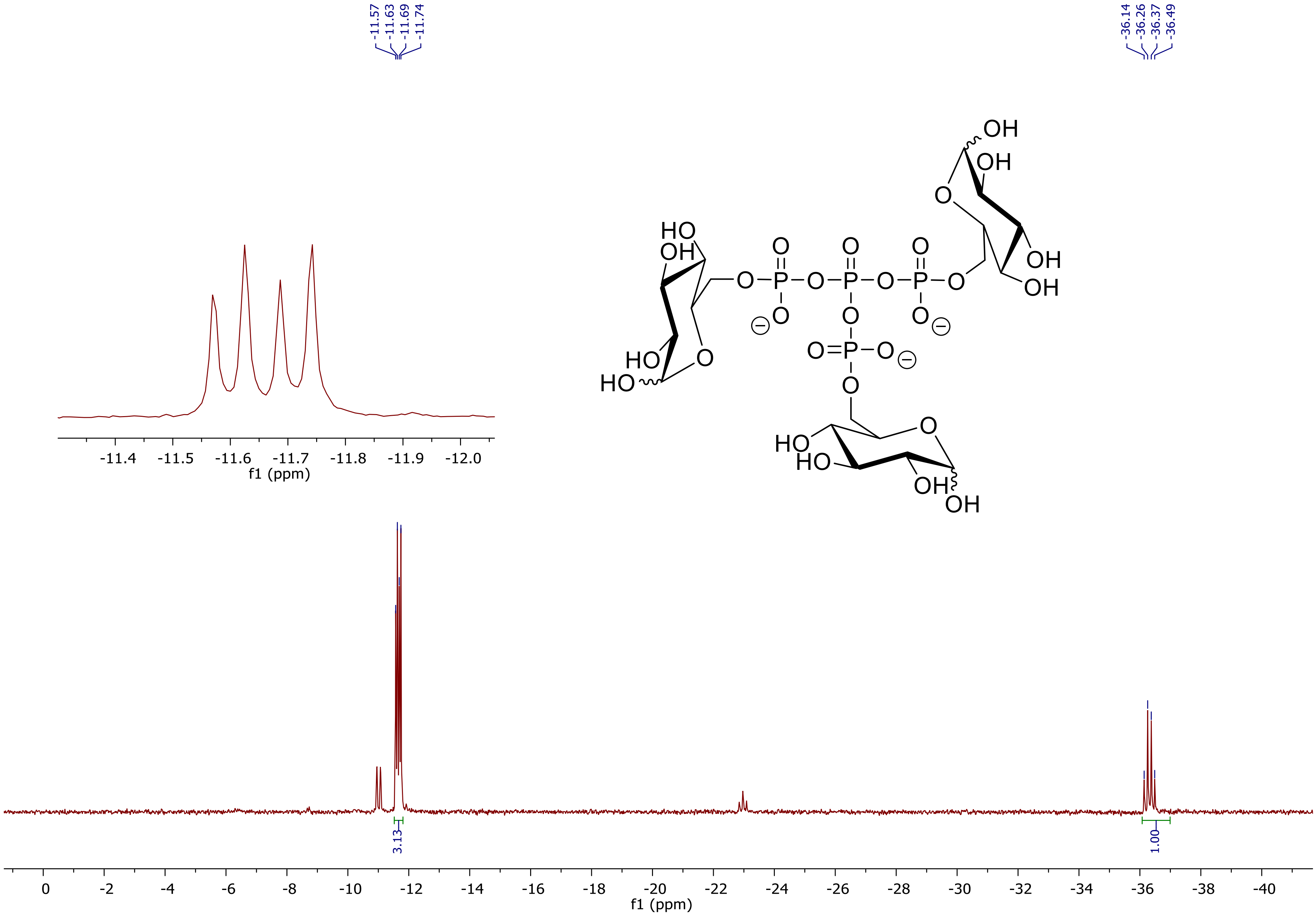

Supplementary Fig. 44| **^{31}P-NMR** (162 MHz, D_2O), compound **28**:

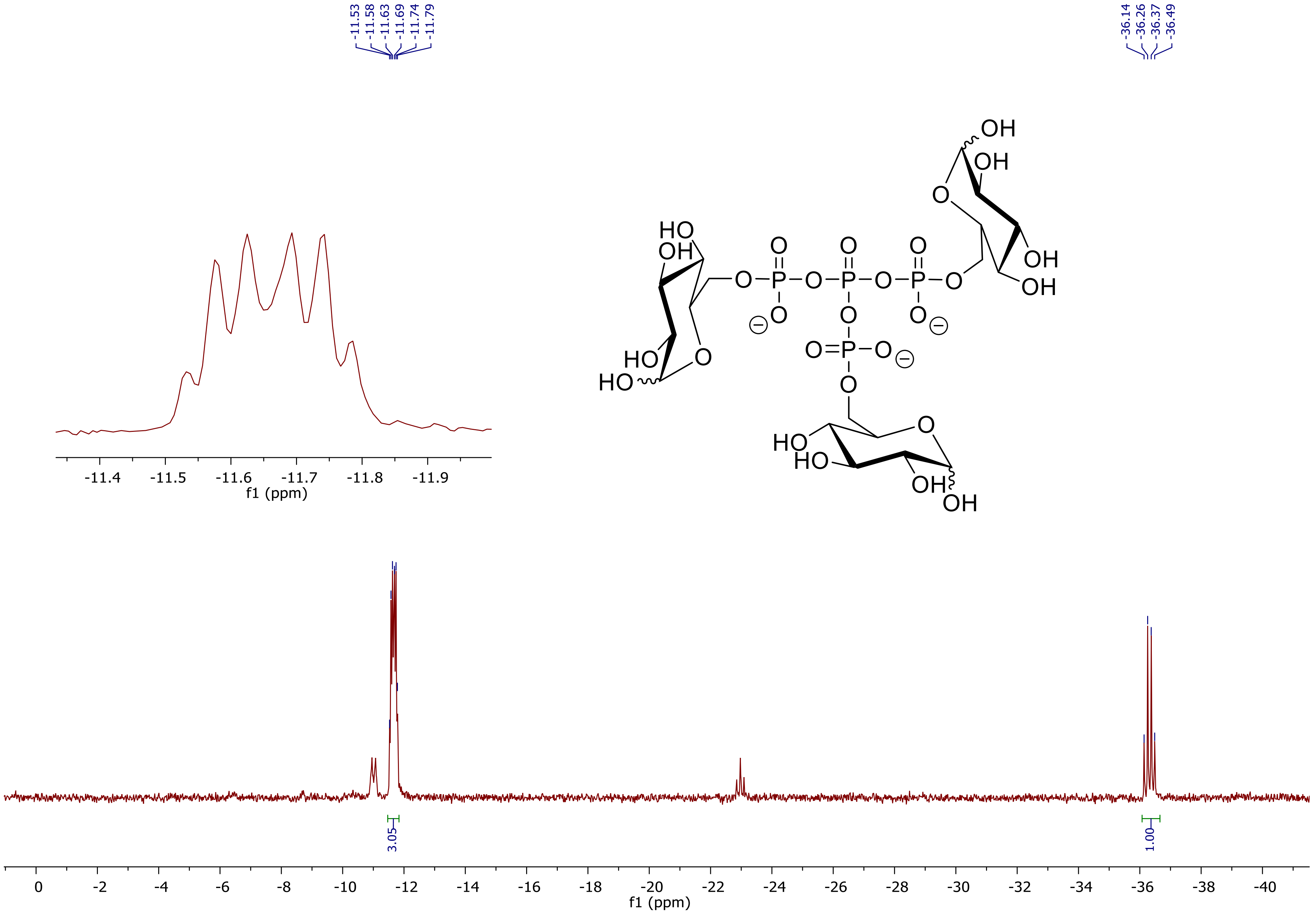

Supplementary Fig. 45 | [1]**H-NMR** (500 MHz, CD_3CN), compound **29**:

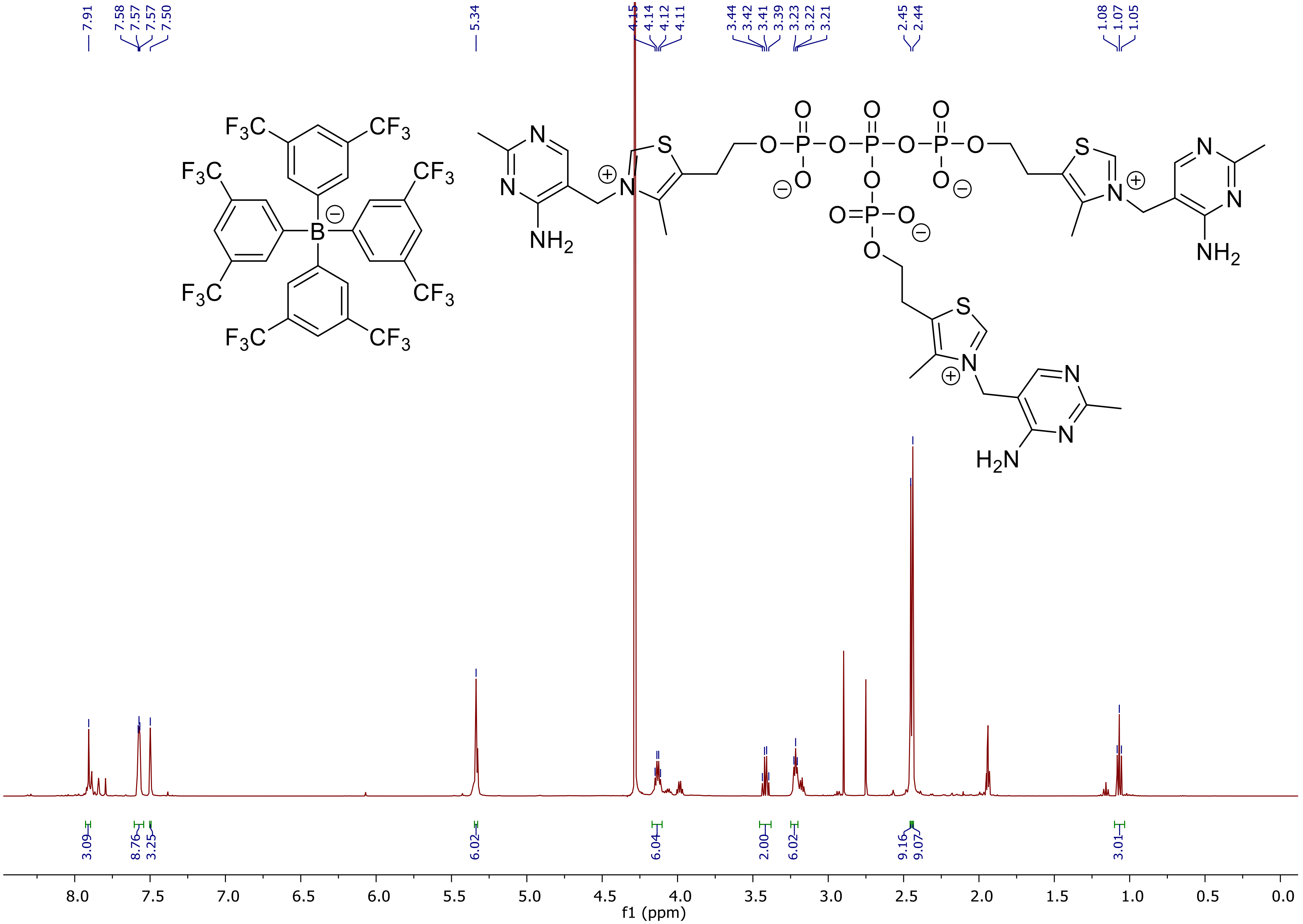

Supplementary Fig. 46| **$^{31}P\{^1H\}$-NMR** (202 MHz, CD_3CN), compound **29**:

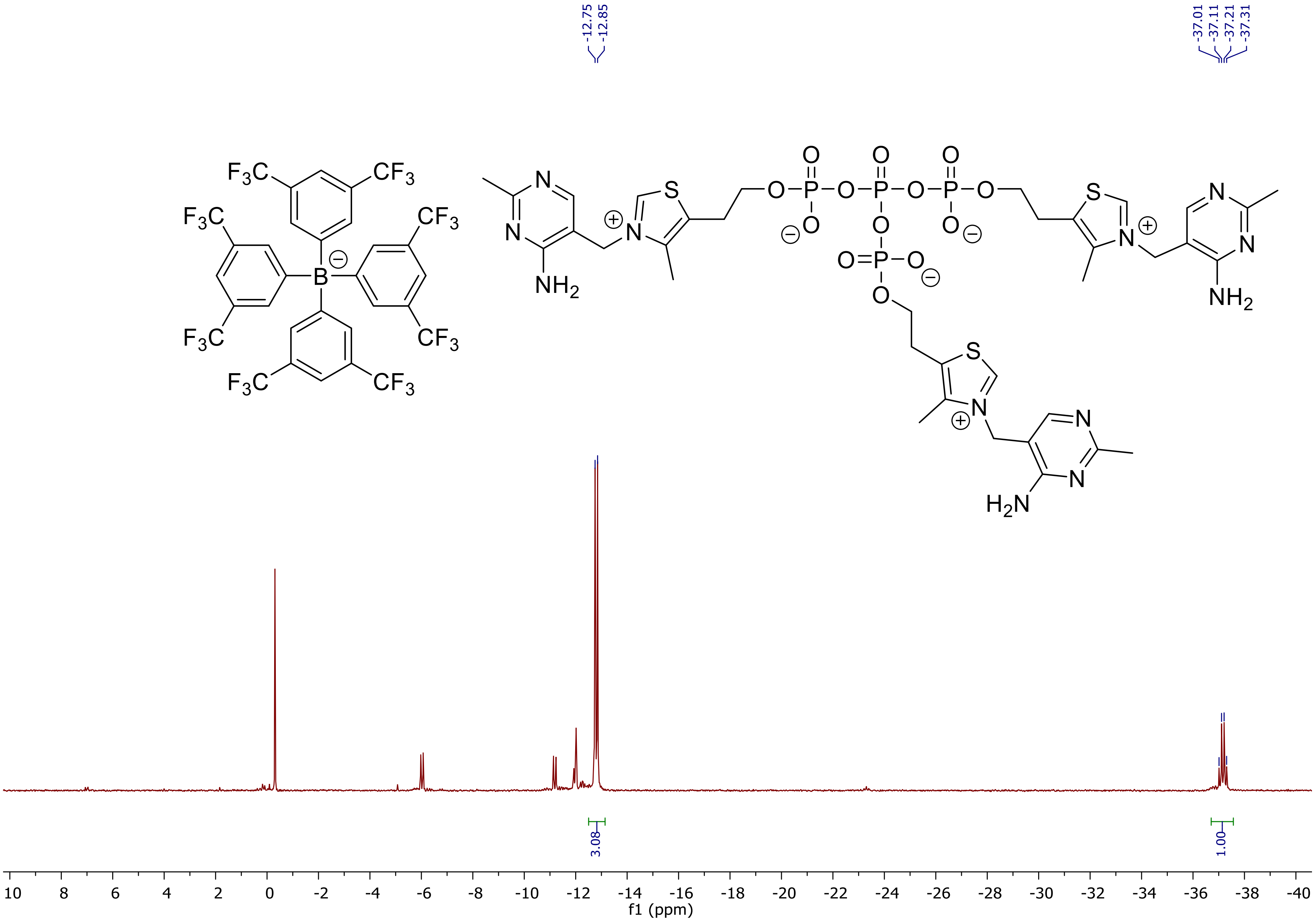

Supplementary Fig. 47 | **^{31}P-NMR** (202 MHz, CD_3CN), compound **29**:

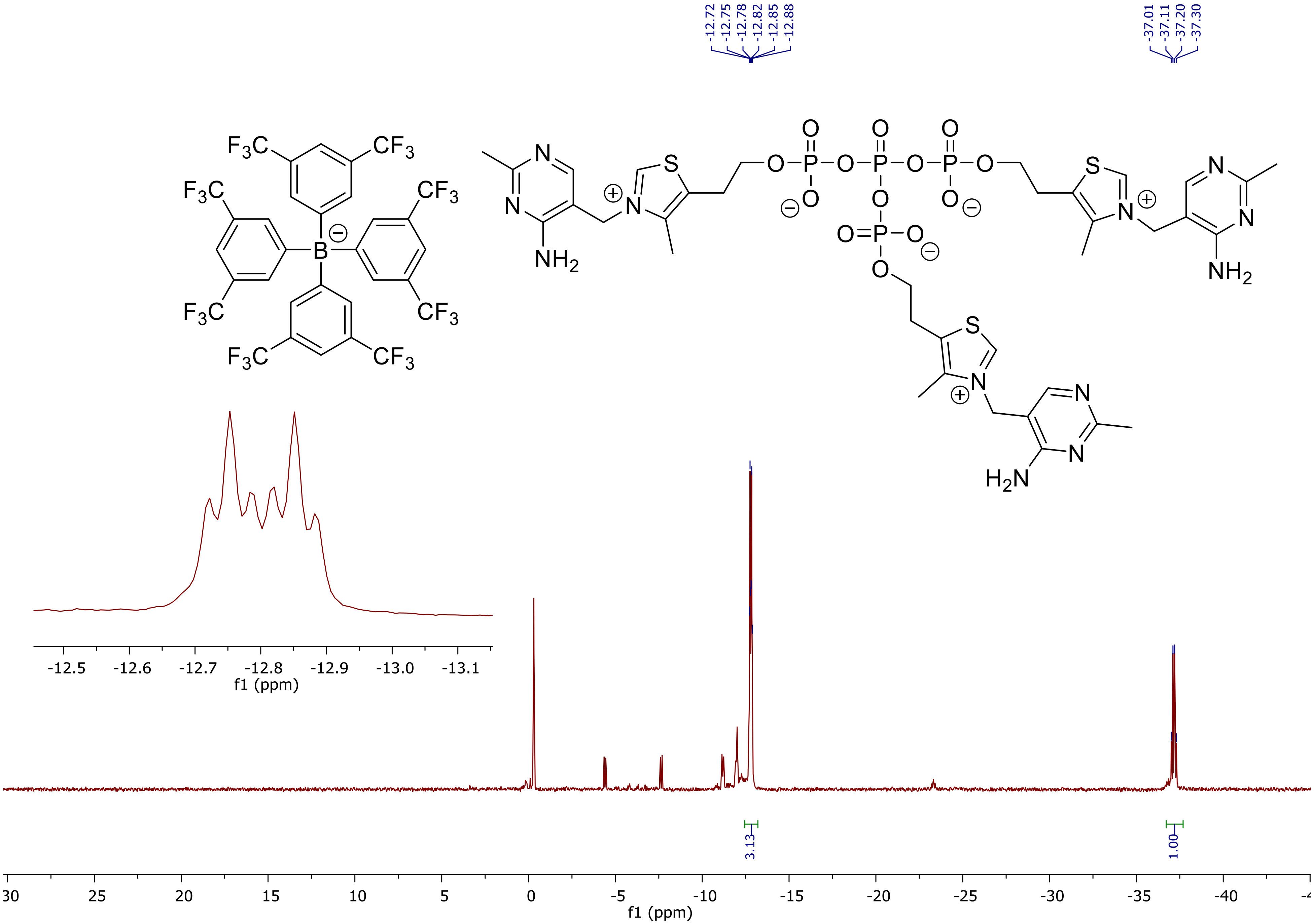

Supplementary Fig. 48| [1]**H-NMR** (400 MHz, CD_3CN), compound **30**:

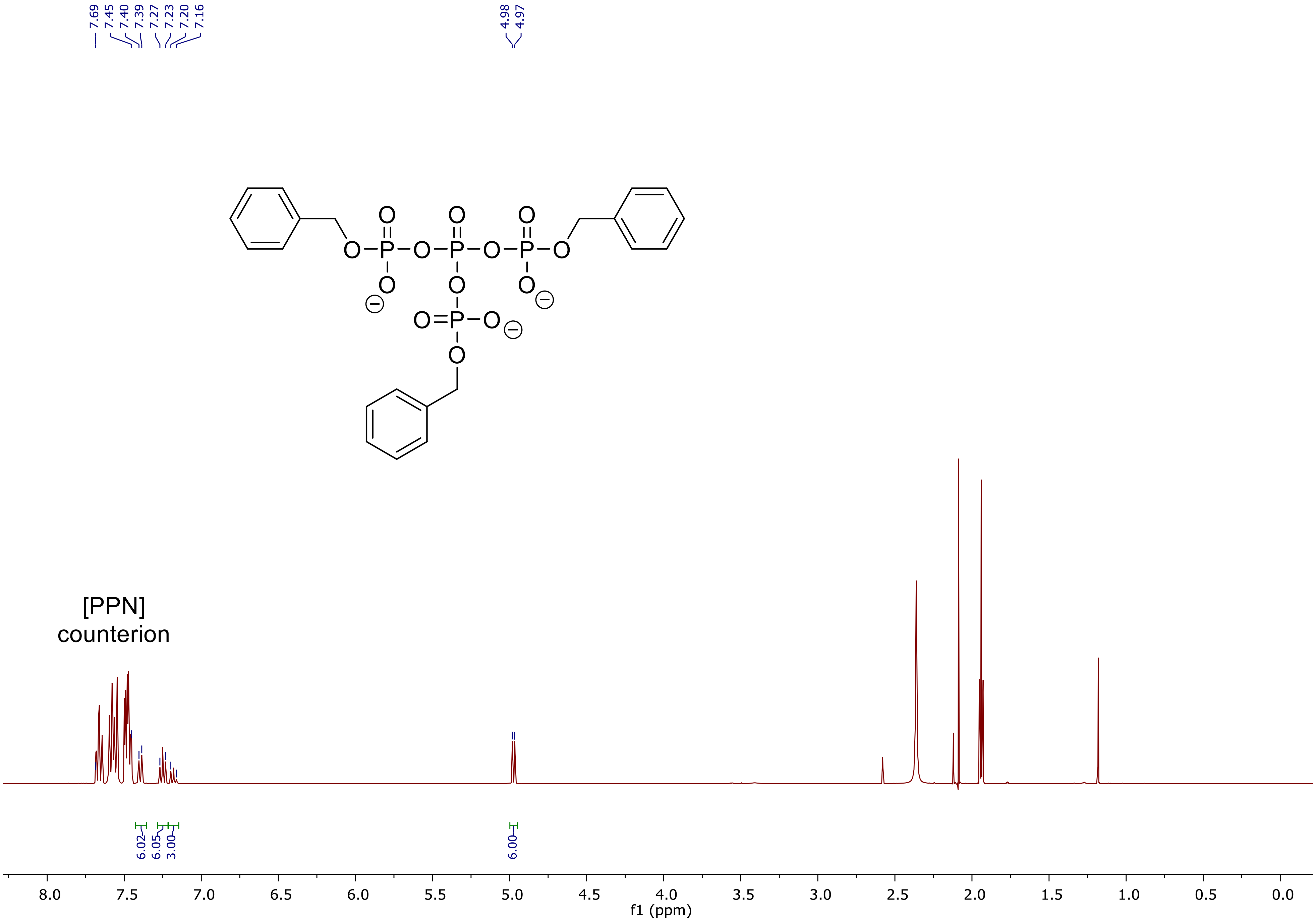

Supplementary Fig. 49| **^{31}P{^{1}H}-NMR** (162 MHz, CD_3CN), compound **30**:

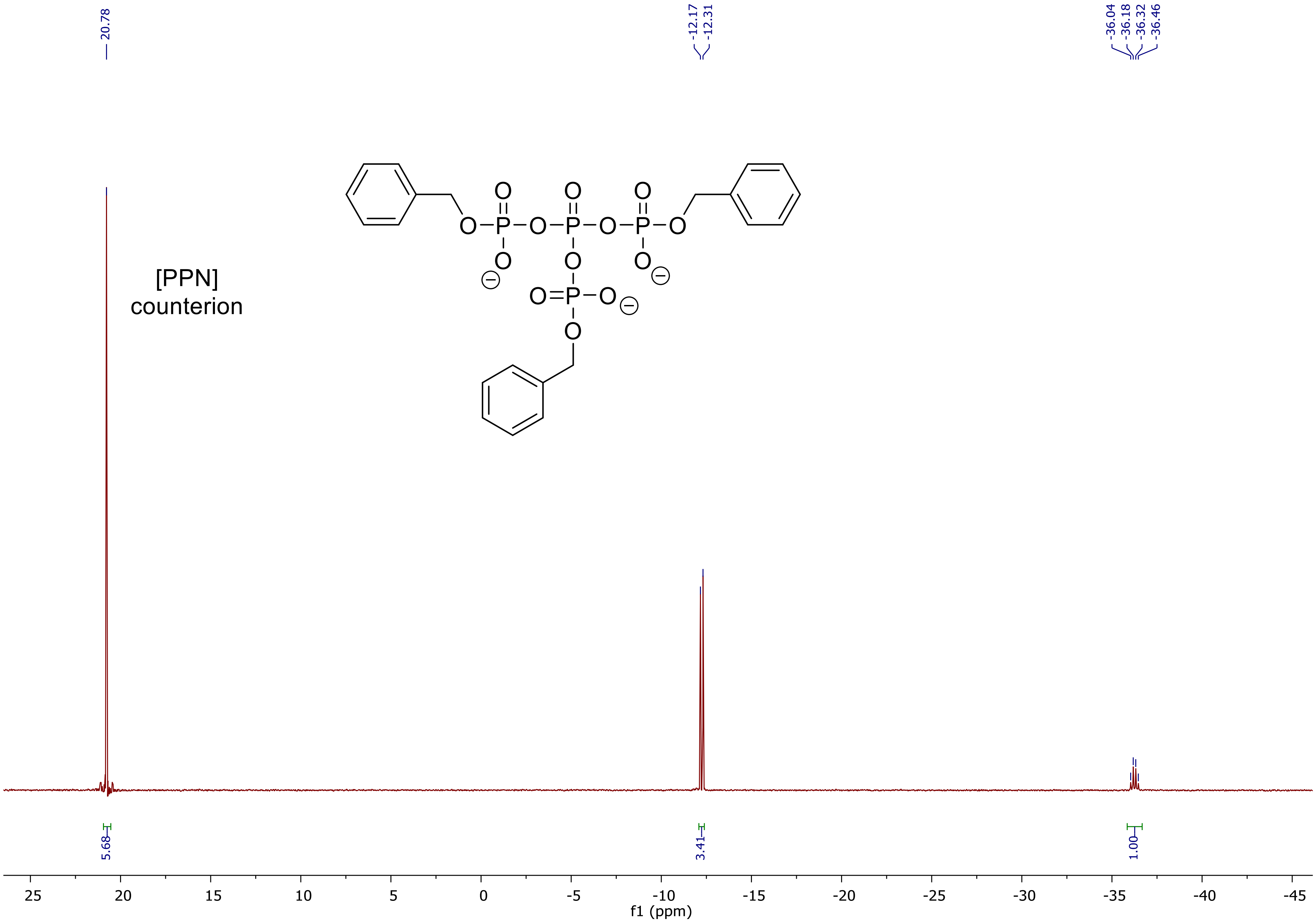

Supplementary Fig. 50| ^{31}**P-NMR** (162 MHz, CD_3CN), compound **30**:

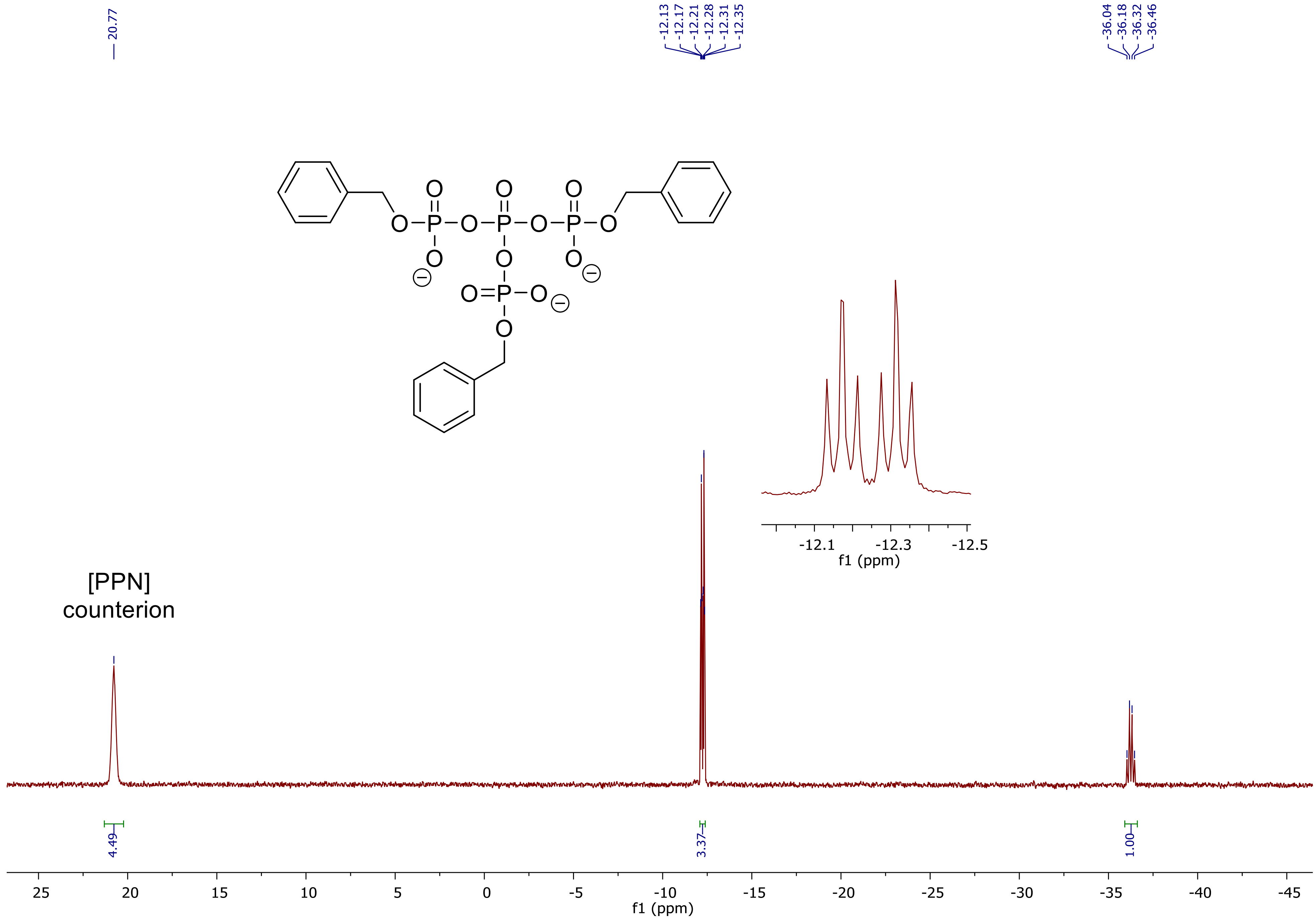

Supplementary Fig. 51│ **^{13}C-NMR** (101 MHz, CD_3CN), compound **30**:

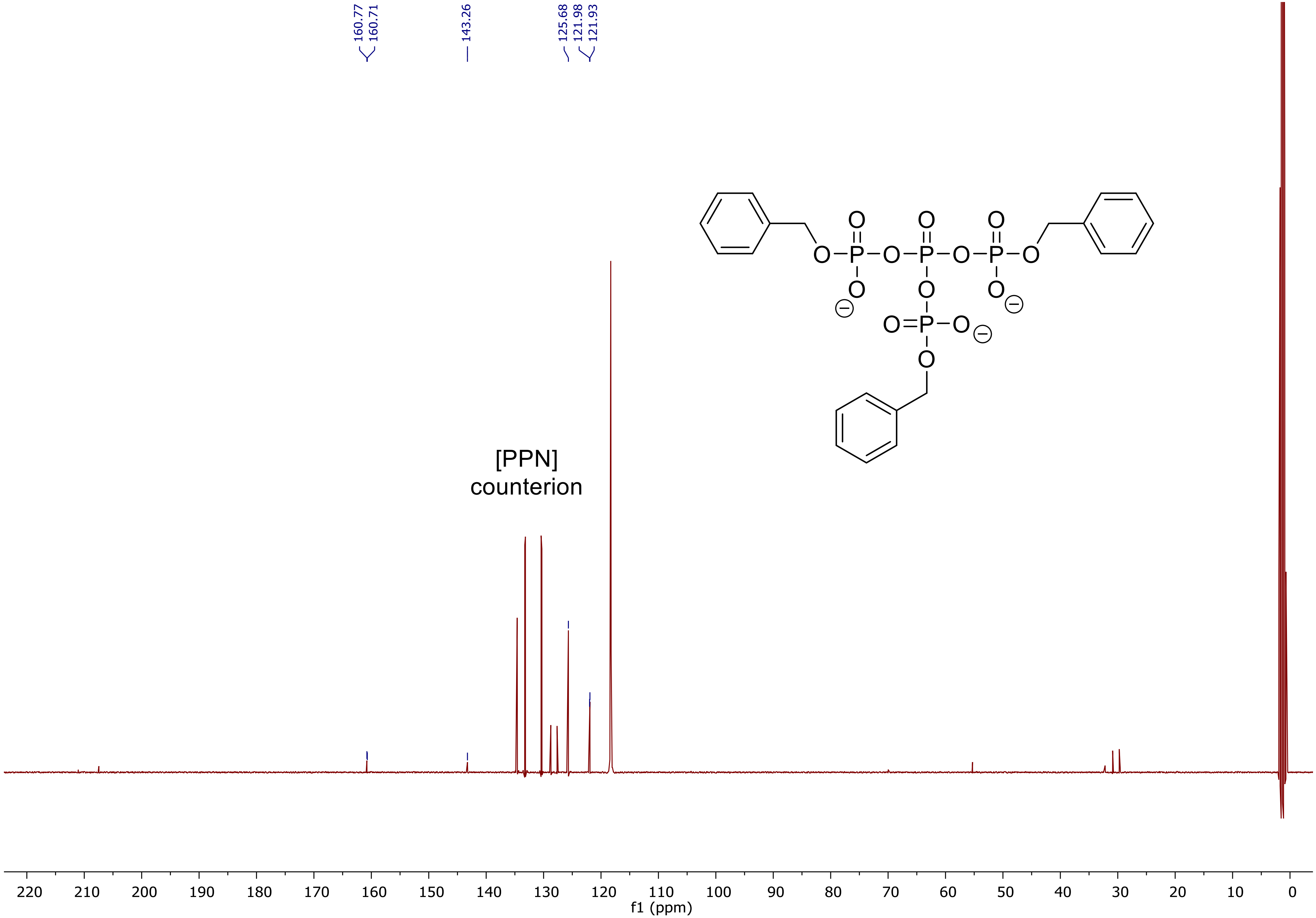

Supplementary Fig. 52 | **¹H-NMR** (400 MHz, CD_3CN), compound **31**:

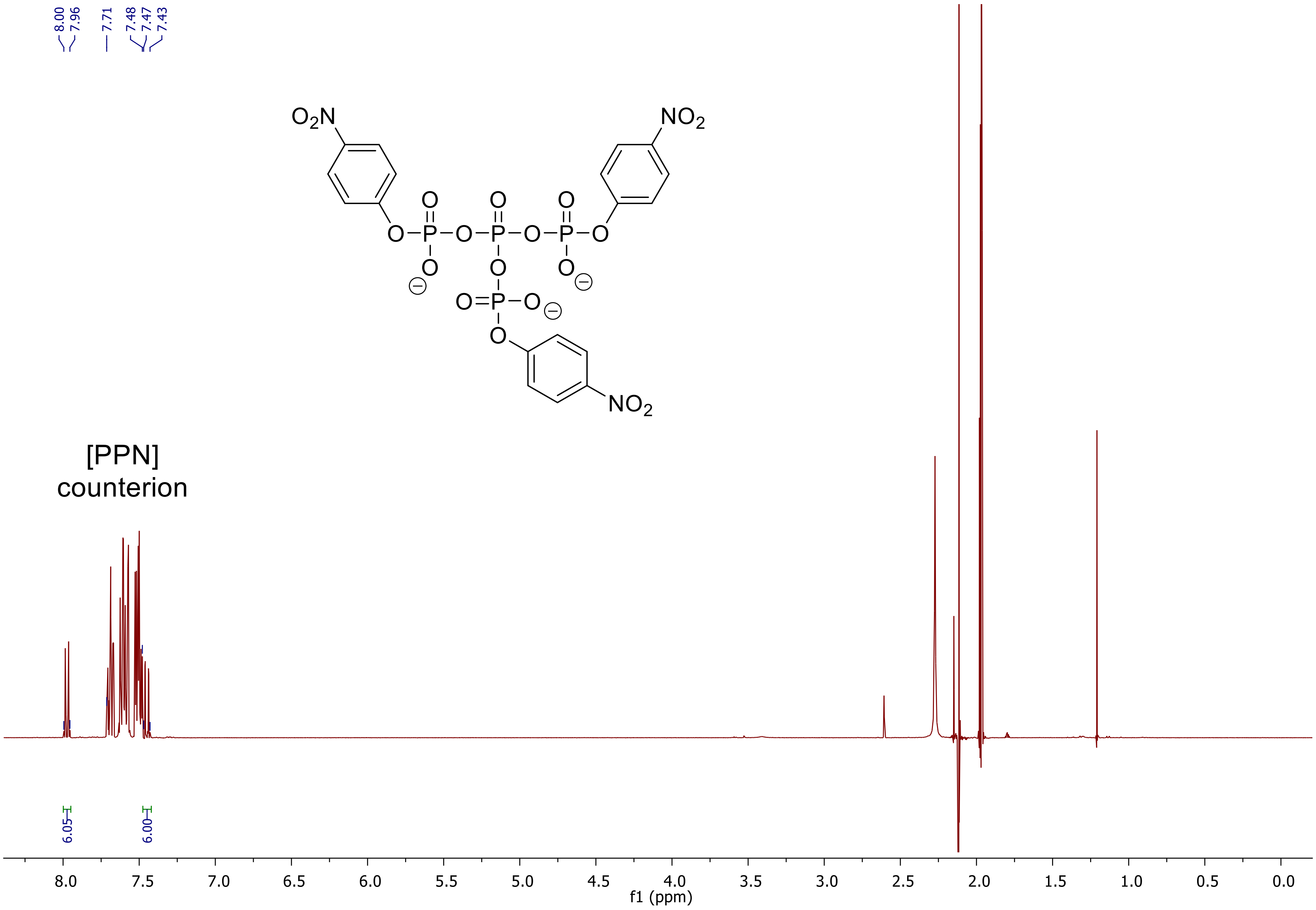

Supplementary Fig. 53 | **^{31}P{^{1}H}-NMR** (162 MHz, CD_3CN), compound **31**:

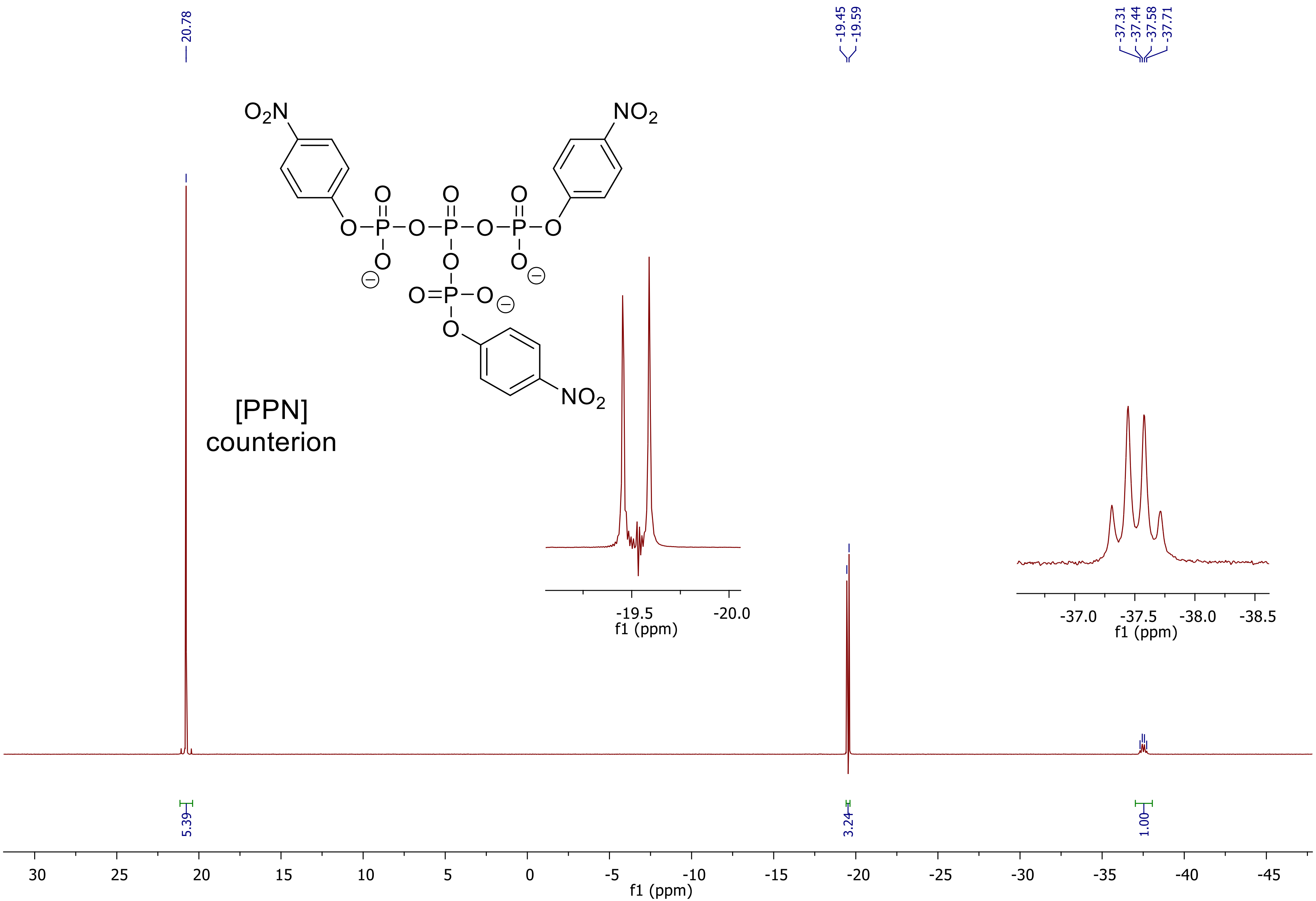

Supplementary Fig. 54│ **^{13}C-NMR** (101 MHz, CD_3CN), compound **31**:

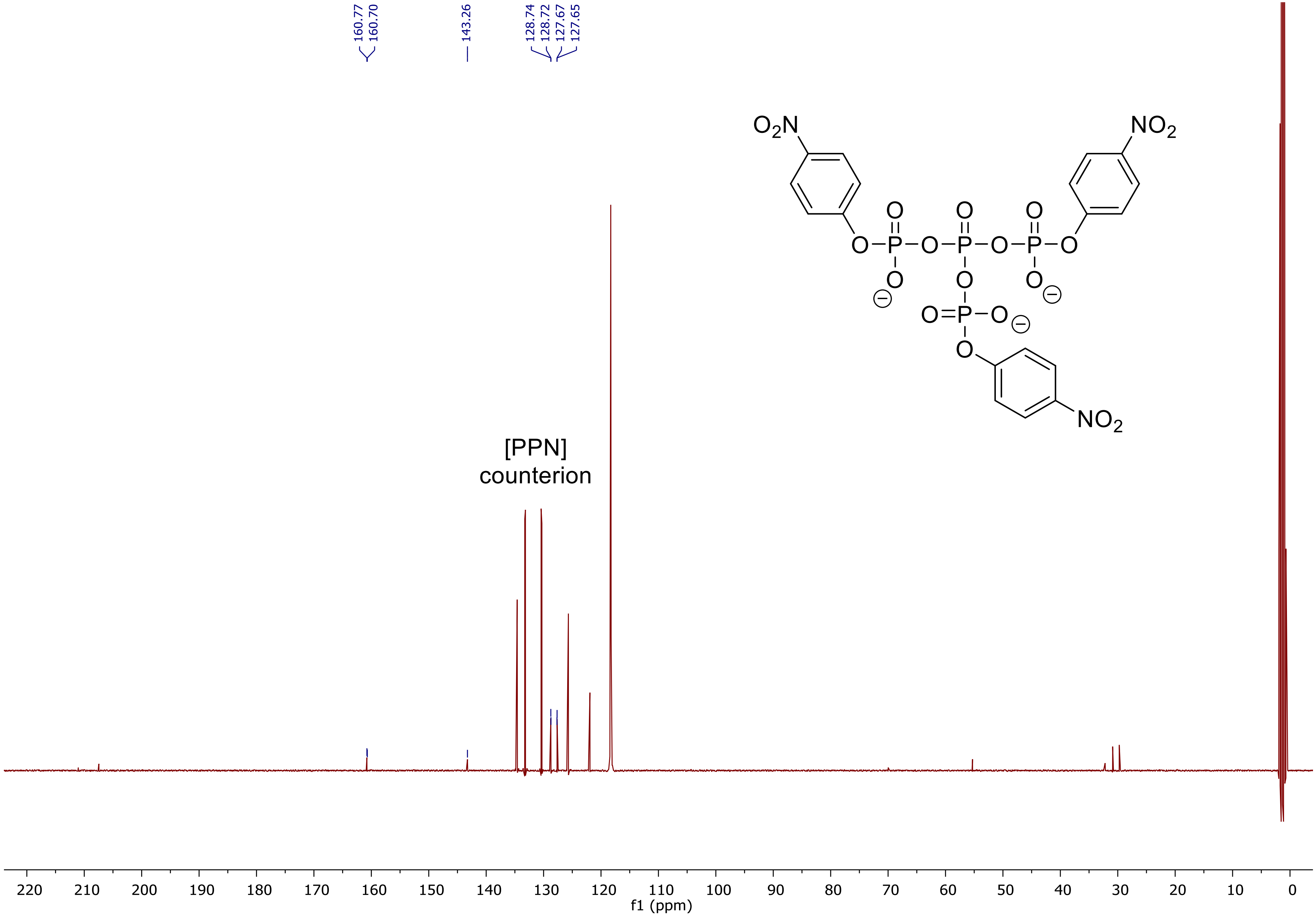

Supplementary Fig. 55 | **^{1}H-NMR** (400 MHz, D_2O, presat), compound **64**:

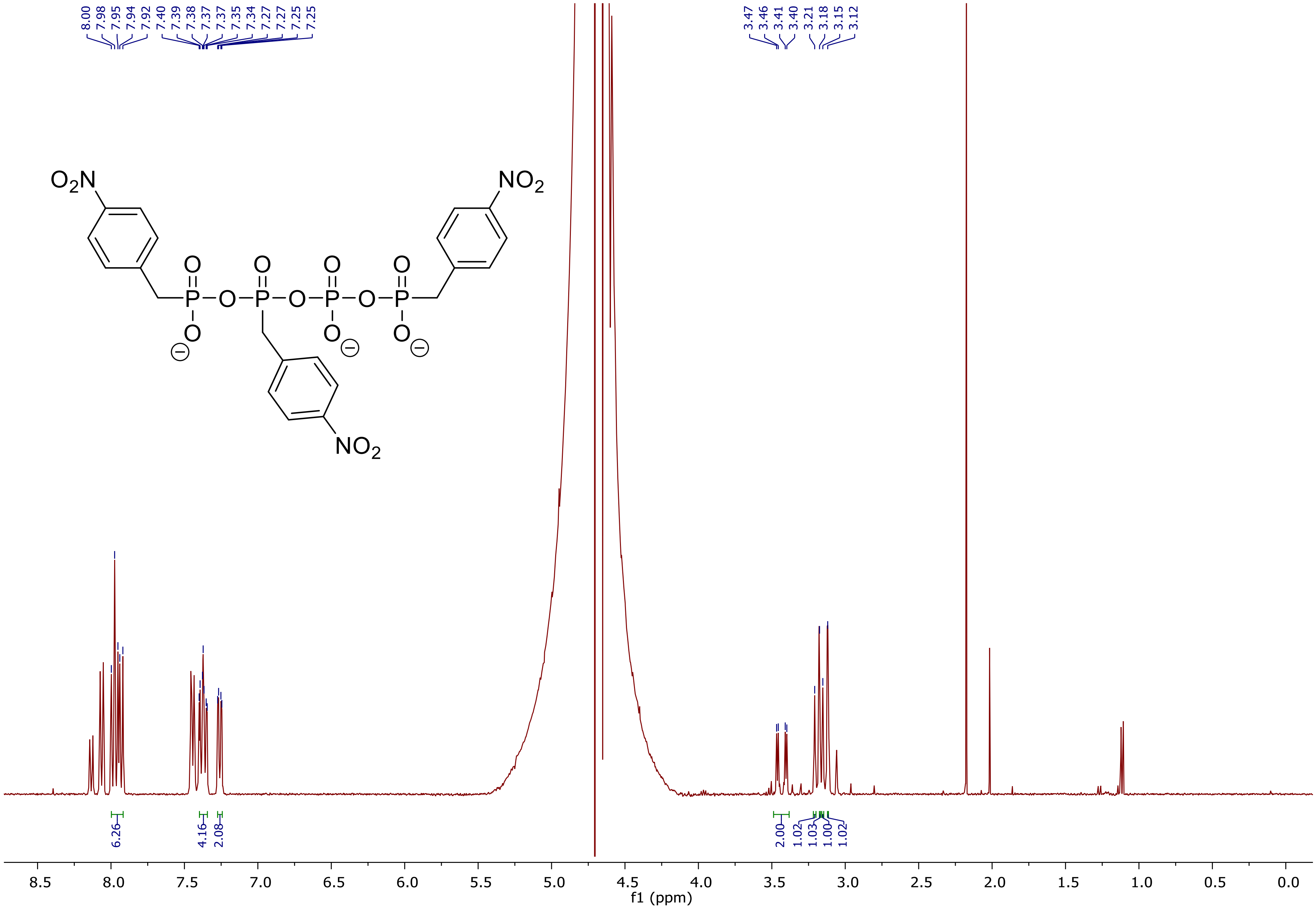

Supplementary Fig. 56 | **^{31}P{^{1}H}-NMR** (162 MHz, D_2O), compound **64**:

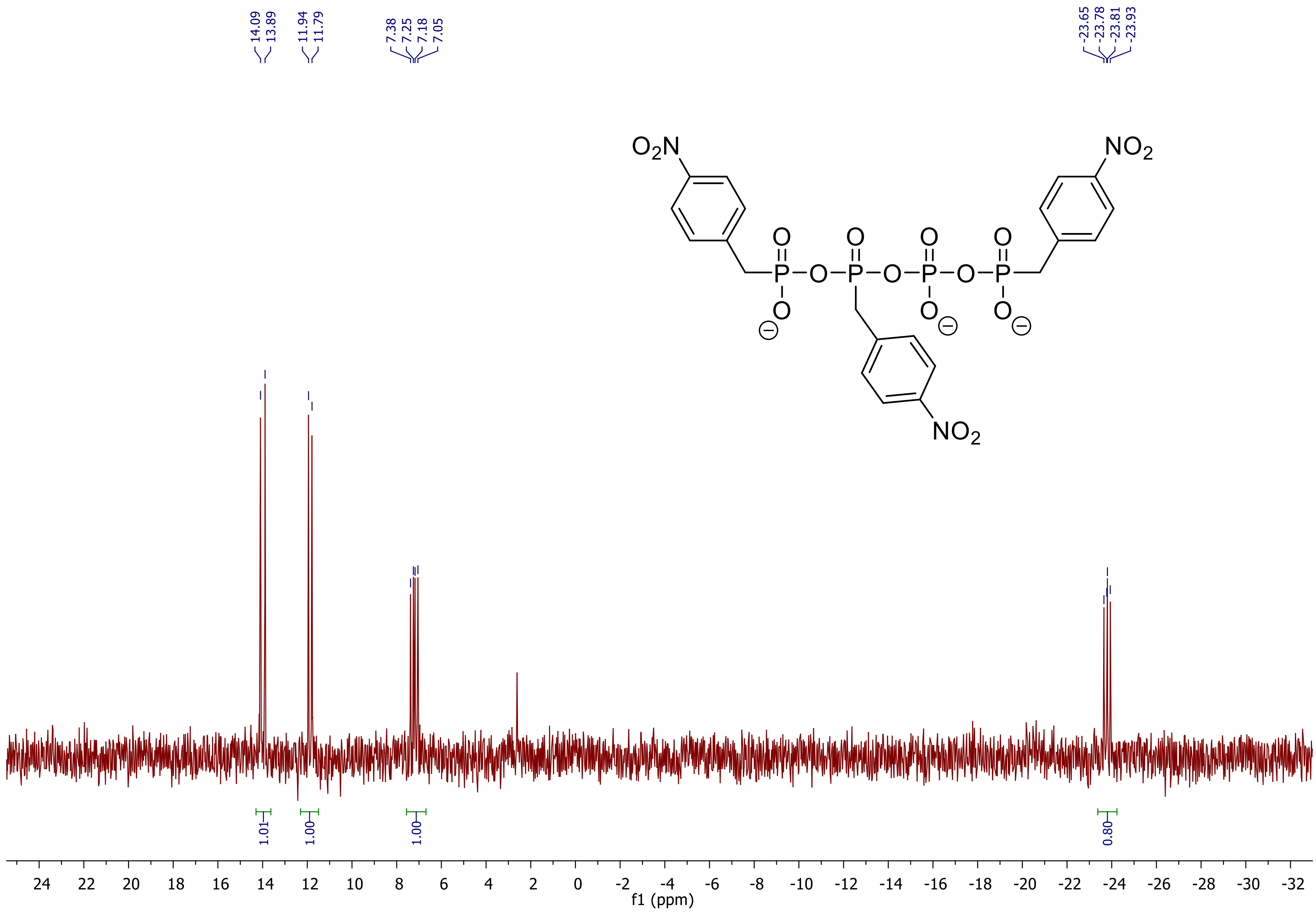

Supplementary Fig. 57| **^{31}P-NMR** (162 MHz, D_2O), compound **64**:

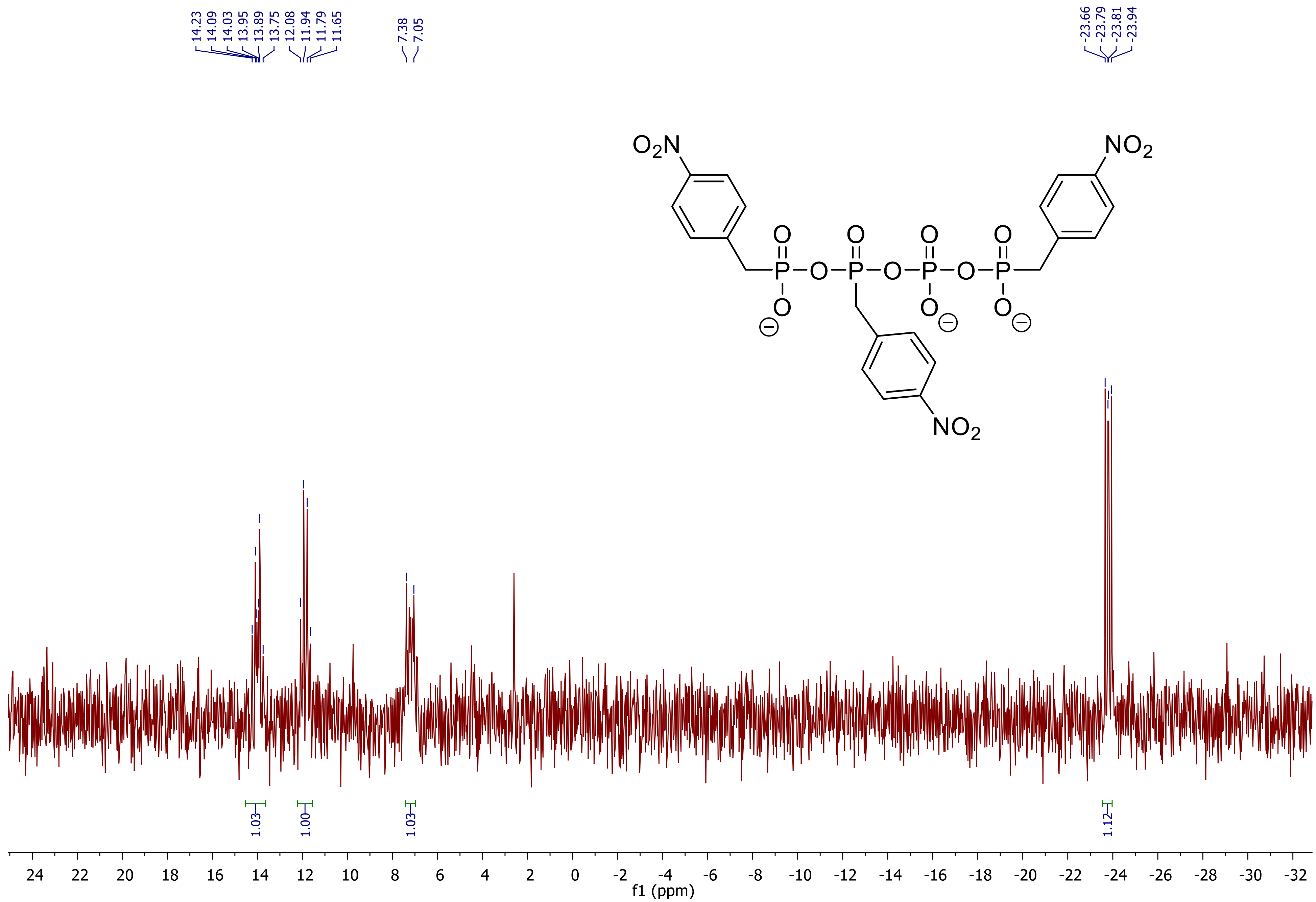

Supplementary Fig. 58| [1]**H-NMR** (400 MHz, D_2O, presat), compound **32**:

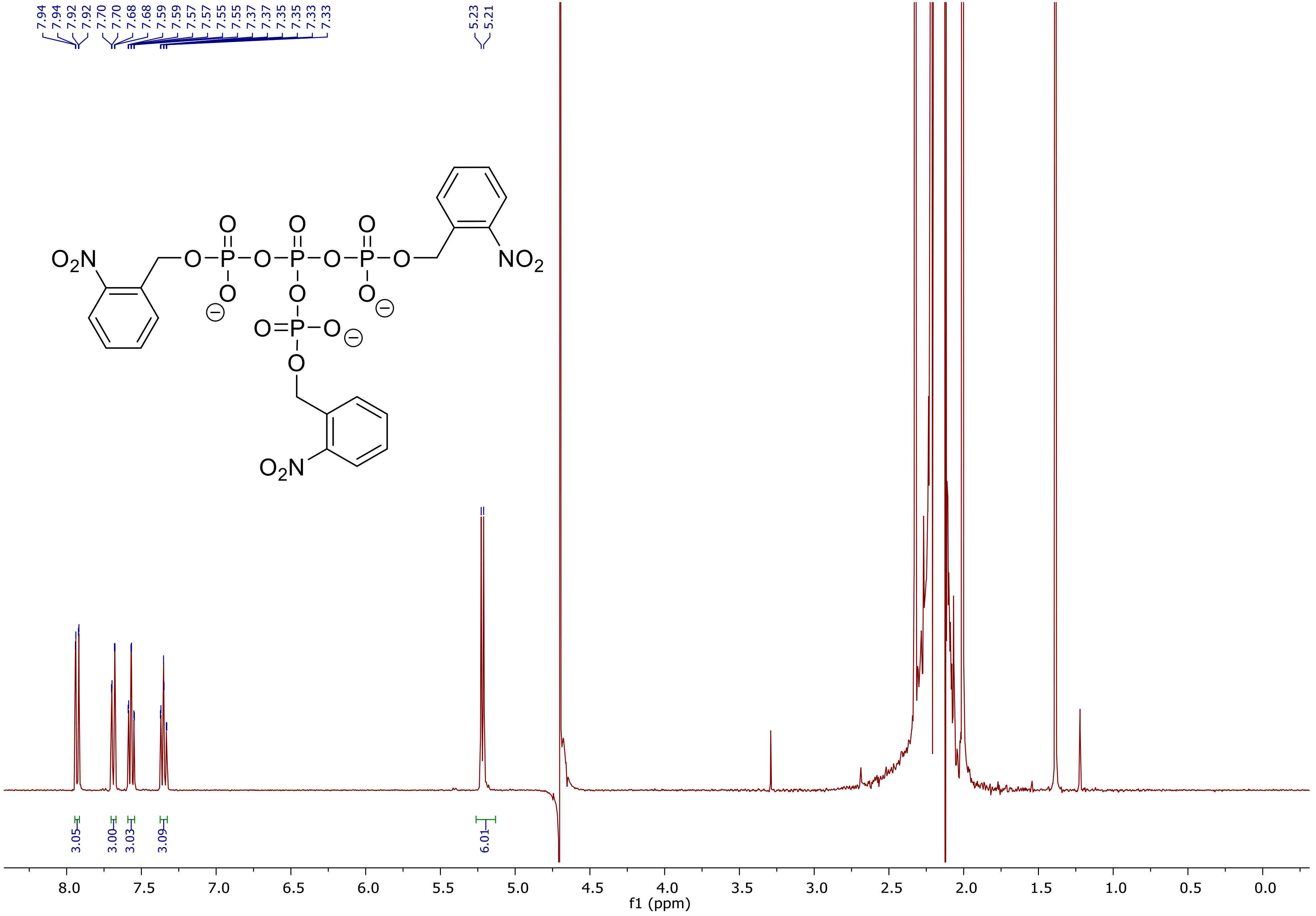

Supplementary Fig. 59| **^{31}P{^{1}H}-NMR** (162 MHz, D_2O), compound **32**:

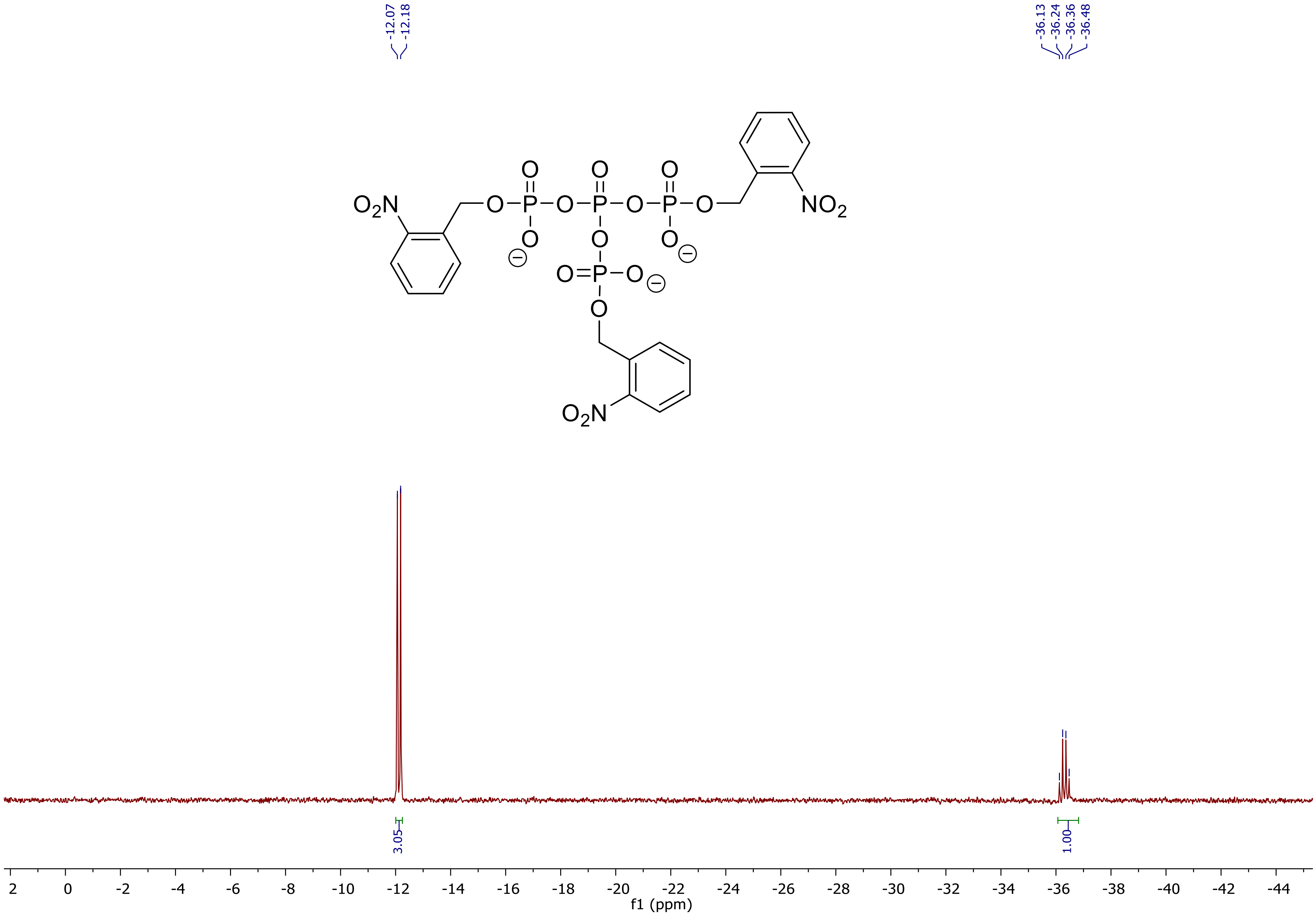

Supplementary Fig. 60| ^{31}**P-NMR** (162 MHz, D_2O), compound **32**:

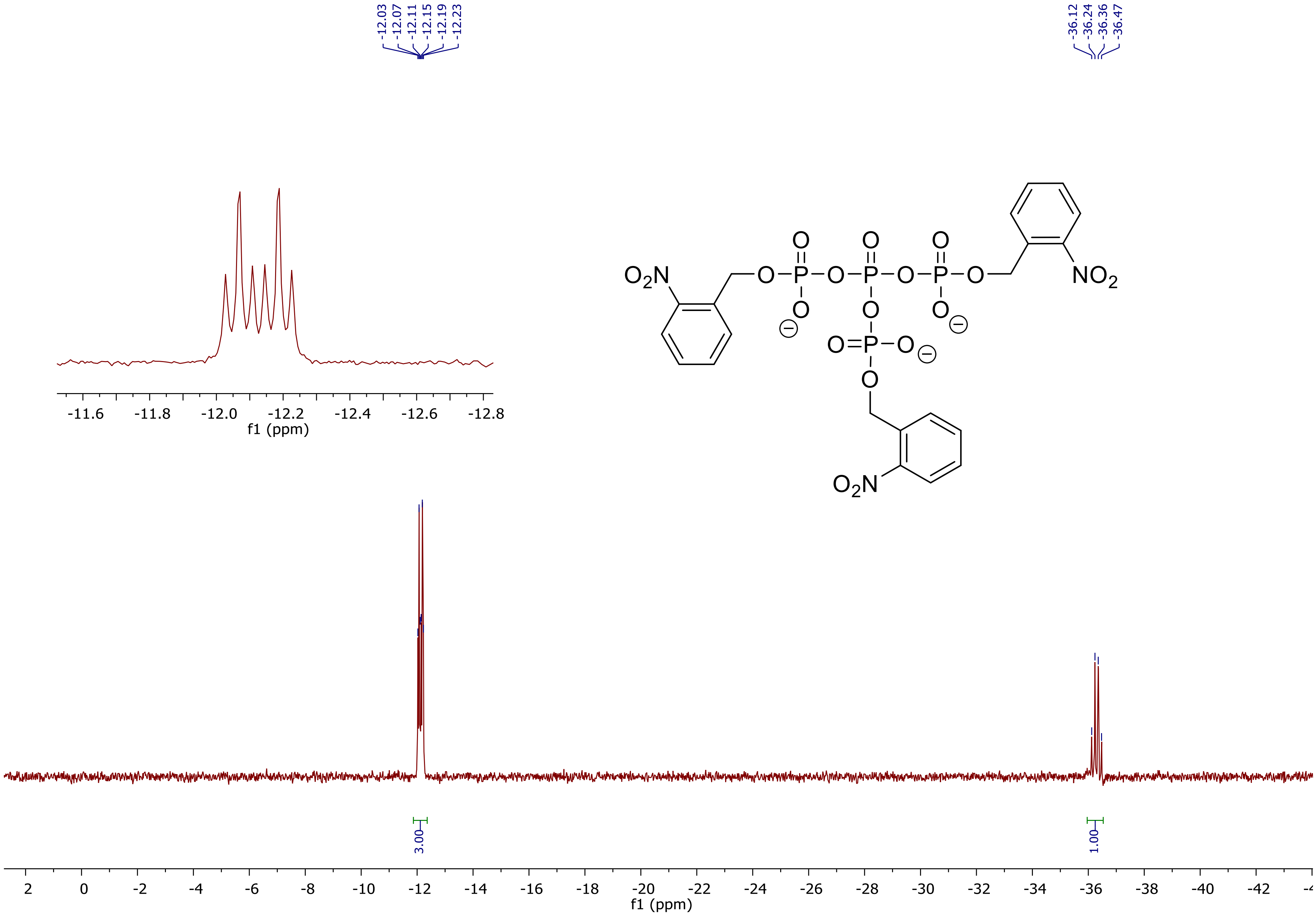

Supplementary Fig. 61│ **^{1}H-NMR** (400 MHz, D_2O, presat), compound **33**:

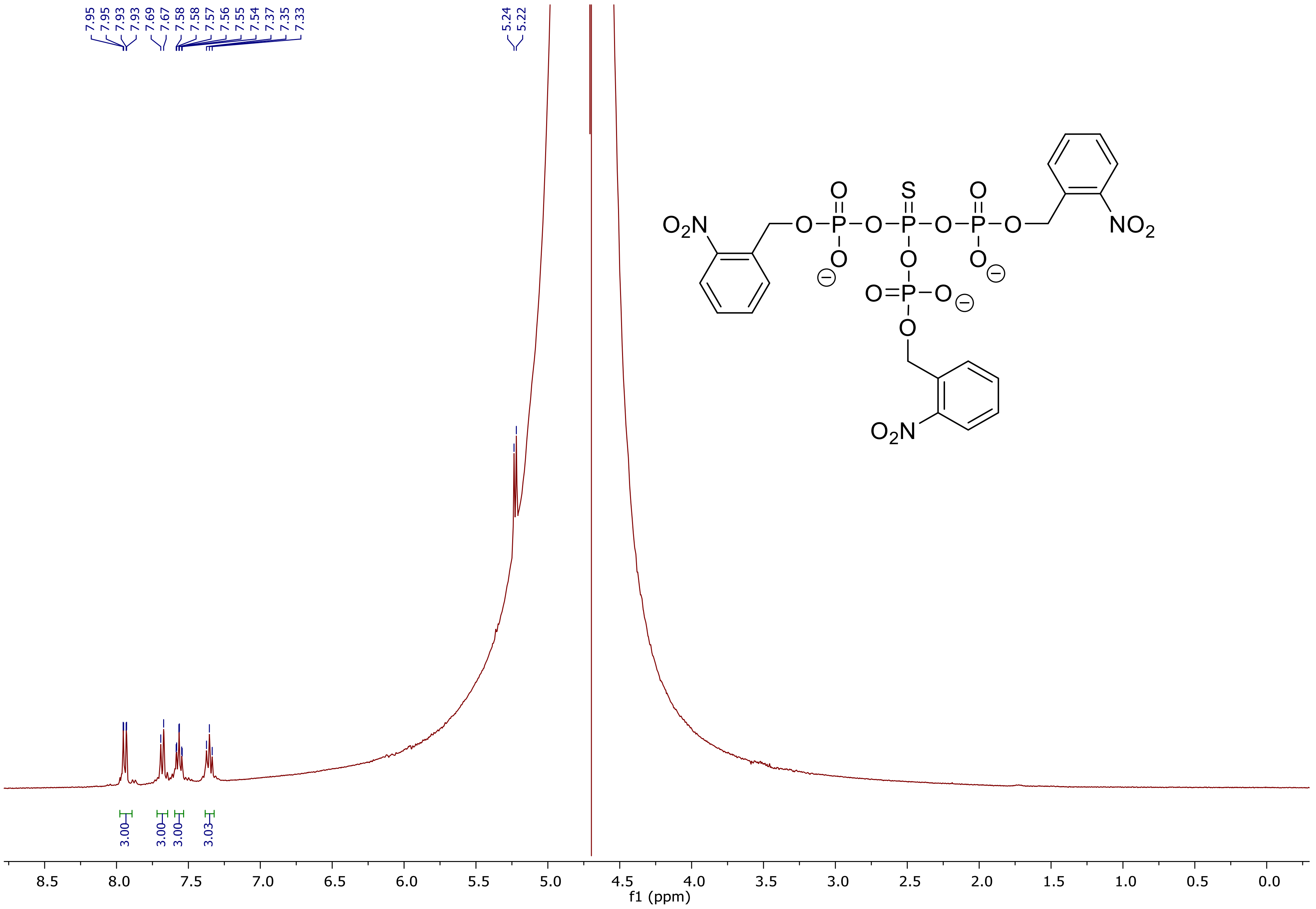

Supplementary Fig. 62 | **^{31}P{^{1}H}-NMR** (162 MHz, D_2O), compound **33**:

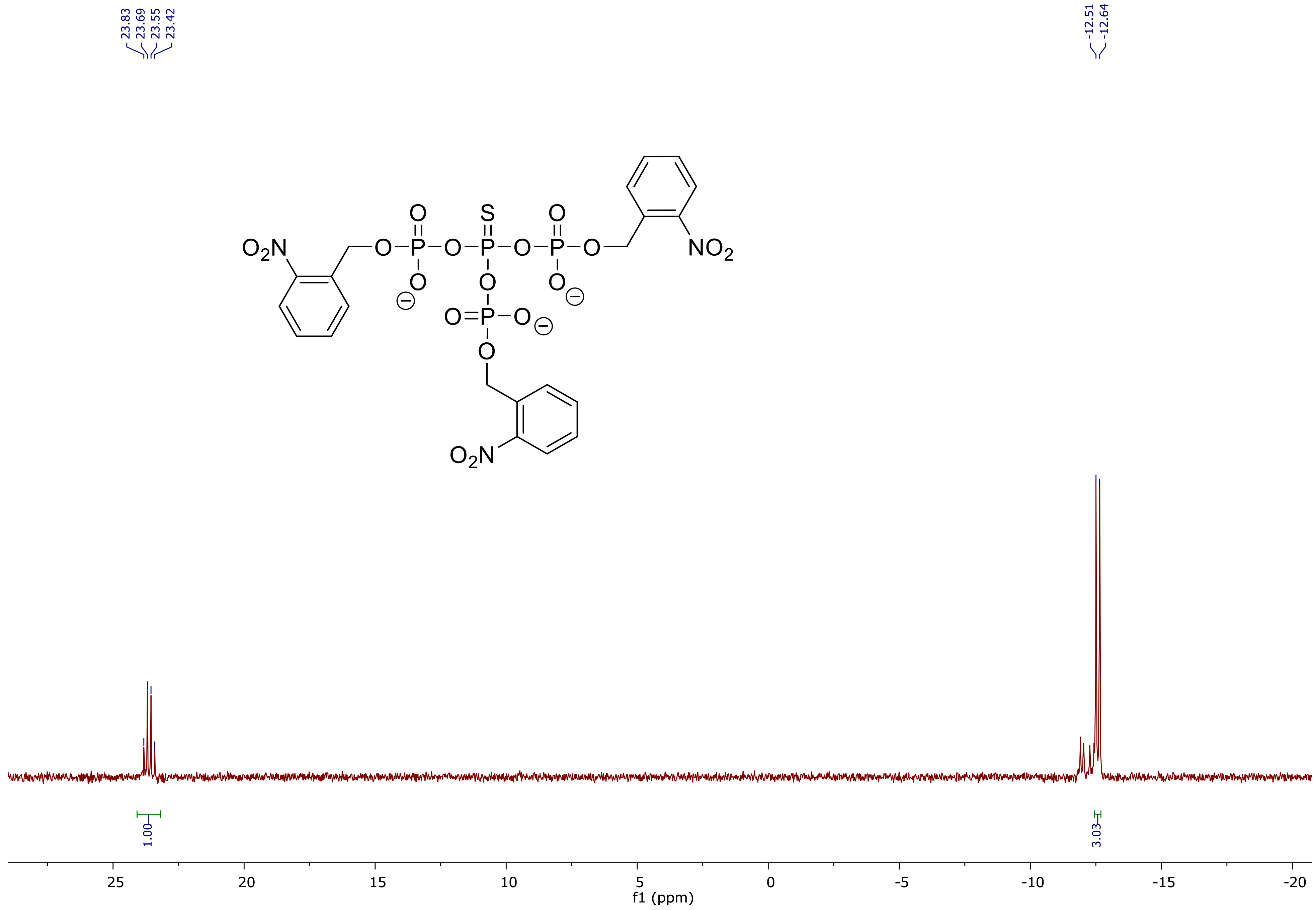

Supplementary Fig. 63│ **^{31}P-NMR** (162 MHz, D_2O), compound **33**:

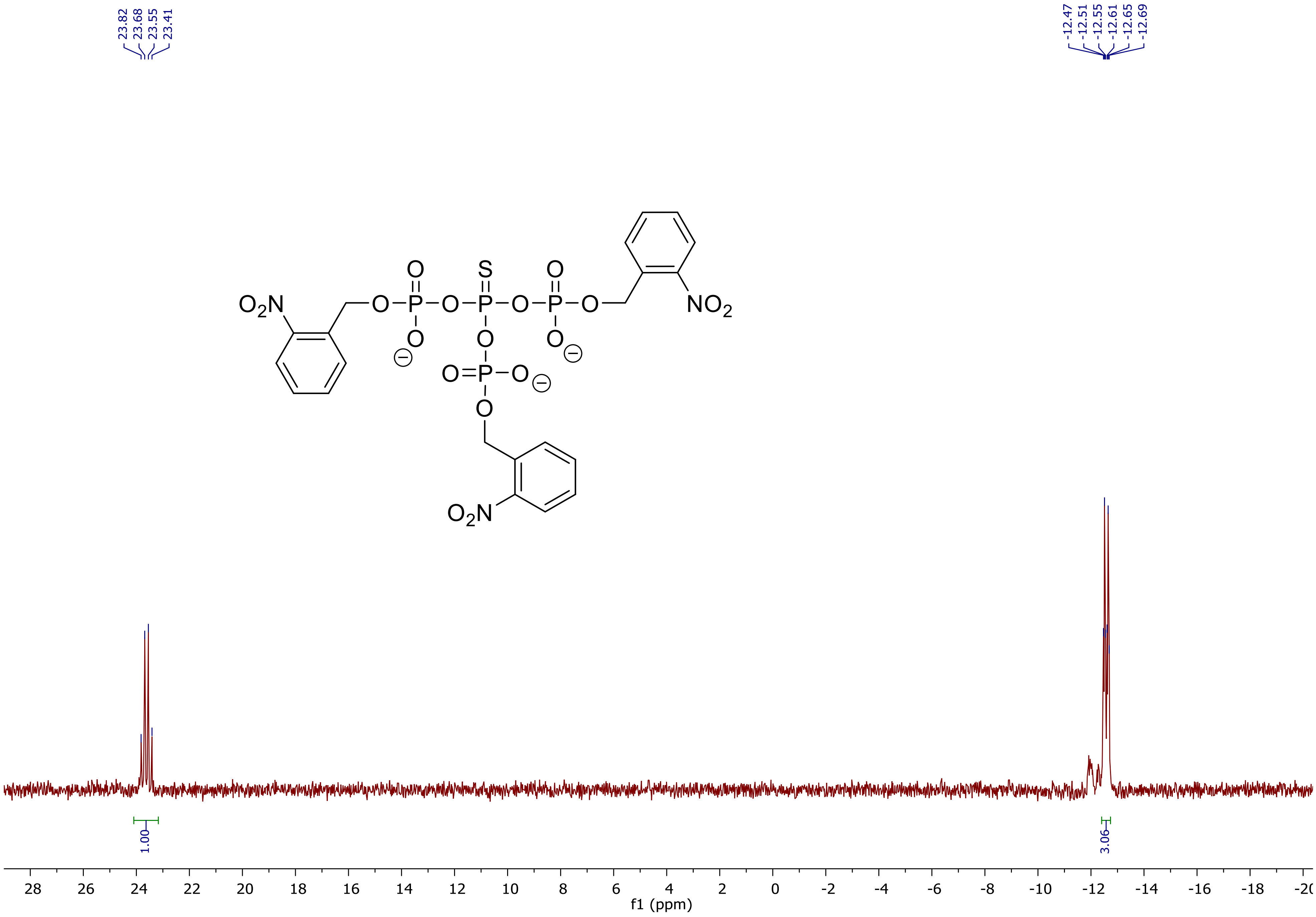

Supplementary Fig. 64│ [1]**H-NMR** (400 MHz, D_2O, presat), compound **34**:

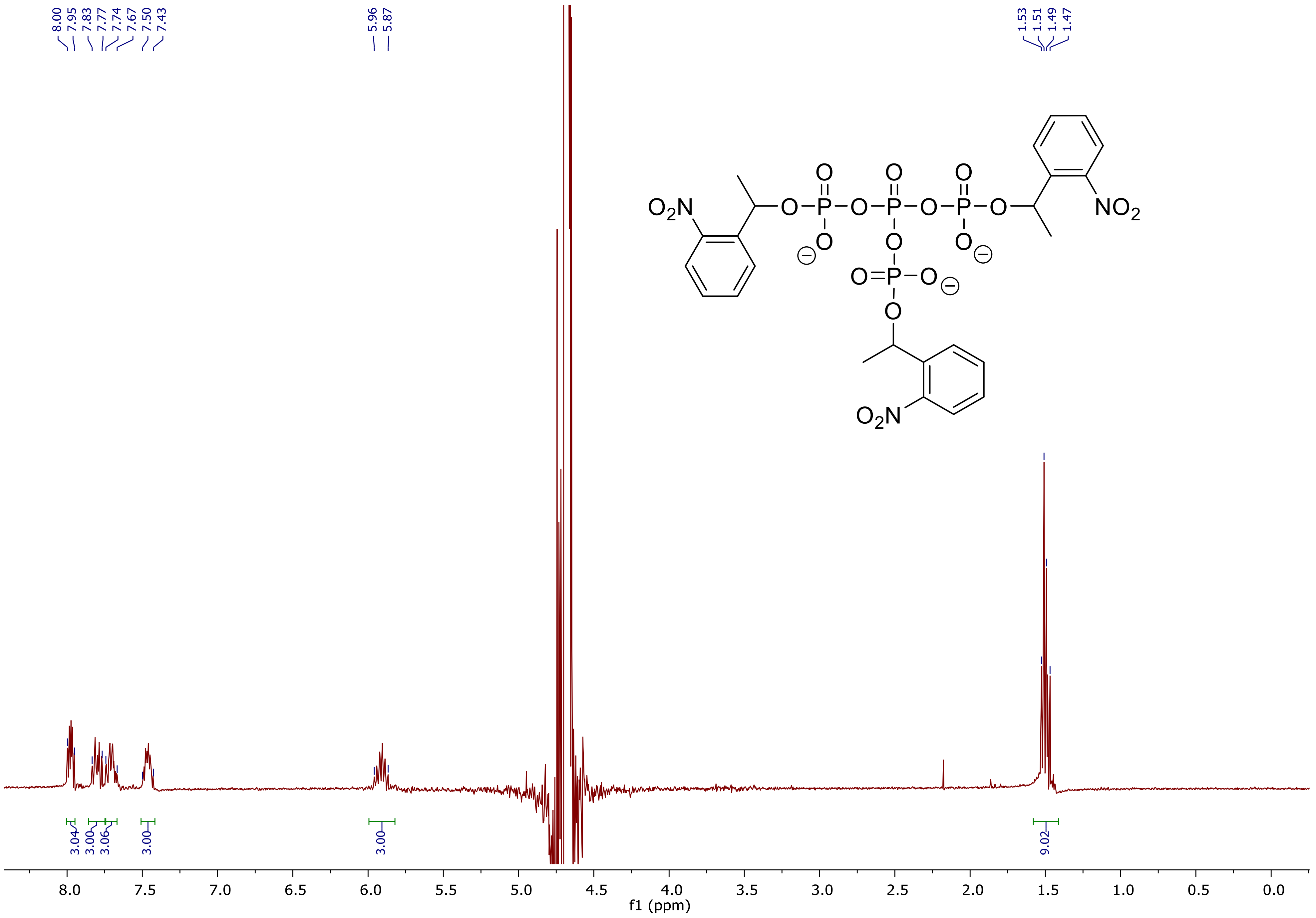

Supplementary Fig. 65 | **^{31}P{^{1}H}-NMR** (162 MHz, D_2O), compound **34**:

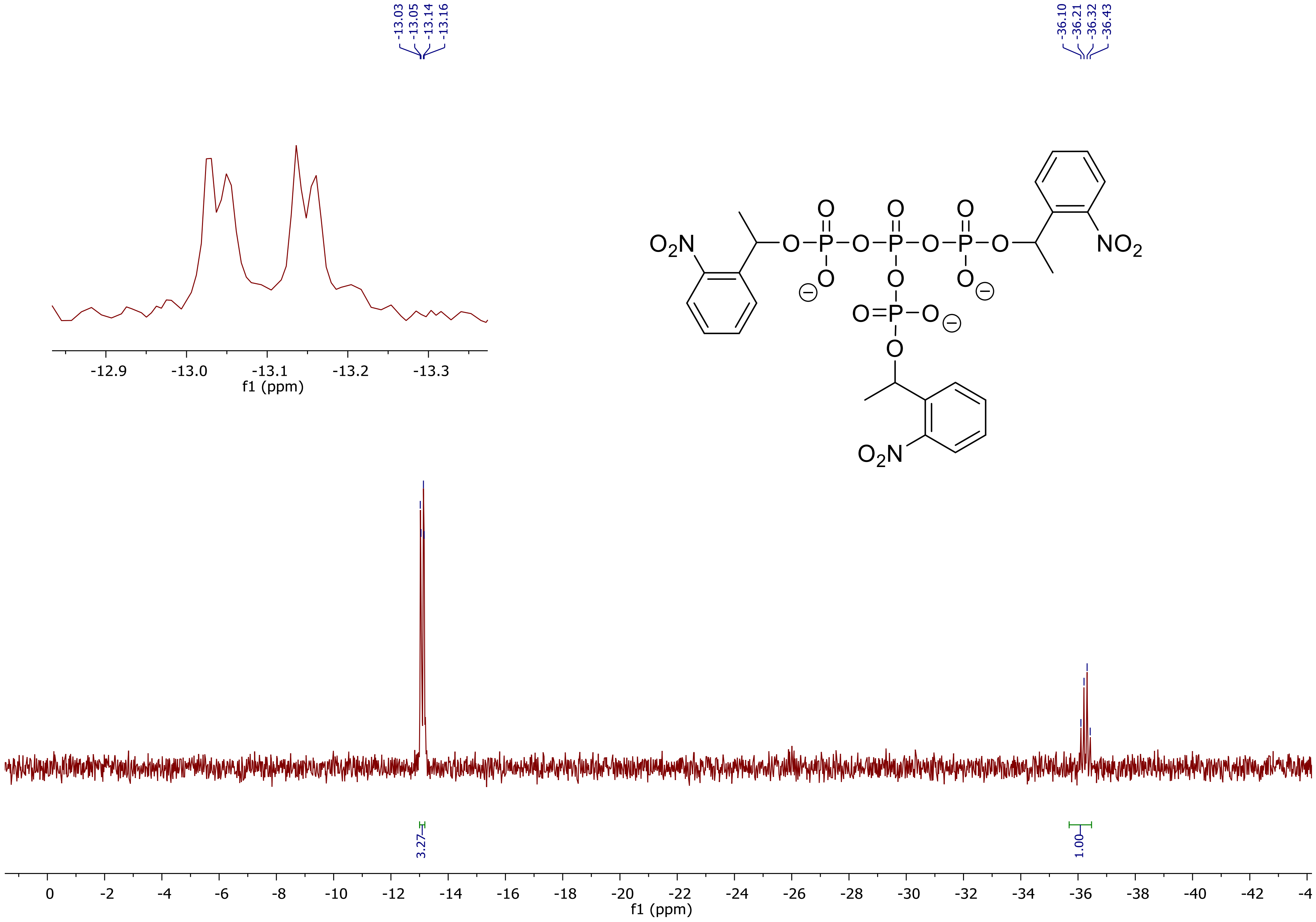

Supplementary Fig. 66| **^{31}P-NMR** (162 MHz, D_2O), compound **34**:

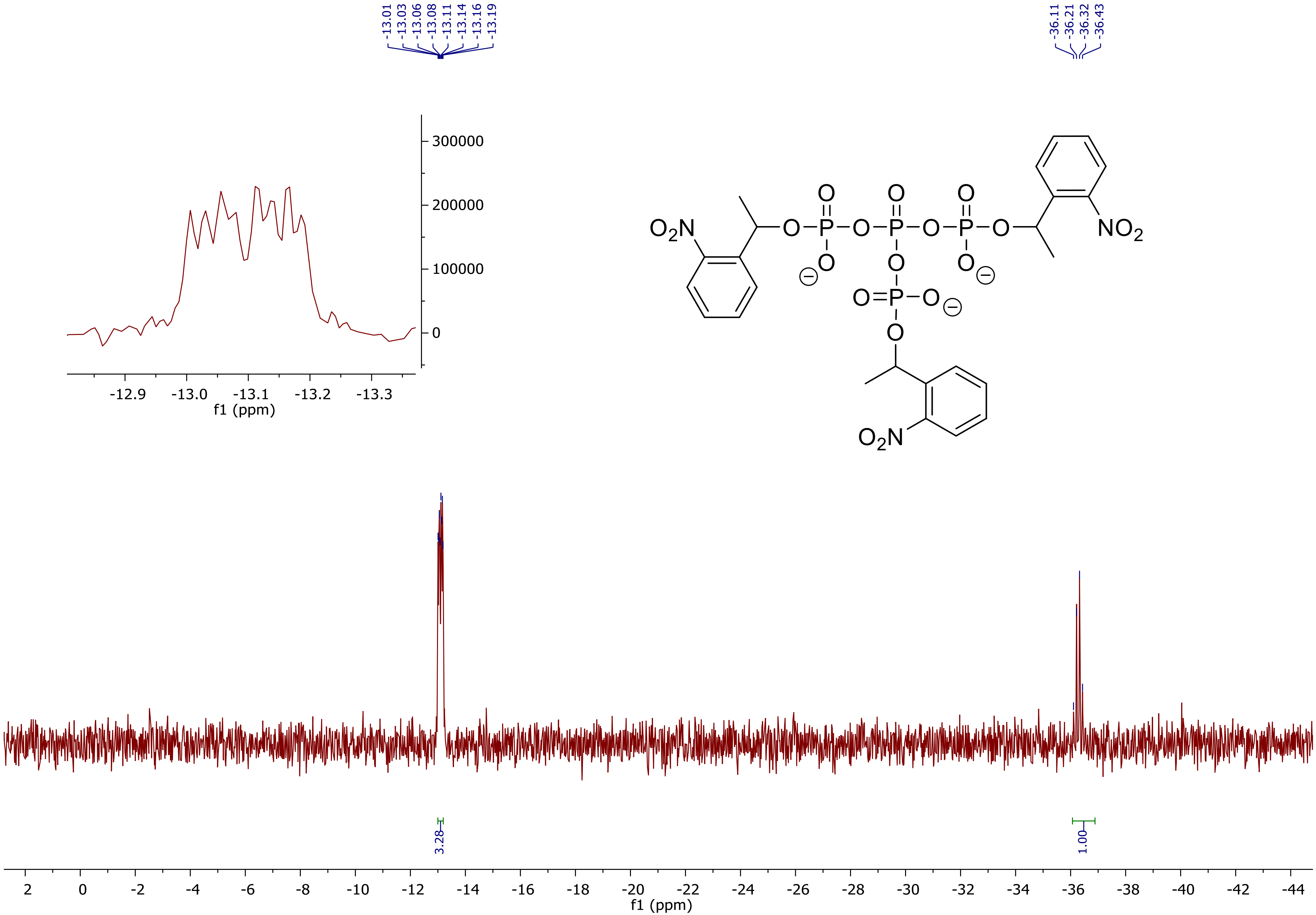

Supplementary Fig. 67| ^{1}**H-NMR** (500 MHz, D_2O, presat), compound **35**:

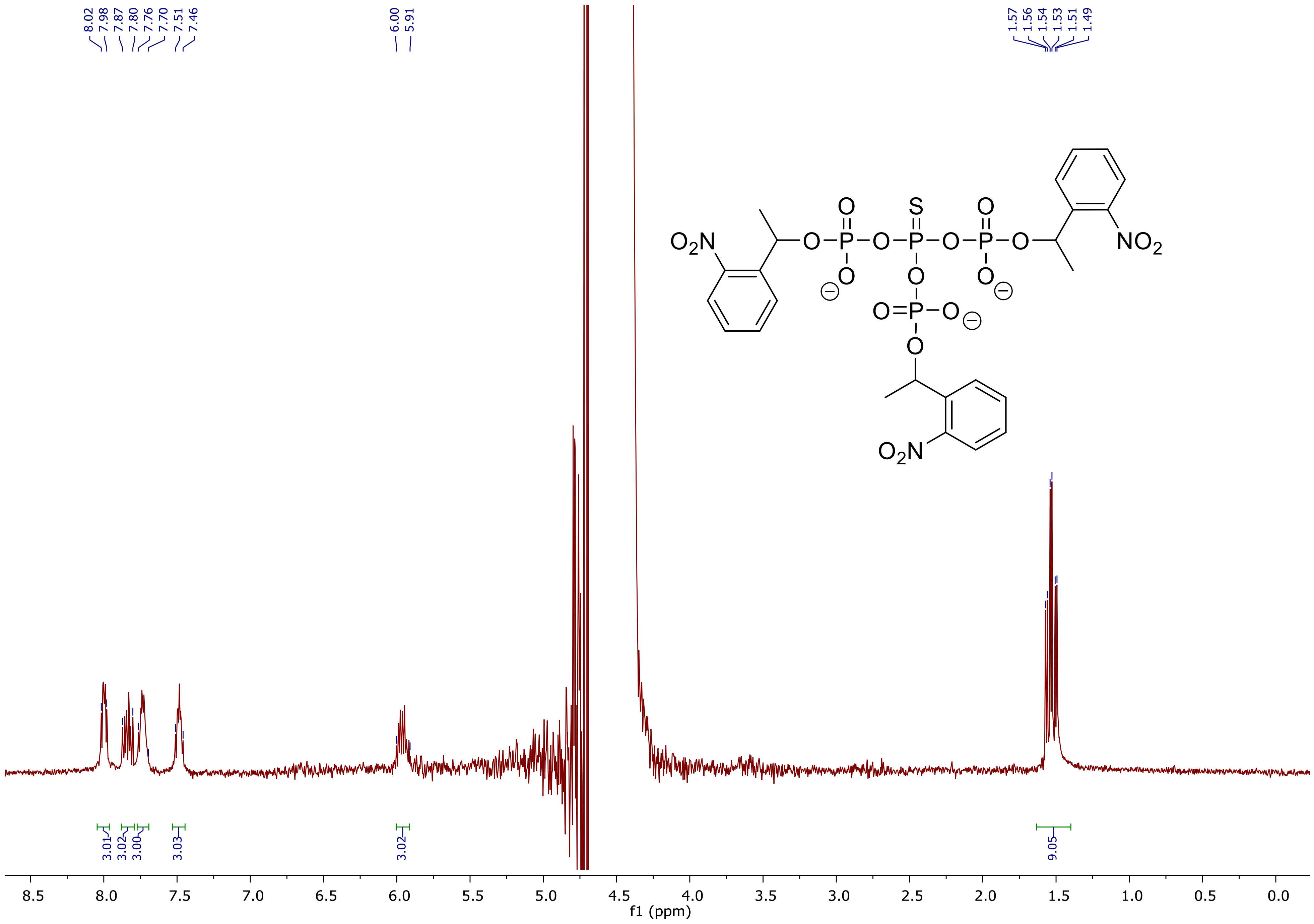

Supplementary Fig. 68| ^{31}**P{^{1}H}-NMR** (202 MHz, D_2O), compound **35**:

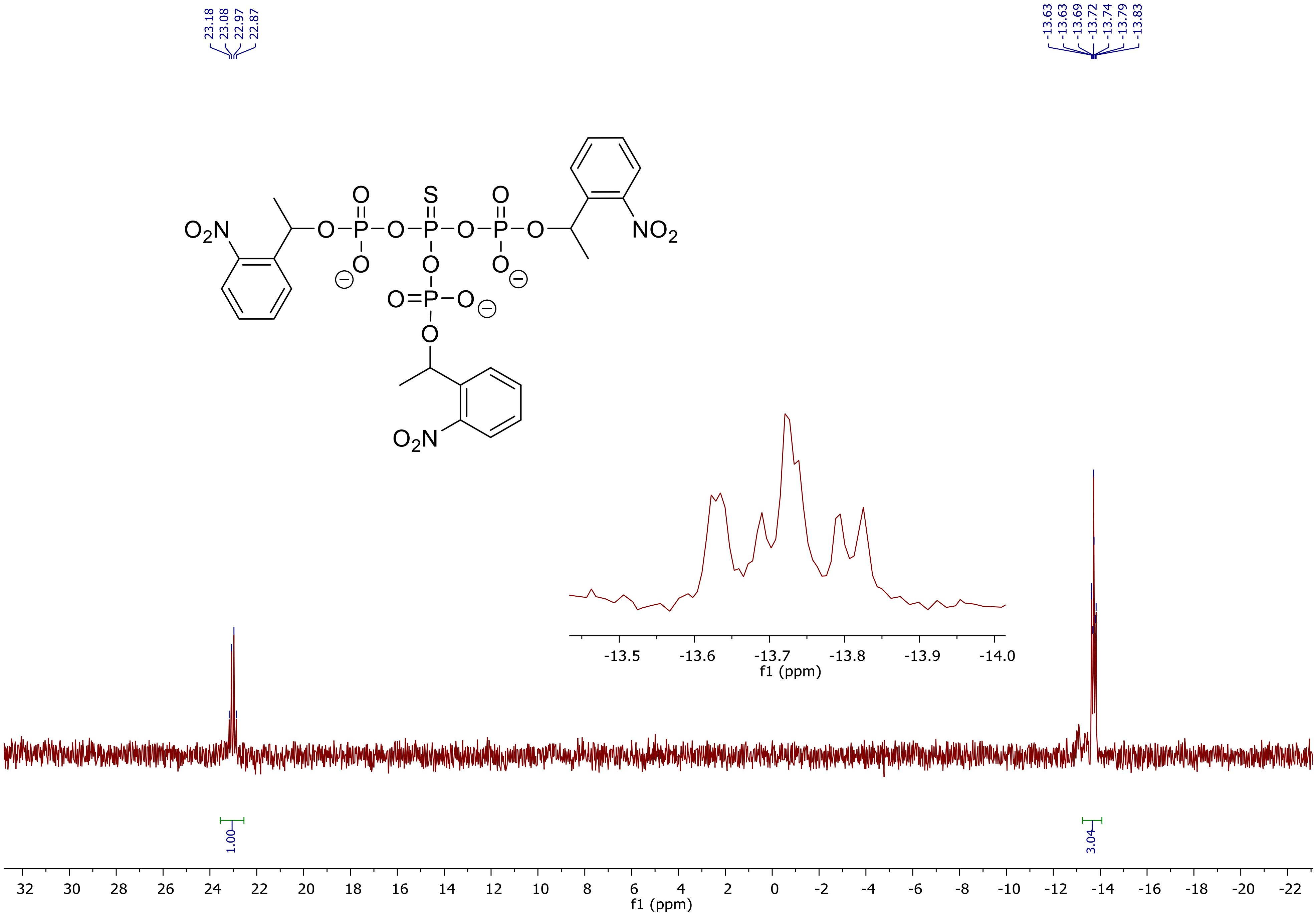

Supplementary Fig. 69| **^{31}P-NMR** (202 MHz, D_2O), compound **35**:

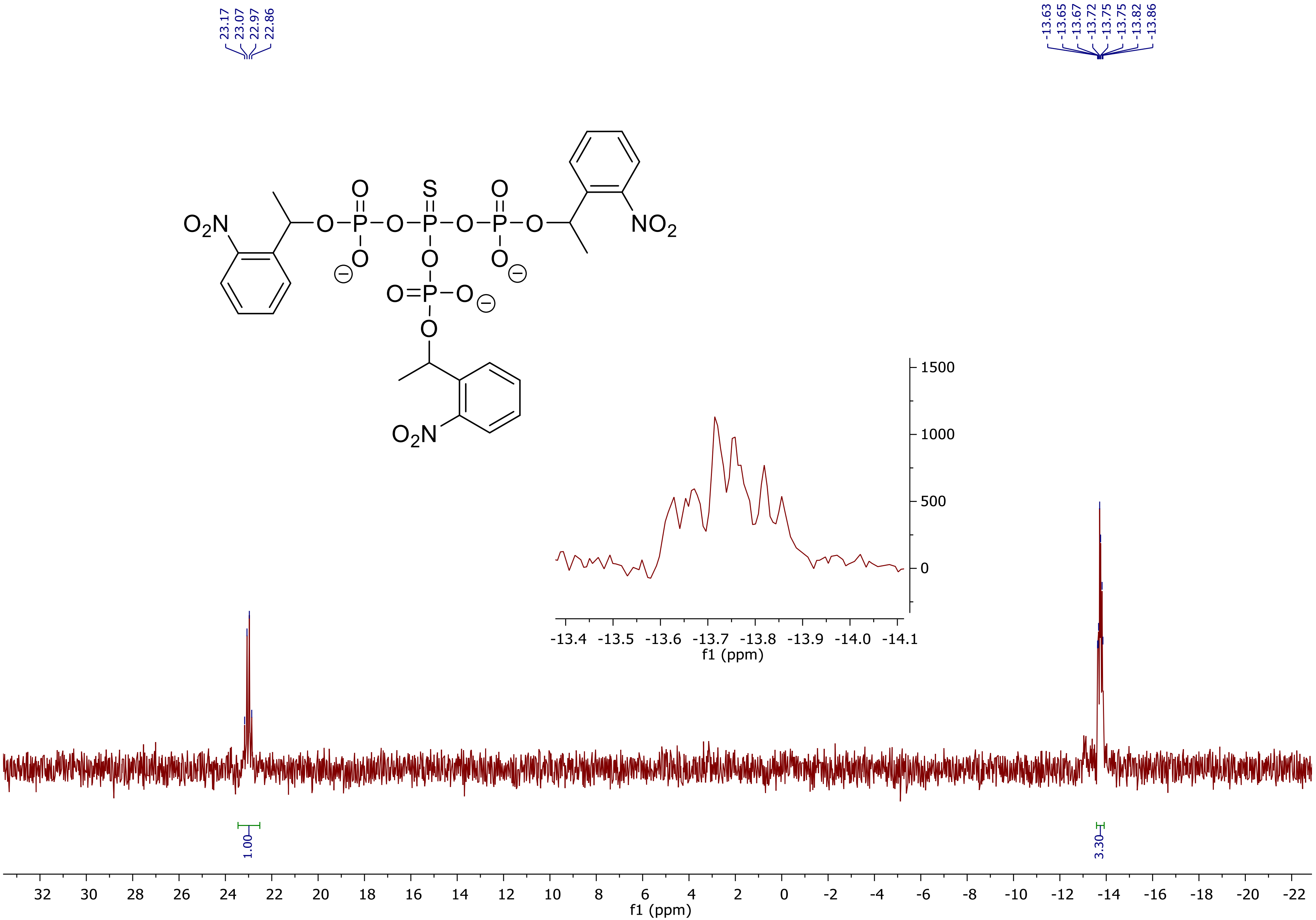

Supplementary Fig. 70 | [1]**H-NMR** (400 MHz, D_2O, presat), compound **36**:

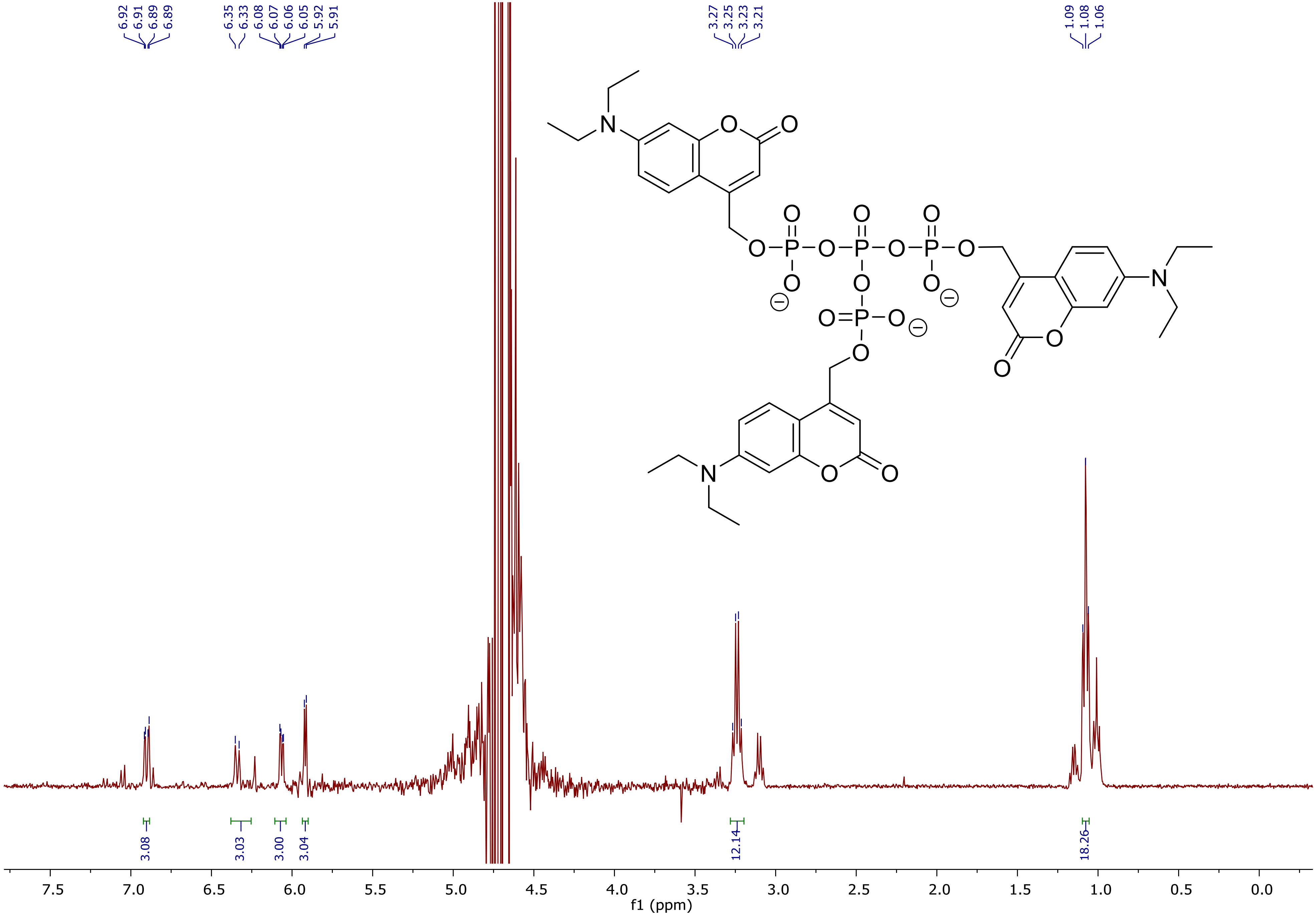

Supplementary Fig. 71 | **^{31}P{^{1}H}-NMR** (162 MHz, D_2O), compound **36**:

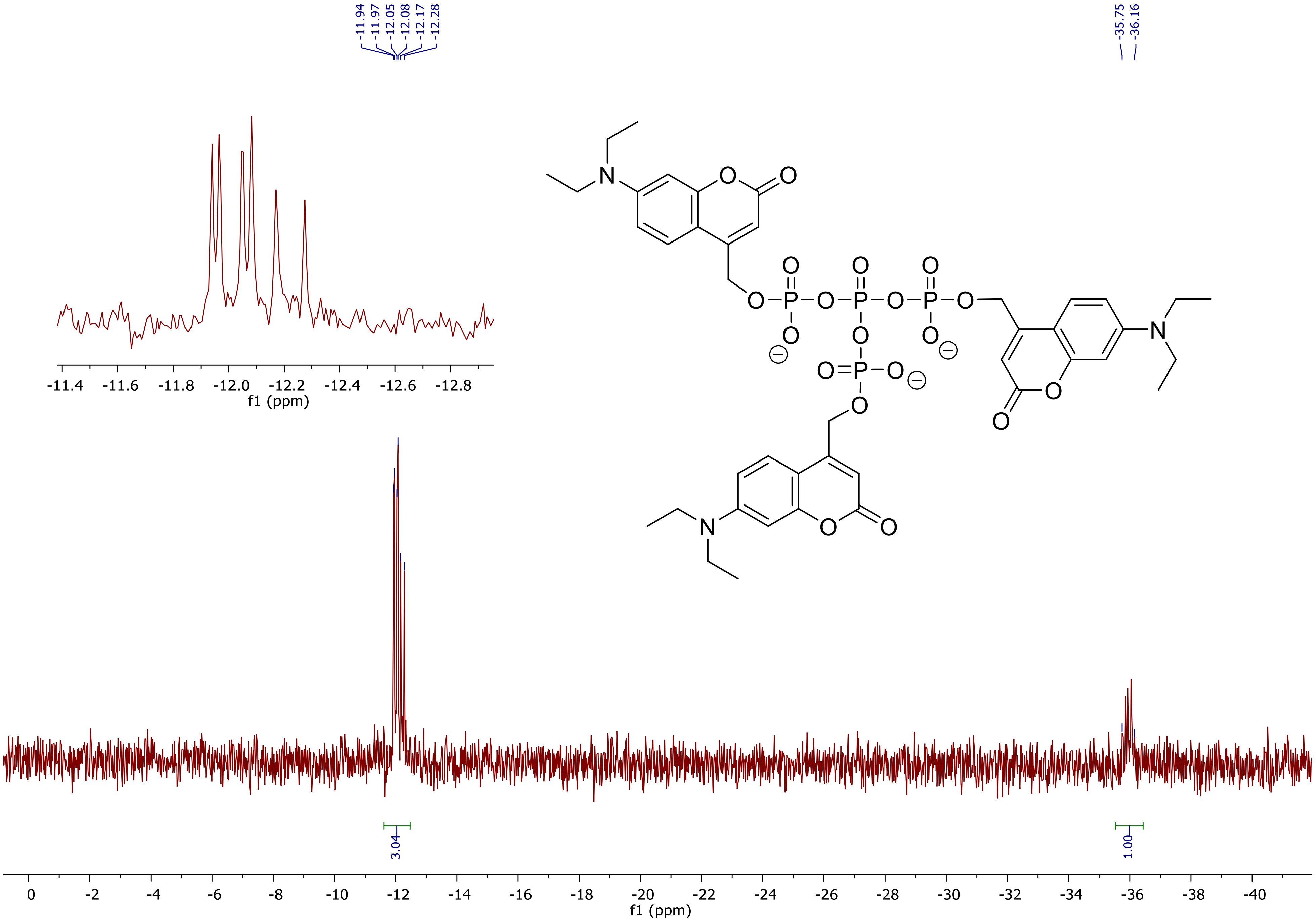

Supplementary Fig. 72| **^{31}P-NMR** (162 MHz, D_2O), compound **36**:

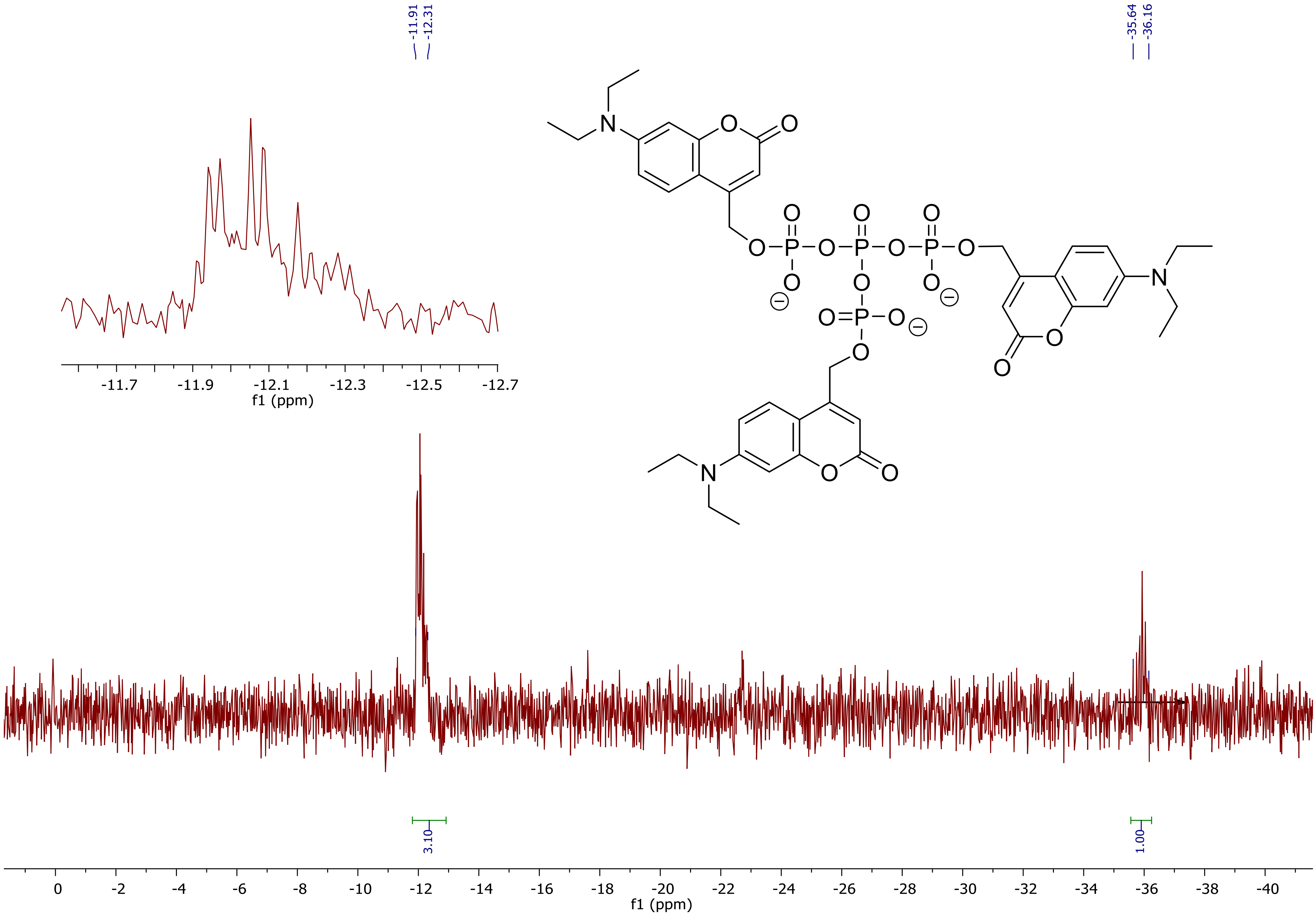

Supplementary Fig. 73│ **^{1}H-NMR** (400 MHz, D_2O, presat), compound **37**:

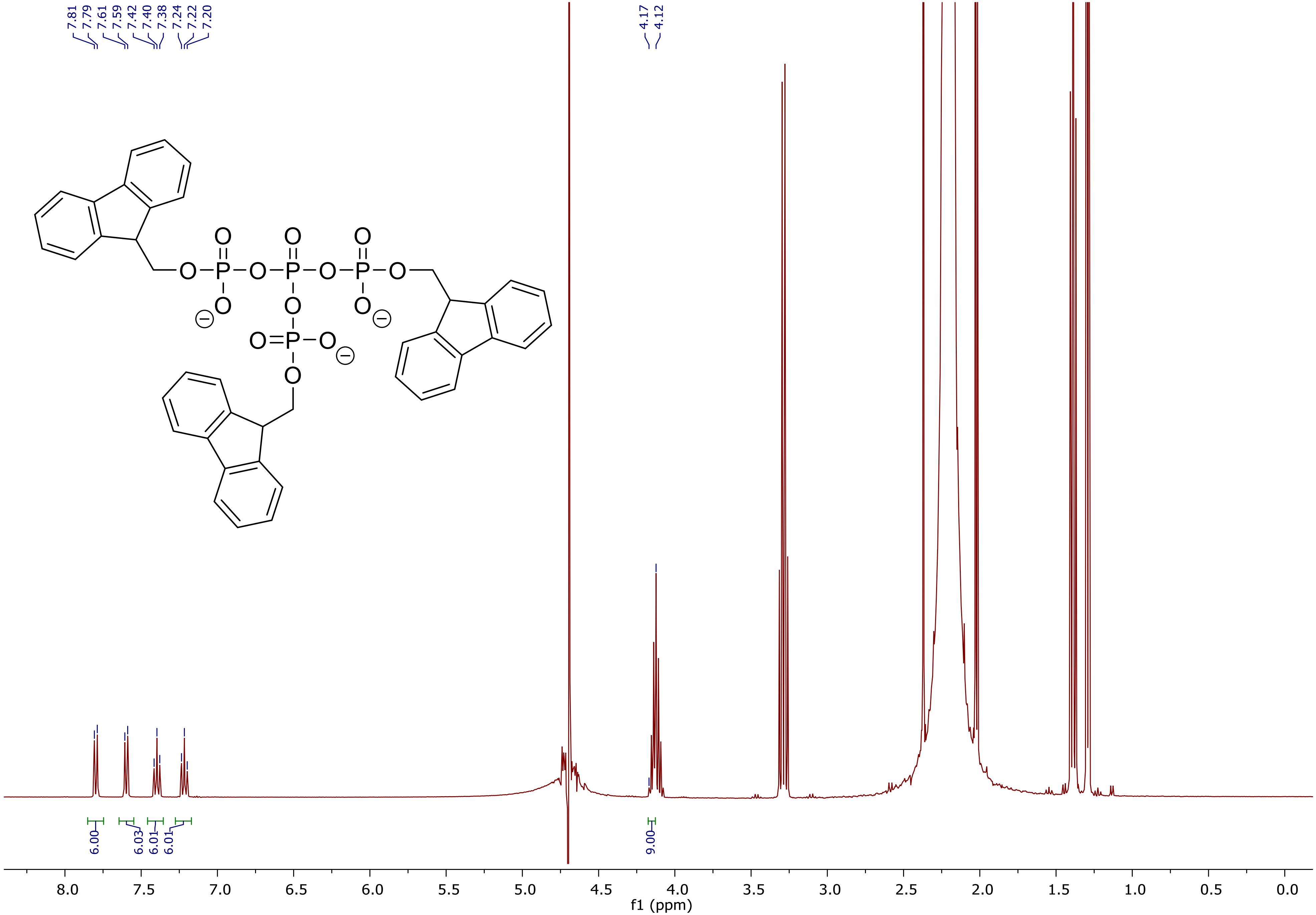

Supplementary Fig. 74 | **^{31}P{^{1}H}-NMR** (162 MHz, D_2O), compound **37**:

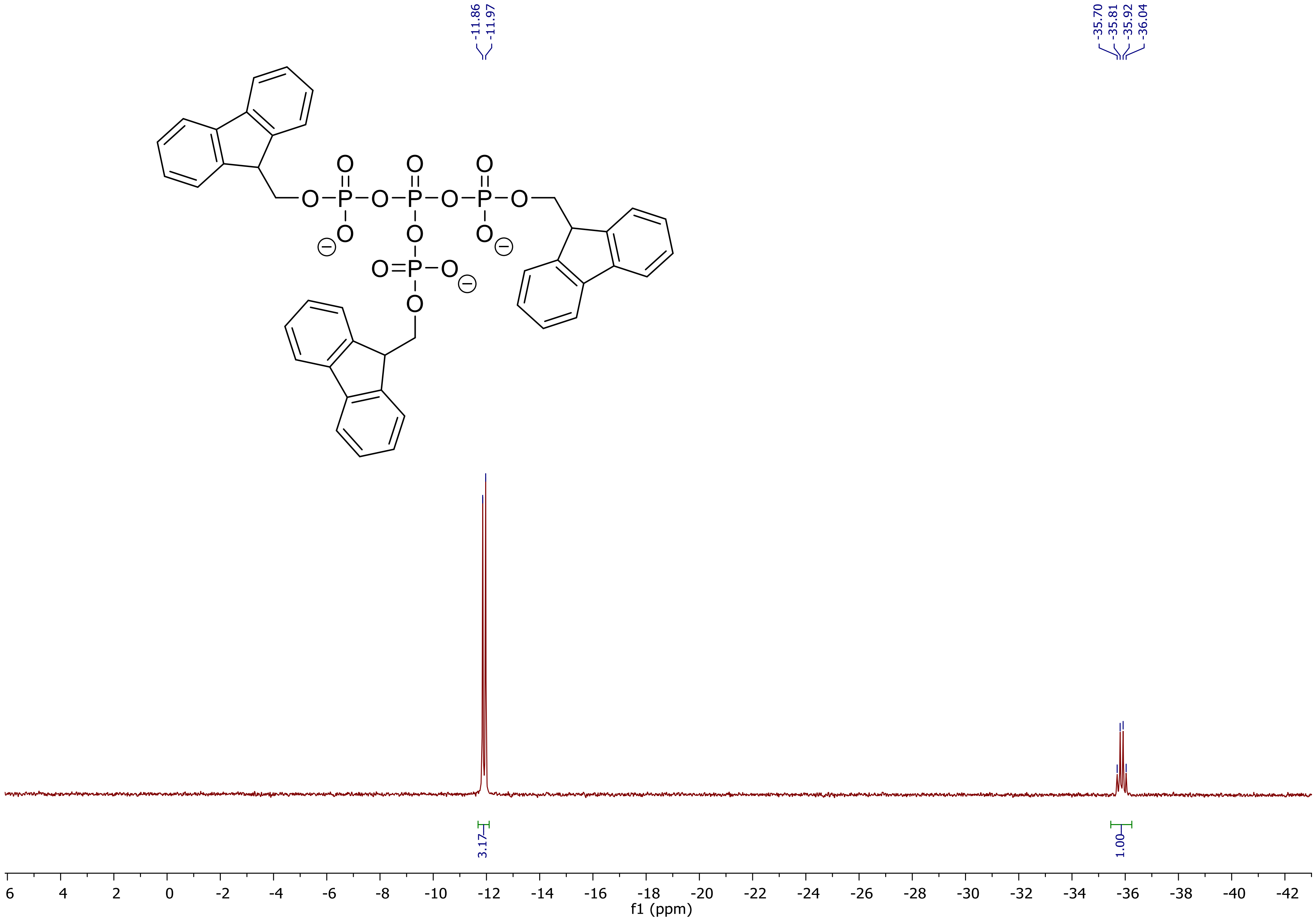

Supplementary Fig. 75 | **^{31}P-NMR** (162 MHz, D_2O), compound **37**:

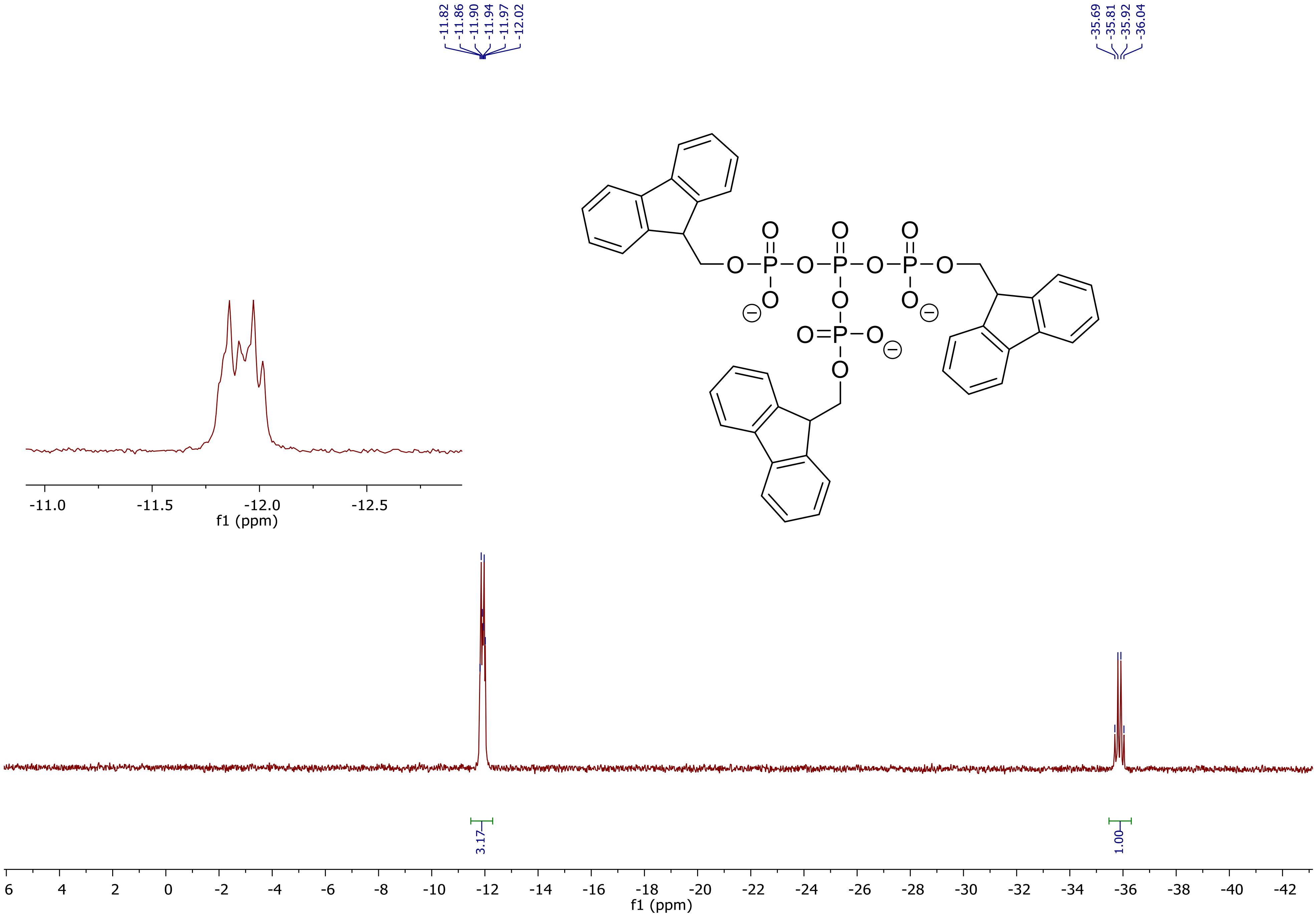

Supplementary Fig. 76 | **^{13}C-NMR** (101 MHz, D_2O), compound **37**:

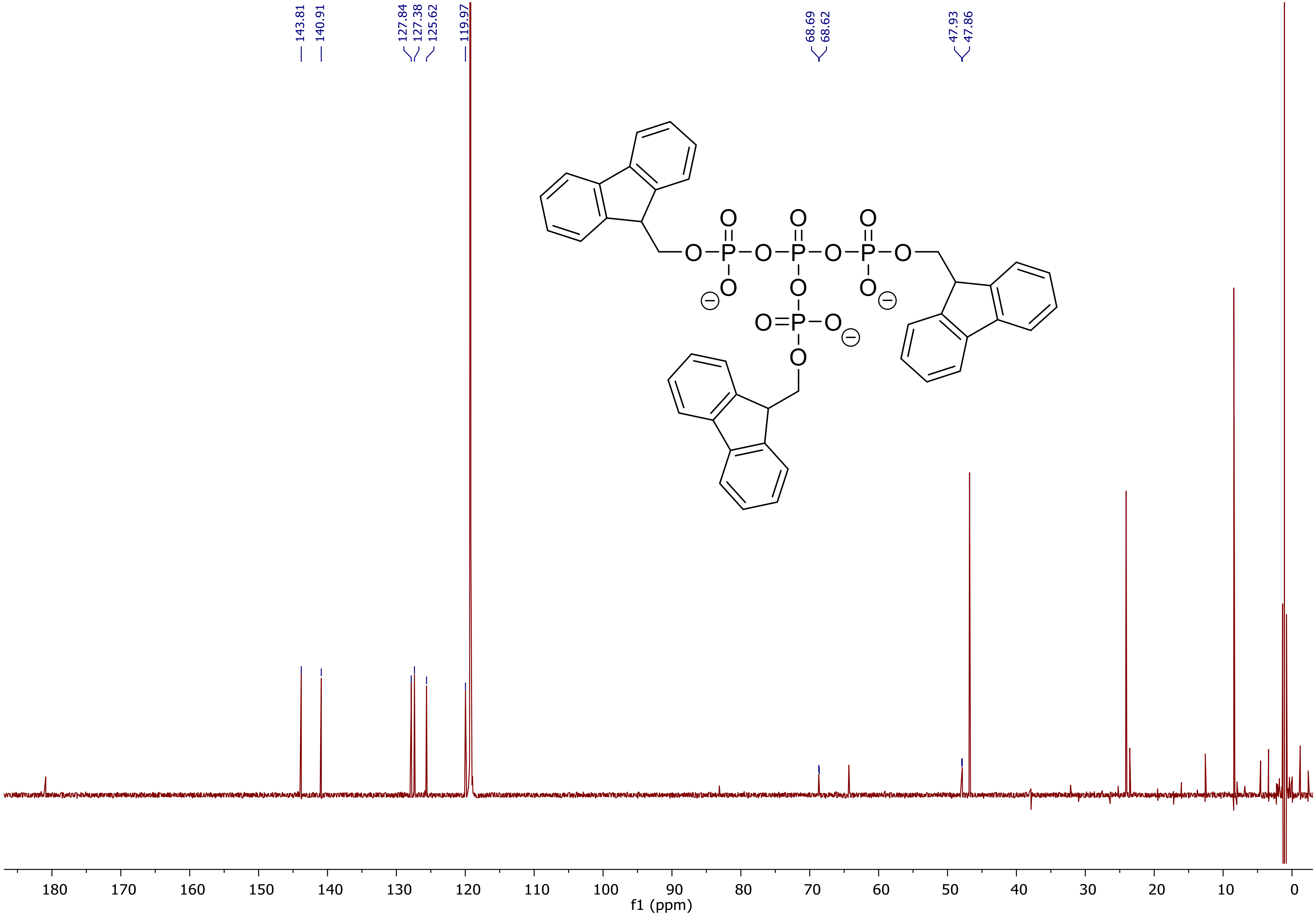

Supplementary Fig. 77│ **$^{31}P\{^{1}H\}$-NMR** (162 MHz, D_2O), compound **2**:

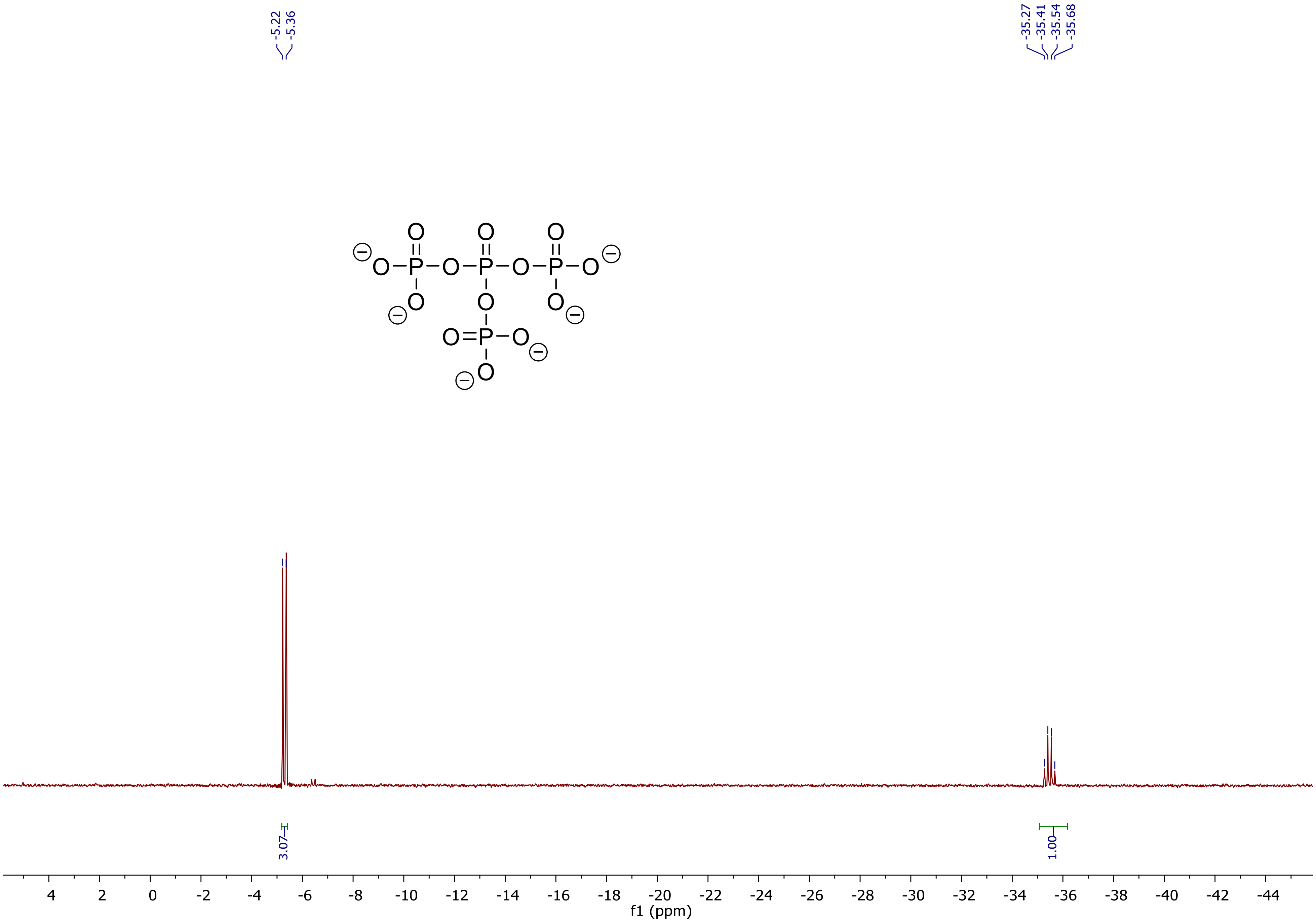

Supplementary Fig. 78| ^{1}**H-NMR** (400 MHz, CD_3CN), compound **2** $^{[PPN]}$:

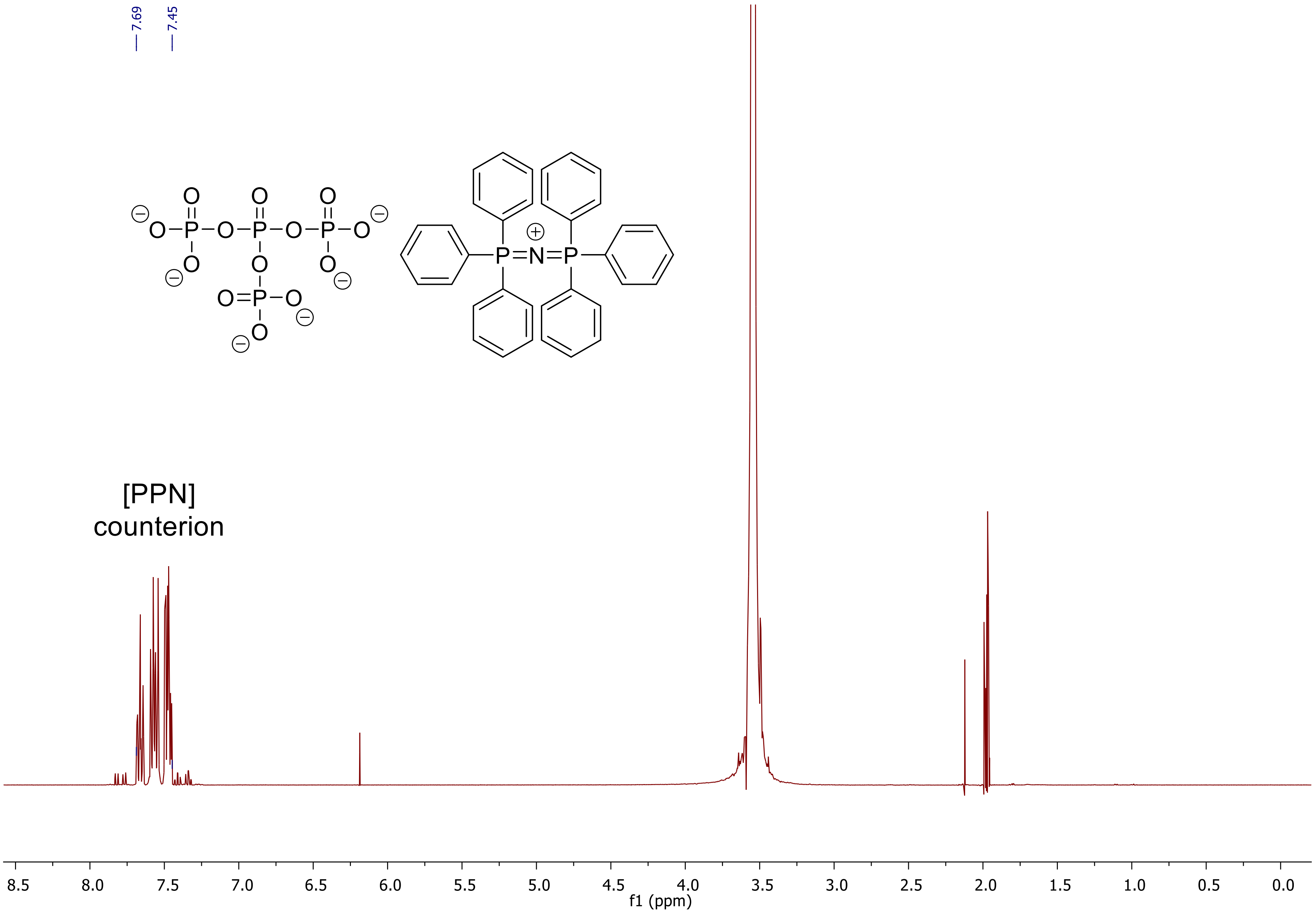

Supplementary Fig. 79 | **^{31}P{^{1}H}-NMR** (162 MHz, CD_3CN), compound **2** **[PPN]**:

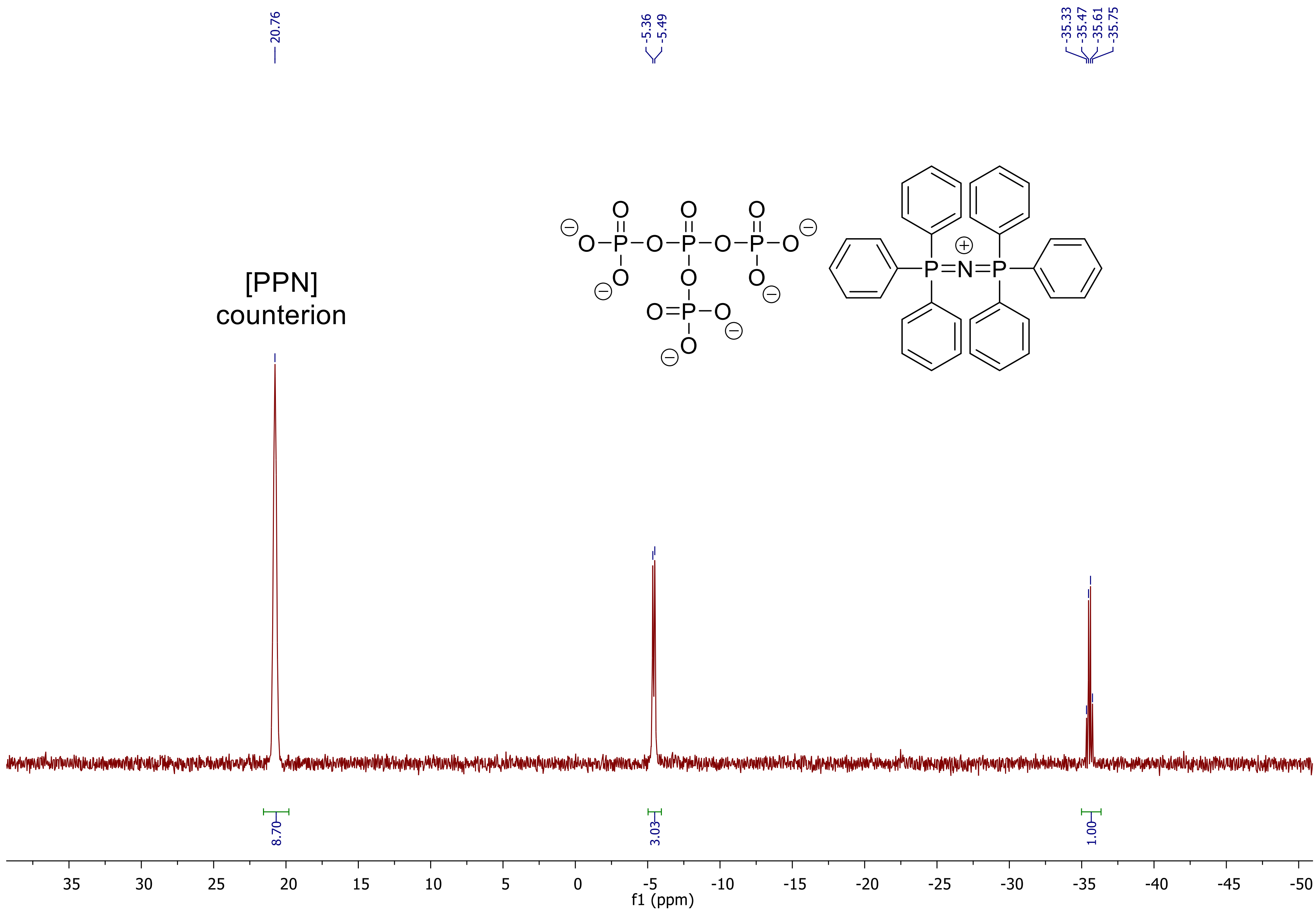

Supplementary Fig. 80 | **^{1}H-NMR** (400 MHz, D_2O, presat), compound **38**:

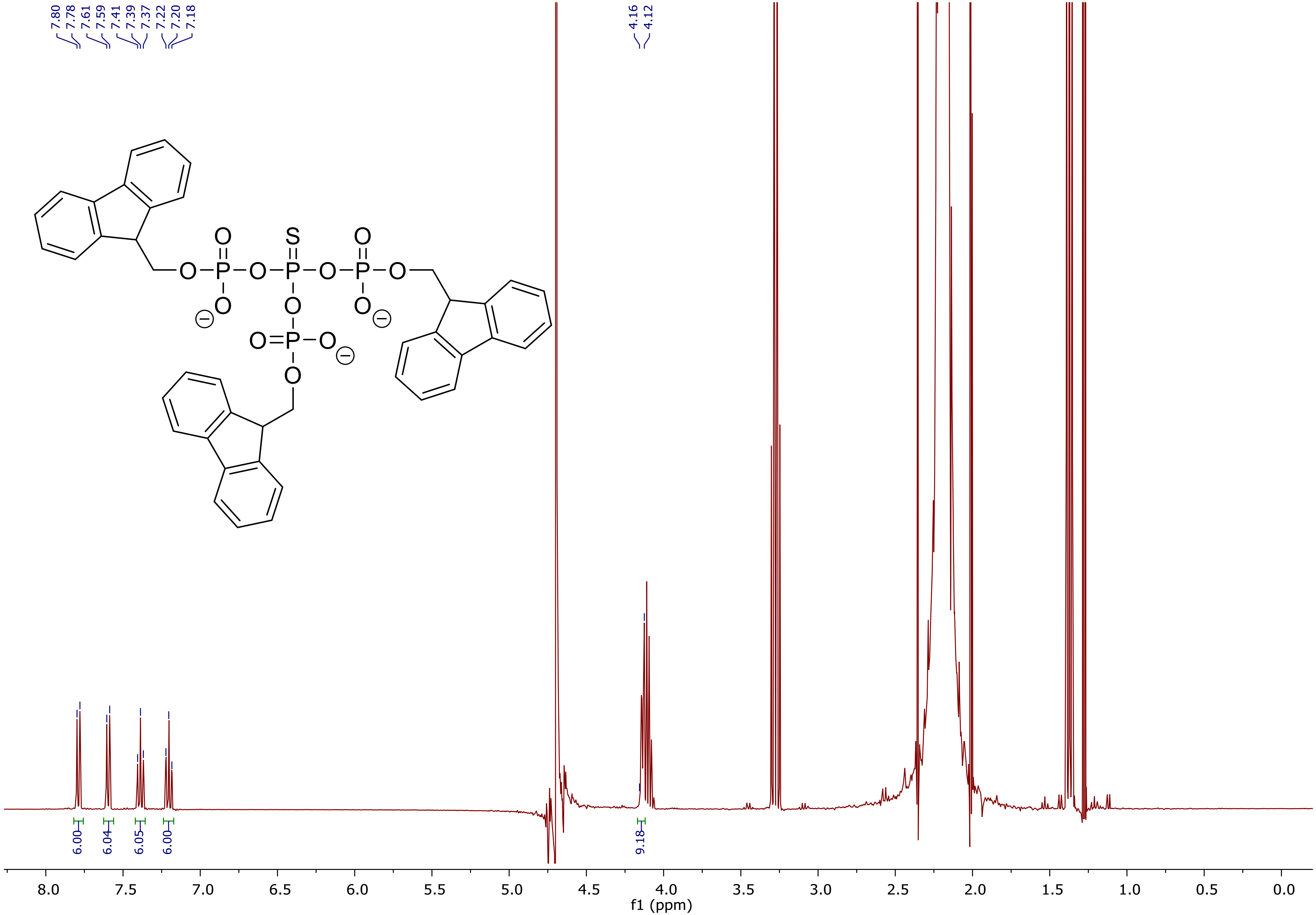

Supplementary Fig. 81│ **$^{31}P\{^{1}H\}$-NMR** (162 MHz, D_2O), compound **38**:

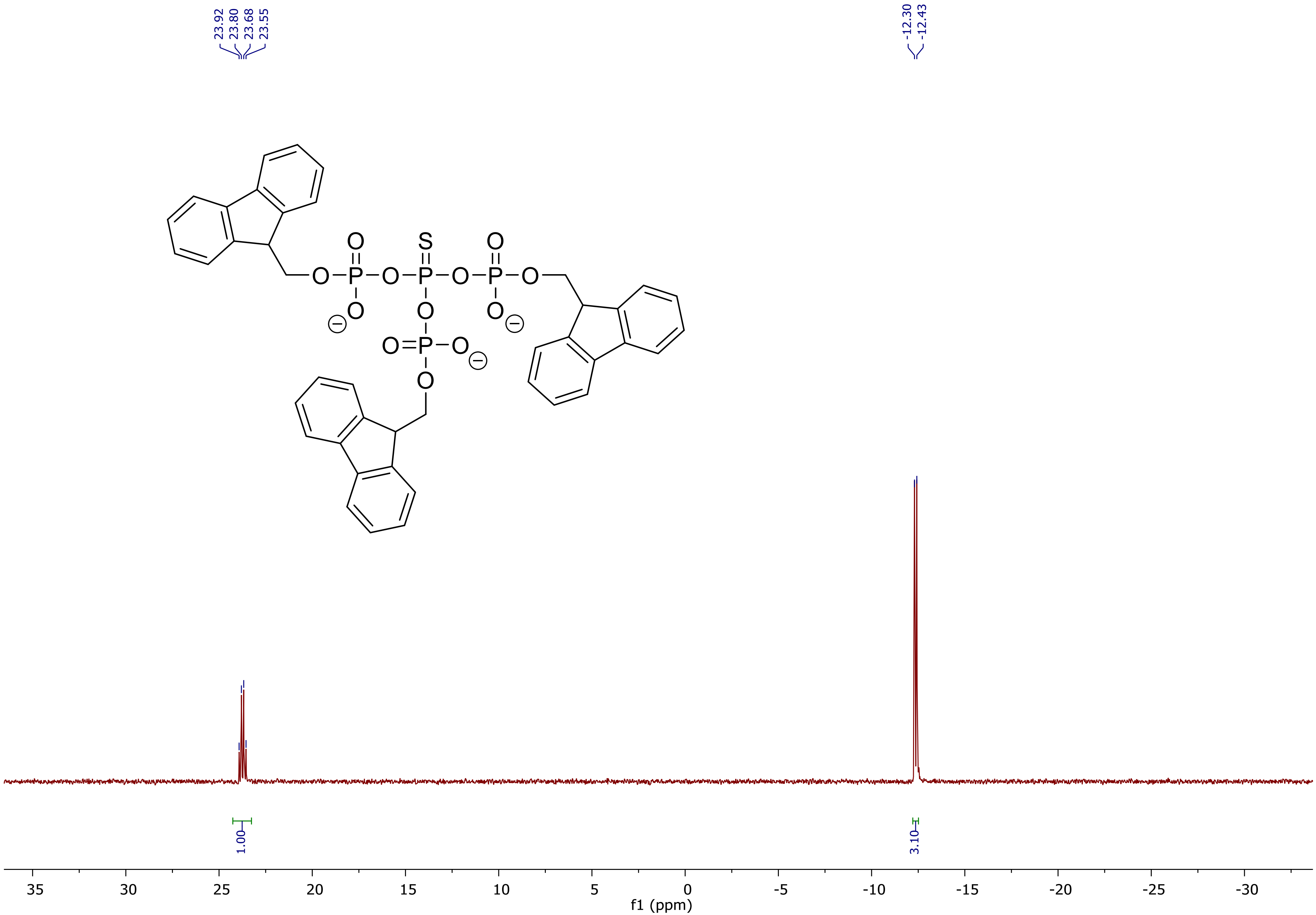

Supplementary Fig. 82 | **^{31}P-NMR** (162 MHz, D_2O), compound **38**:

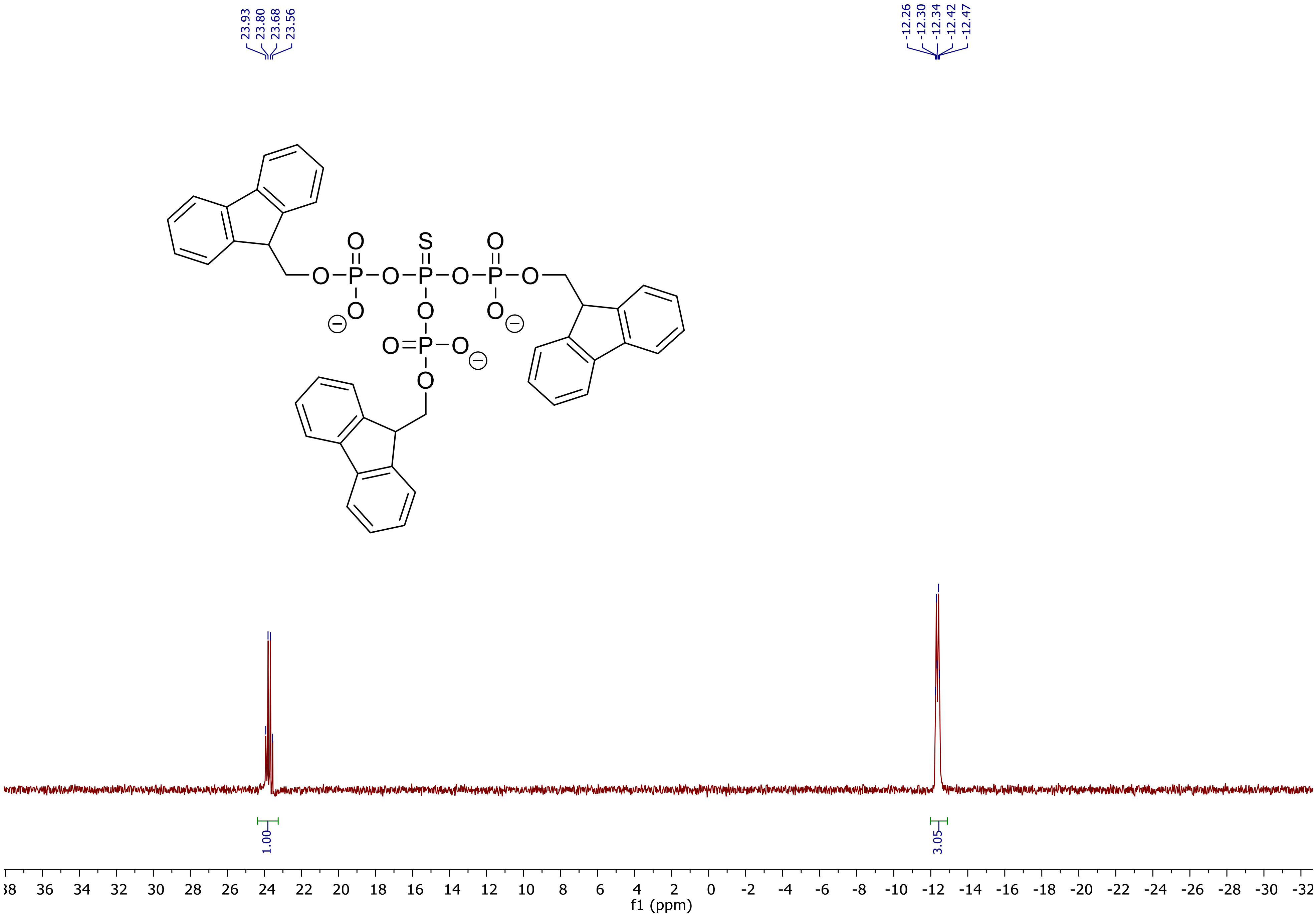

Supplementary Fig. 83 | $^{31}P\{^{1}H\}$-NMR (162 MHz, D_2O), compound **15**:

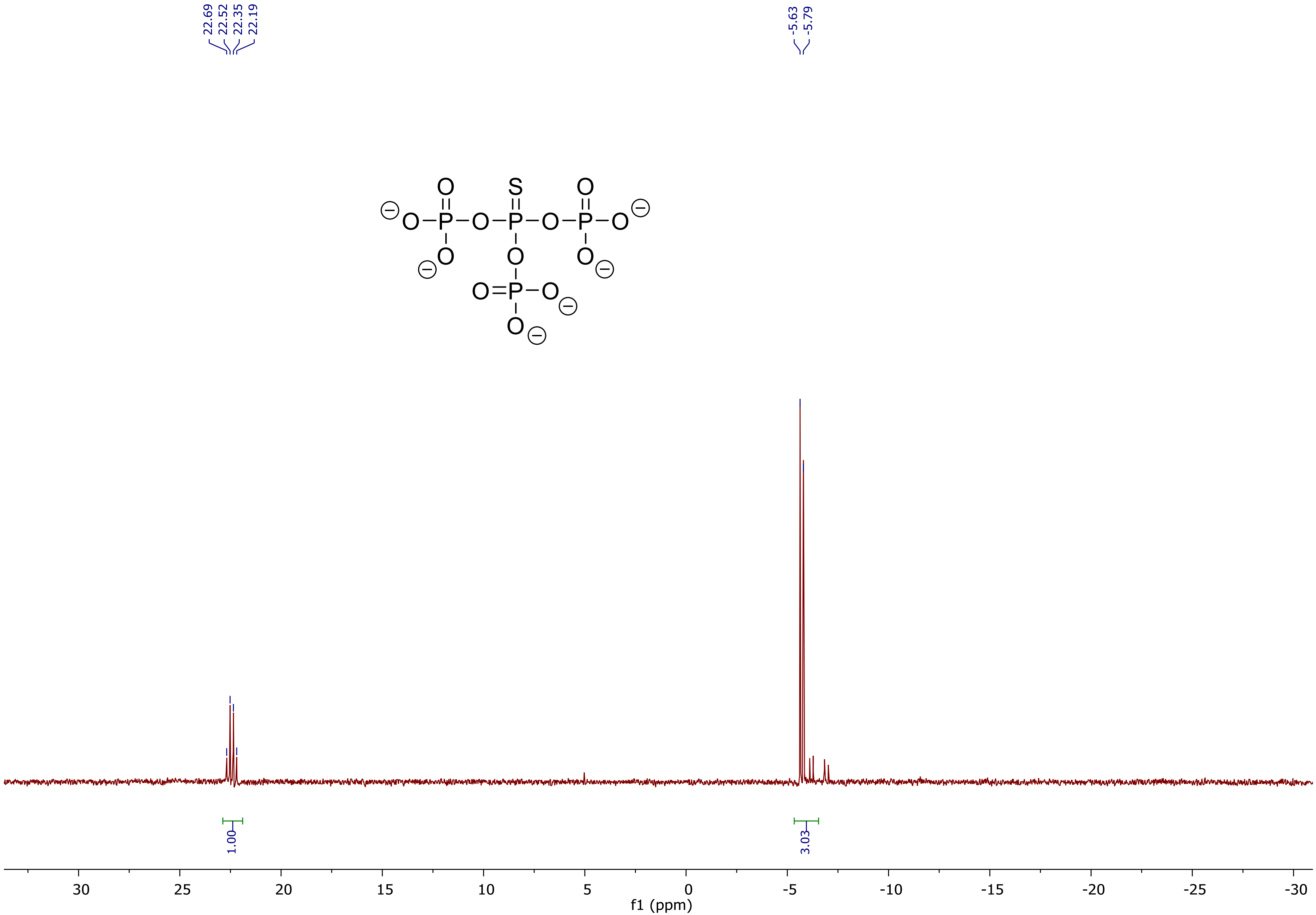

Supplementary Fig. 84 | **^{1}H-NMR** (400 MHz, D_2O, presat), compound **43**:

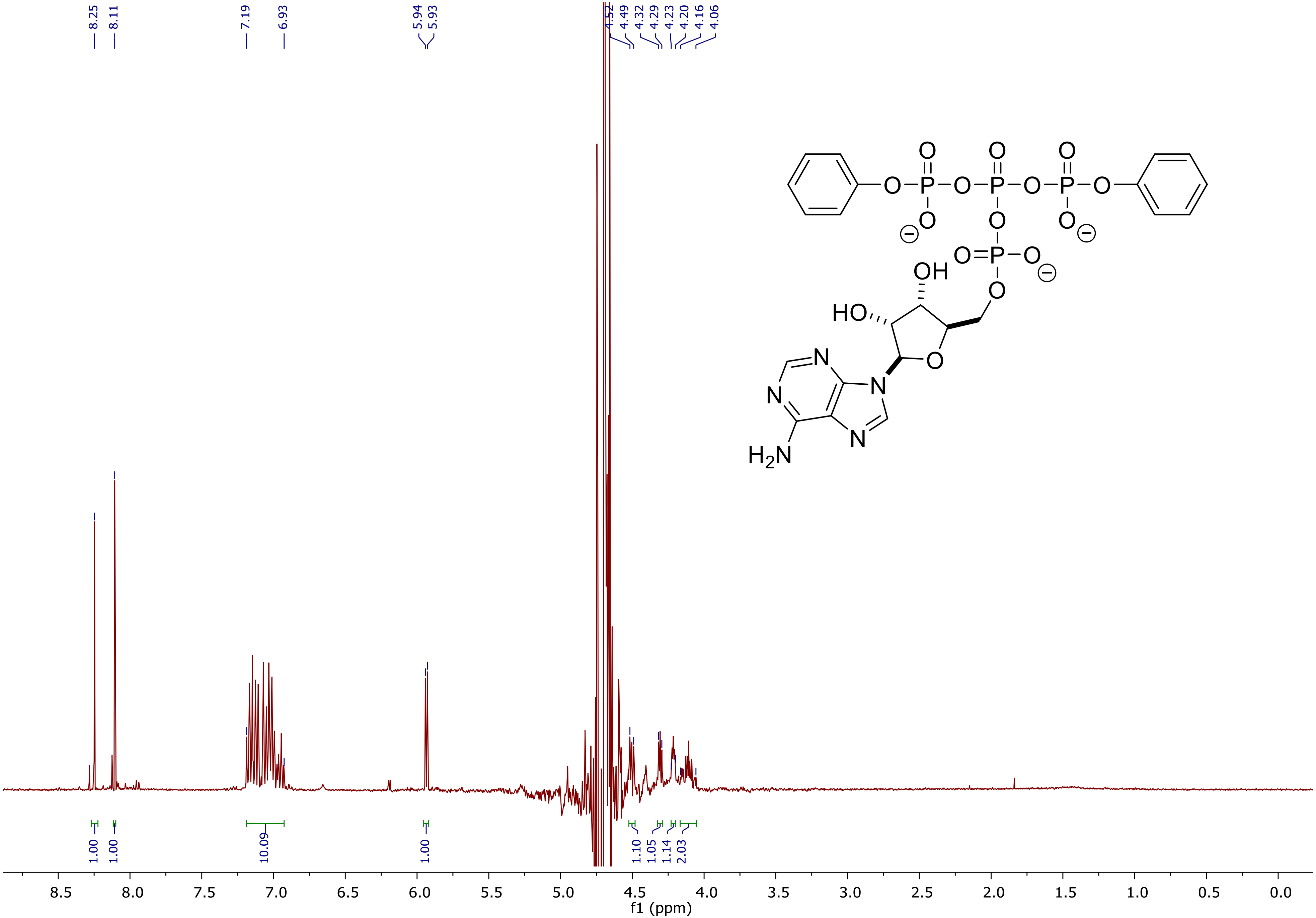

Supplementary Fig. 85 | **^{31}P{^{1}H}-NMR** (162 MHz, D_2O), compound **43**:

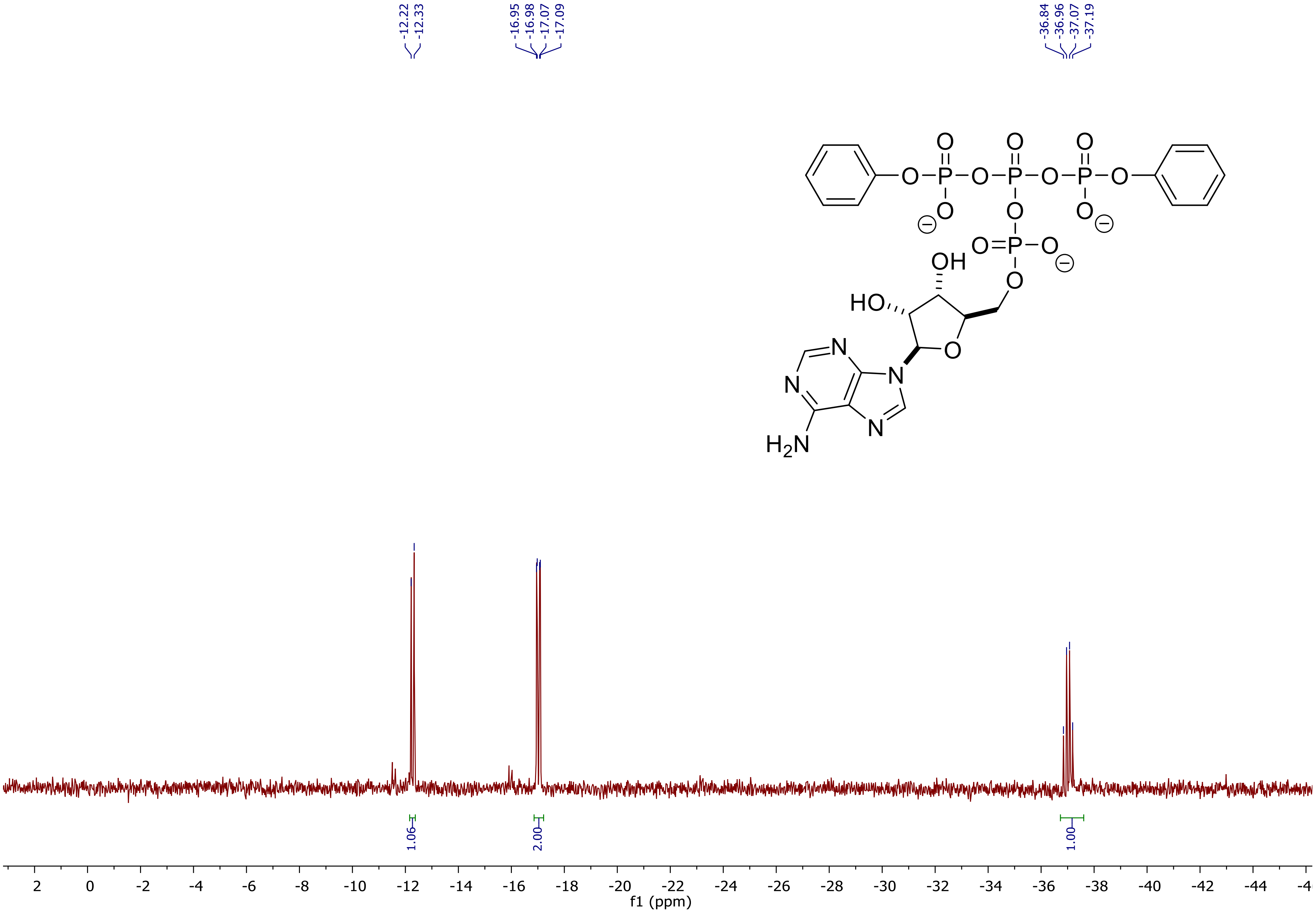

Supplementary Fig. 86 | **^{31}P-NMR** (162 MHz, D_2O), compound **43**:

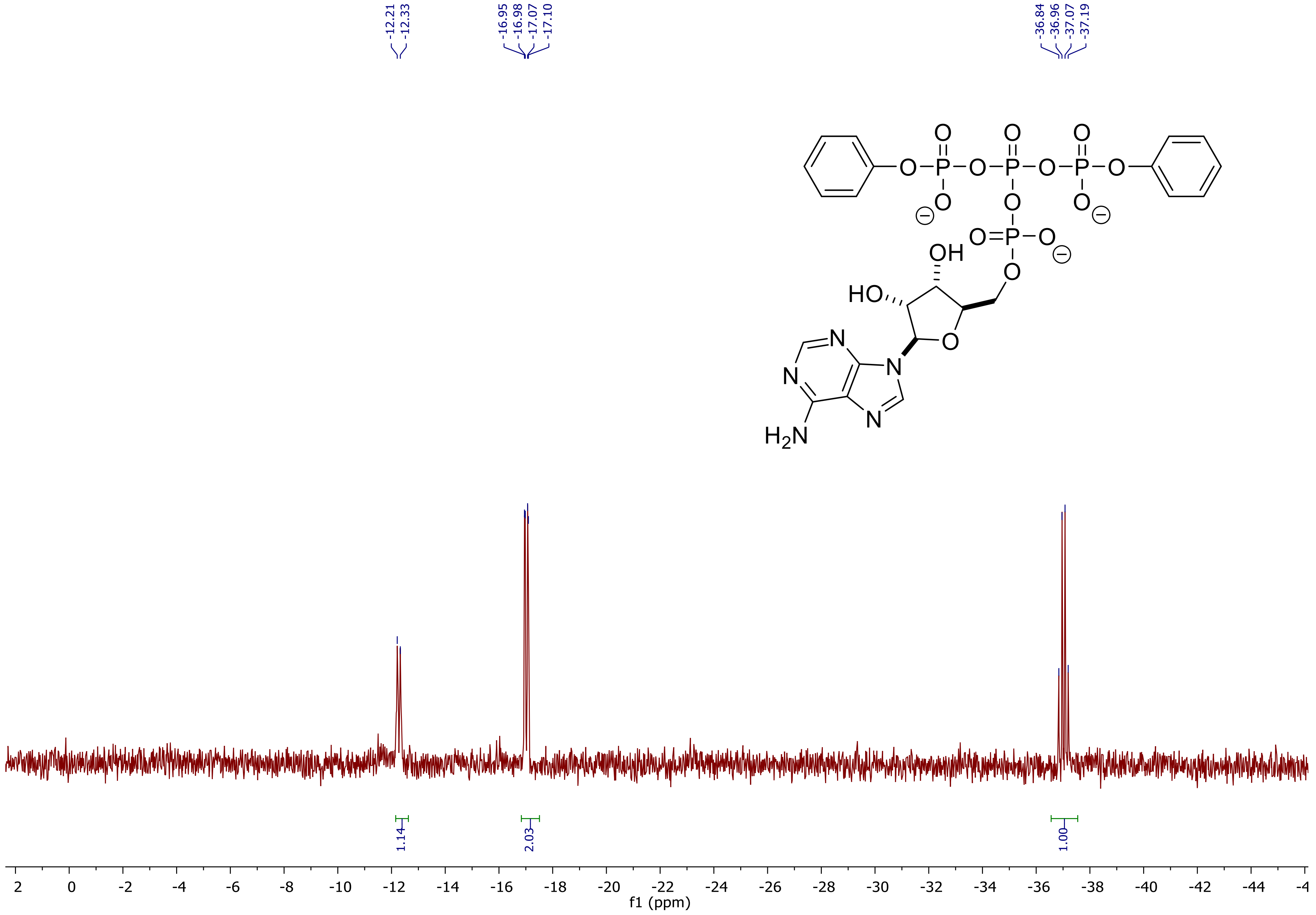

Supplementary Fig. 87| [1]**H-NMR** (400 MHz, D_2O, presat), compound **44**:

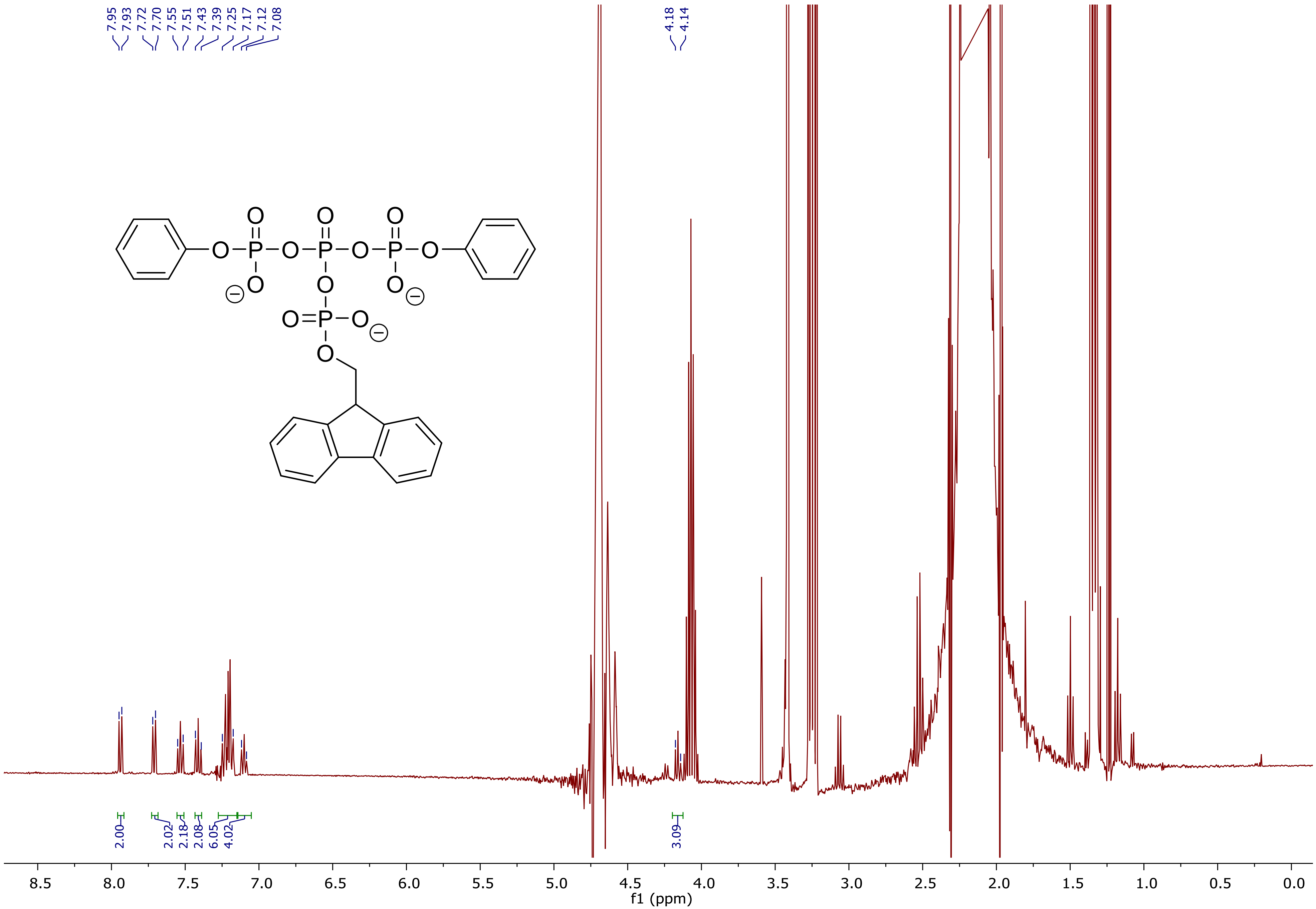

Supplementary Fig. 88│ **^{31}P{^{1}H}-NMR** (162 MHz, D_2O), compound **44**:

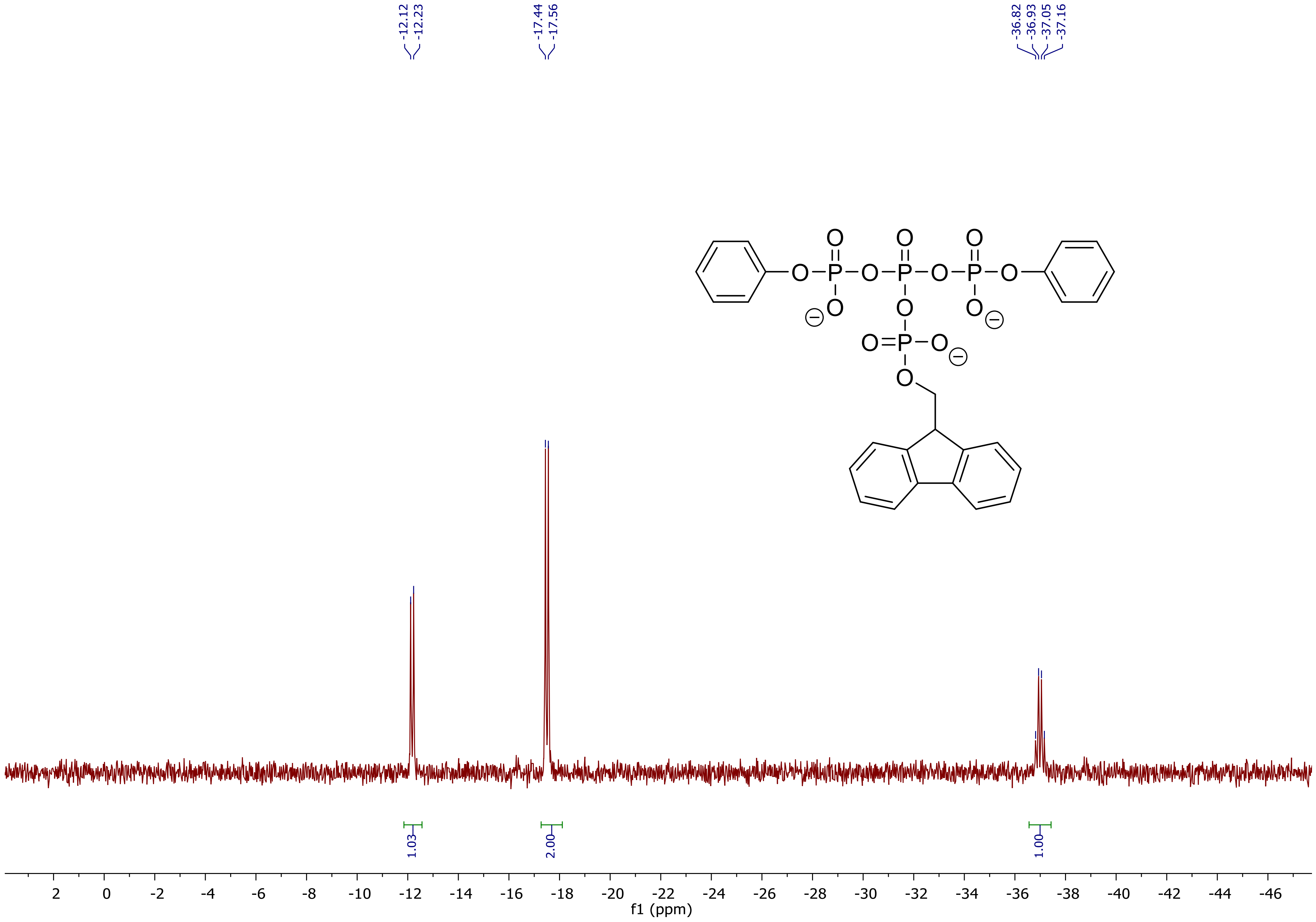

Supplementary Fig. 89│ [31]**P-NMR** (162 MHz, D_2O), compound **44**:

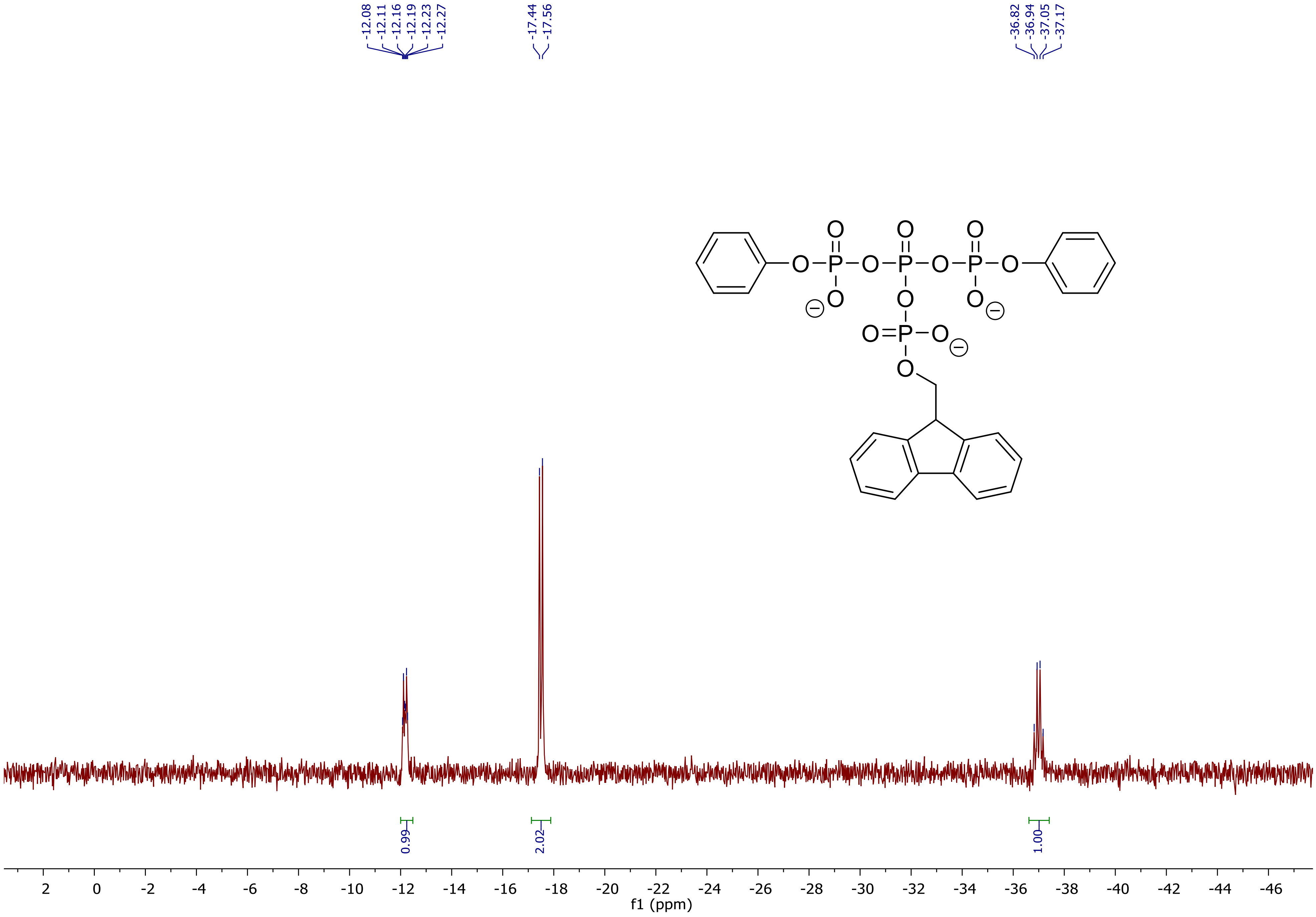

Supplementary Fig. 90| ^{31}**P{^{1}H}-NMR** (162 MHz, D_2O), compound **53**:

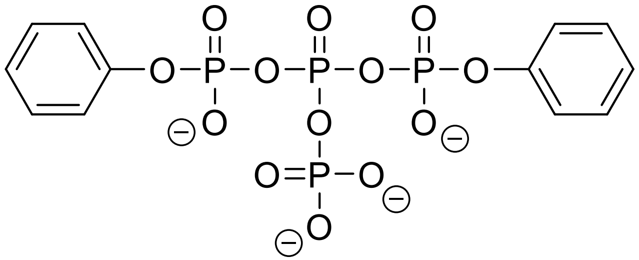

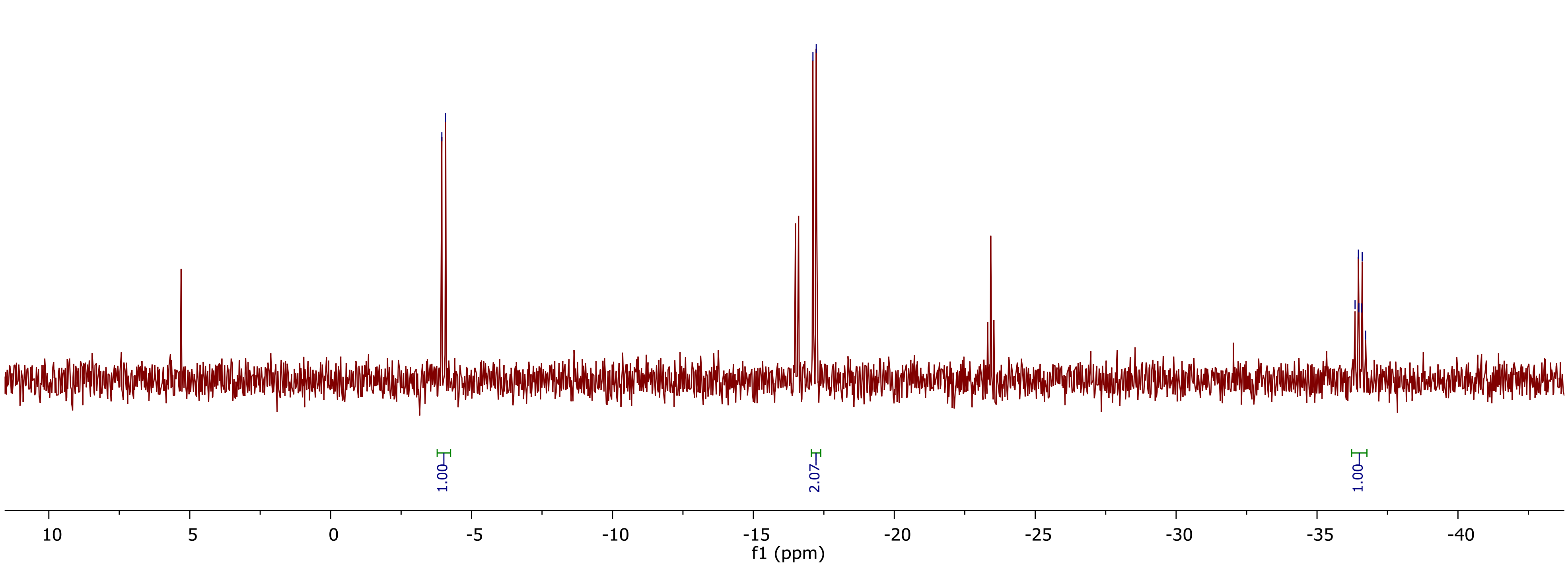

Supplementary Fig. 91| **^{1}H-NMR** (400 MHz, CD_3CN), compound **45**:

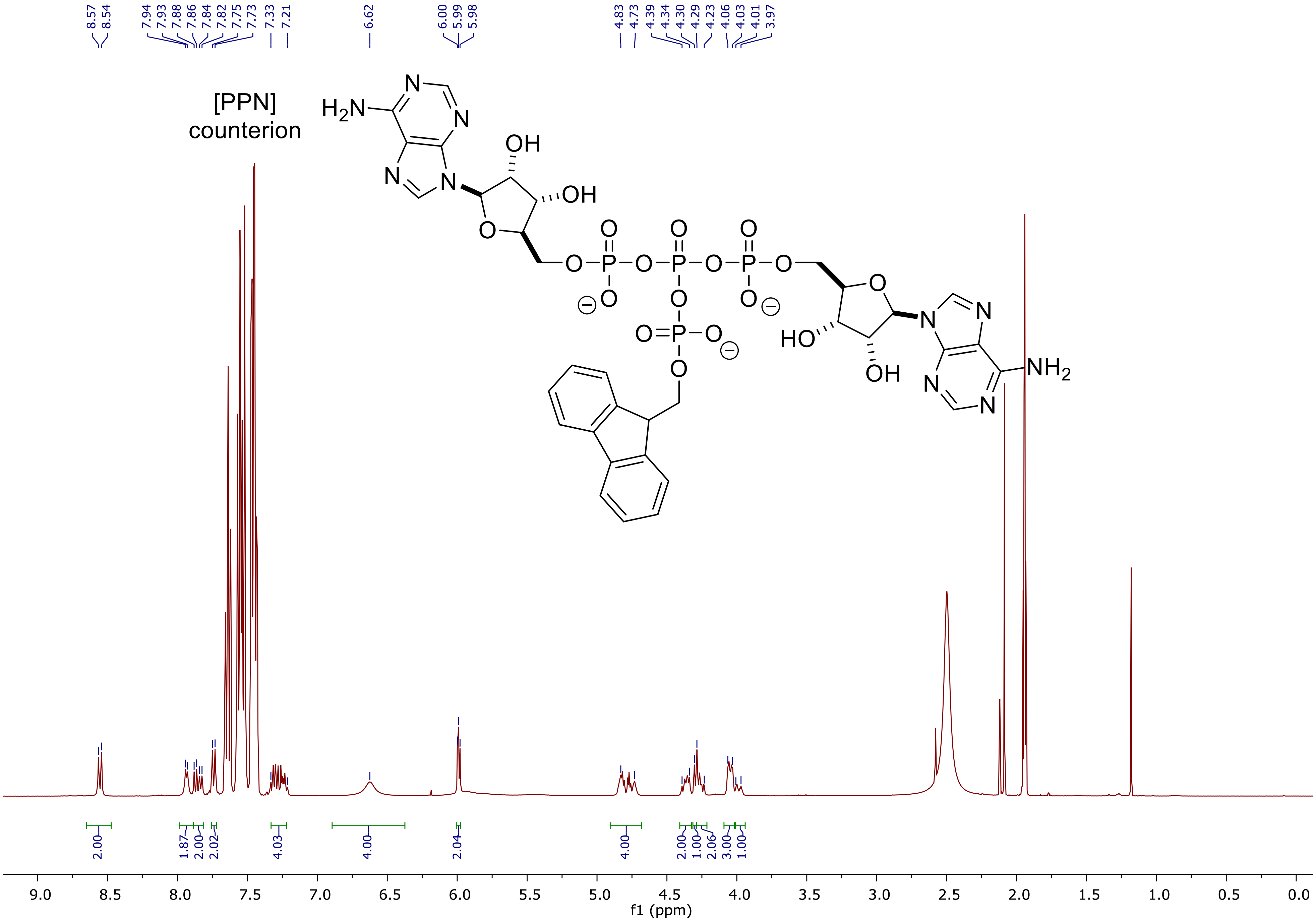

Supplementary Fig. 92 | $^{31}P\{^1H\}$**-NMR** (162 MHz, CD_3CN), compound **45**:

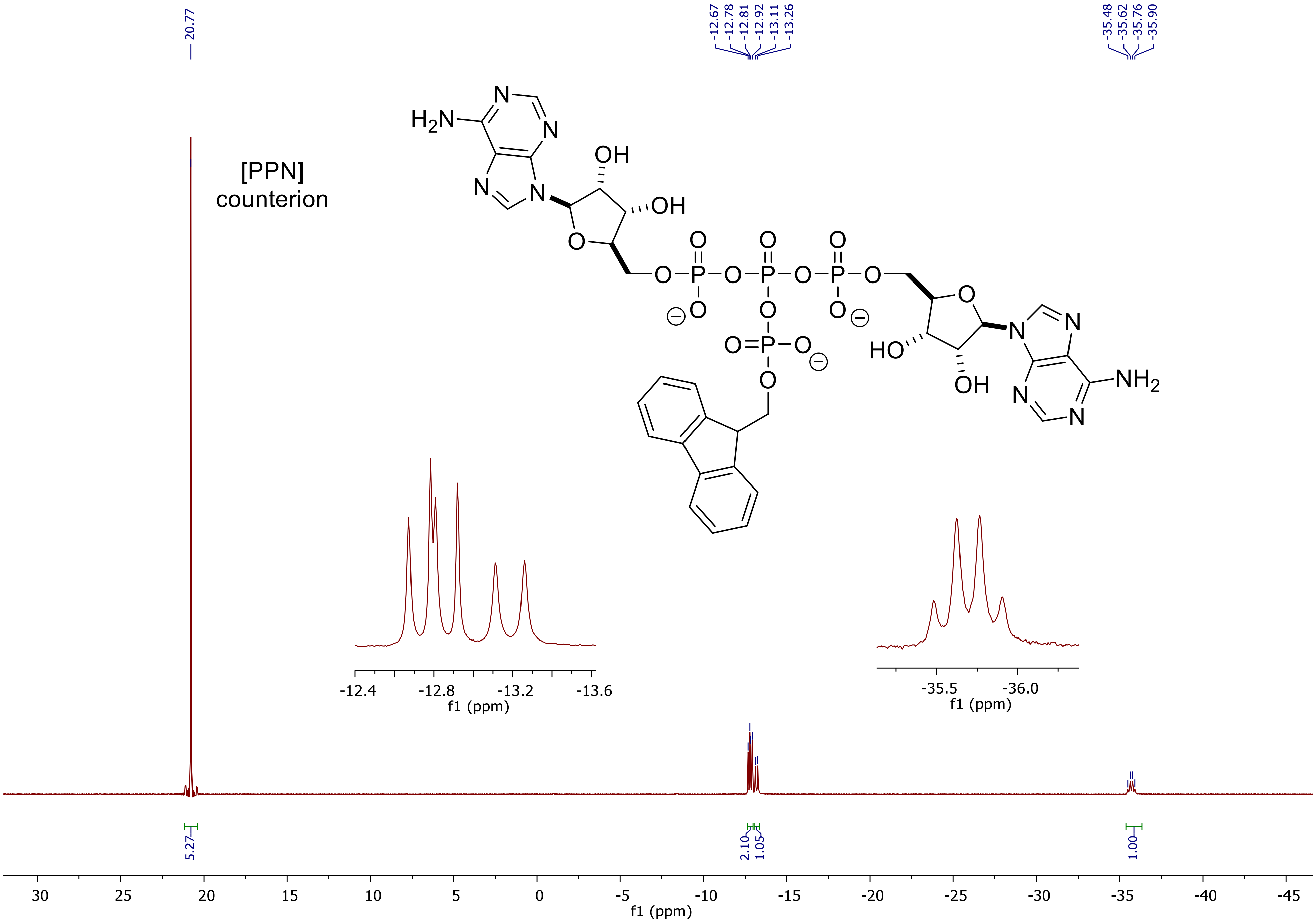

Supplementary Fig. 93 | **^{31}P-NMR** (162 MHz, CD_3CN), compound **45**:

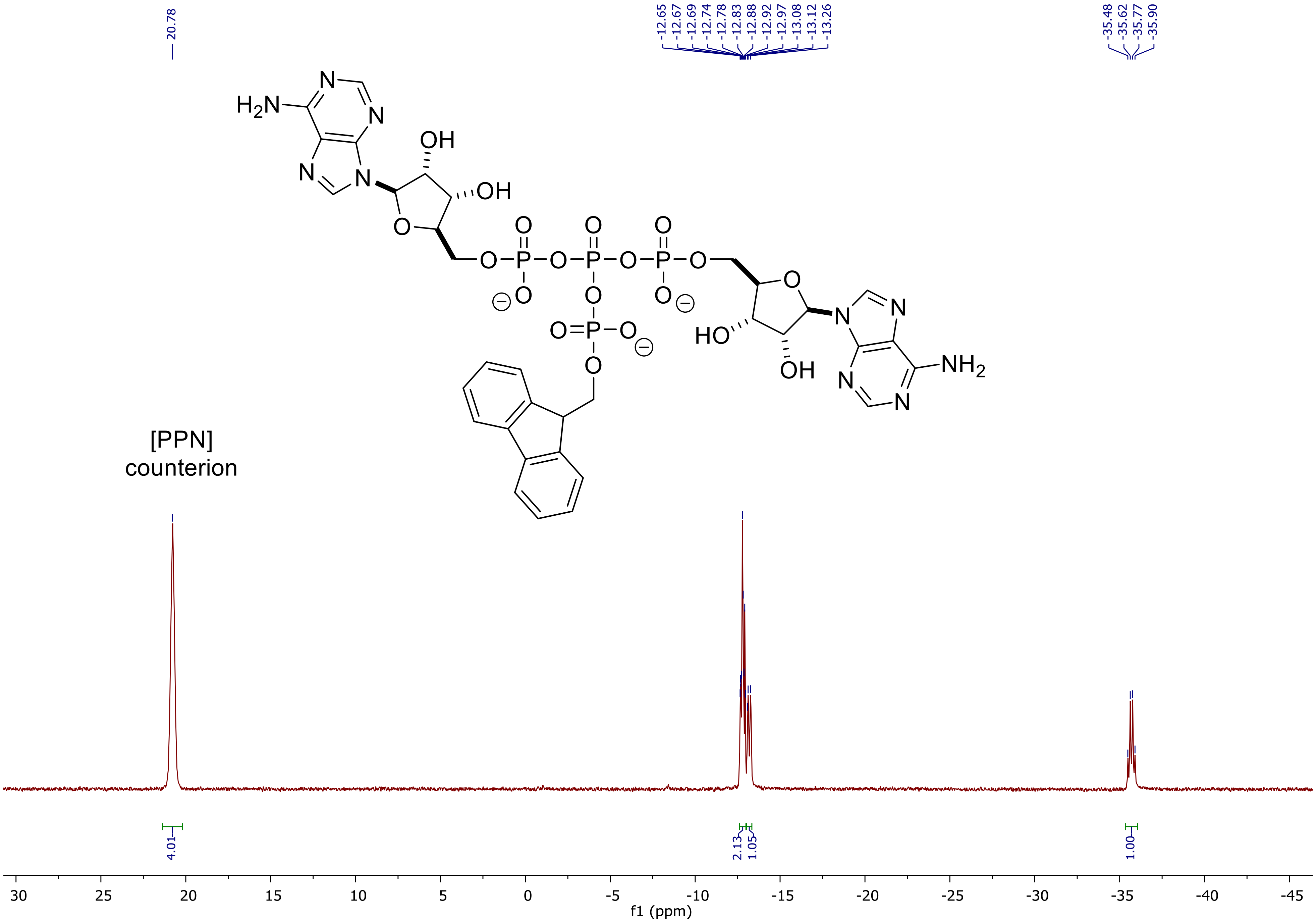

Supplementary Fig. 94 | **^{13}C-NMR** (101 MHz, CD_3CN), compound **45**:

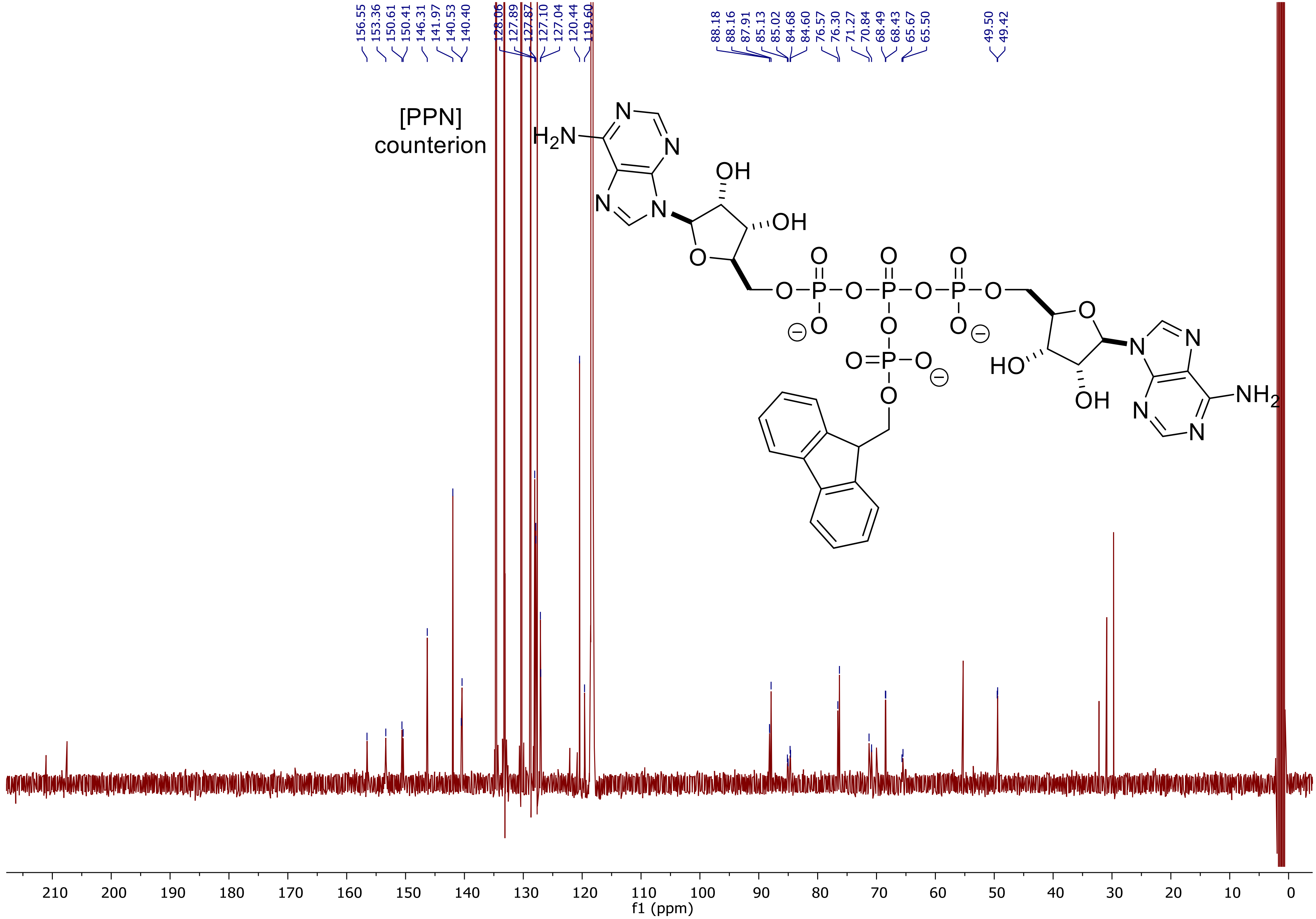

Supplementary Fig. 95 | **^{1}H-^{31}P-HMBC** (CD_3CN), compound **45**:

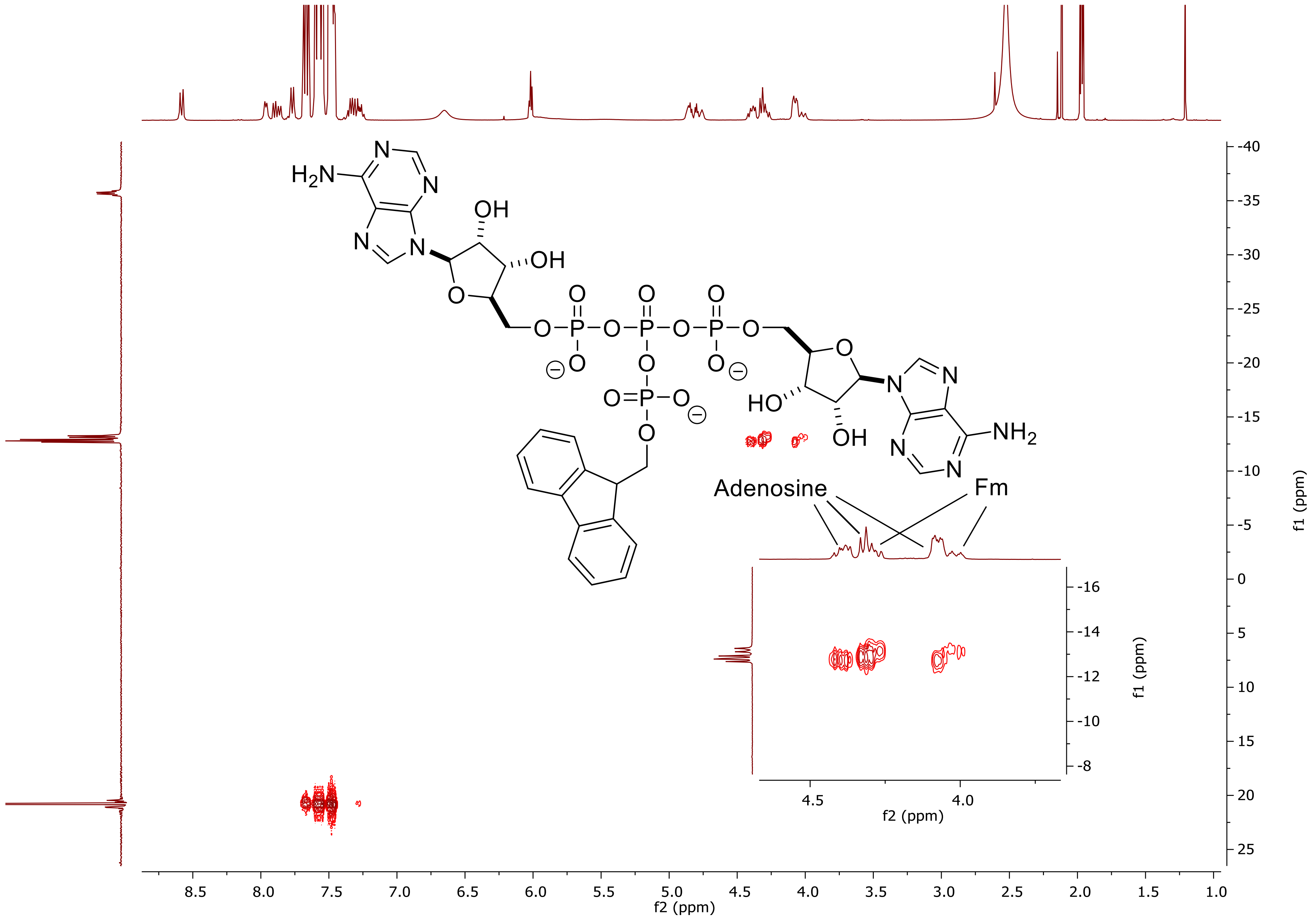

Supplementary Fig. 96│ **DQF-COSY** (CD_3CN), compound **45**:

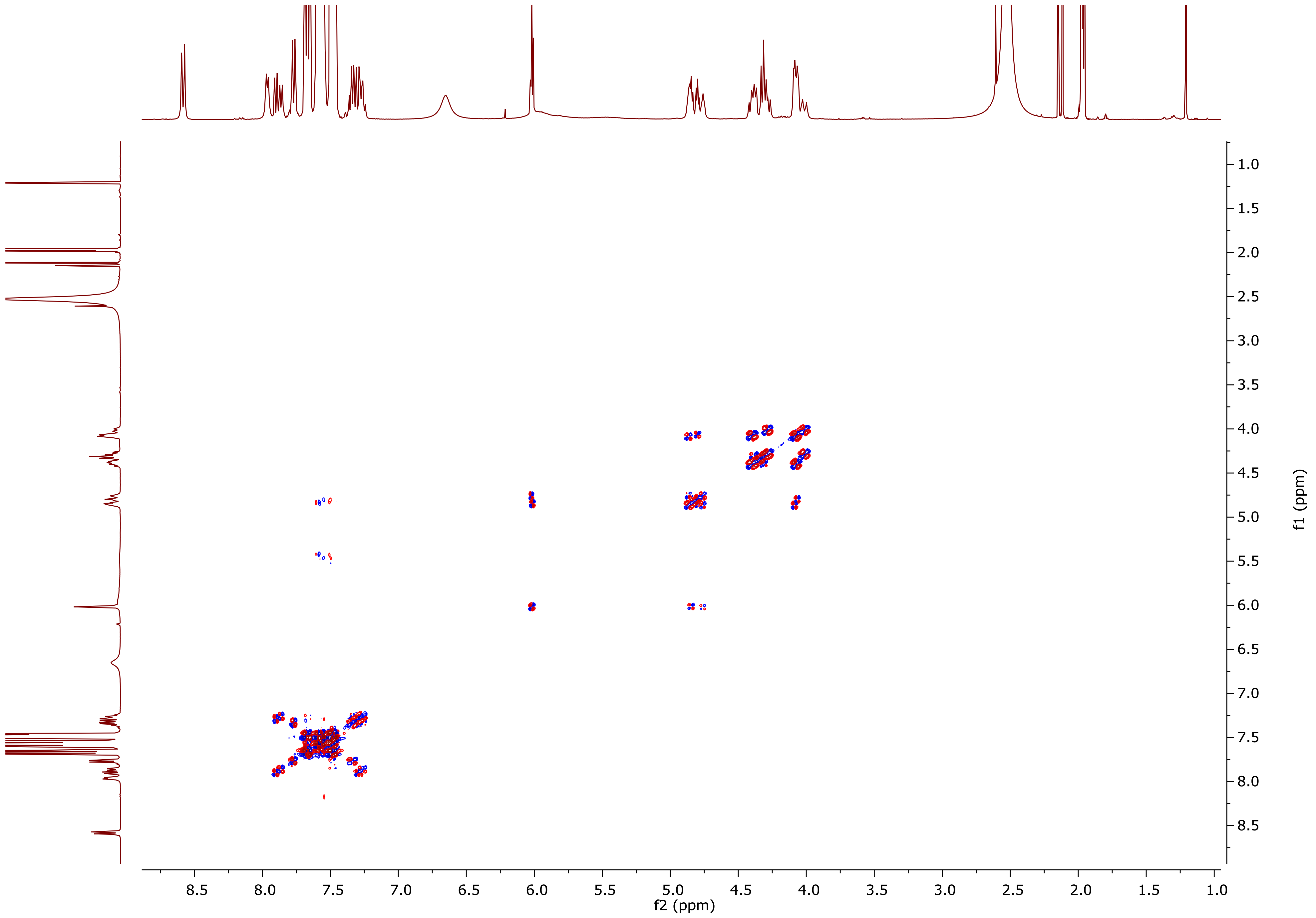

Supplementary Fig. 97 | **edHSQC** (CD_3CN), compound **45**:

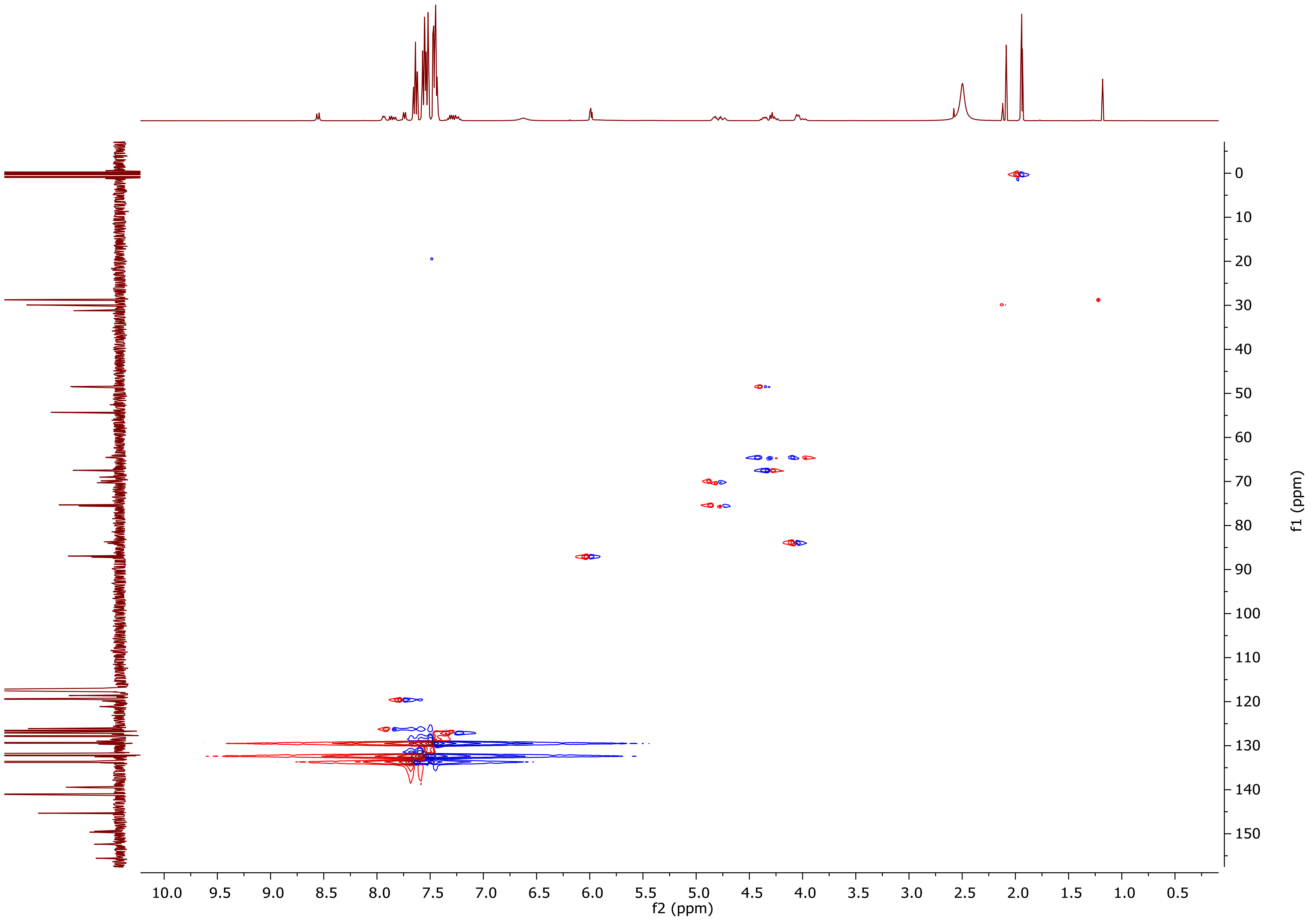

Supplementary Fig. 98│ **HMBC** (CD_3CN), compound **45**:

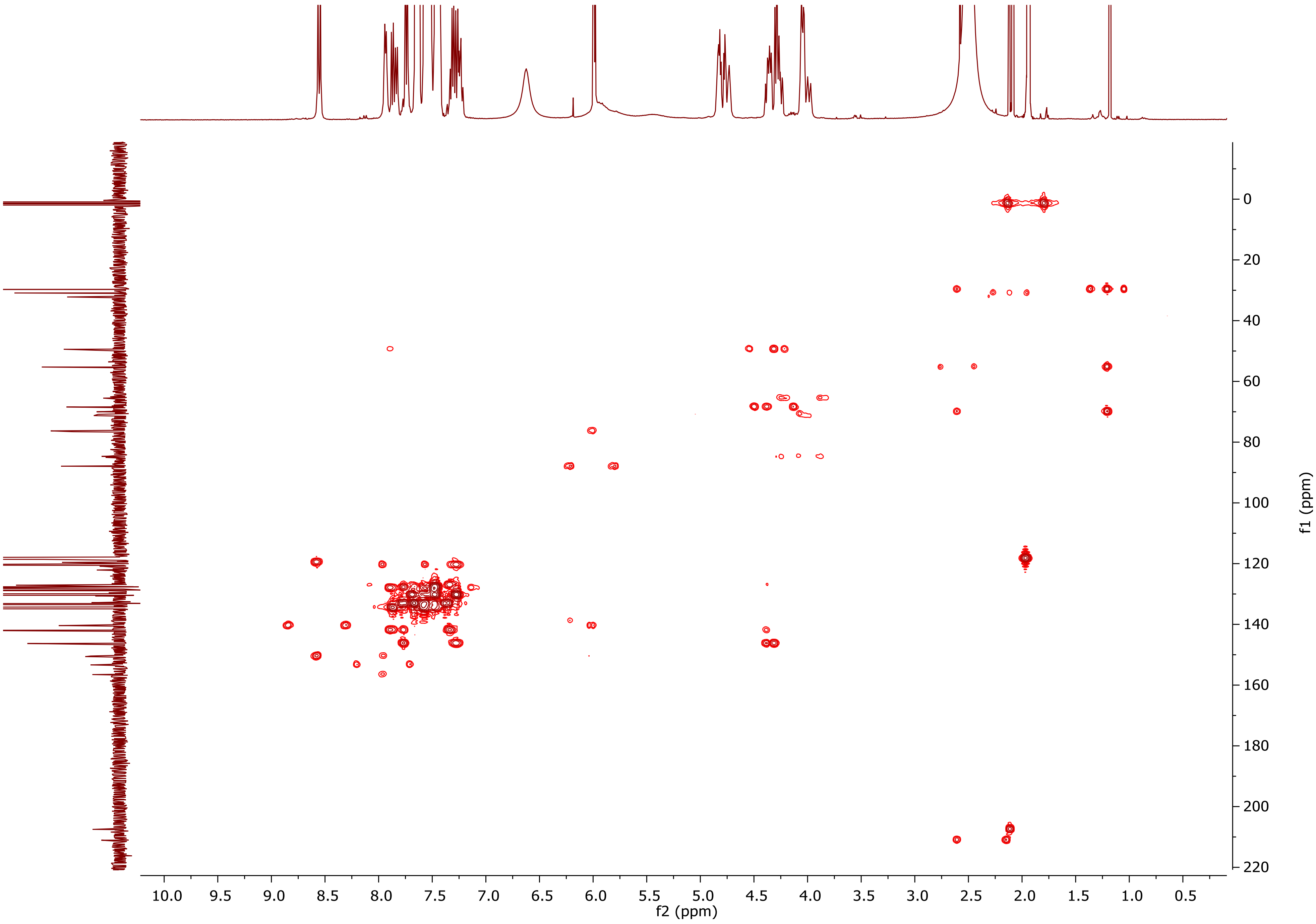

Supplementary Fig. 99| $^{31}P\{^{1}H\}$**-NMR** (162 MHz, D_2O), compound **54**:

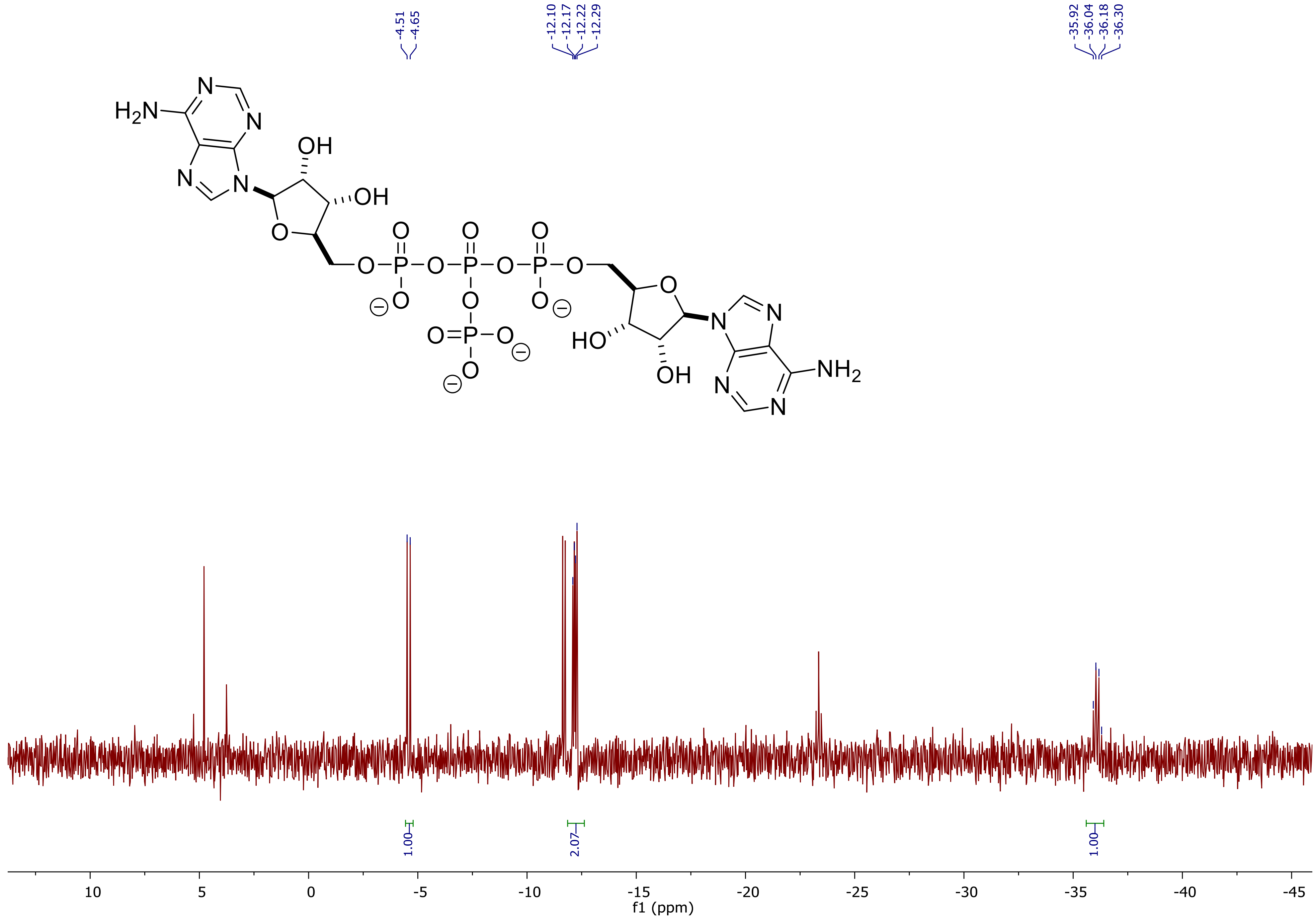

Supplementary Fig. 100 | **^{31}P-NMR** (162 MHz, D_2O), compound **54**:

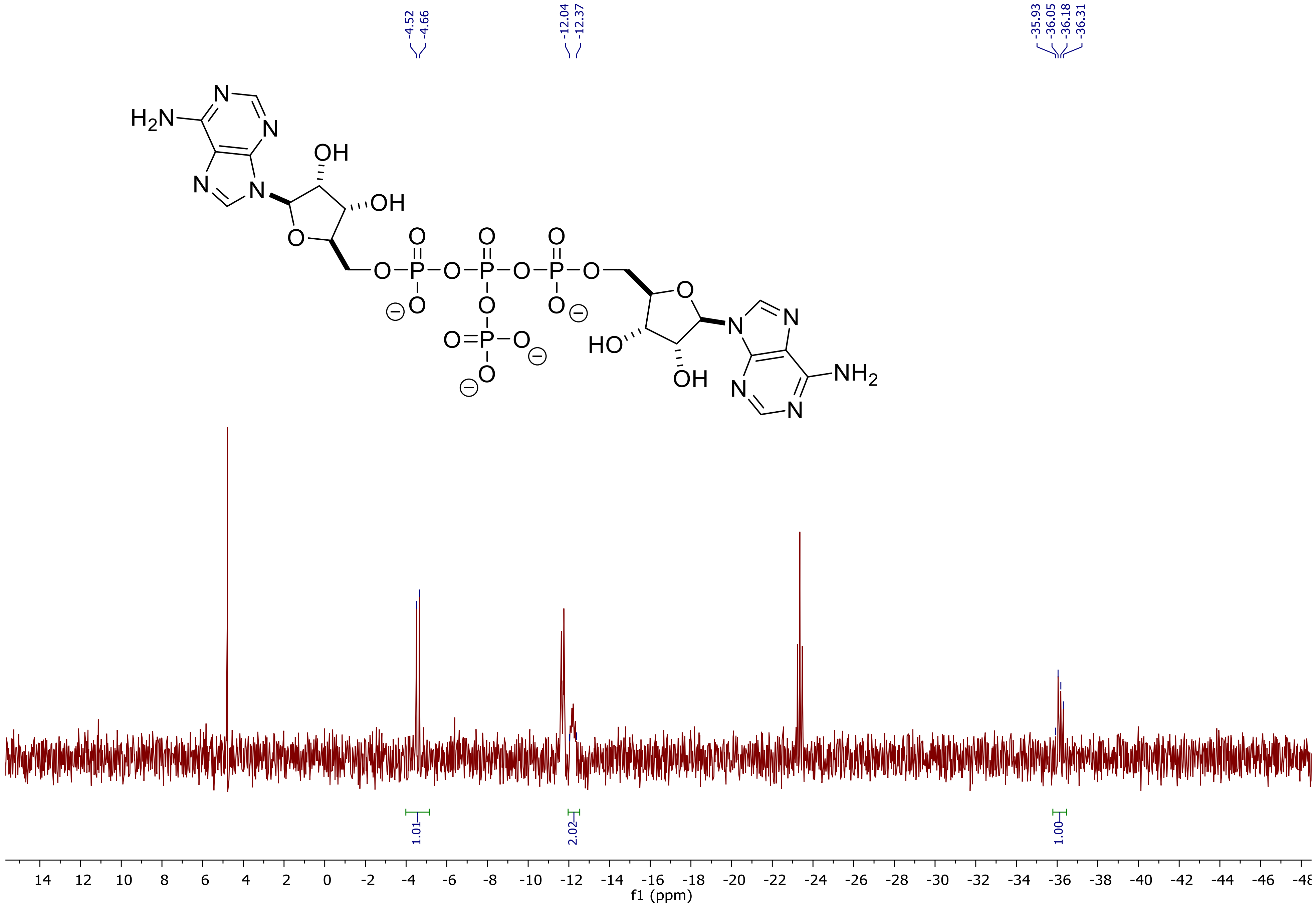

Supplementary Fig. 101 | **^{1}H-NMR** (400 MHz, MeCN, presat), compound **46**:

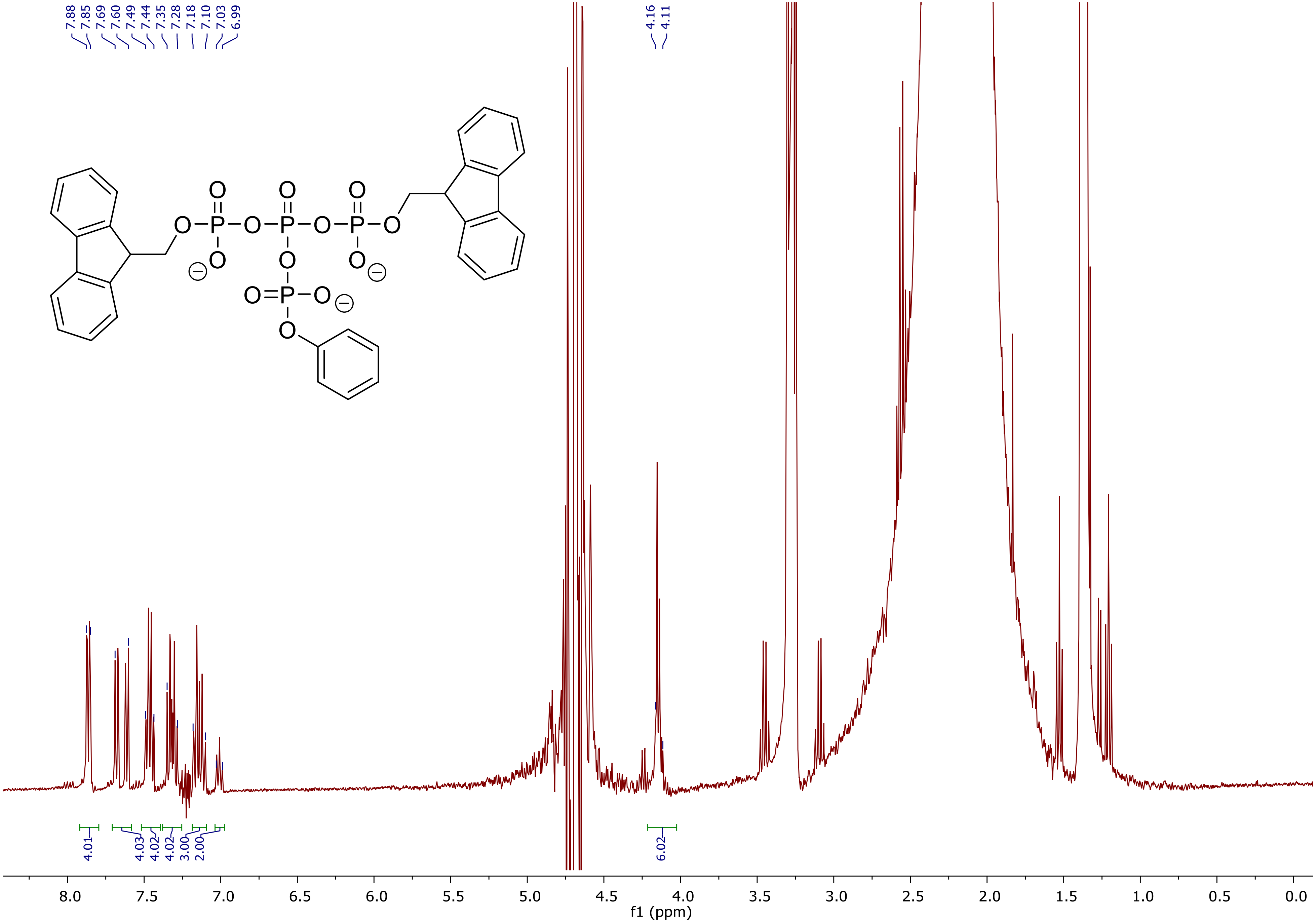

Supplementary Fig. 102 | **^{31}P{^{1}H}-NMR** (162 MHz, MeCN), compound **46**:

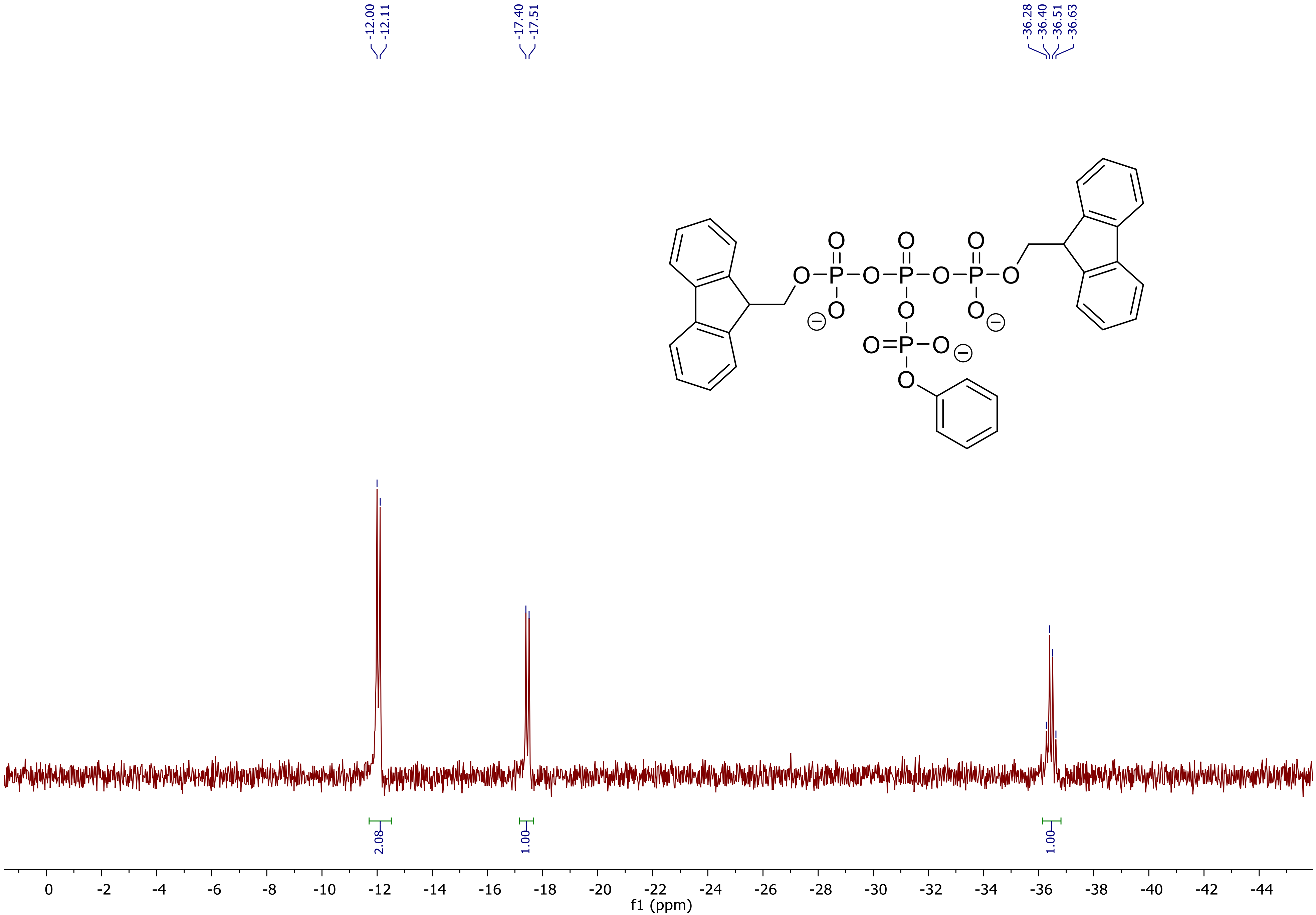

Supplementary Fig. 103 | **^{31}P-NMR** (162 MHz, MeCN), compound **46**:

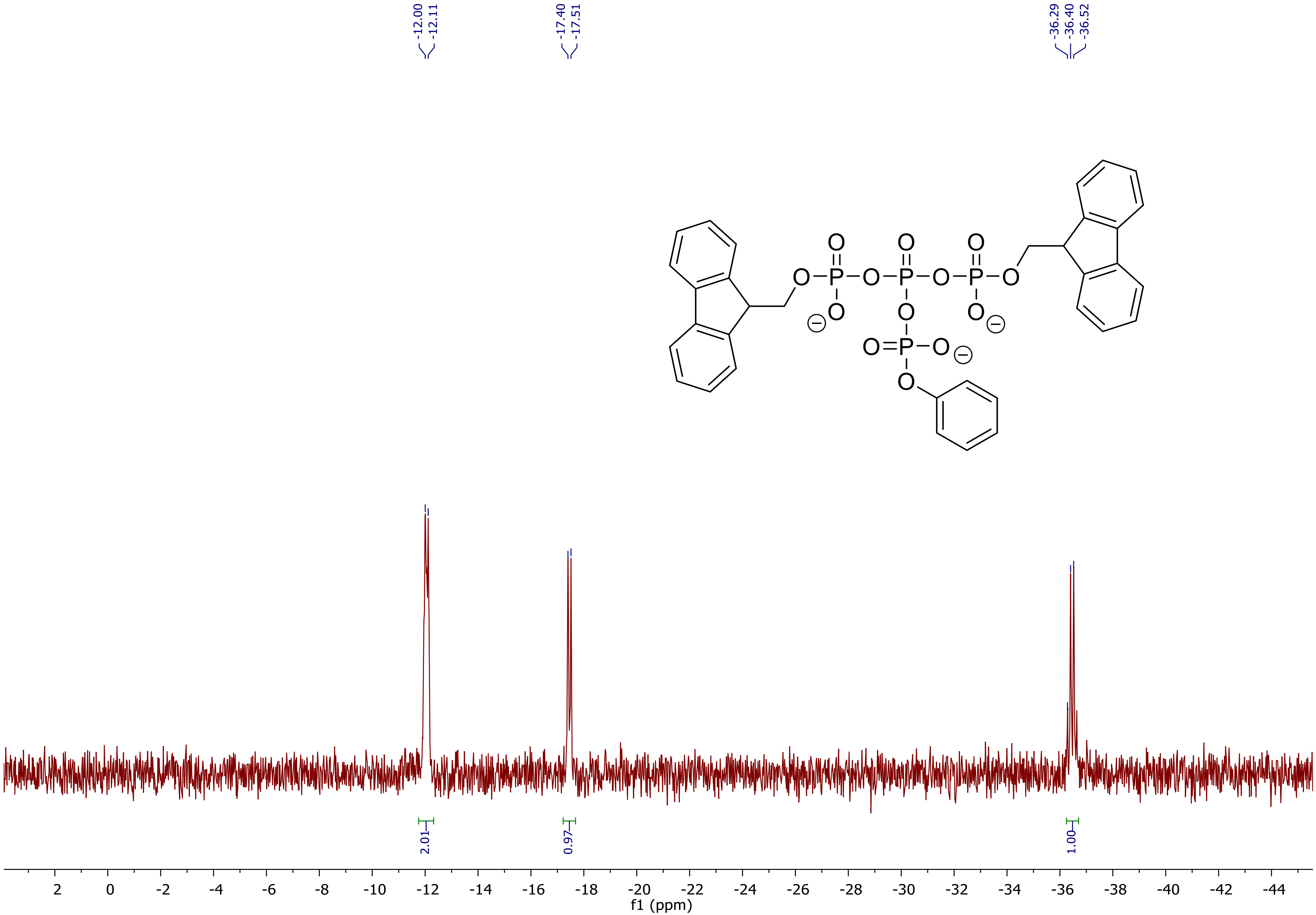

Supplementary Fig. 104 | $^{31}P\{^{1}H\}$**-NMR** (162 MHz, D_2O), compound **50**:

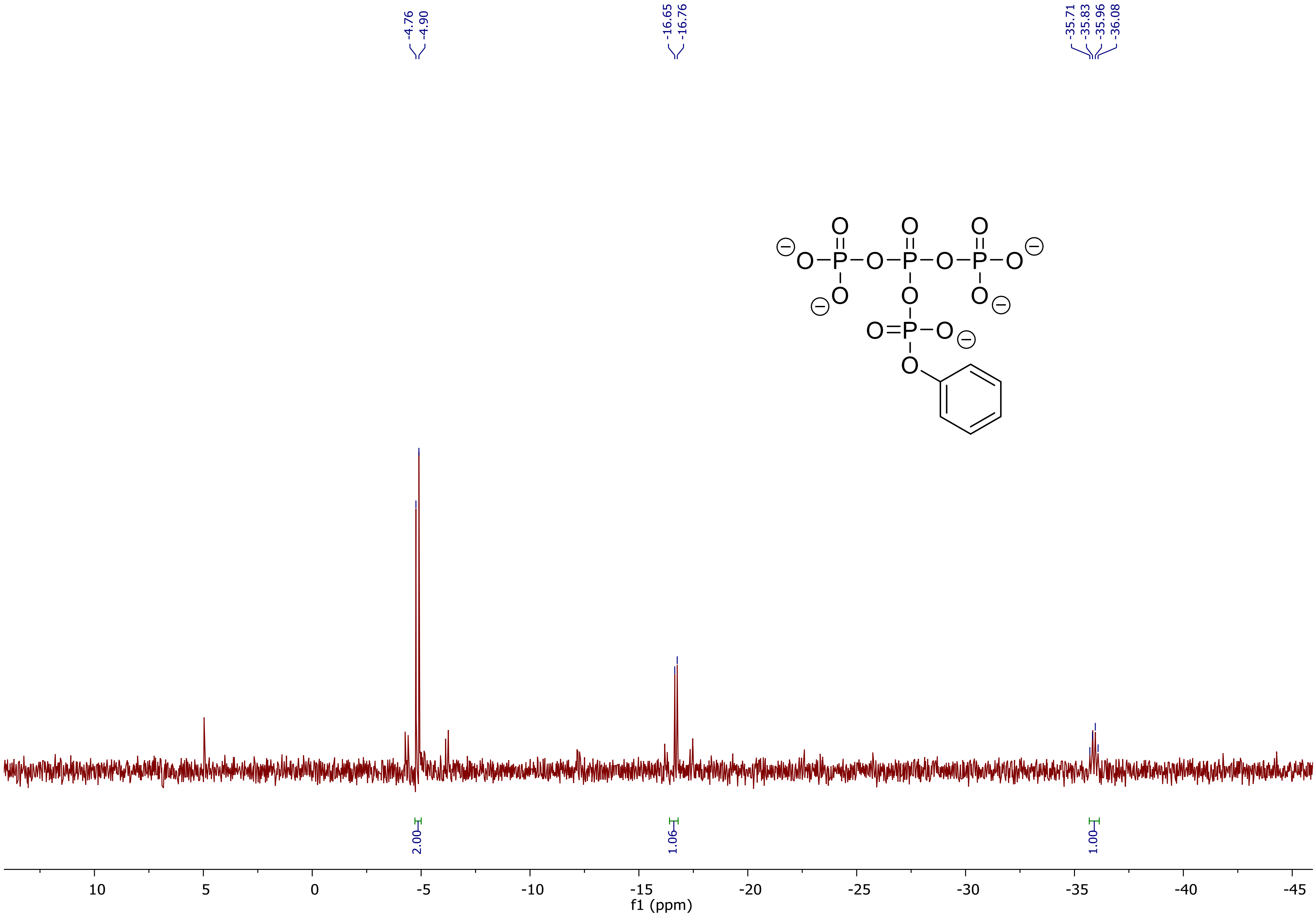

Supplementary Fig. 105 | **^{1}H-NMR** (400 MHz, D_2O, presat), compound **47**:

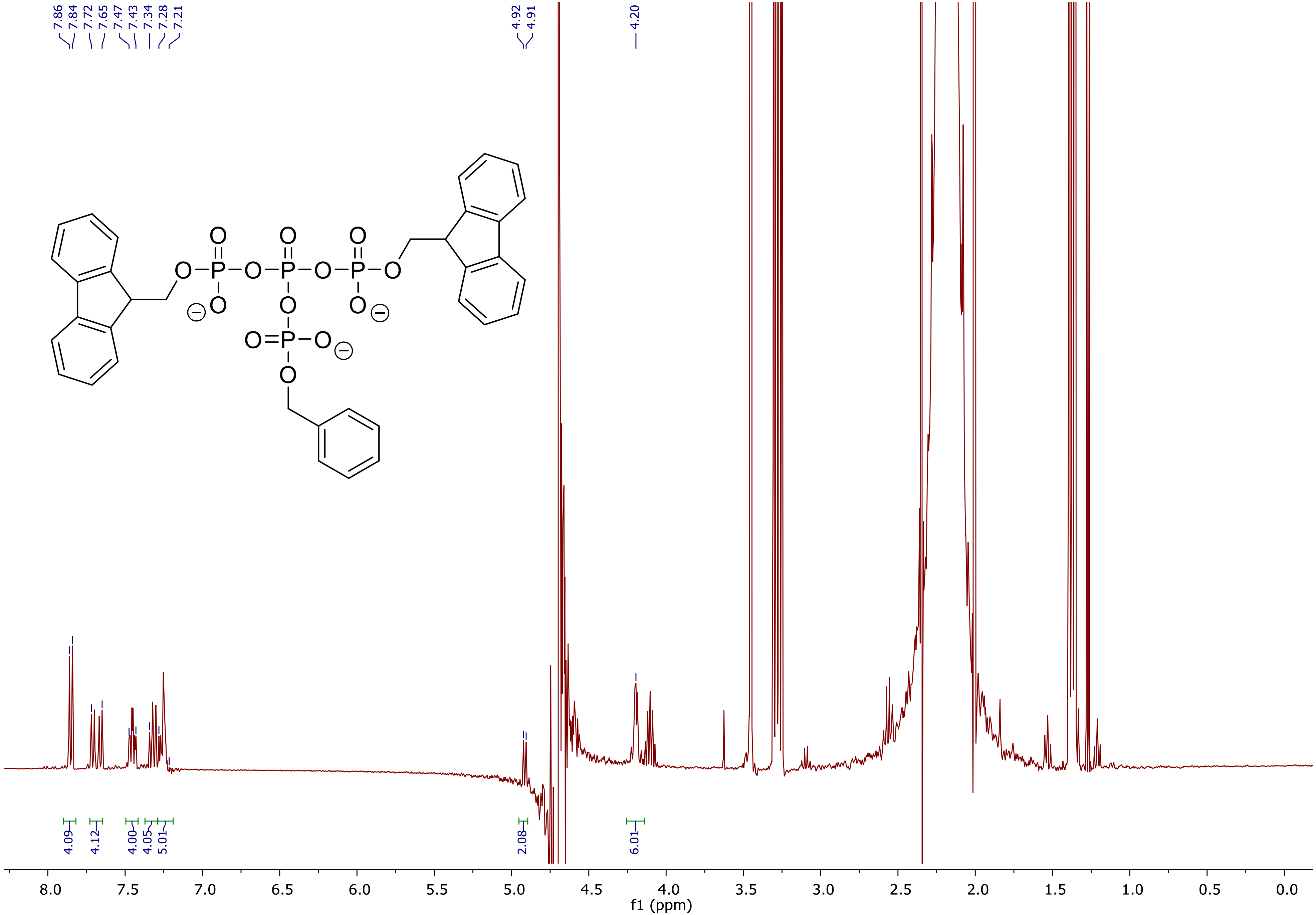

Supplementary Fig. 106│ **^{31}P{^{1}H}-NMR** (162 MHz, D_2O), compound **47**:

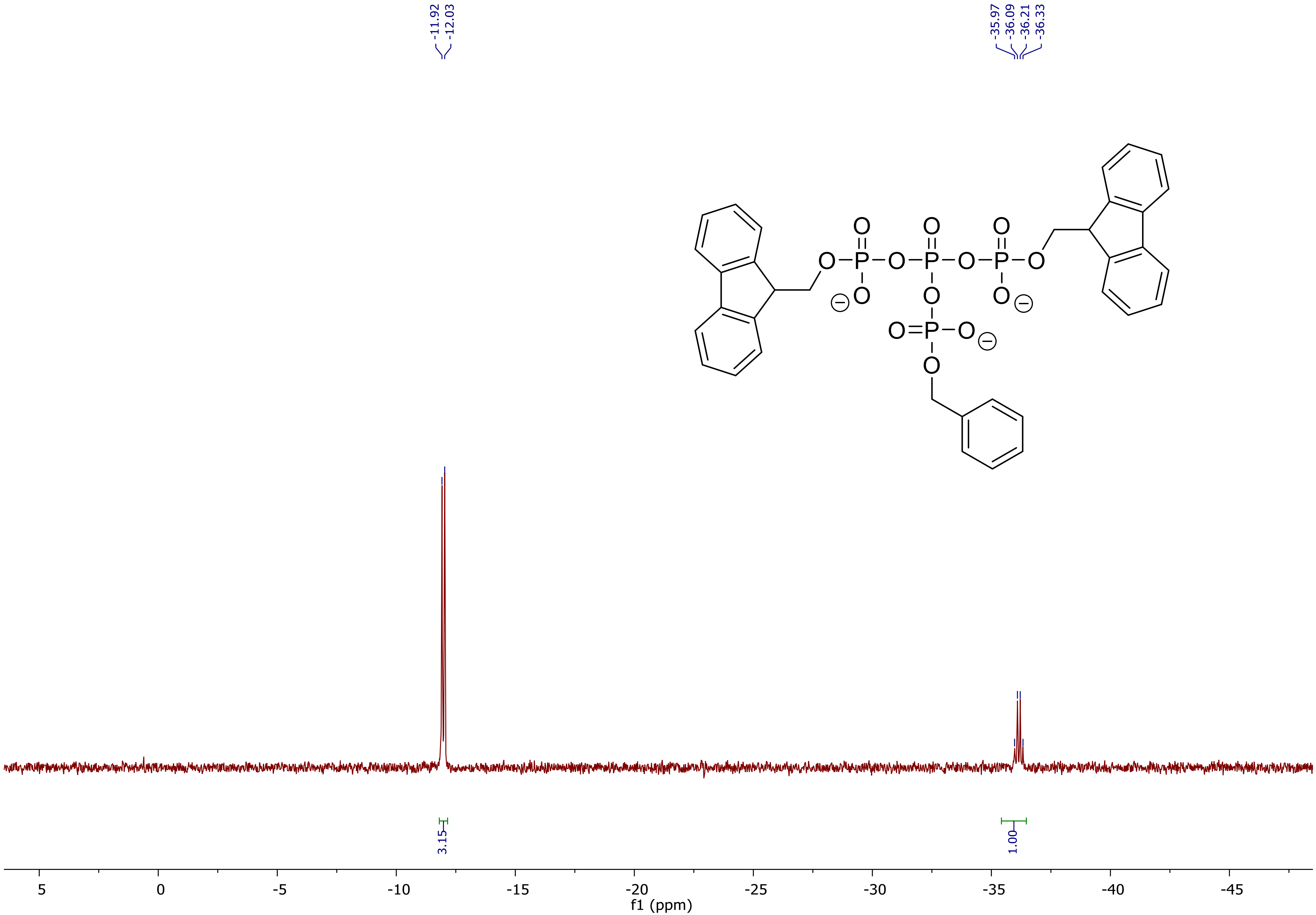

Supplementary Fig. 107 | **^{31}P-NMR** (162 MHz, D_2O), compound **47**:

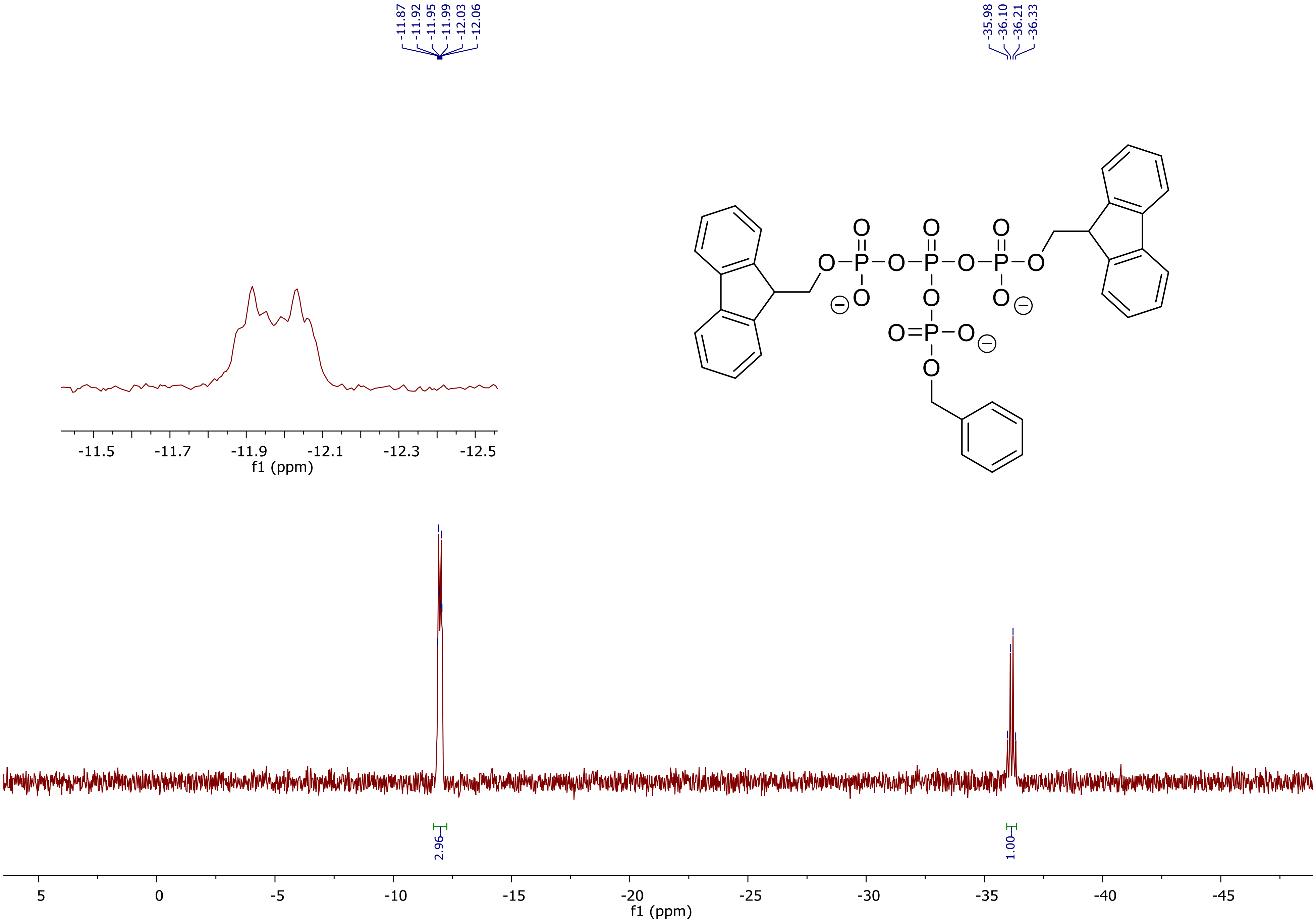

Supplementary Fig. 108| **1H-31P-HMBC** (D_2O), compound **47**:

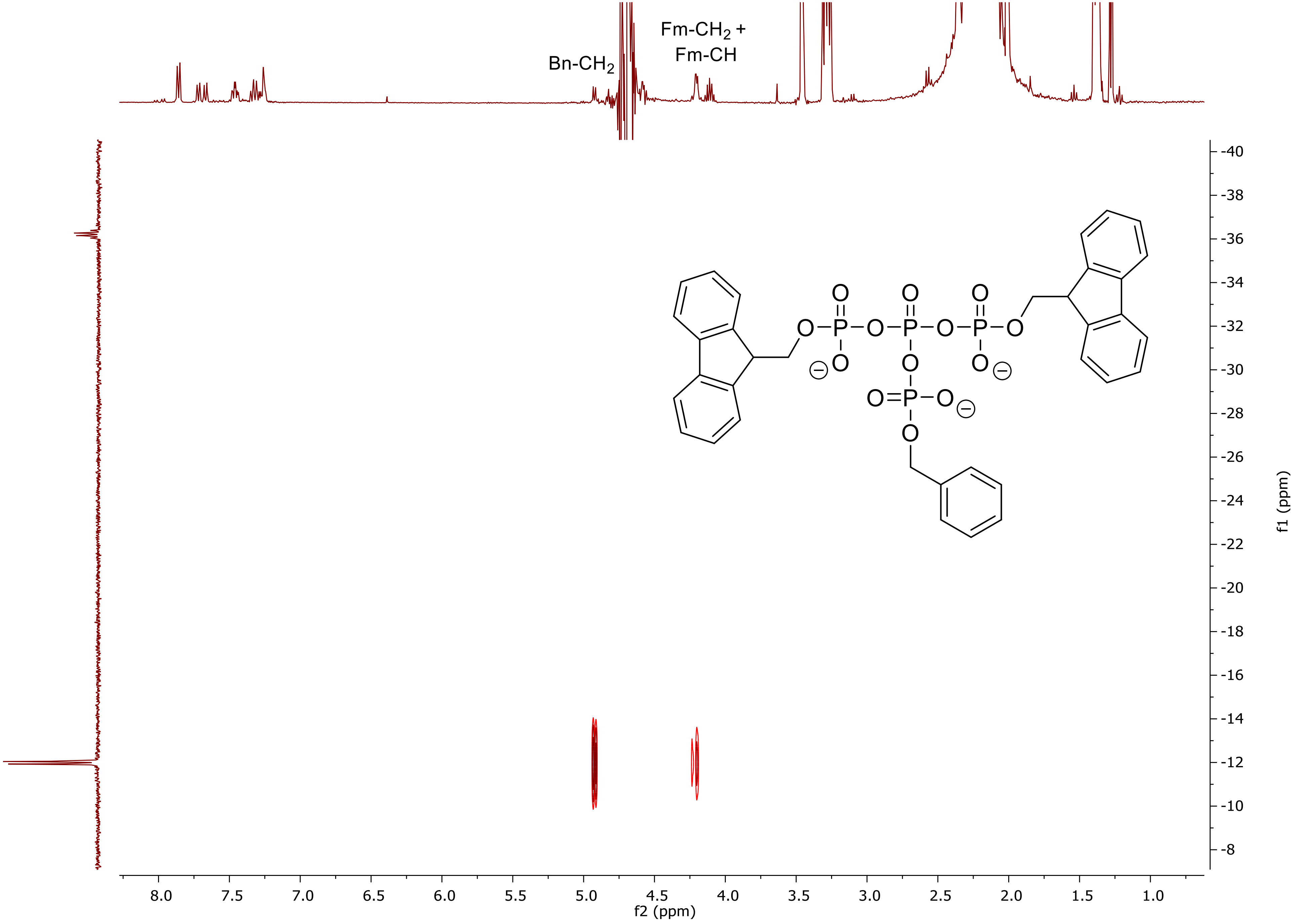

Supplementary Fig. 109| **$^{31}P\{^{1}H\}$-NMR** (162 MHz, D_2O), compound **51**:

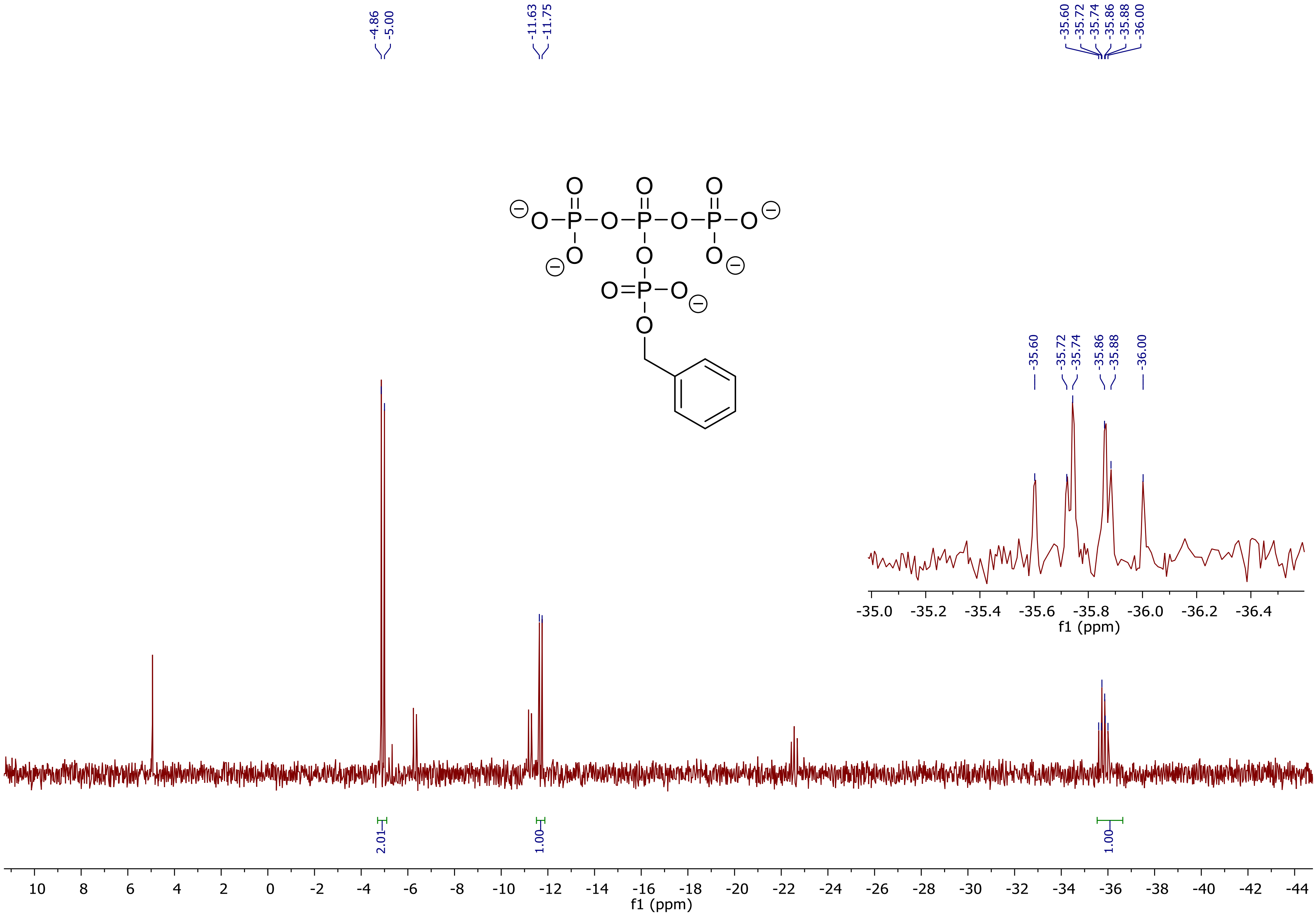

Supplementary Fig. 110│ **^{1}H-NMR** (400 MHz, D_2O, presat), compound **48**:

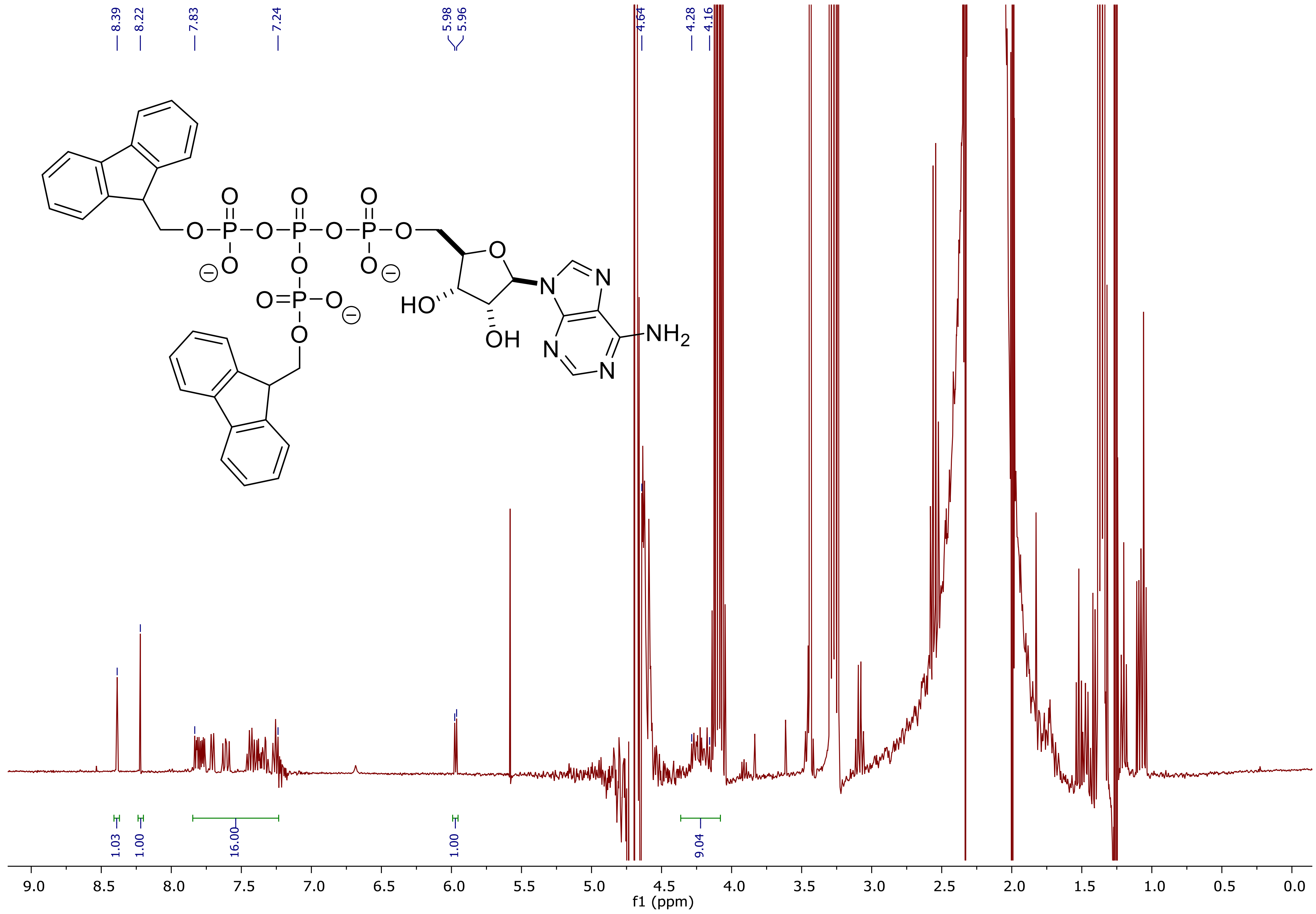

Supplementary Fig. 111 | **$^{31}P\{^1H\}$-NMR** (162 MHz, D_2O), compound **48**:

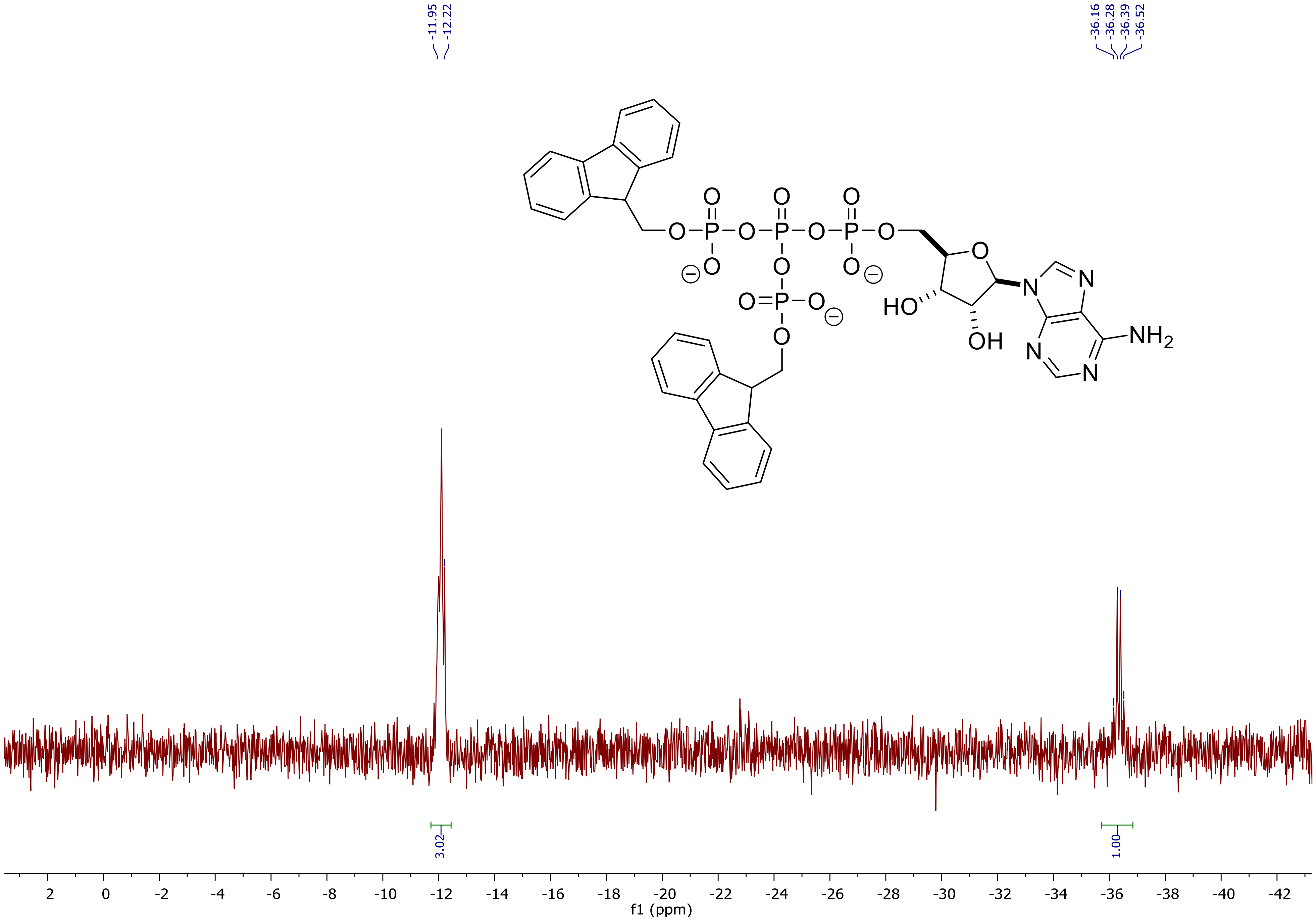

Supplementary Fig. 112| **^{31}P-NMR** (162 MHz, D_2O), compound **48**:

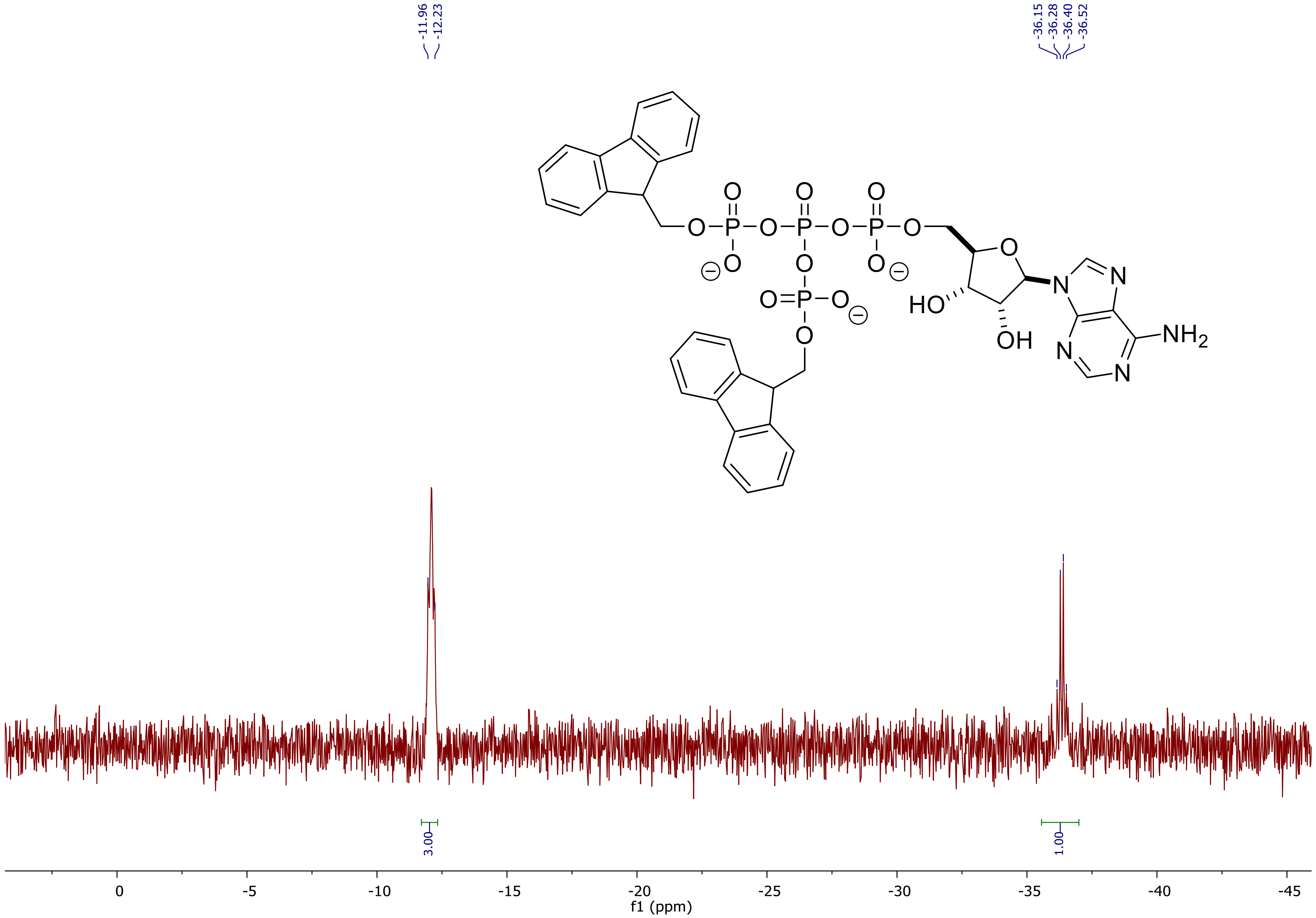

Supplementary Fig. 113 | **^{1}H-^{31}P-HMBC** (D_2O), compound **48**:

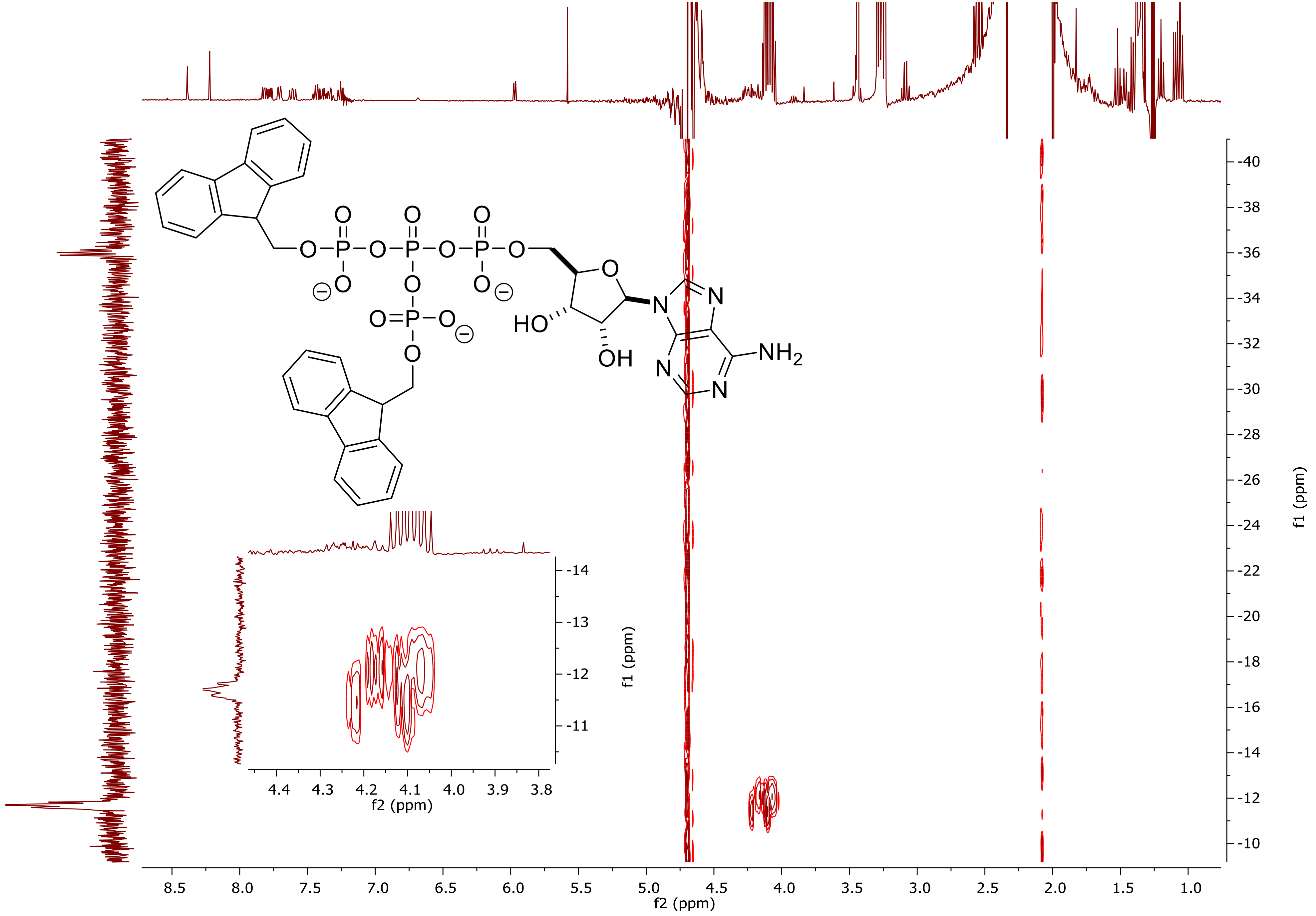

Supplementary Fig. 114 | **^{31}P{^{1}H}-NMR** (162 MHz, D_2O), compound **52**:

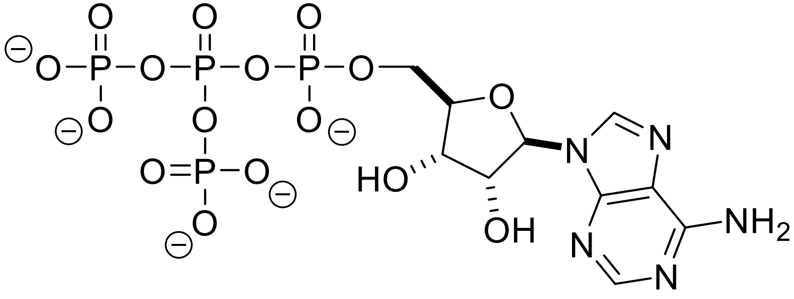

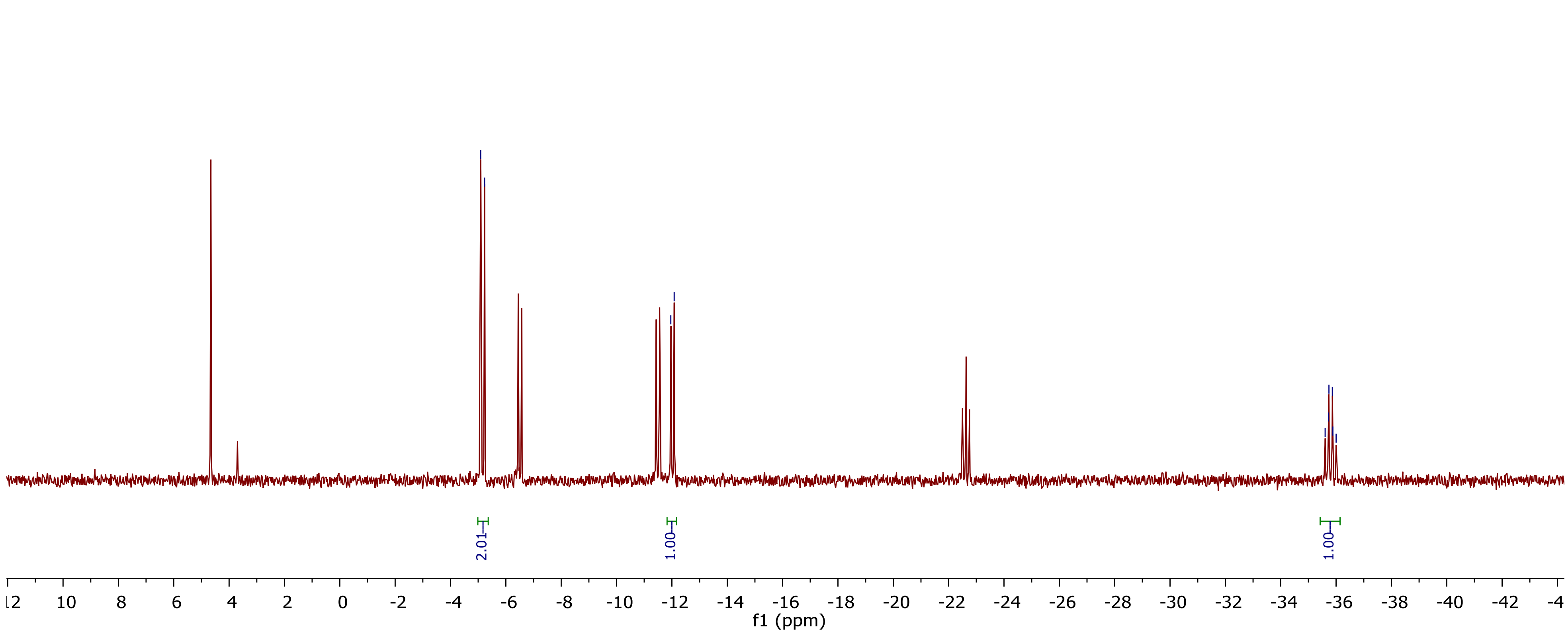

Supplementary Fig. 115 | **^{31}P-NMR** (162 MHz, D_2O), compound **52**:

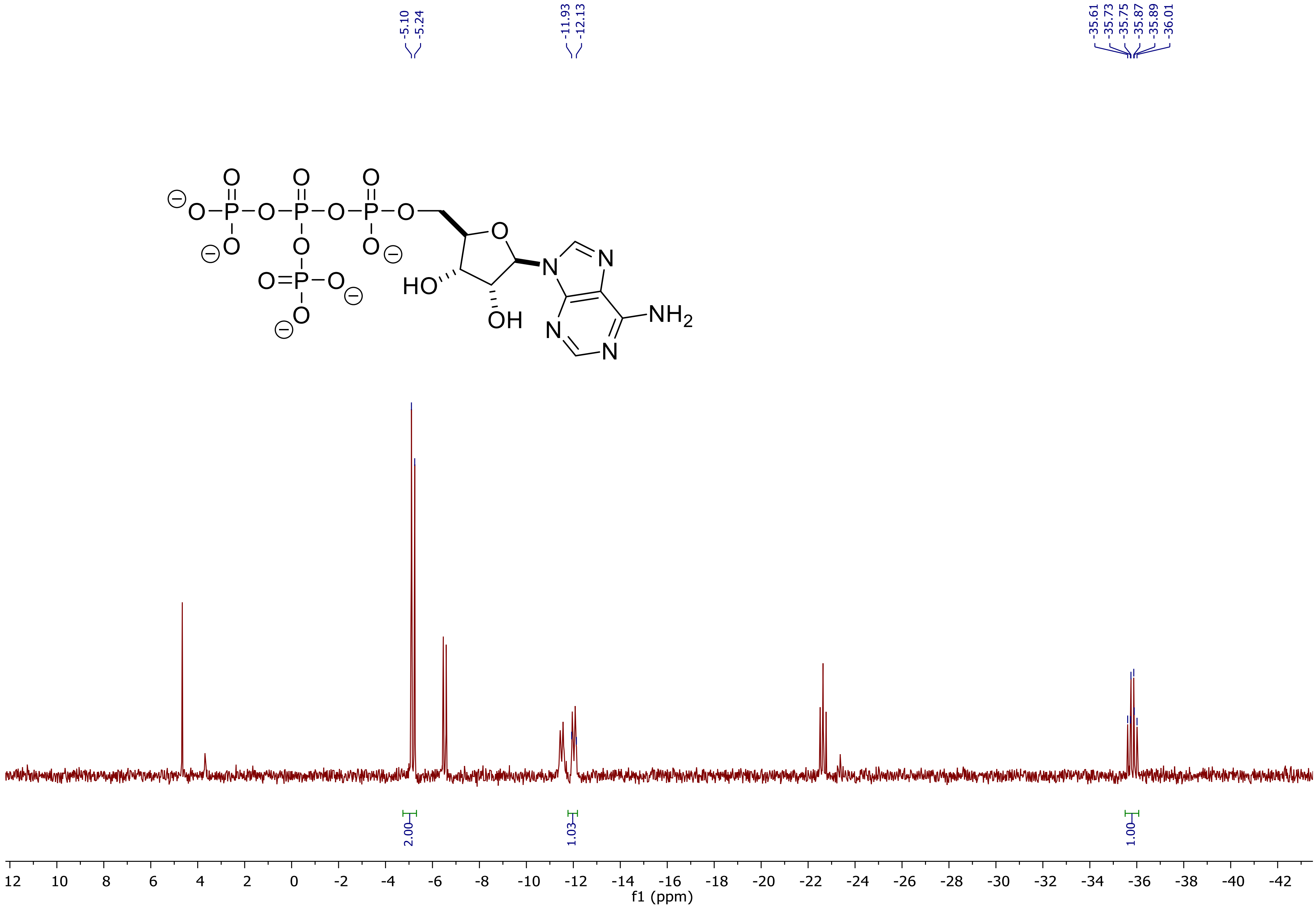

Supplementary Fig. 116 | **^{1}H-NMR** (400 MHz, D_2O, presat), compound **49**:

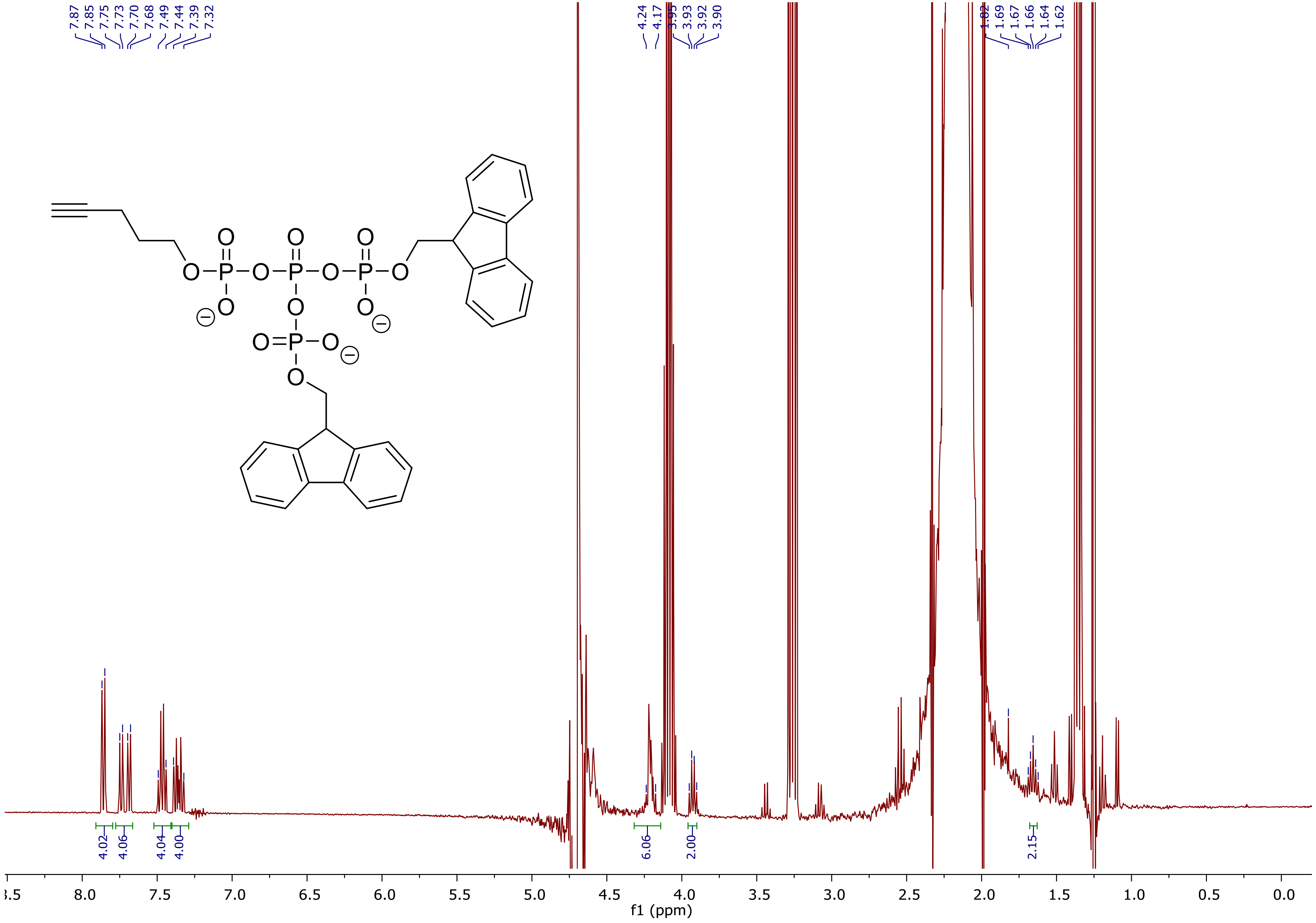

Supplementary Fig. 117 | **$^{31}P\{^{1}H\}$-NMR** (162 MHz, D_2O), compound **49**:

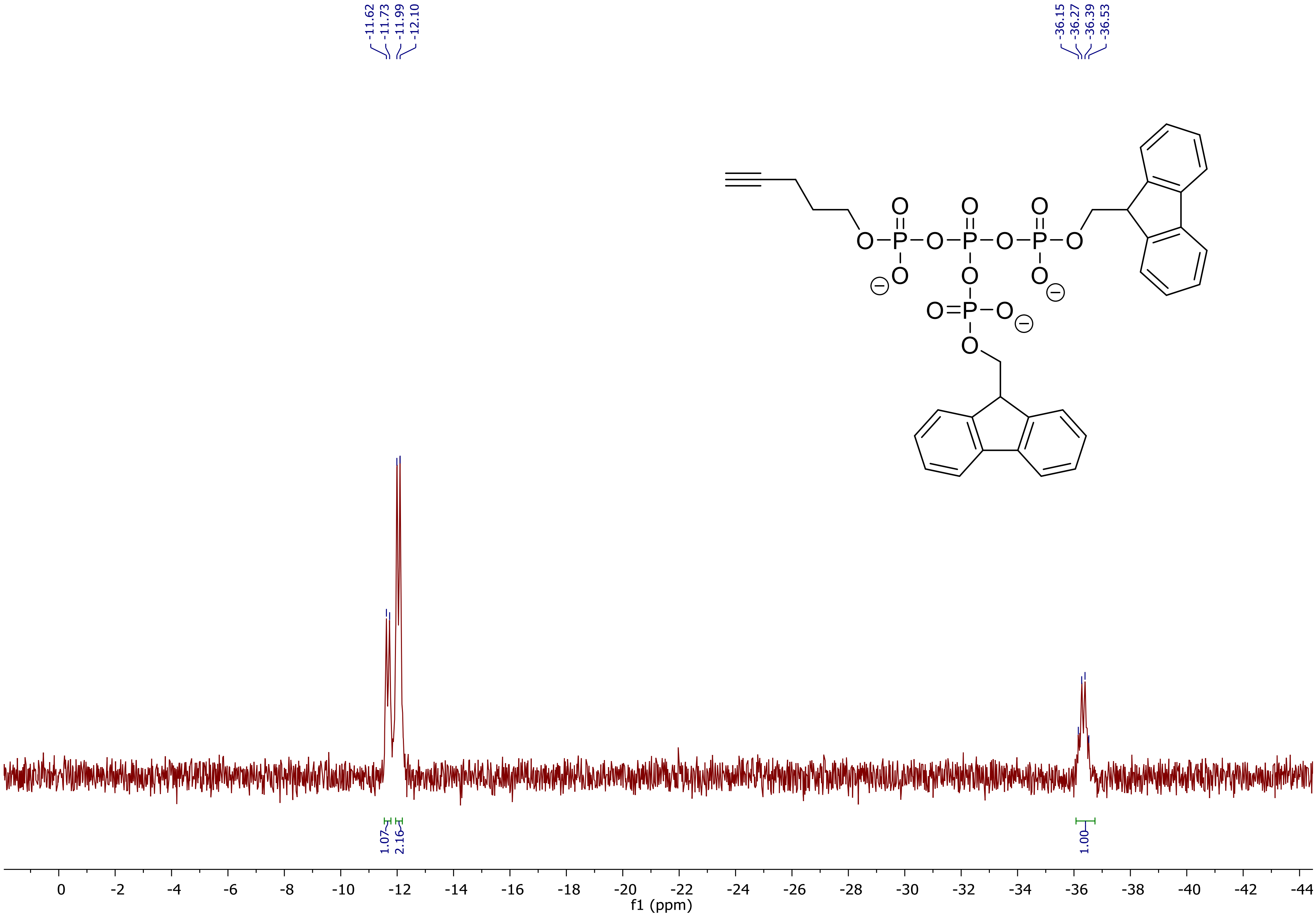

Supplementary Fig. 118 | **^{31}P-NMR** (162 MHz, D_2O), compound **49**:

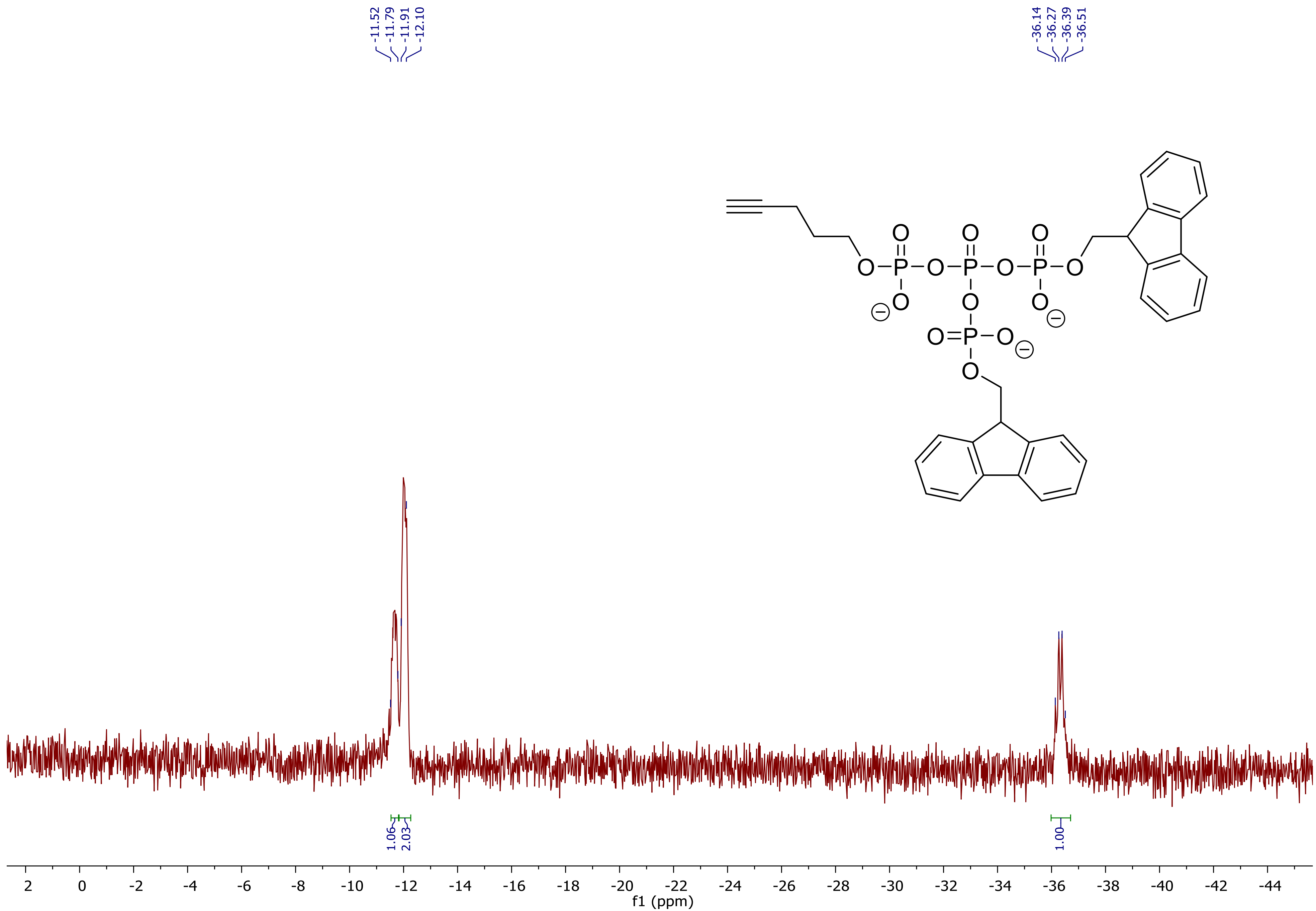

Supplementary Fig. 119│ **^{1}H-^{31}P-HMBC** (D_2O), compound **49**:

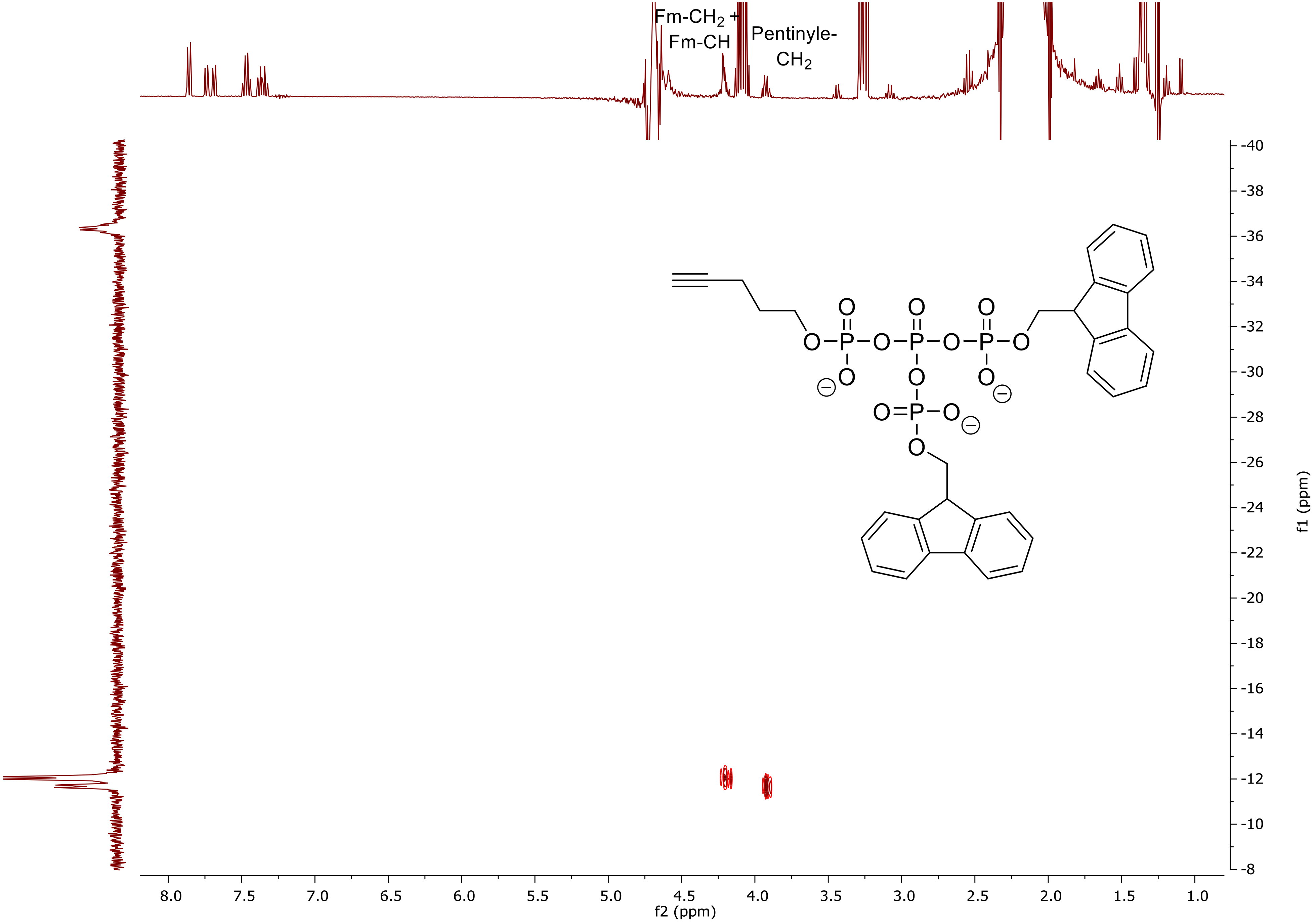

Mass spectrometry data

Supplementary Fig. 120│ HRMS (ESI), compound **20**:

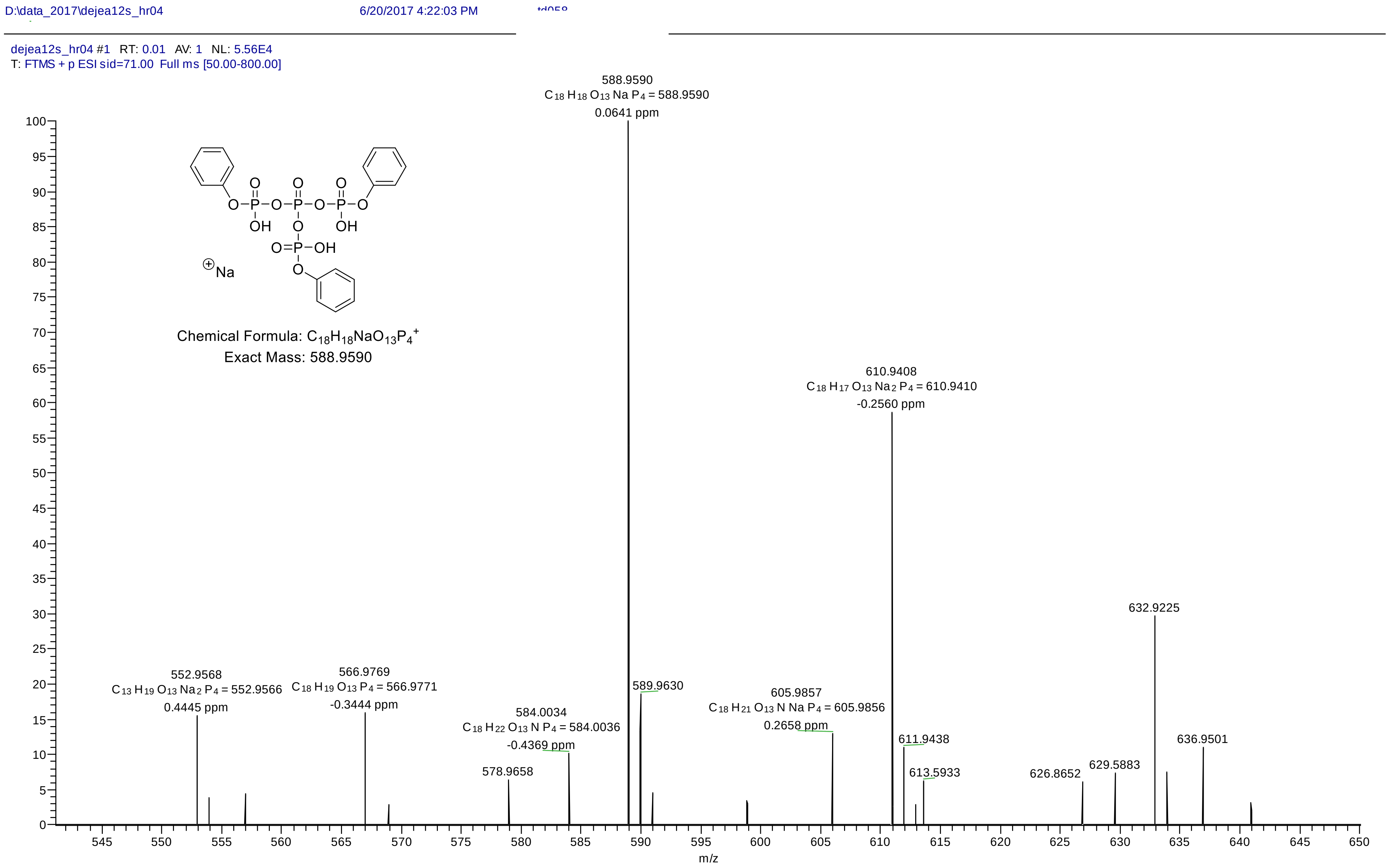

Supplementary Fig. 121 | HRMS (ESI), compound **21**:

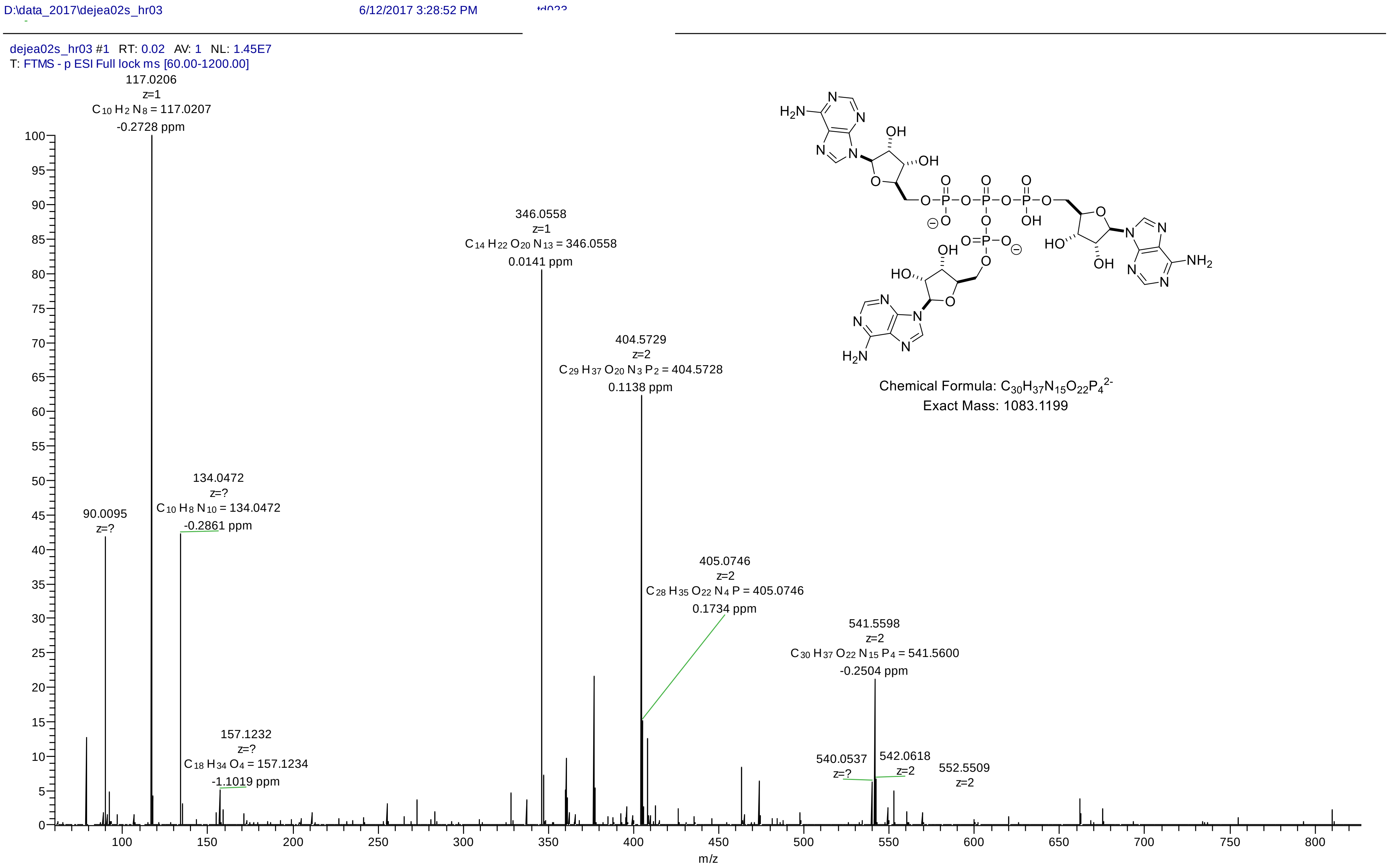

Supplementary Fig. 122 | HRMS (ESI), compound **22**:

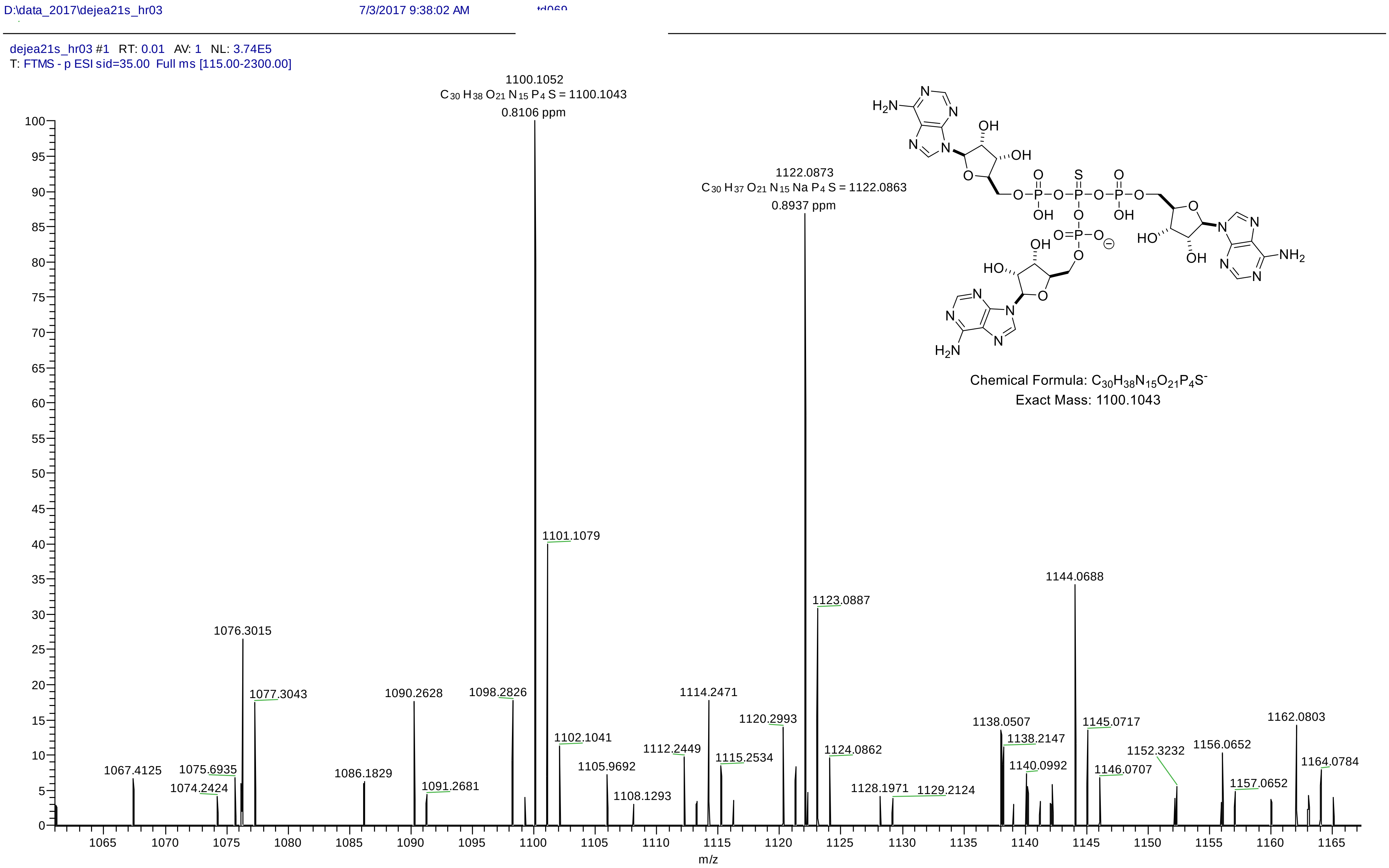

Supplementary Fig. 123│ HRMS (ESI), compound **23**:

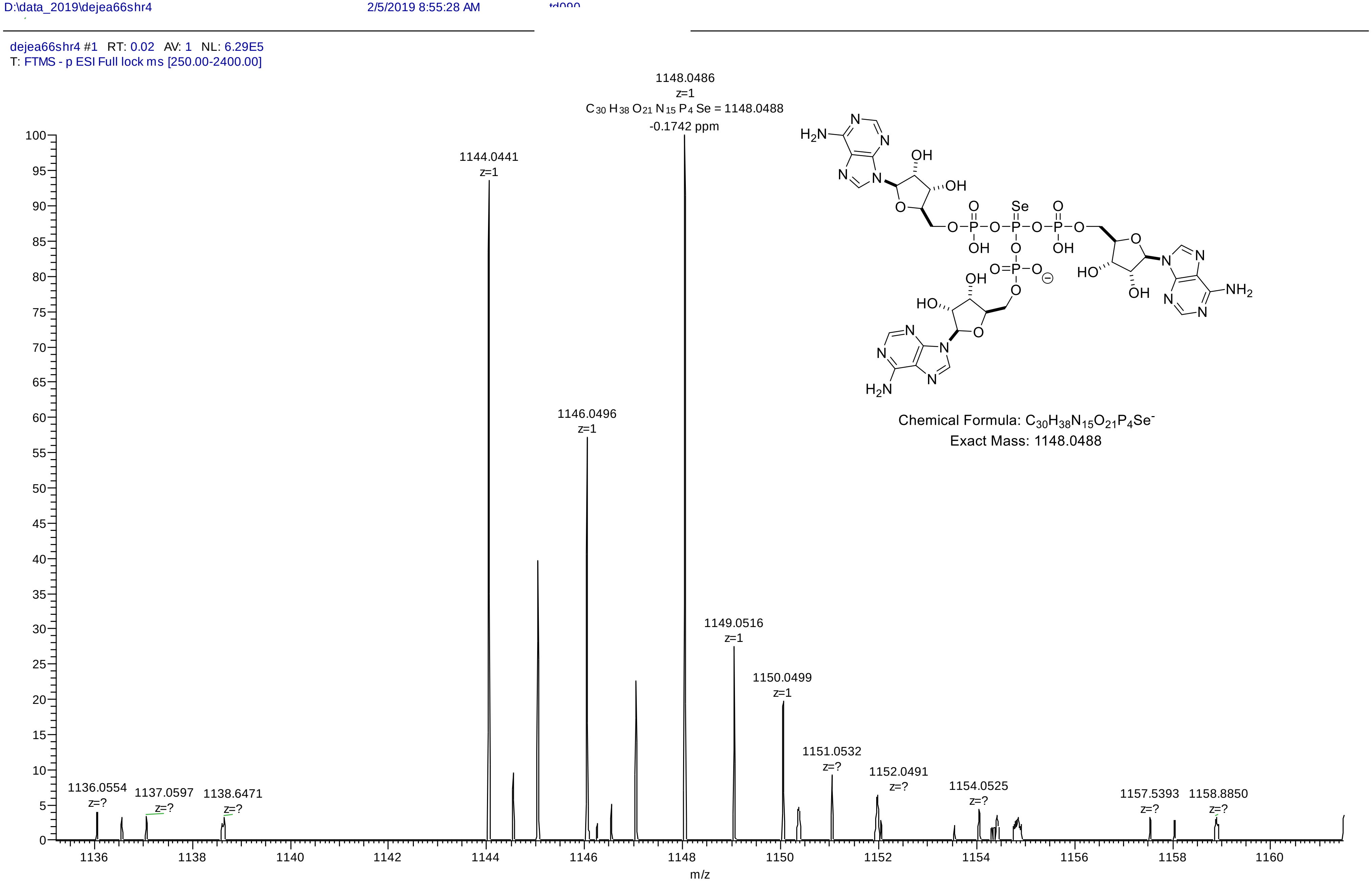

Supplementary Fig. 124 | **HRMS (ESI)**, compound **24**:

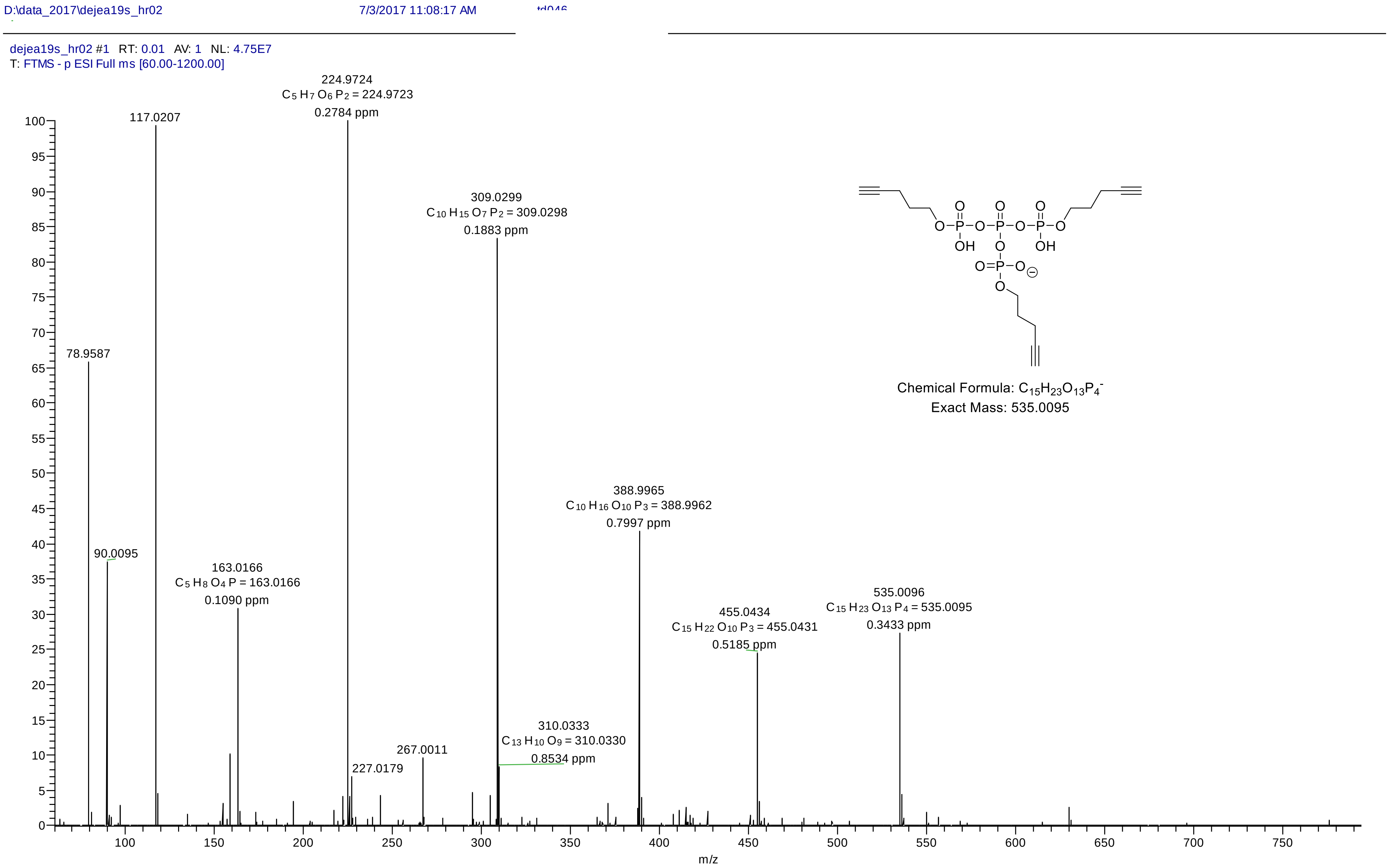

Supplementary Fig. 125│ HRMS (ESI), compound **25**:

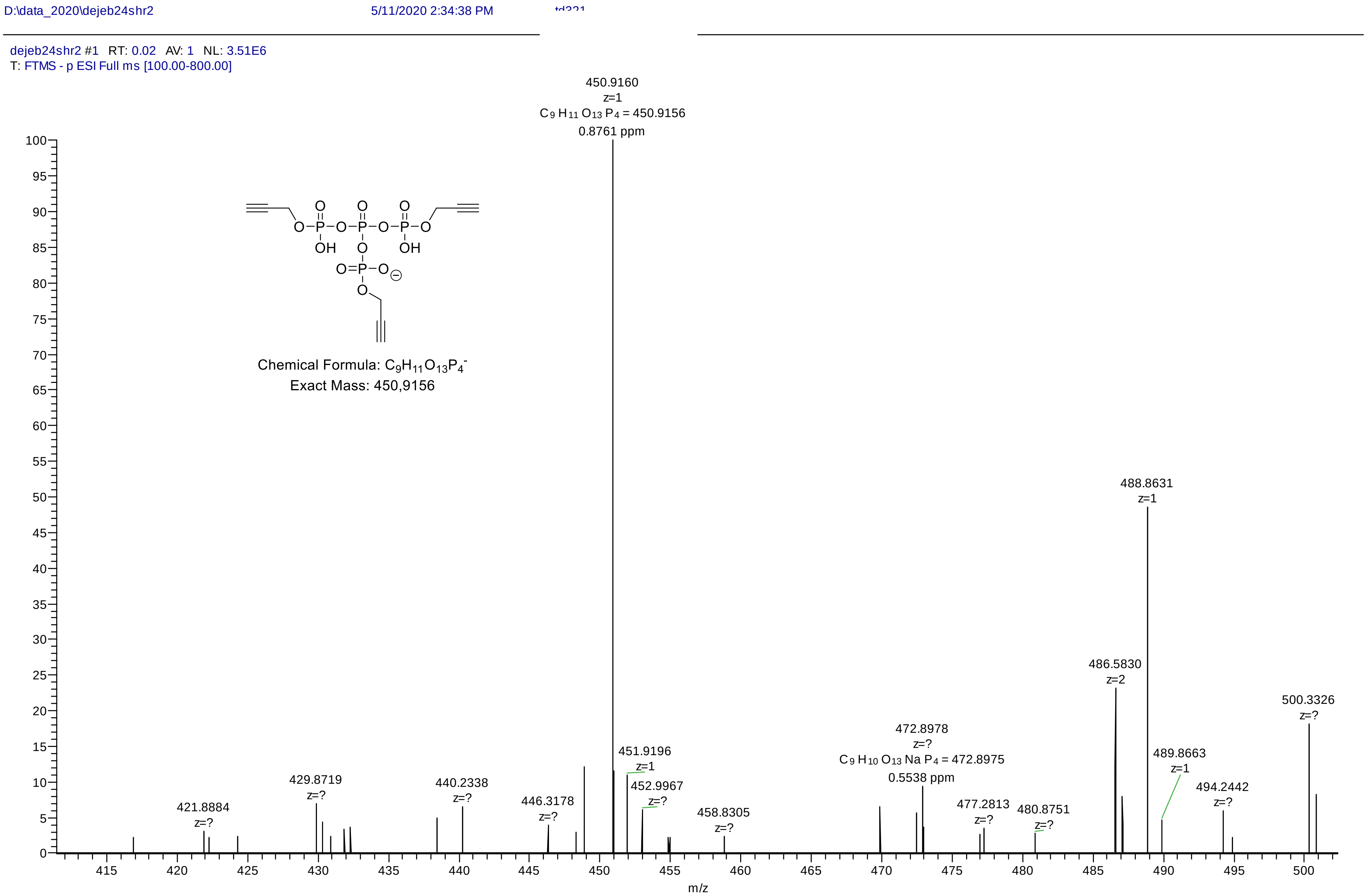

Supplementary Fig. 126 | HRMS (ESI), compound **26**:

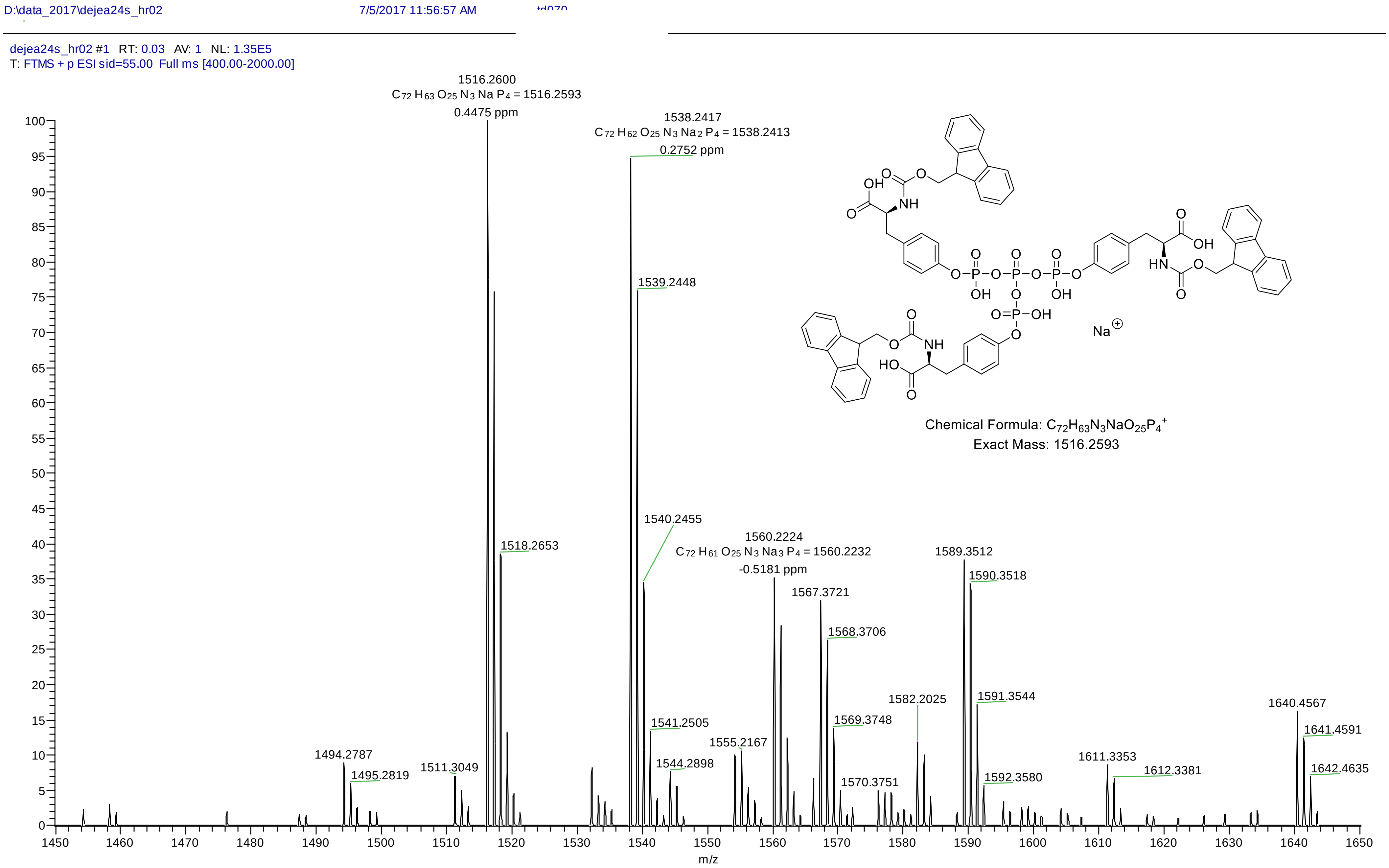

Supplementary Fig. 127 | HRMS (ESI), compound **27**:

D:\data_2017\dejea26s_hr07 7/12/2017 10:15:50 AM td077

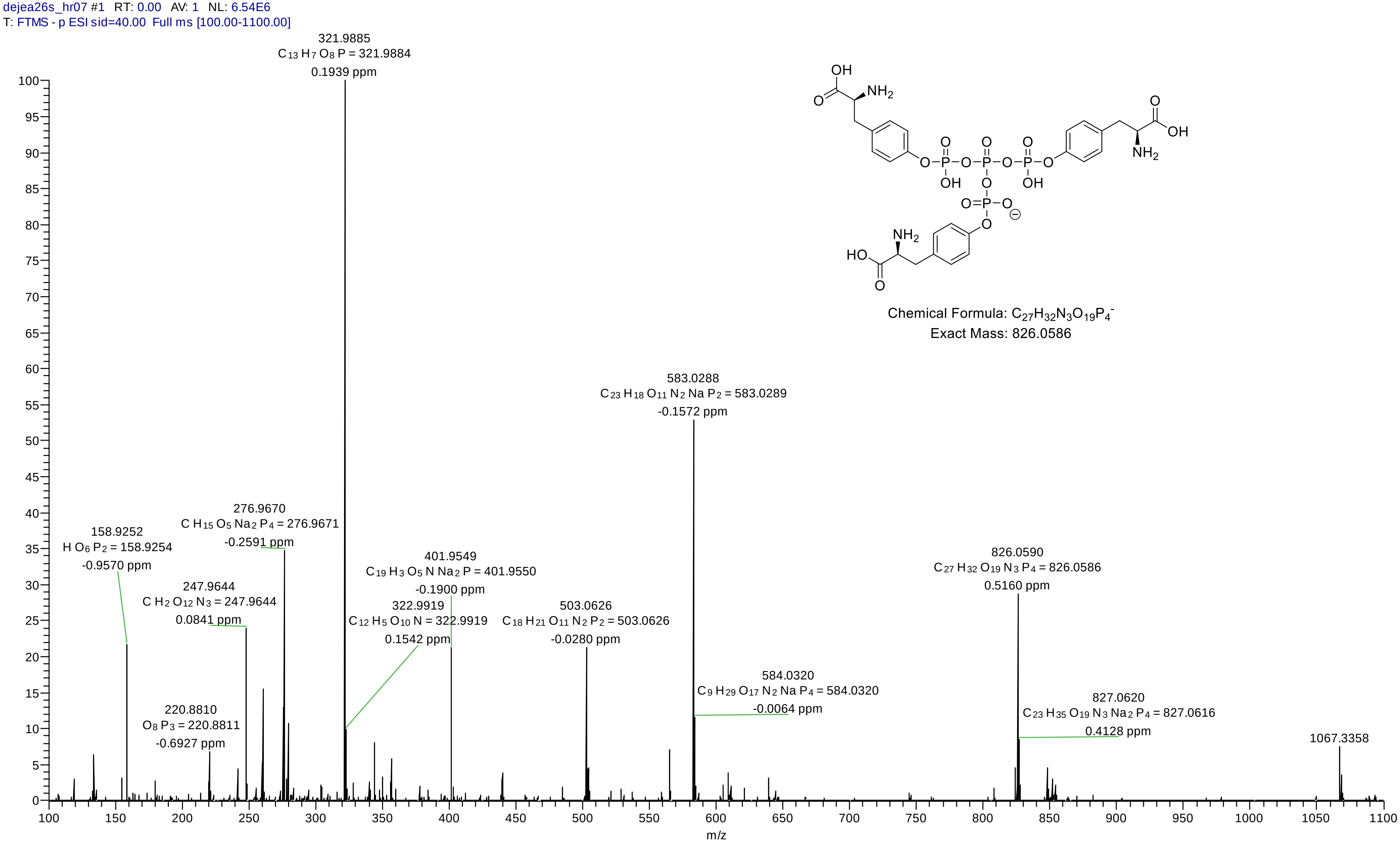

Chemical Formula: $C_{27}H_{32}N_3O_{19}P_4^-$
Exact Mass: 826.0586

Supplementary Fig. 128│ HRMS (ESI), compound **28**:

D:\data_2017\dejea22s_hr04 7/5/2017 11:34:55 AM td027

dejea22s_hr04 #1 RT: 0.02 AV: 1 NL: 5.83E5
T: FTMS - p ESI sid=60.00 Full ms [100.00-1200.00]

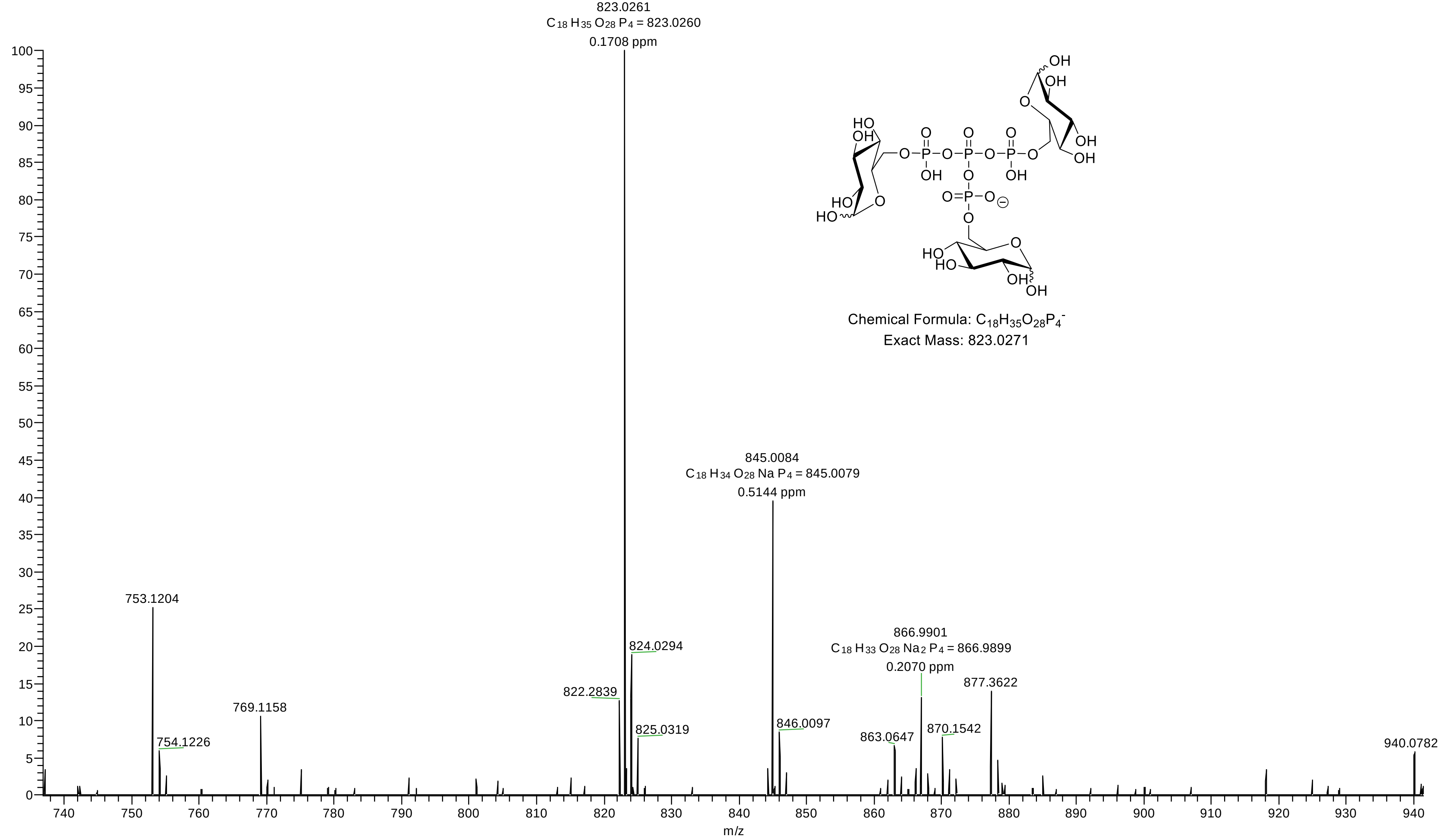

Supplementary Fig. 129 | HRMS (ESI), compound **29**:

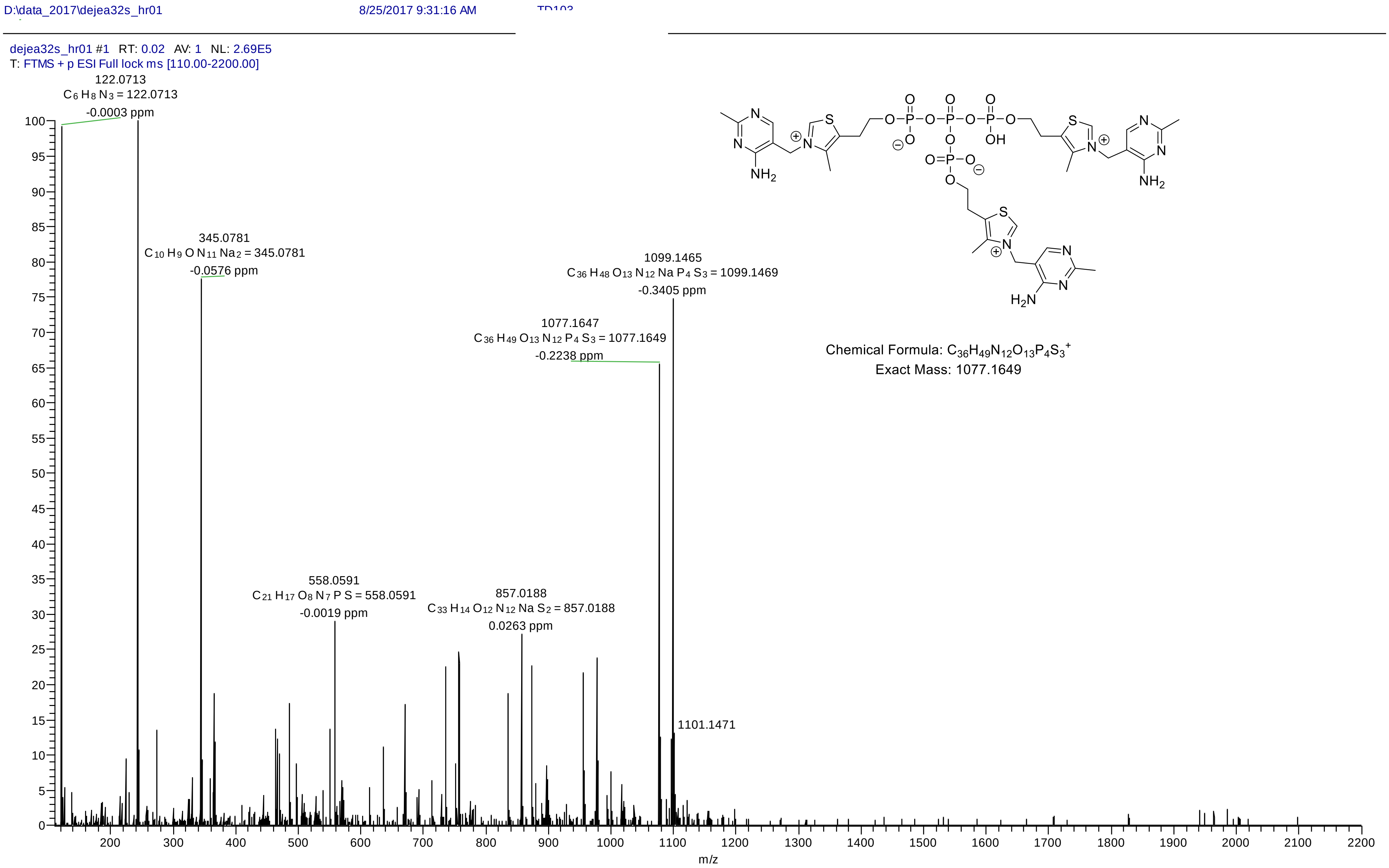

Chemical Formula: $C_{36}H_{49}N_{12}O_{13}P_4S_3^+$

Exact Mass: 1077.1649

Supplementary Fig. 130│ HRMS (ESI), compound **30**:

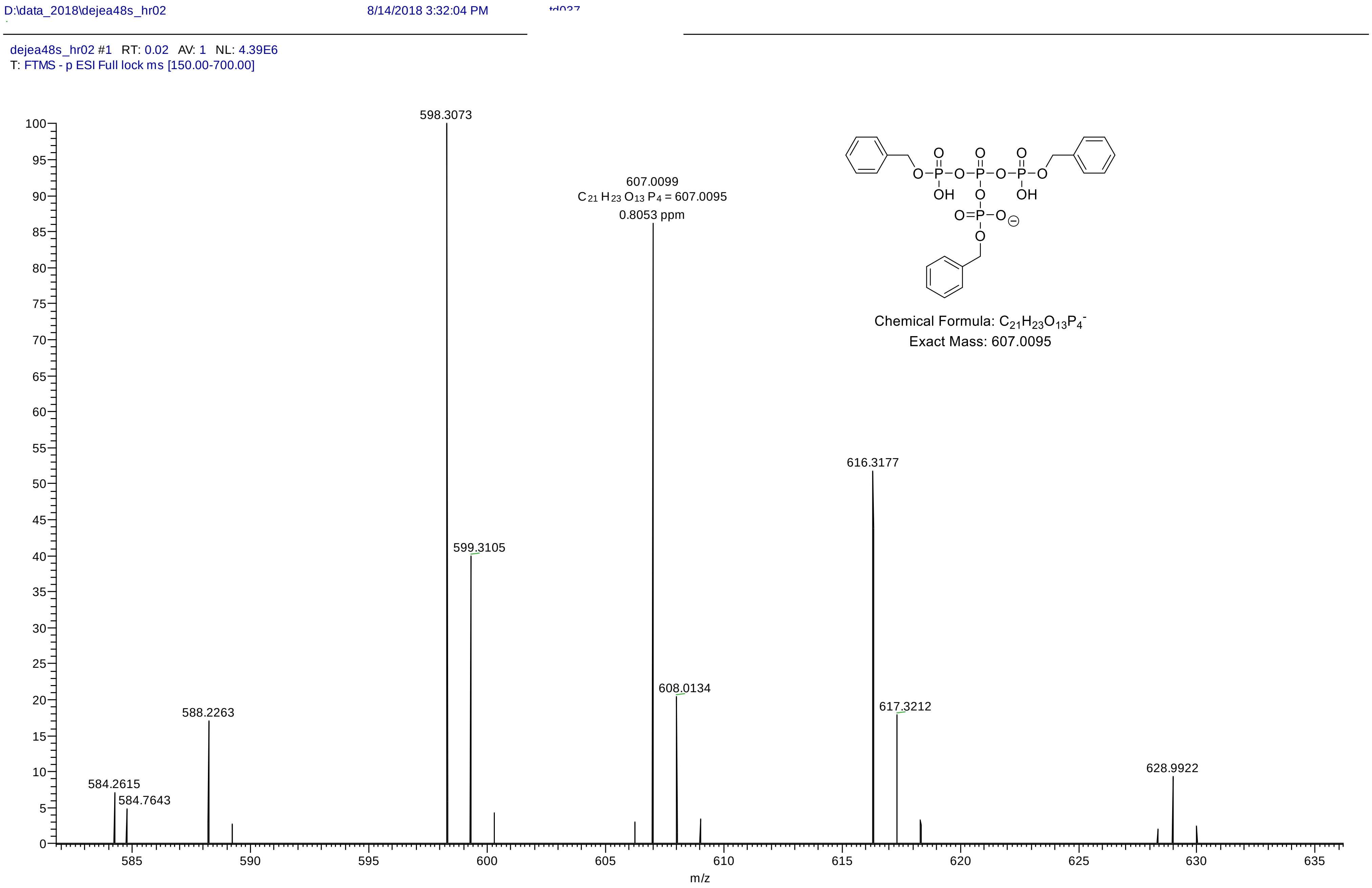

Supplementary Fig. 131 | HRMS (ESI), compound **31**:

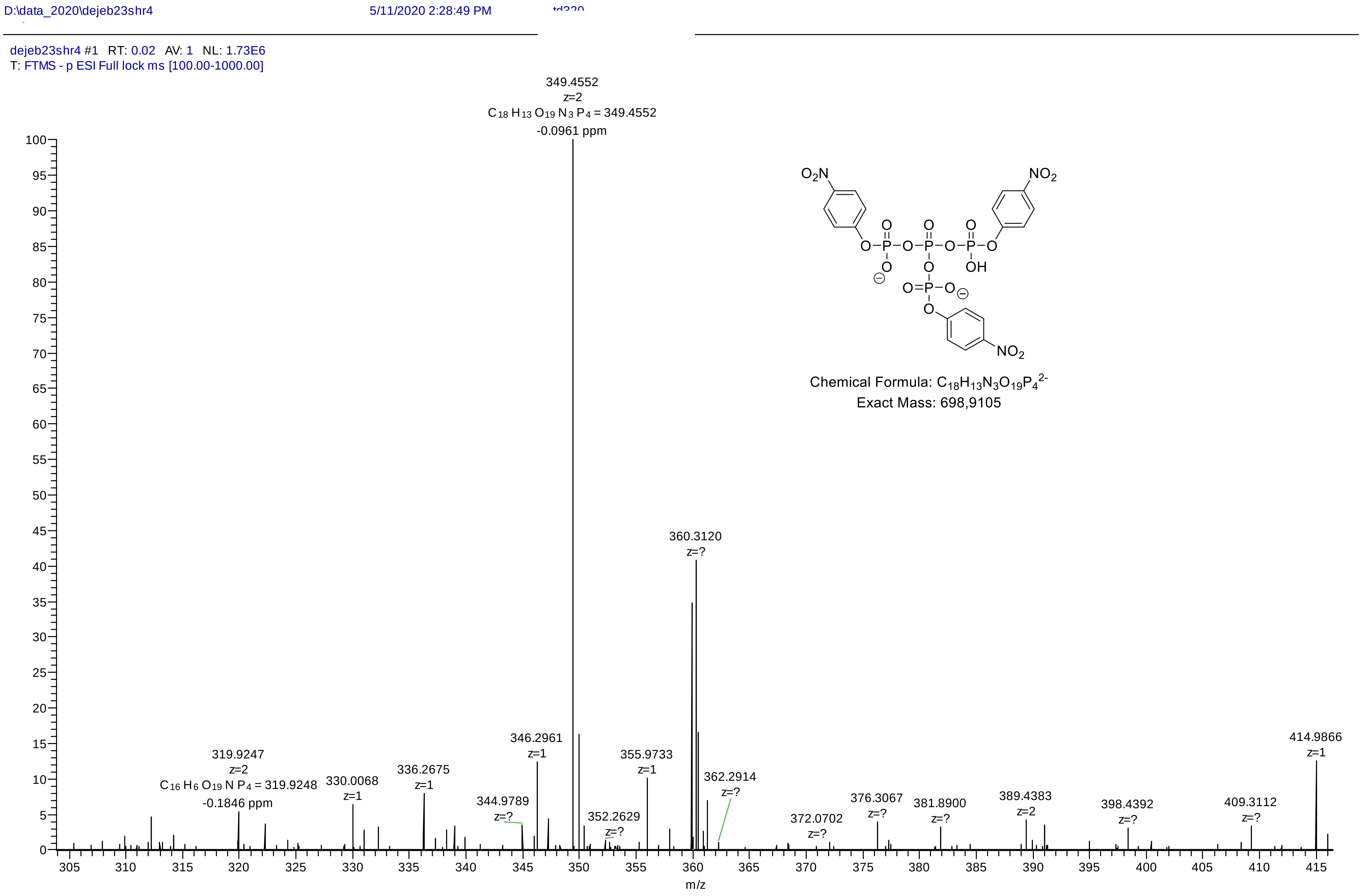

Chemical Formula: $C_{18}H_{13}N_3O_{19}P_4^{2-}$
Exact Mass: 698,9105

Supplementary Fig. 132│ HRMS (ESI), compound **64**:

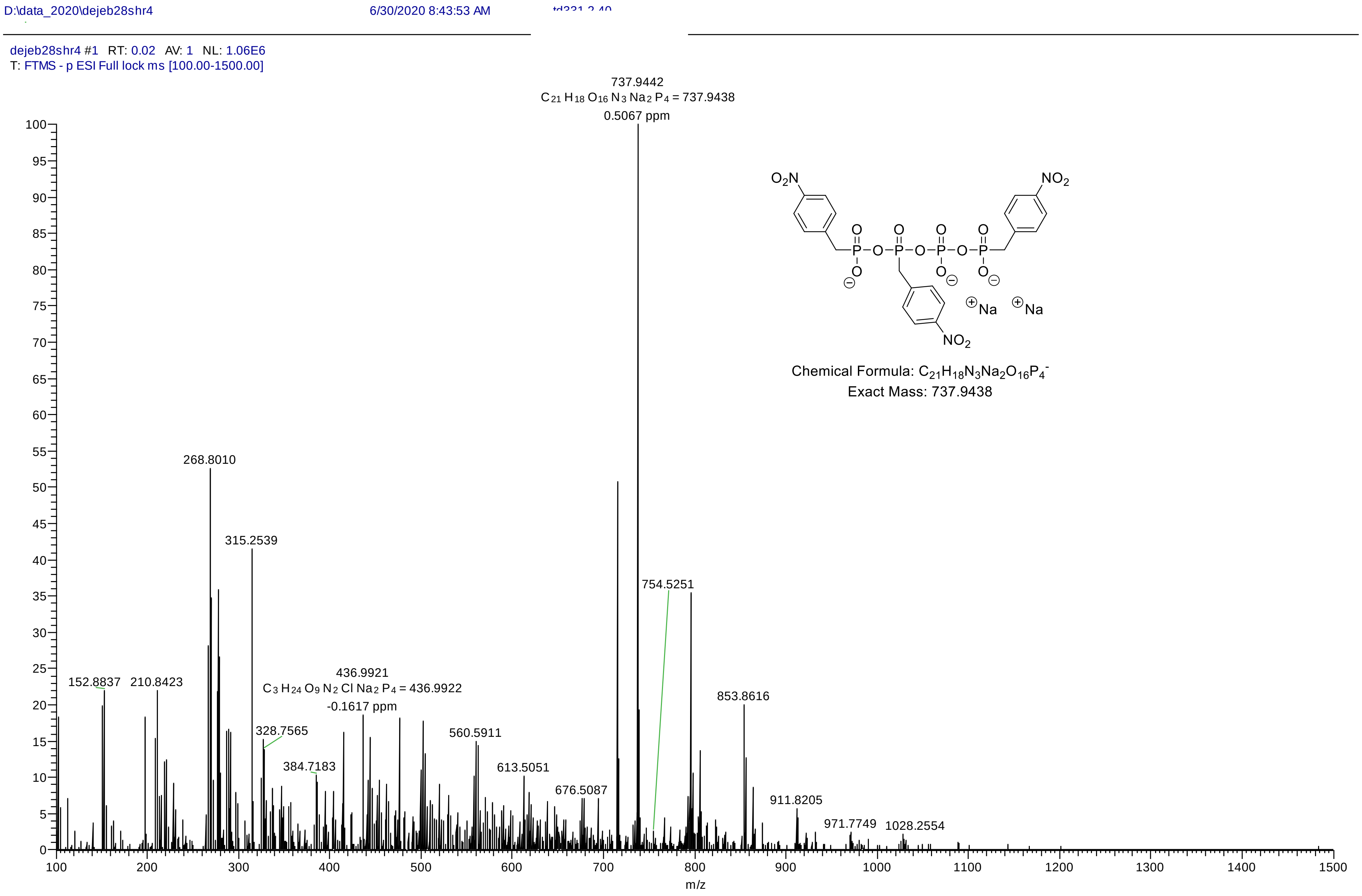

Supplementary Fig. 133 | HRMS (ESI), compound **32**:

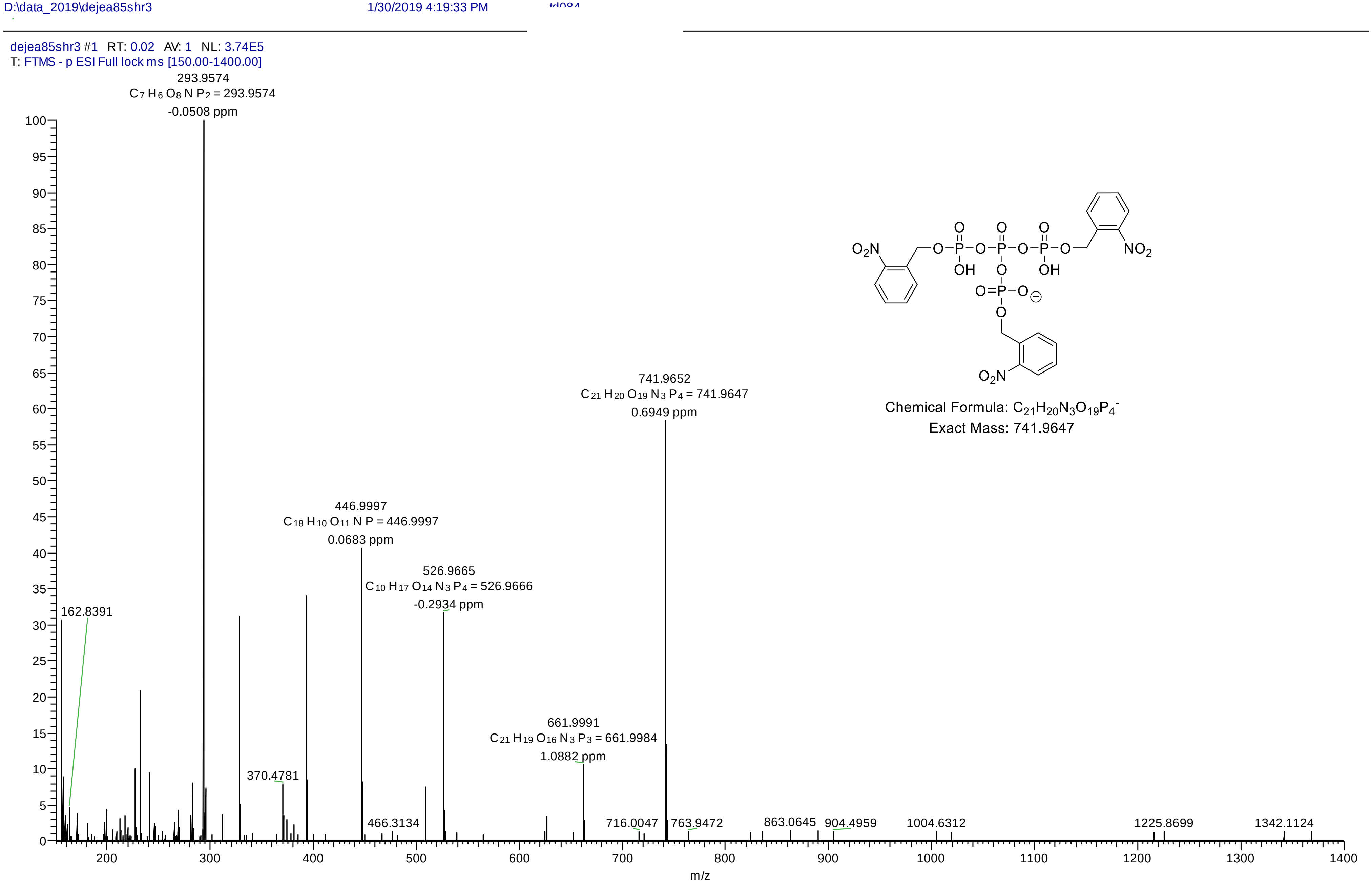

Chemical Formula: $C_{21}H_{20}N_3O_{19}P_4^-$

Exact Mass: 741.9647

Supplementary Fig. 134 | **HRMS (ESI)**, compound **33**:

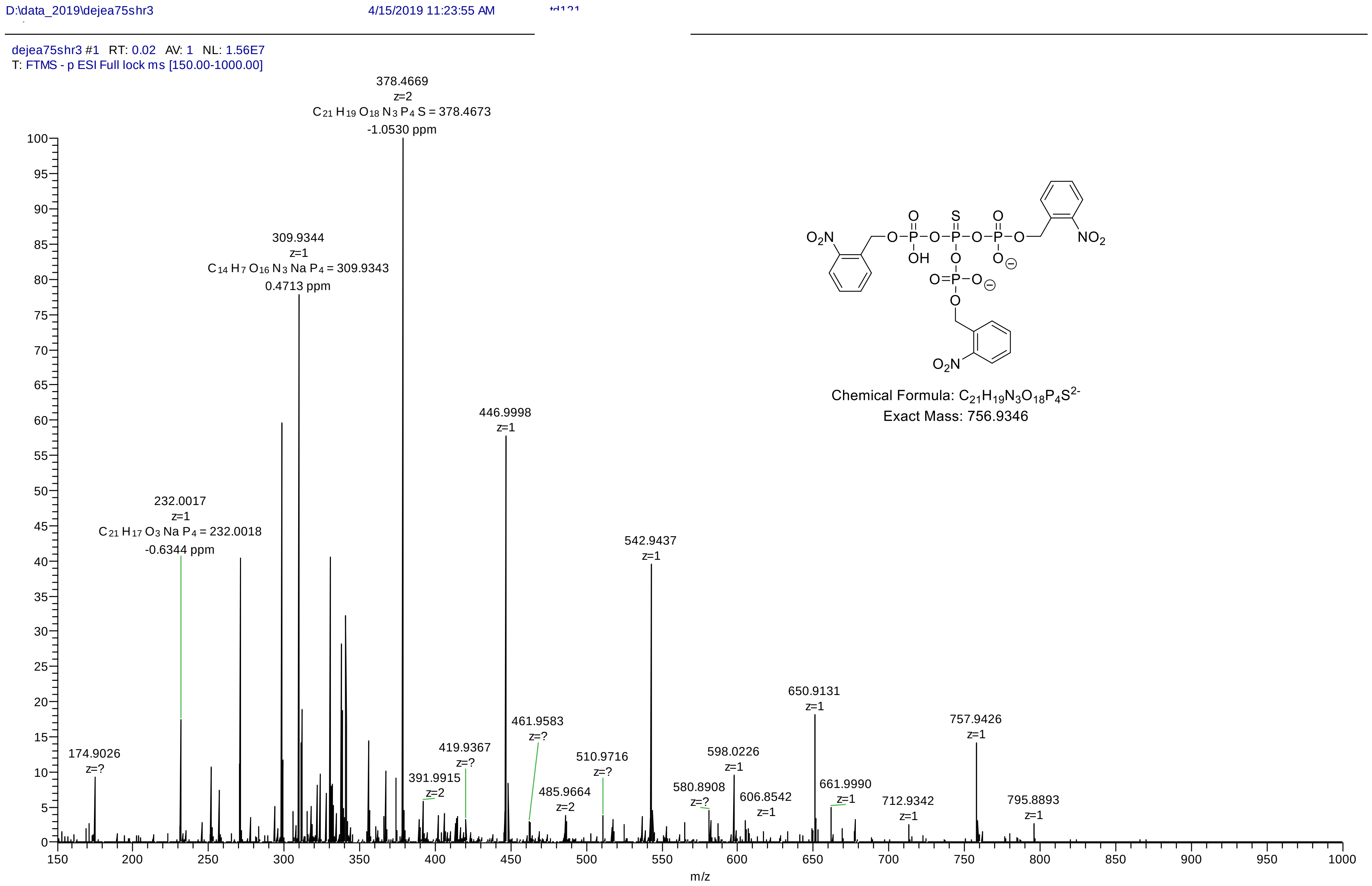

Supplementary Fig. 135 | HRMS (ESI), compound **34**:

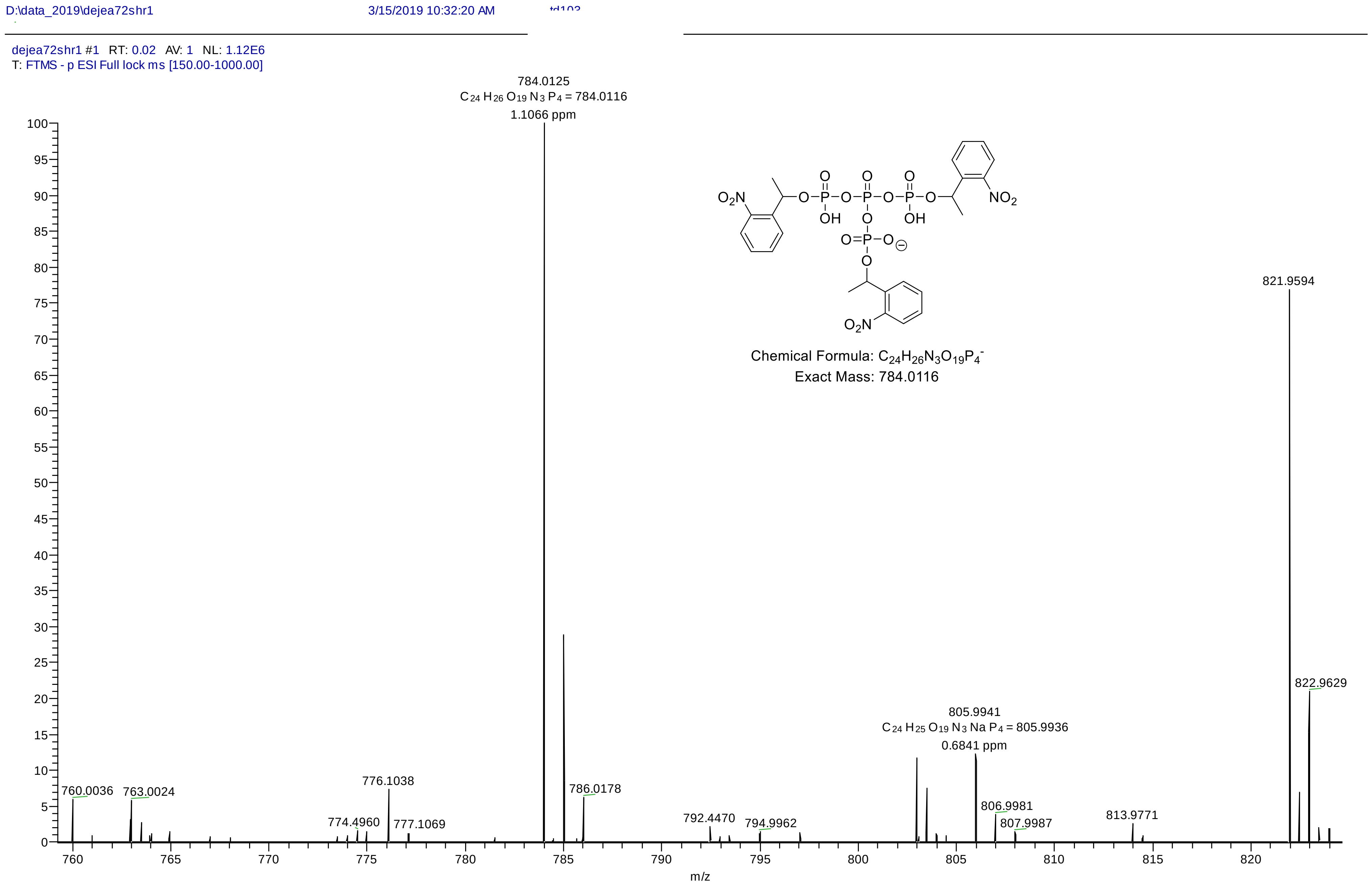

Supplementary Fig. 136 | HRMS (ESI), compound **35**:

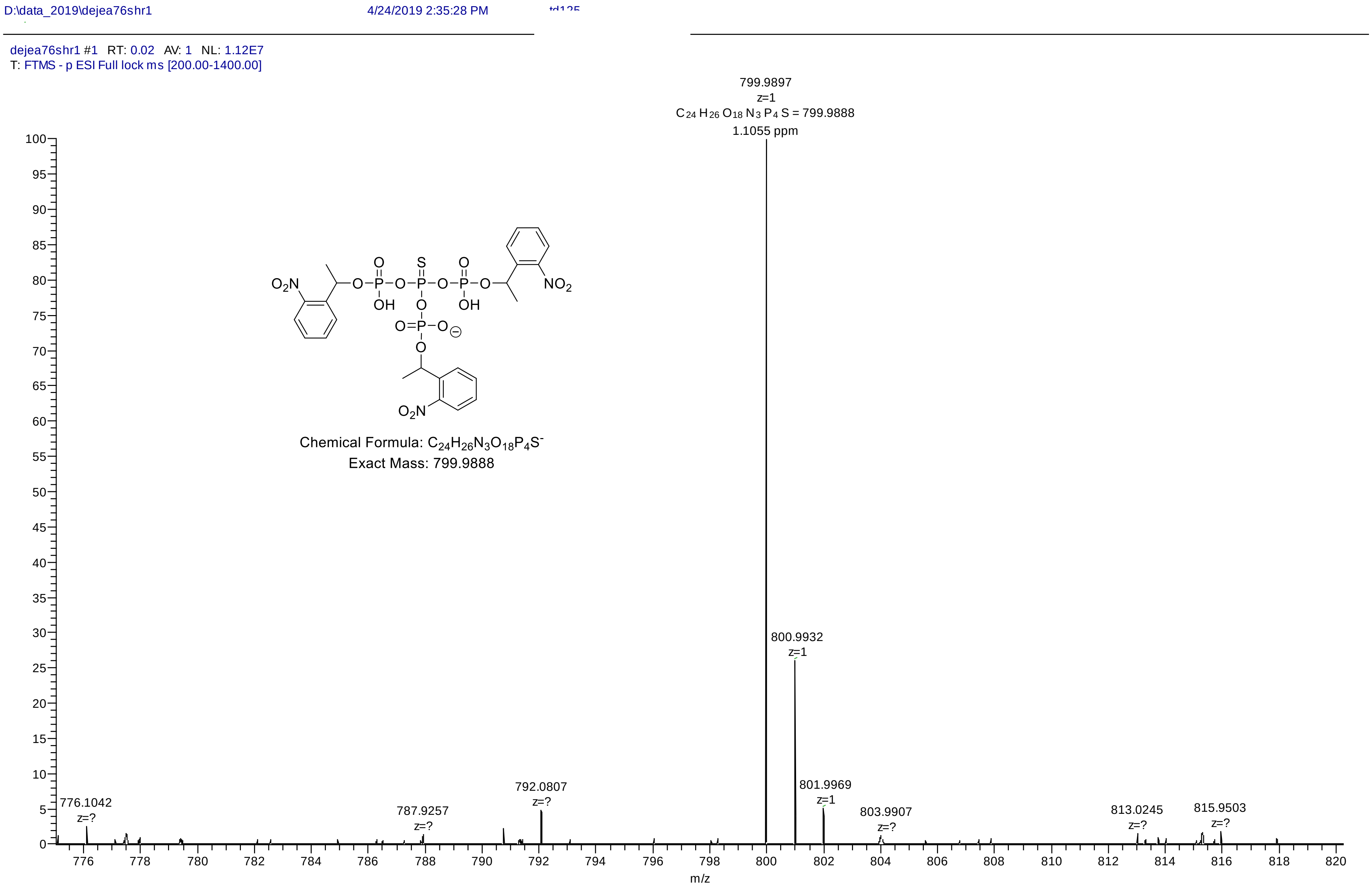

Supplementary Fig. 137 | HRMS (ESI), compound **36**:

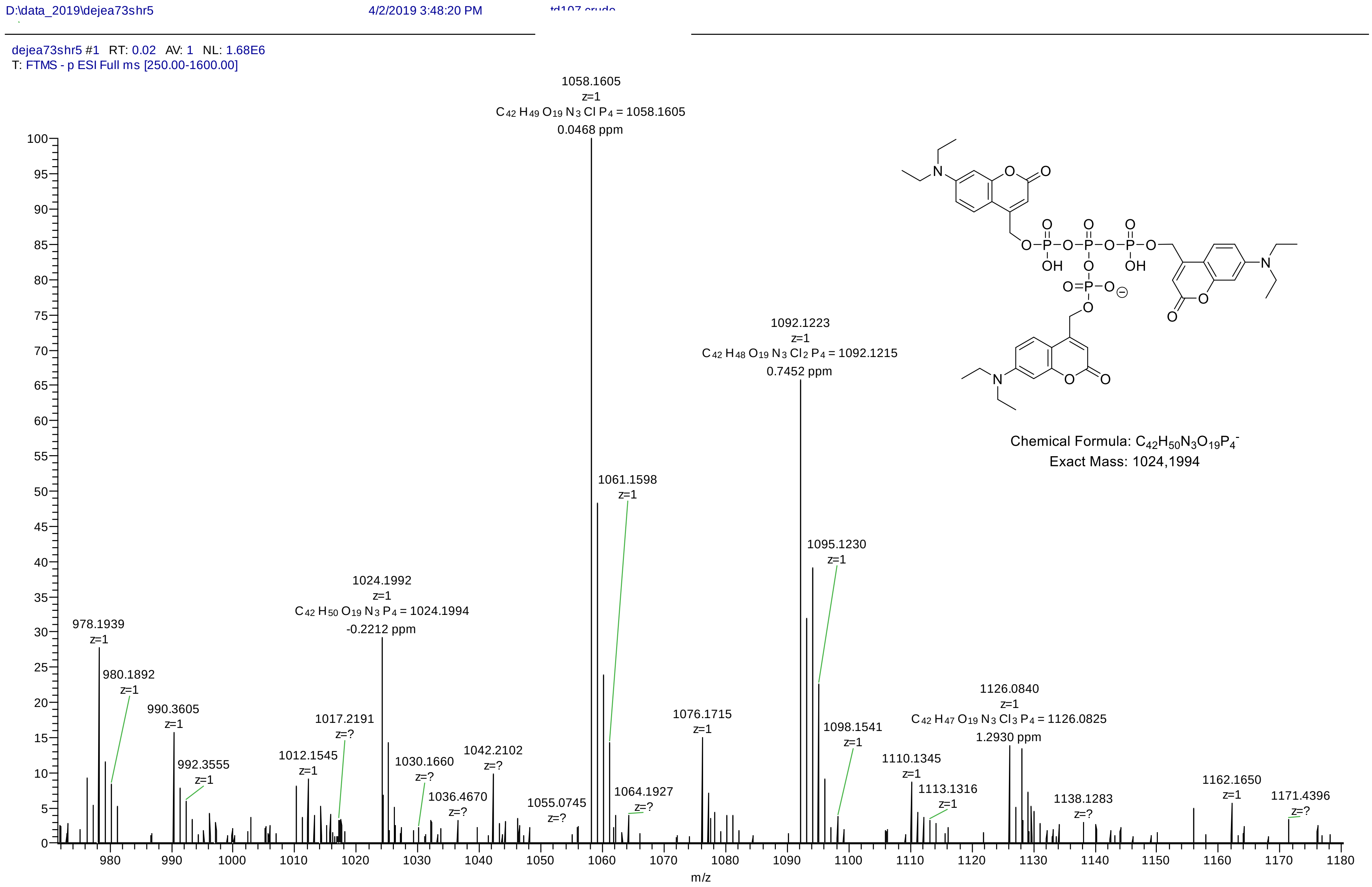

Chemical Formula: $C_{42}H_{50}N_3O_{19}P_4^-$

Exact Mass: 1024,1994

Supplementary Fig. 138 | HRMS (ESI), compound **37**:

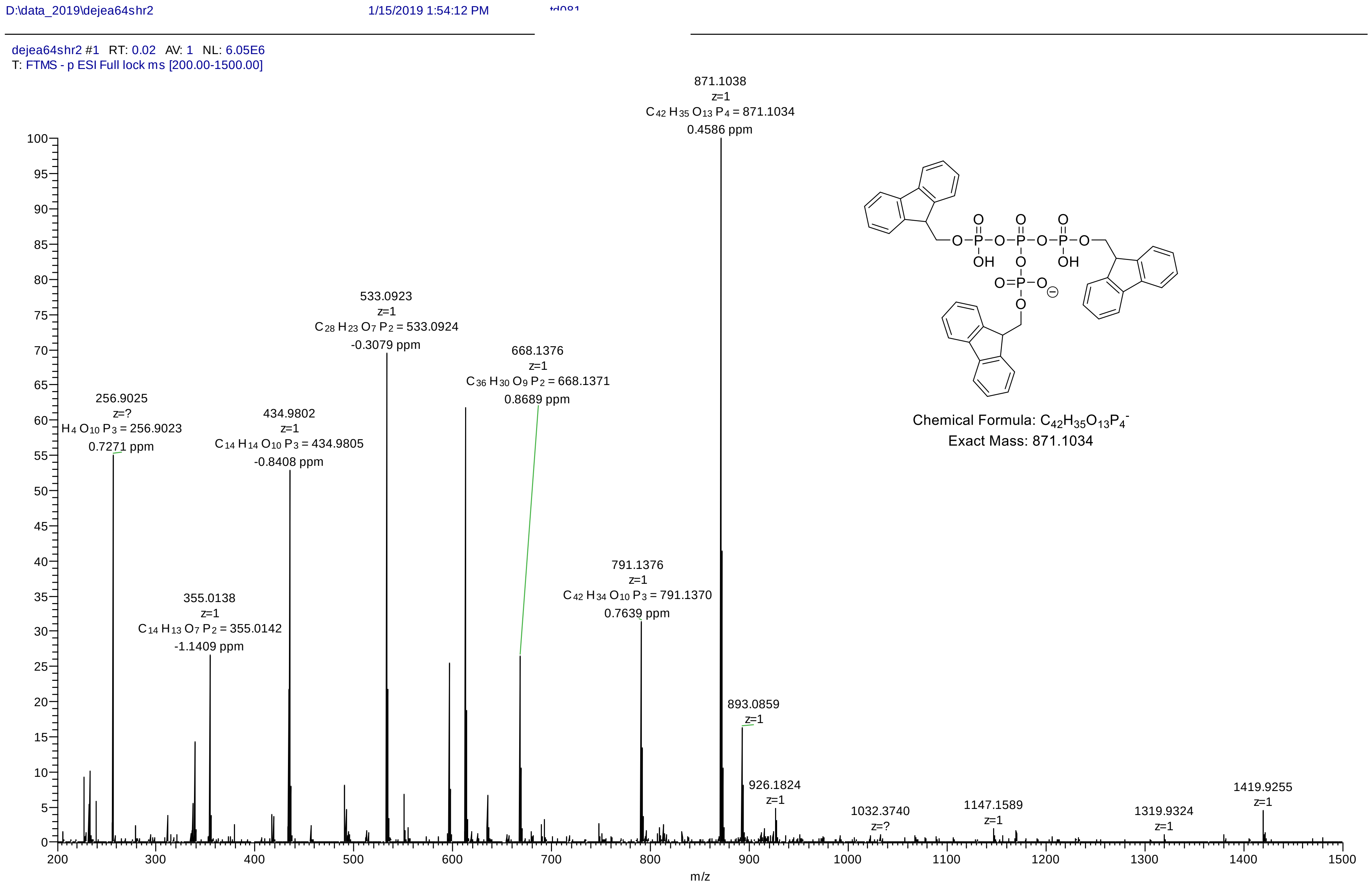

Supplementary Fig. 139│ HRMS (ESI), compound **2**:

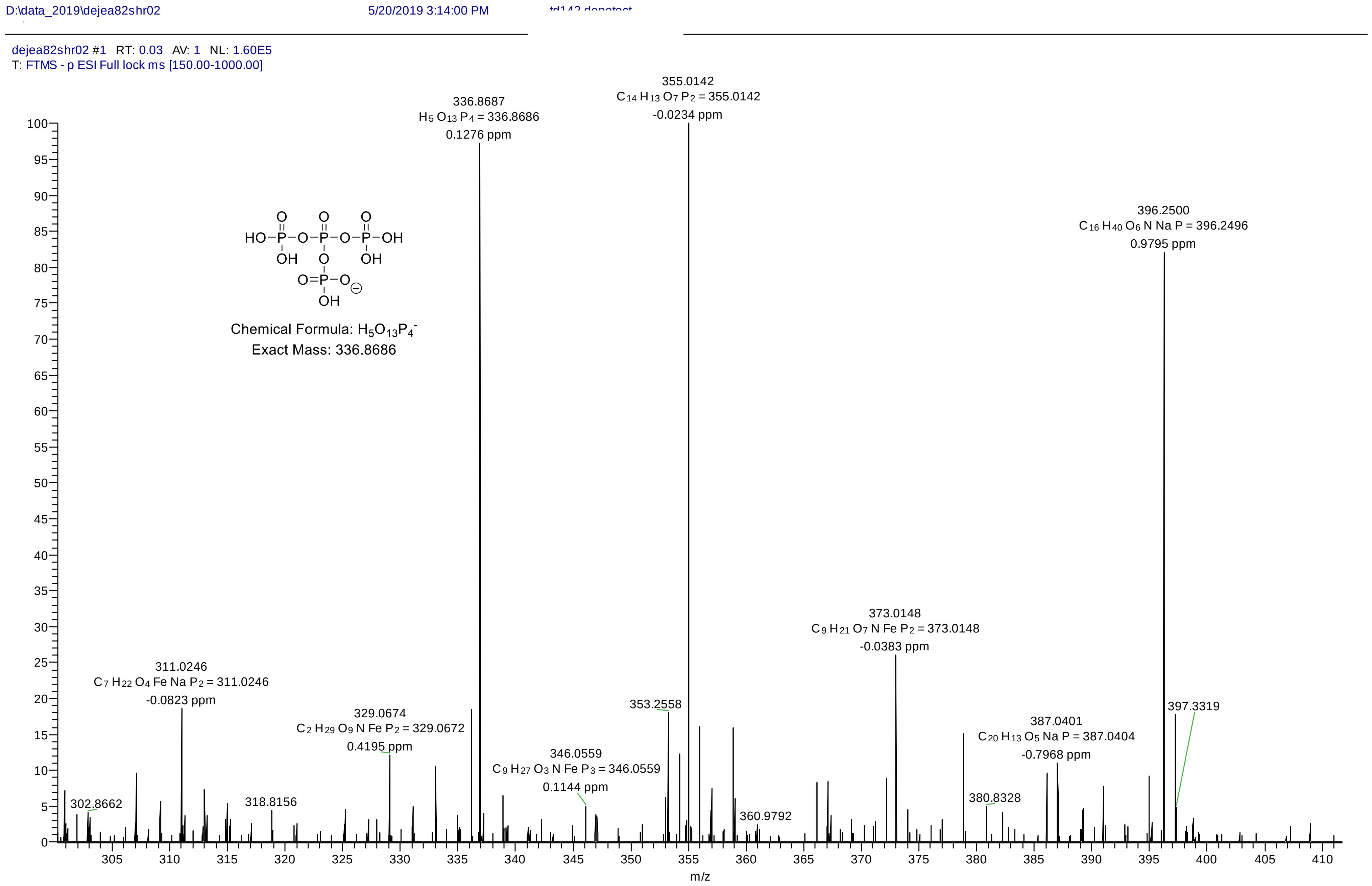

Supplementary Fig. 140│ HRMS (ESI), compound **38**:

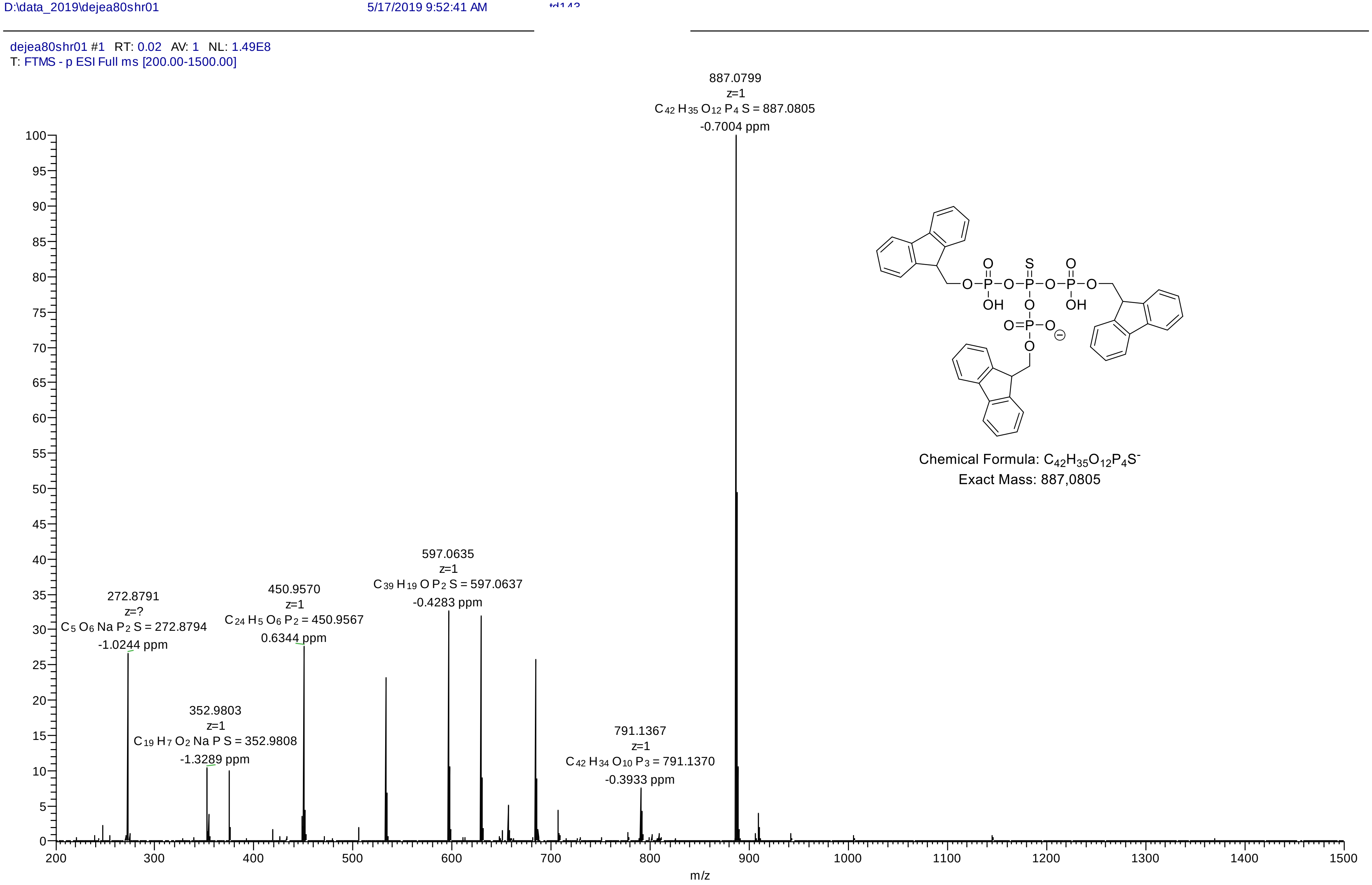

Chemical Formula: $C_{42}H_{35}O_{12}P_4S^-$
Exact Mass: 887,0805

Supplementary Fig. 141 | **HRMS (ESI)**, compound **15**:

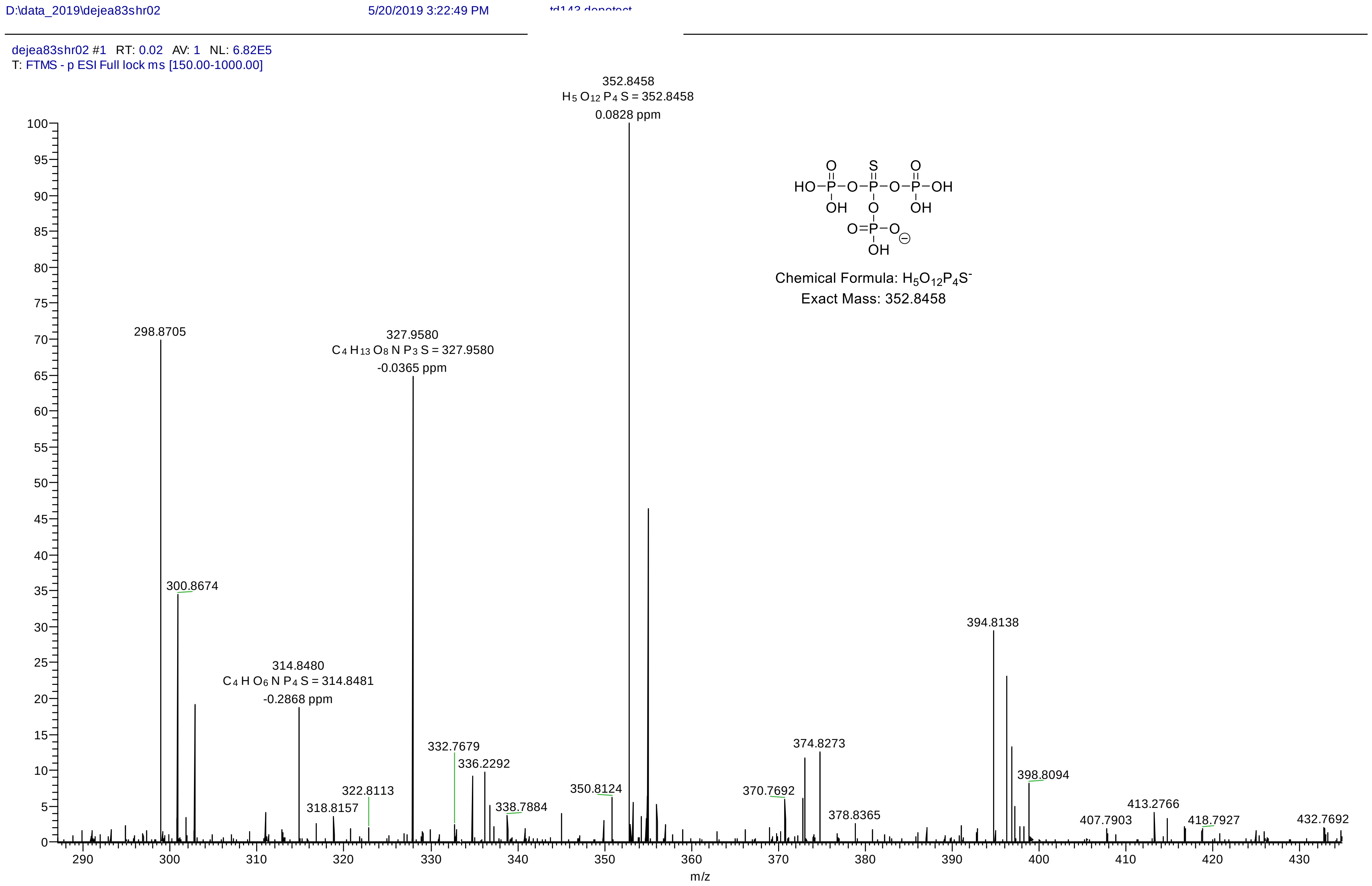

Supplementary Fig. 142 | HRMS (ESI), compound **43**:

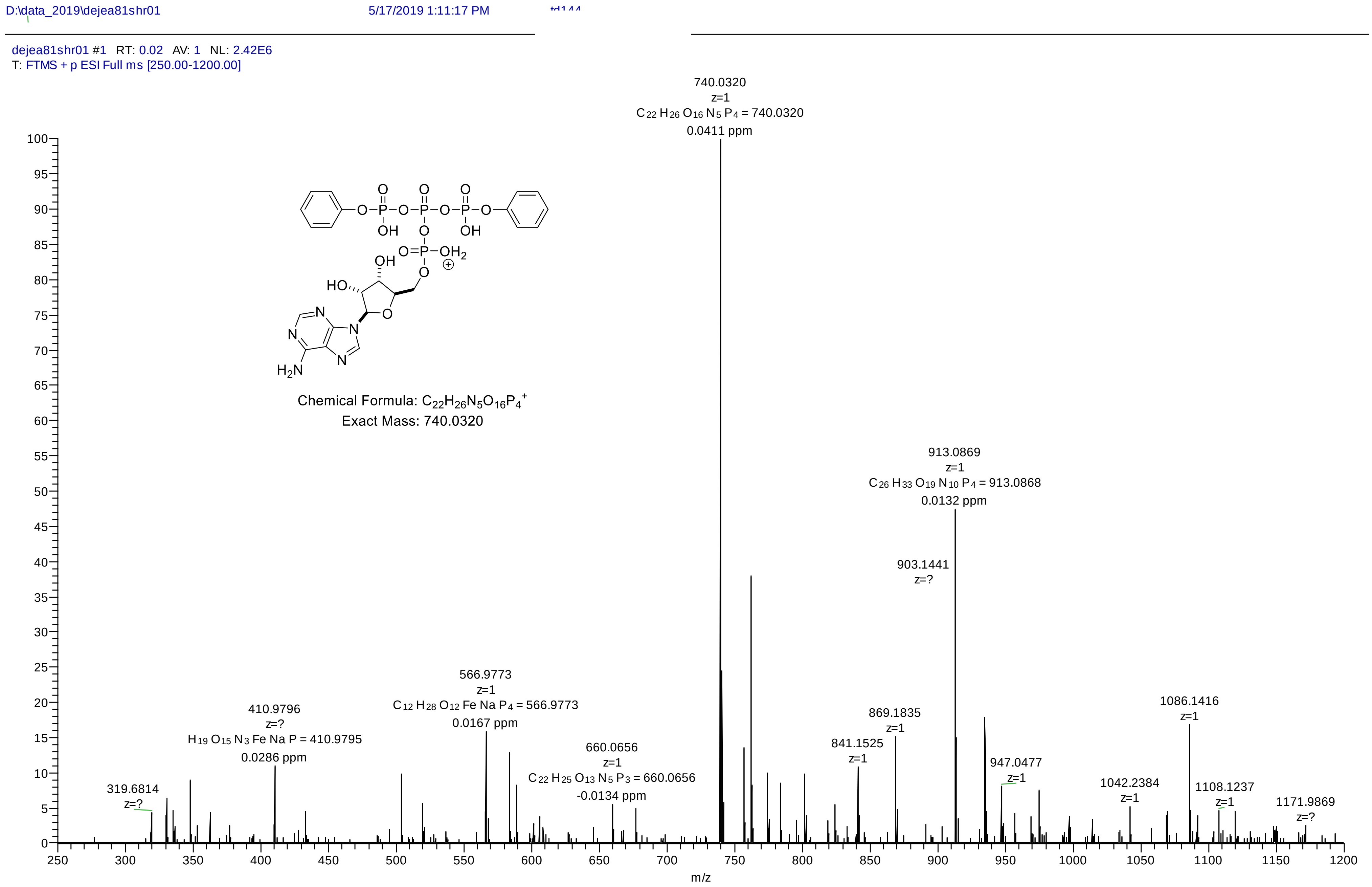

Supplementary Fig. 143 | HRMS (ESI), compound **44**:

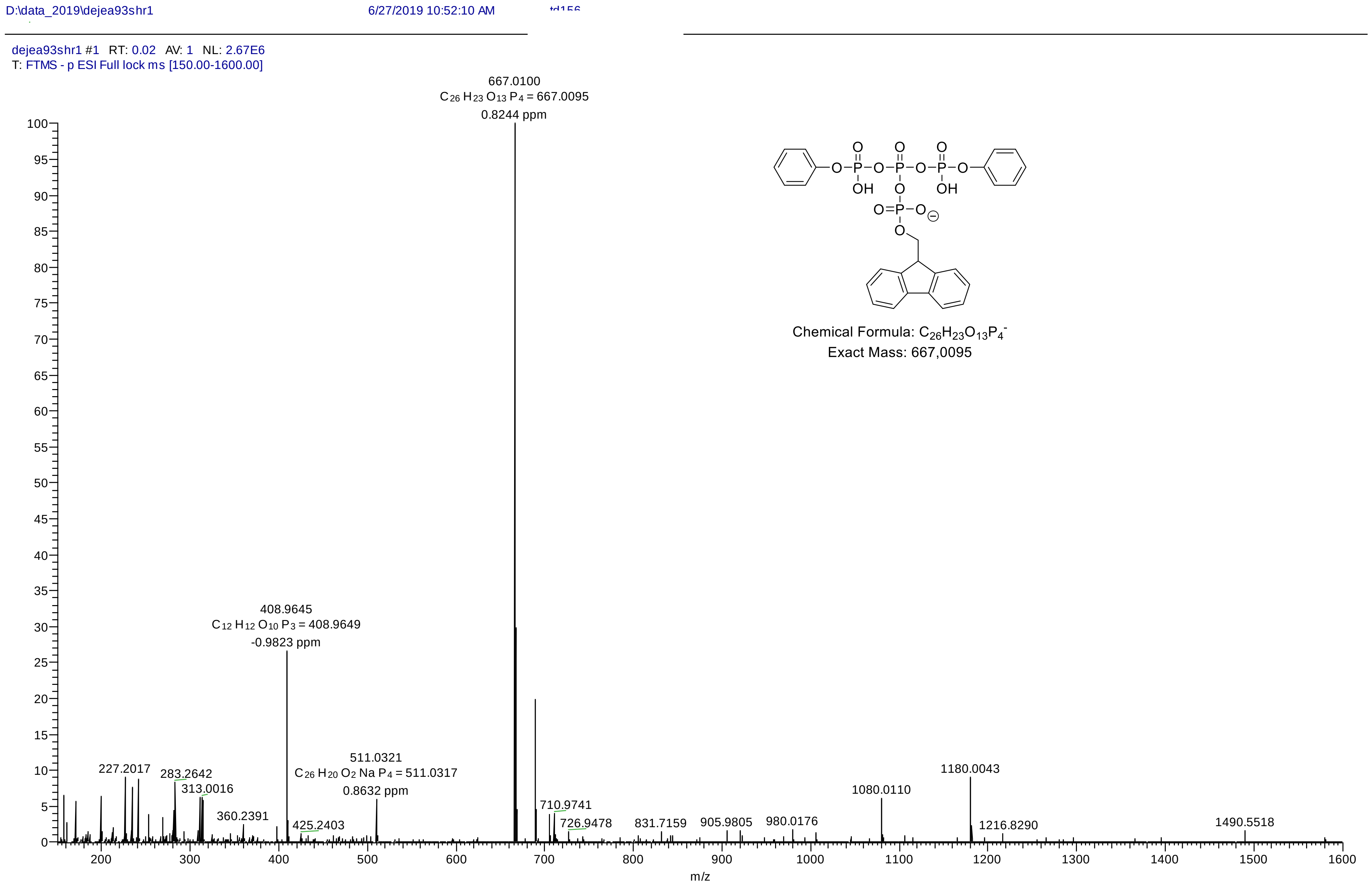

Supplementary Fig. 144│ HRMS (ESI), compound **45**:

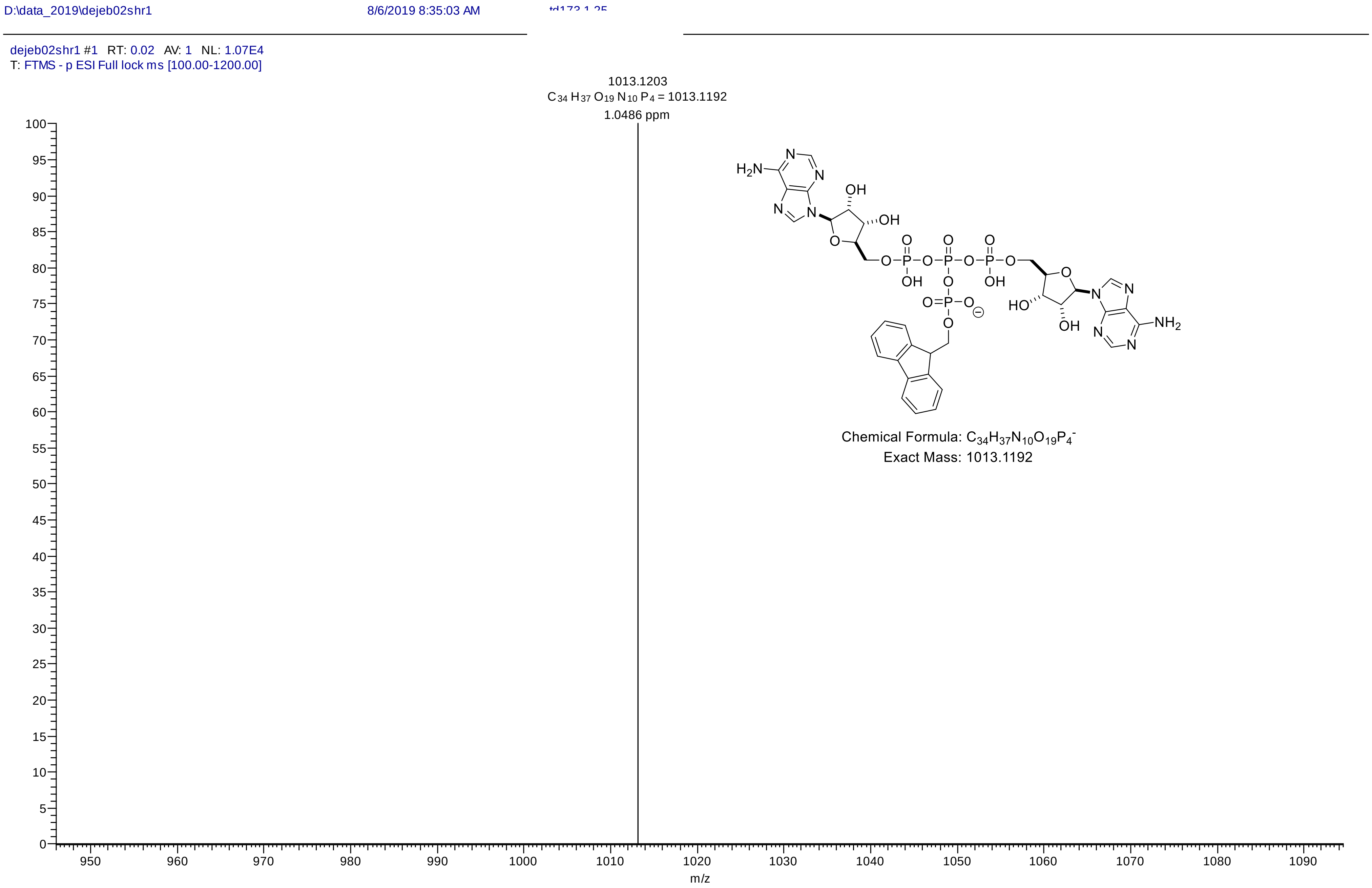

Supplementary Fig. 145 | HRMS (ESI), compound **54**:

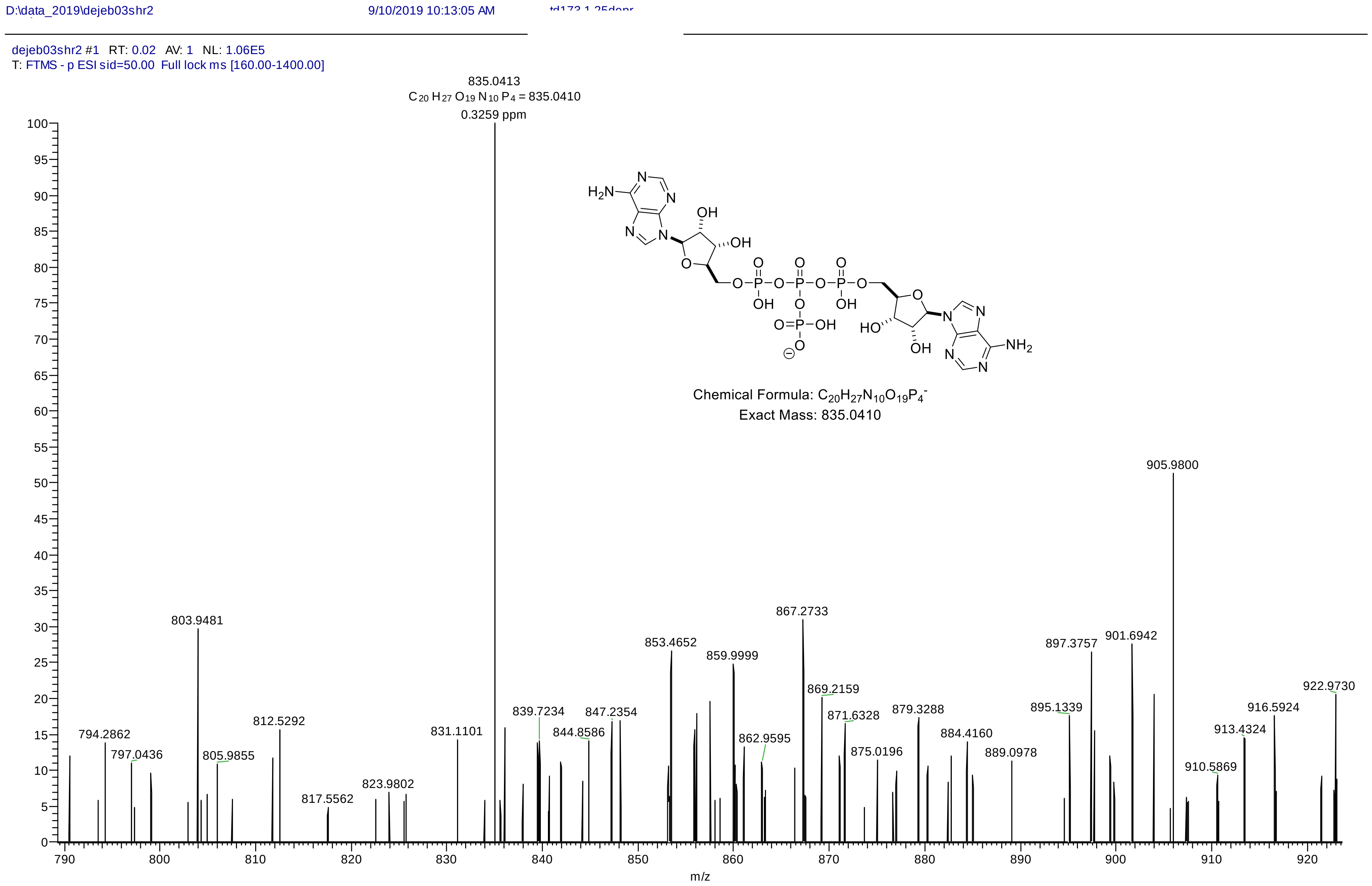

Supplementary Fig. 146│ HRMS (ESI), compound **46**:

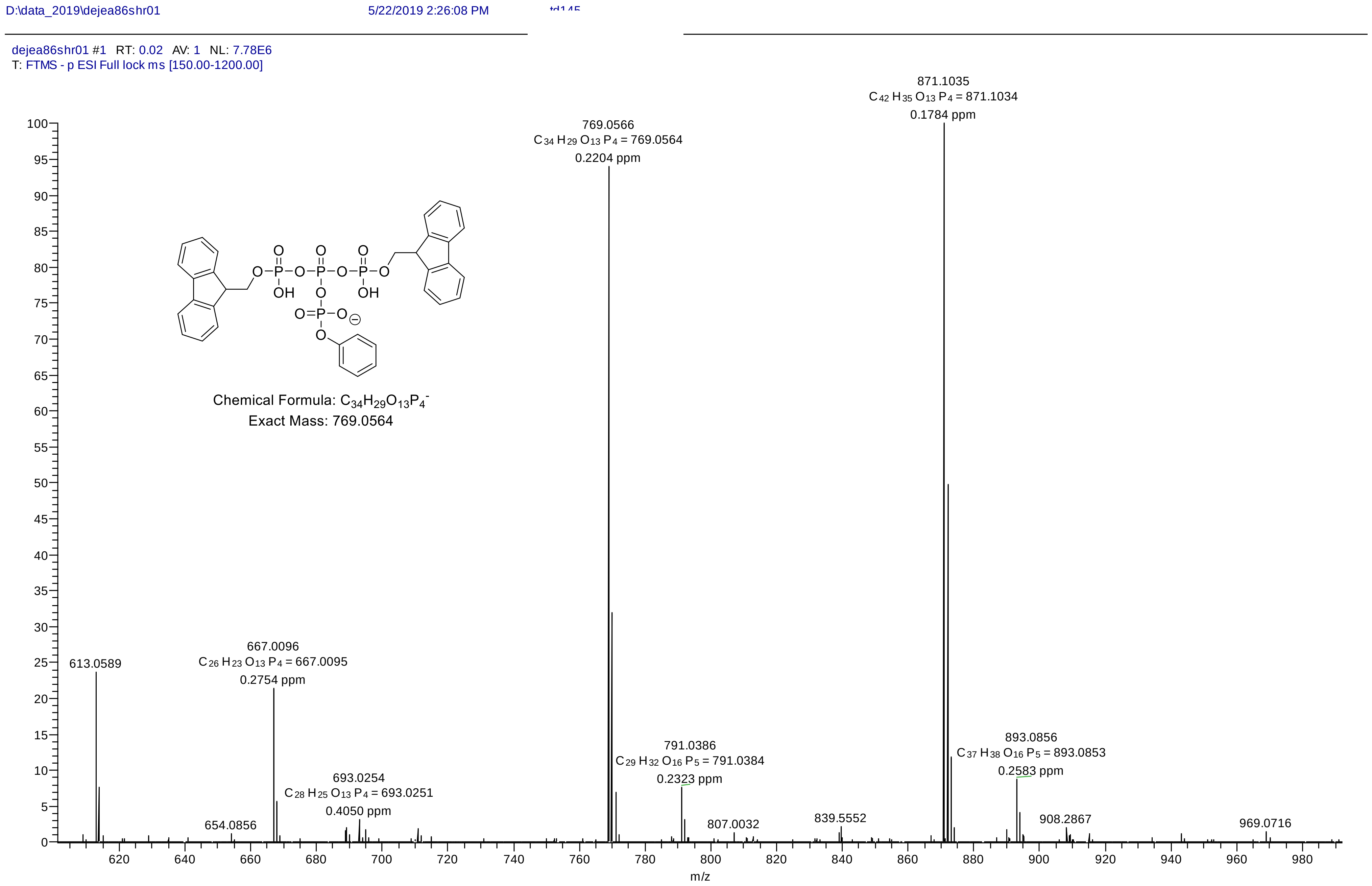

Supplementary Fig. 147│ **HRMS (ESI)**, compound **50**:

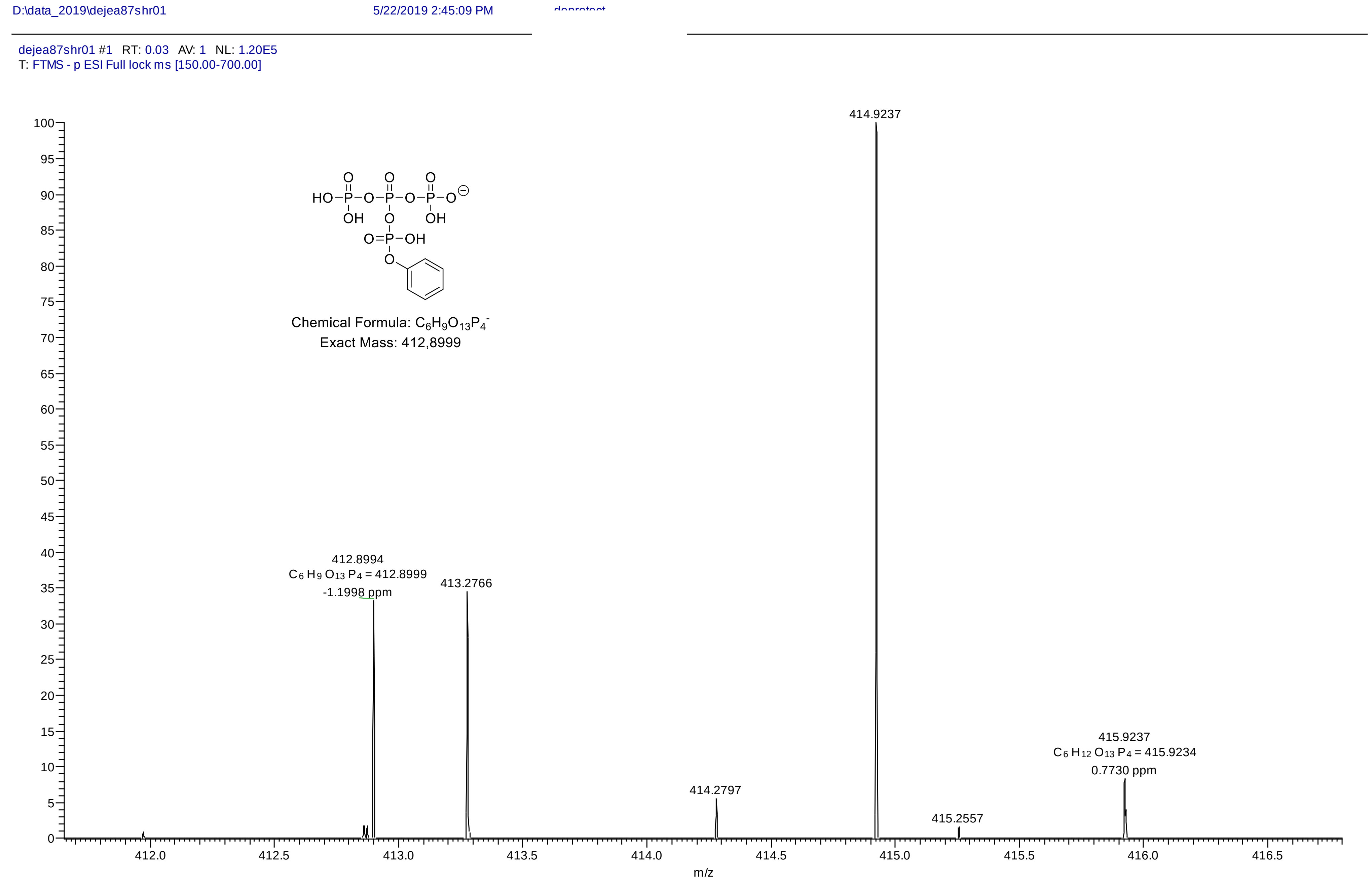

Supplementary Fig. 148│ HRMS (ESI), compound **47**:

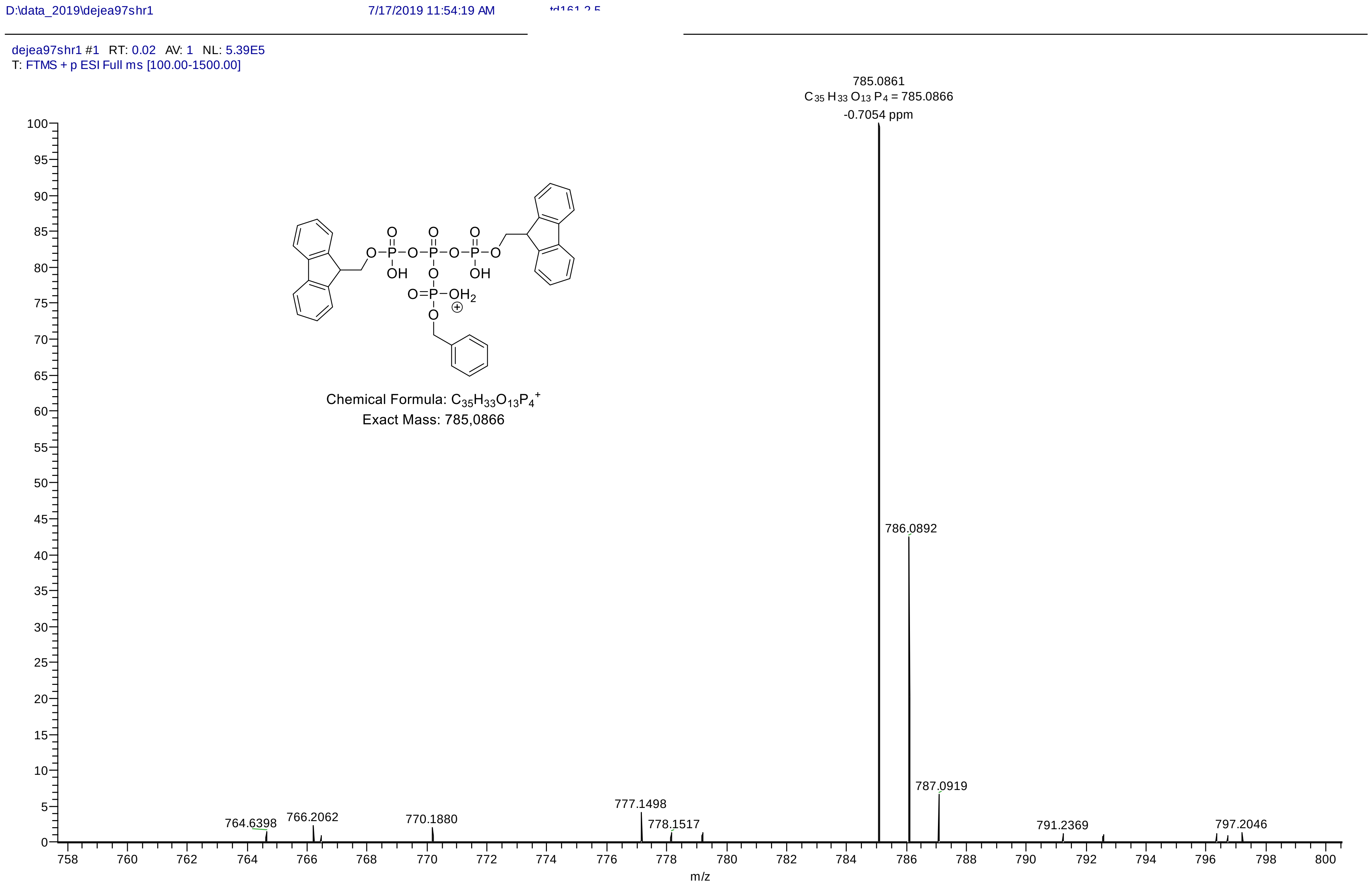

Supplementary Fig. 149│ HRMS (ESI), compound **48**:

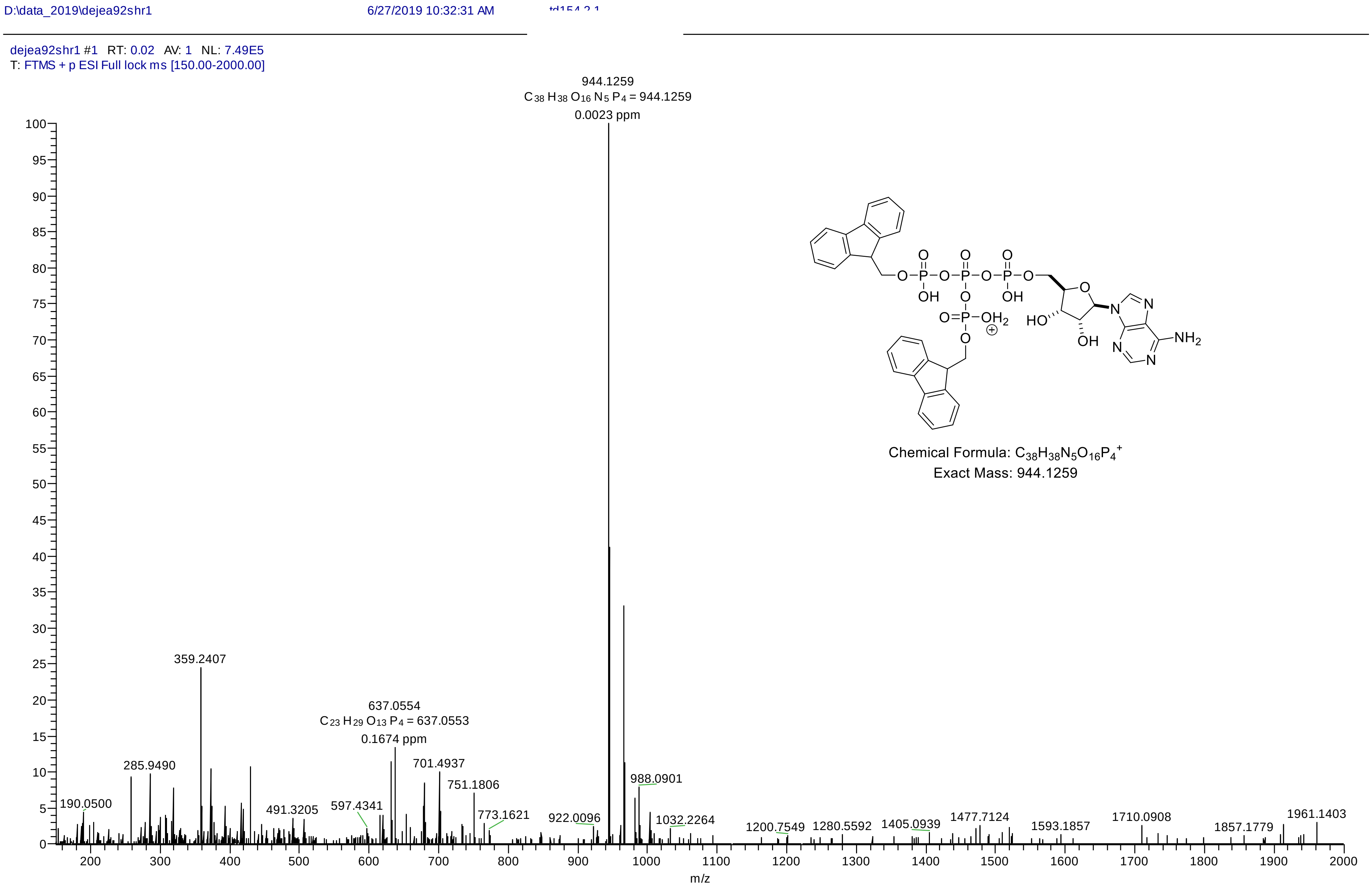

Chemical Formula: $C_{38}H_{38}N_5O_{16}P_4^+$
Exact Mass: 944.1259

Supplementary Fig. 150 | **HRMS (ESI)**, compound **52**:

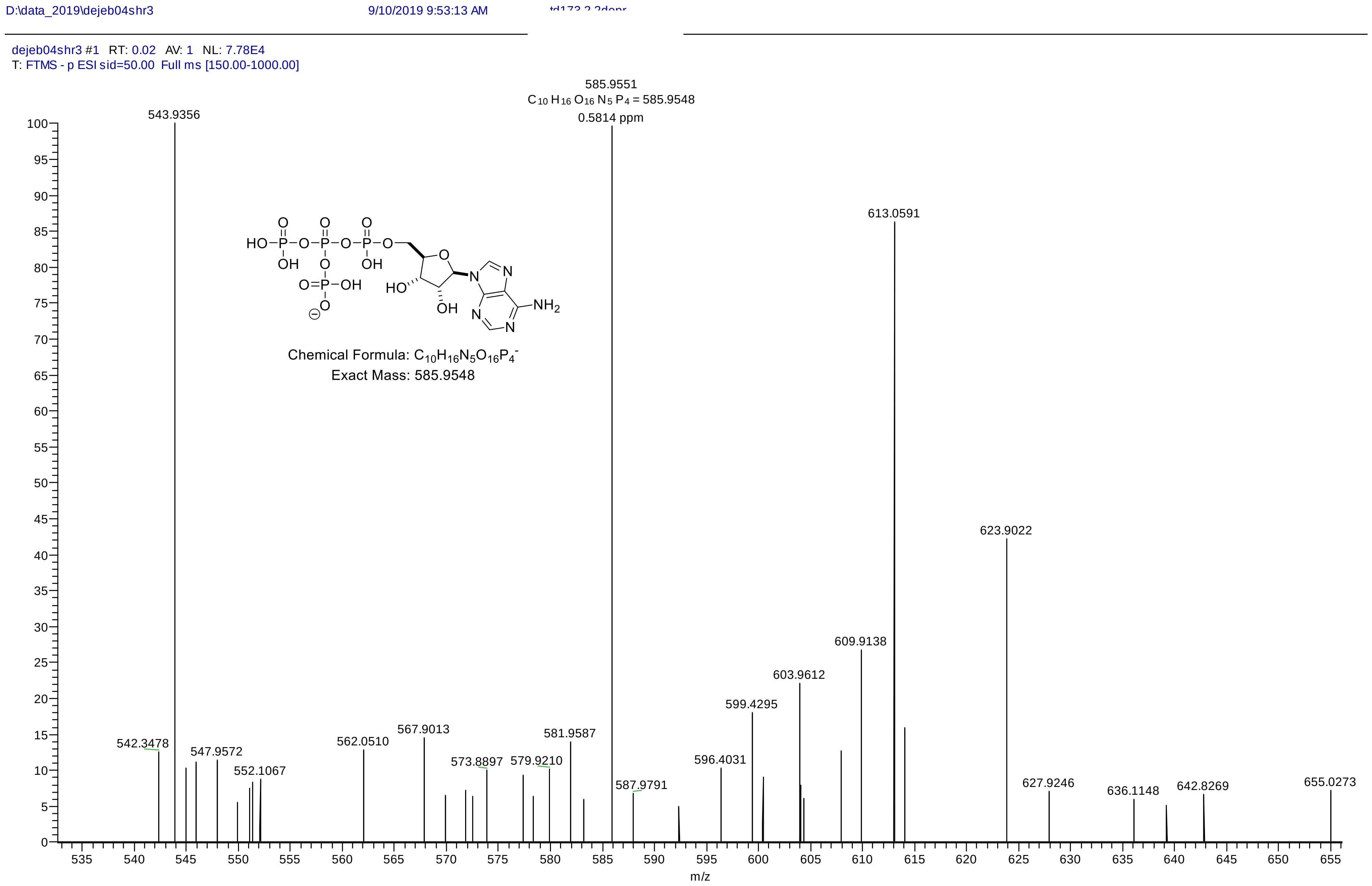

Supplementary Fig. 151 | **HRMS (ESI)**, compound **49**:

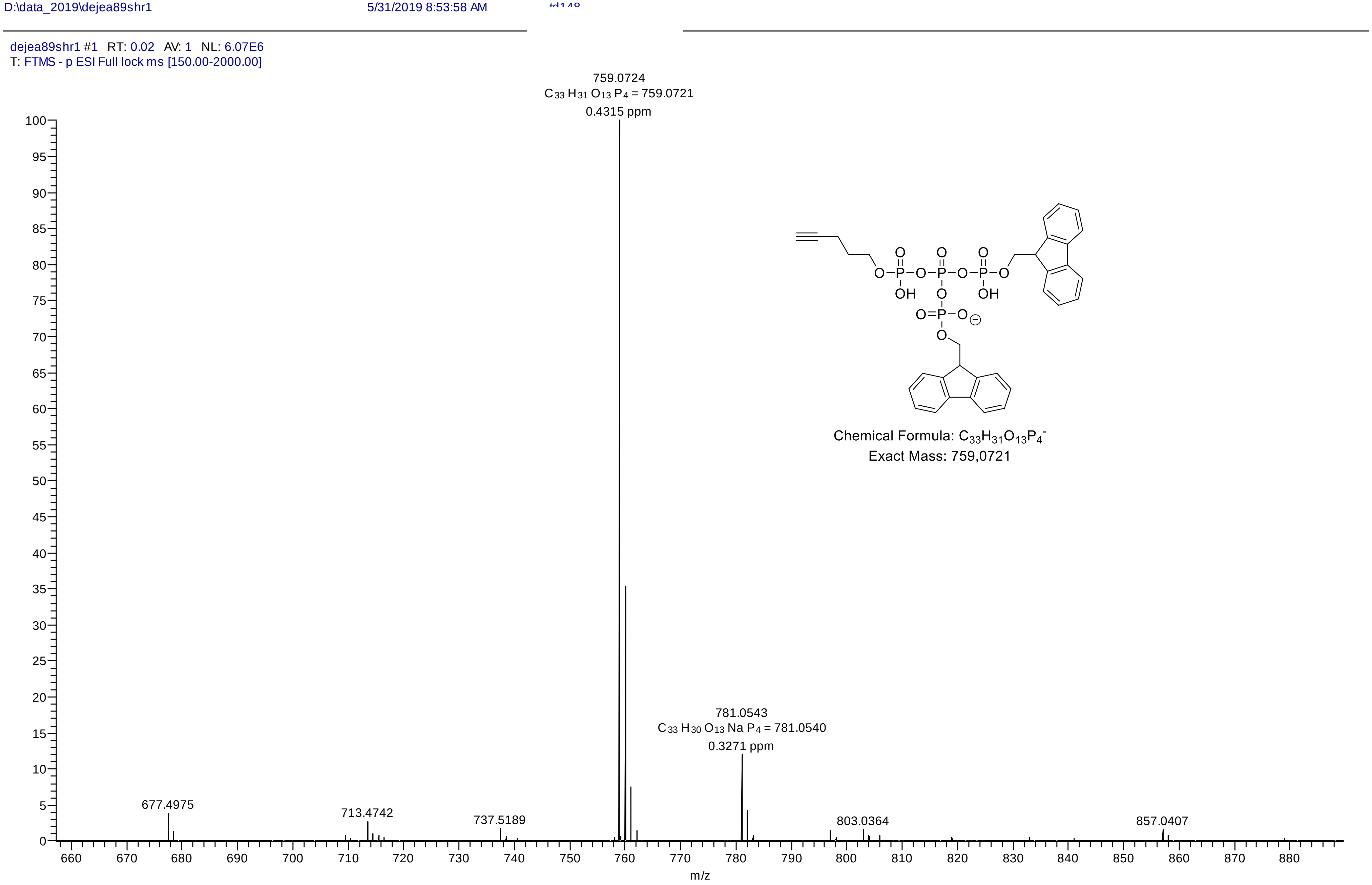

Supplementary Fig. 152│ HRMS (ESI), compound **55**:

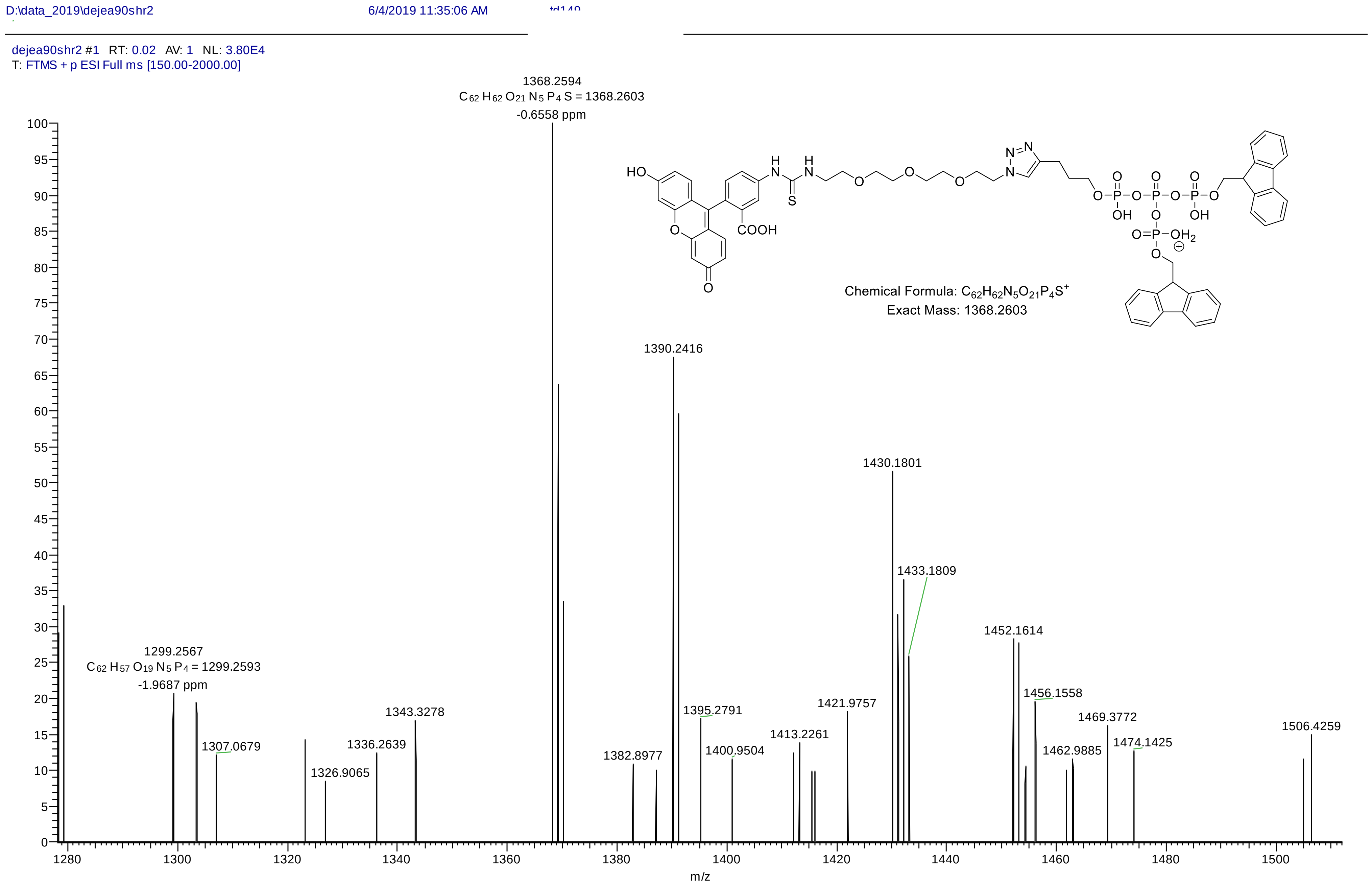

Supplementary Fig. 153 | HRMS (ESI), compound **56**:

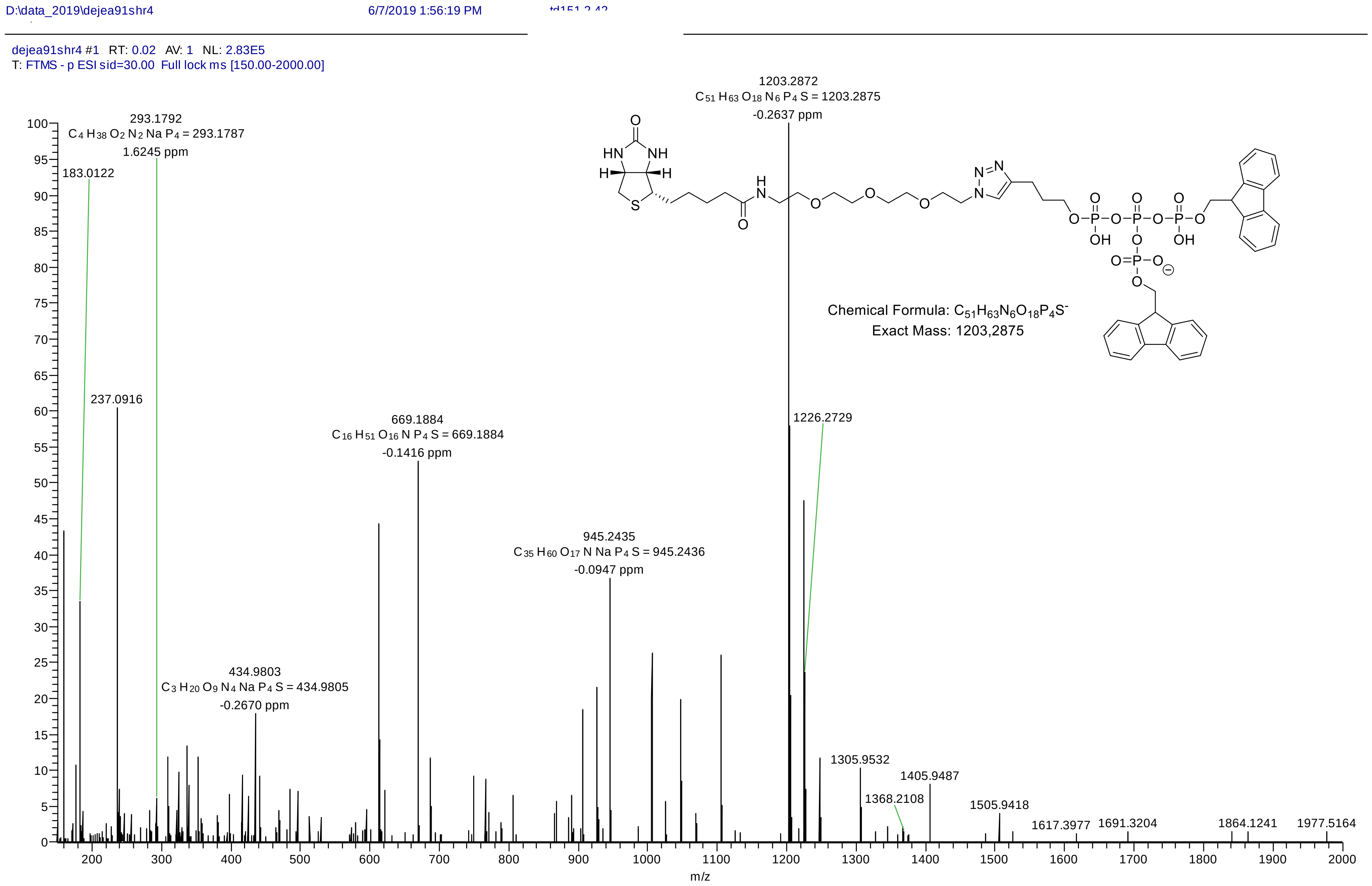

Raman spectra

Supplementary Fig. 154|**Raman**, [PPN]-Cl:

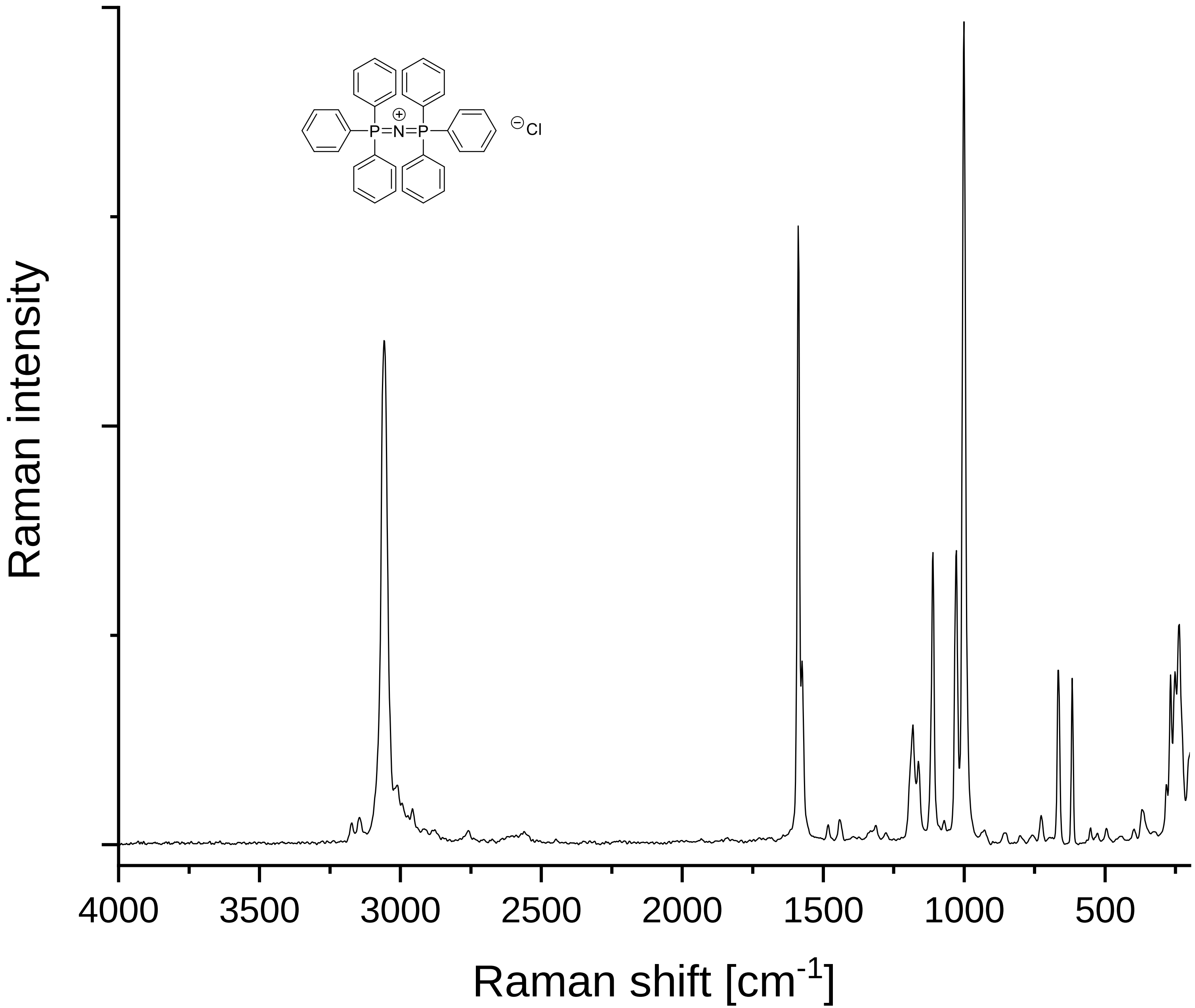

Supplementary Fig. 155│ Raman, compound **20**:

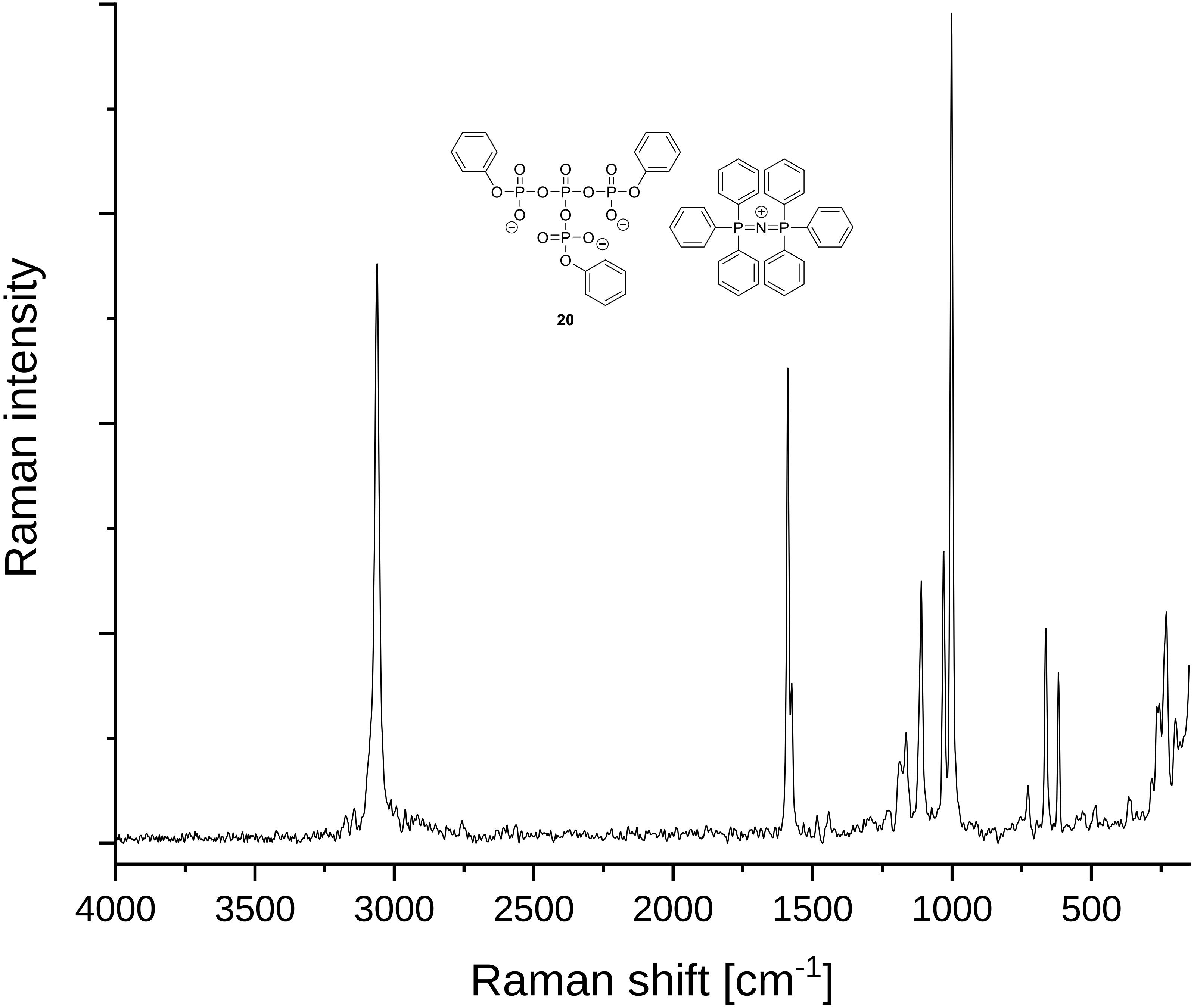

Supplementary Fig. 156│ **Raman**, compound **21**:

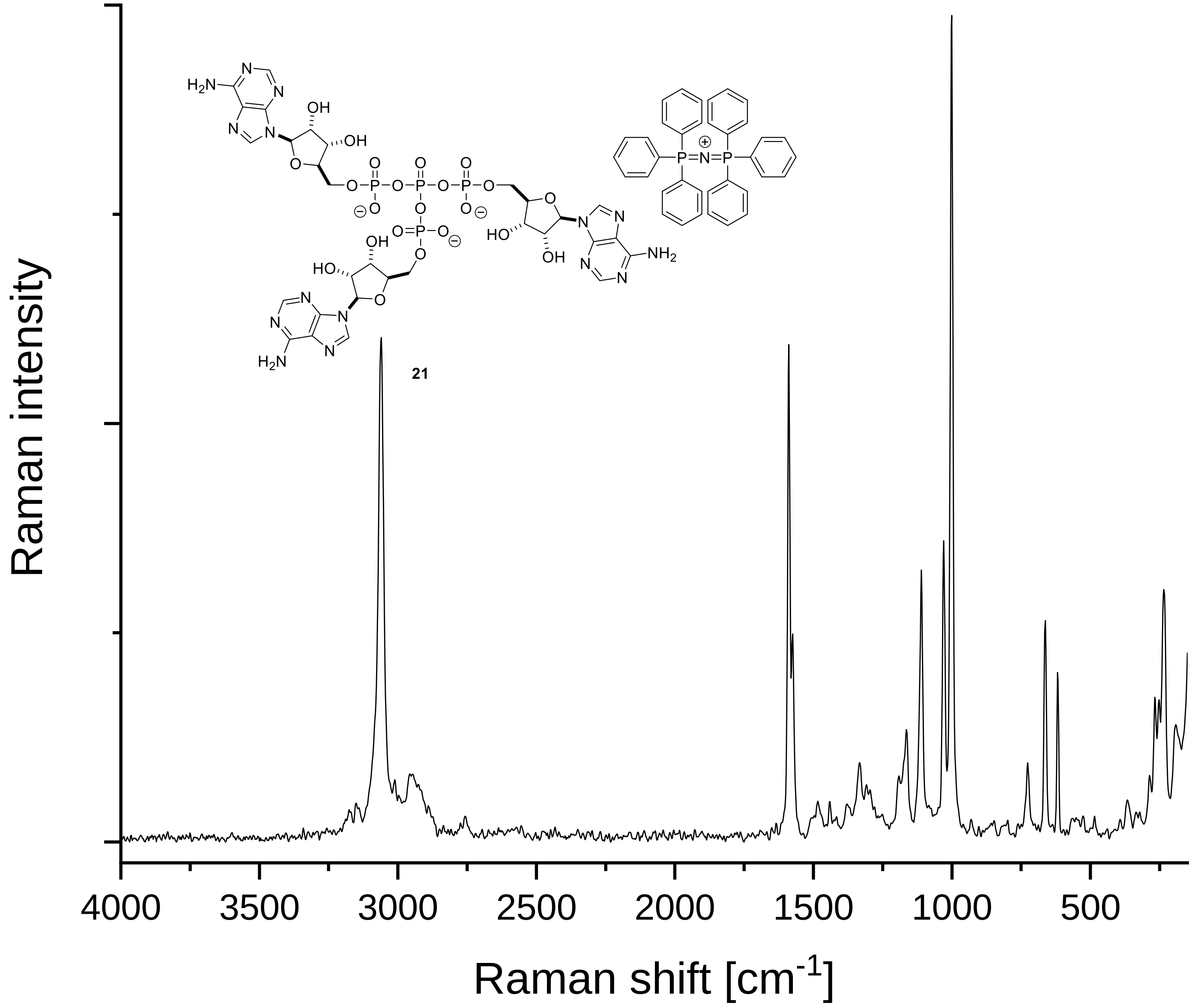

Supplementary Fig. 157│ **Raman**, compound **37**:

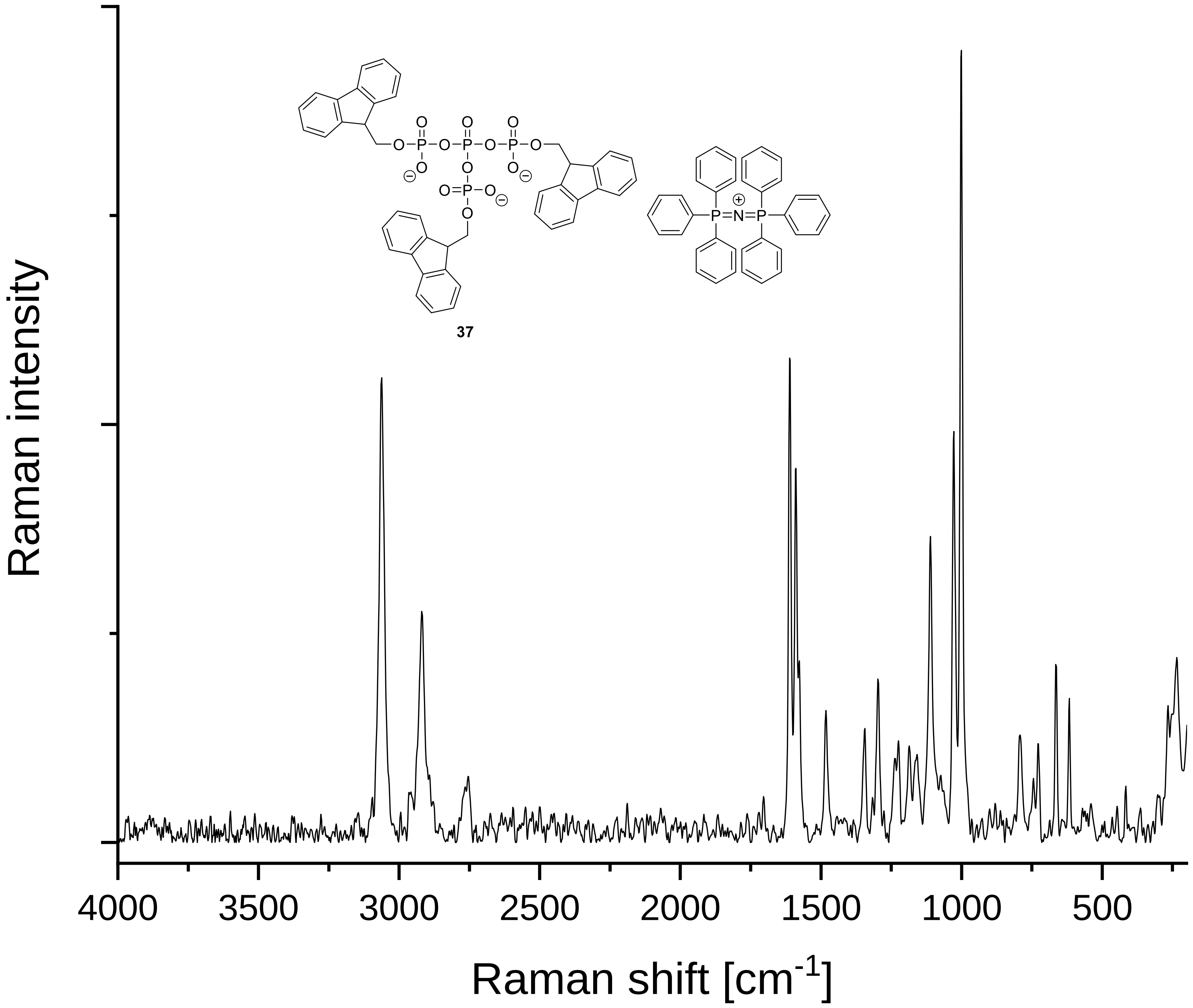

Supplementary Fig. 158│ **Raman**, compound **46**:

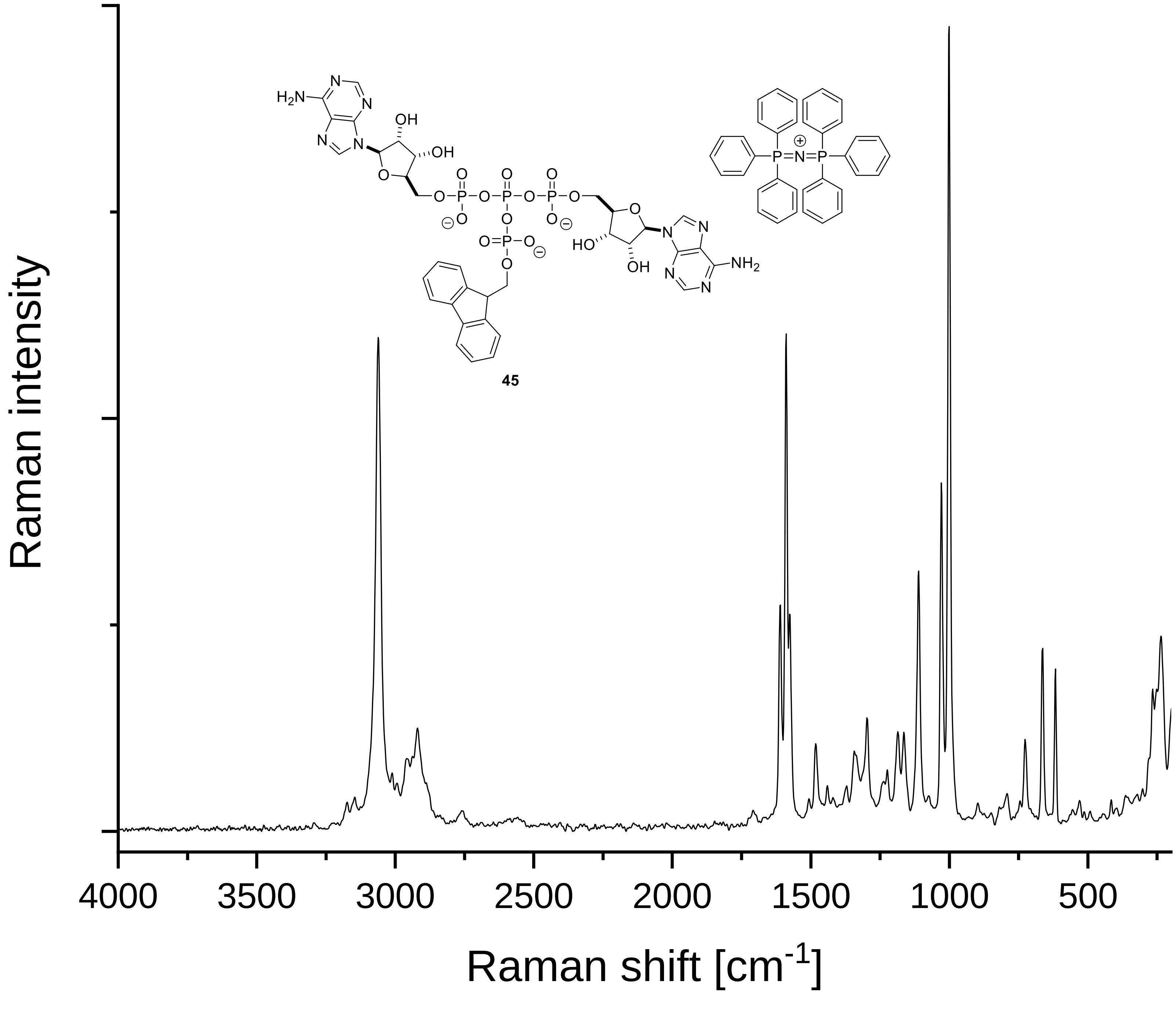

Supplementary References

[1] Method in accordance with: V. Mandala, D. M. Loh, S. M. Shepard, M. B. Geeson, I. V. Sergeyev, D. G. Nocera, C. C. Cummins, M. Hong, *J. Am. Chem. Soc.* **2020**, *142*, 18407-18421.

[2] O. Losito, Z. Szijgyarto, A. C. Resnick, A. Saiardi, *PLoS ONE* **2009**, *4*, 5580.

[3] P. Langen, E. Liss, *Biochem. Z.* **1958**, *330*, 455-466.

[4] M. W. Schmidt, K. K. Baldridge, J. A. Boatz, S. T. Elbert, M. S. Gordon, J. H. Jensen, S. Koseki, N. Matsunaga, K. A. Nguyen, S. Su, T. L. Windus, *J. Comp. Chem.* **1993**, *14*, 1347.

[5] Gaussian 09. Revision E.01, J. M. Frisch, G. W. Trucks, H. B. Schlegel, G. E. Scuseria, M. A. Robb, J. R. Cheeseman, G. Scalmani, V. Barone, G. A. Petersson, H. Nakatsuji, X. Li, M. Caricato, A. V. Marenich, J. Bloino, B. G. Janesko, R. Gomperts, B. Mennucci, H. P. Hratchian, J. V. Ortiz, A. F. Izmaylov, J. L. Sonnenberg, D. Williams-Young, F. Ding, F. Lipparini, F. Egidi, J. Goings, B. Peng, A. Petrone, T. Henderson, D. Ranasinghe, V. G. Zakrzewski, J. Gao, N. Rega, G. Zheng, W. Liang, M. Hada, M. Ehara, K. Toyota, R. Fukuda, J. Hasegawa, M. Ishida, T. Nakajima, Y. Honda, O. Kitao, H. Nakai, T. Vreven, K. Throssell, J. A. Montgomery Jr., J. E. Peralta, F. Ogliaro, M. J. Bearpark, J. J. Heyd, E. N. Brothers, K. N. Kudin, V. N. Staroverov, T. A. Keith, R. Kobayashi, M. Klene, C. Adamo, R. Cammi, J. W. Ochterski, R. L. Martin, K. Morokuma, O. Farkas, J. B. Foresman, D. J. Fox, Gaussian, Inc., Wallingford CT, 2009.

[6] S. Grimme, *J. Comput. Chem.* **2006**, *27*, 1787-1799.

[7] S. Grimme, *J. Chem. Phys.* **2006**, *124*, 034108-034115.

[8] F. Weigend, R. Ahlrichs, *Phys. Chem. Chem. Phys.* **2005**, *7*, 3297-3305.

[9] H. P. Hratchian, H. B. Schlegel, *J. Chem. Phys.*, **2004**, *120*, 9918-9924.

[10] K. K. Baldridge, *Development of original methodologies for reaction path following in GAMESS and GAUSSIAN*, PhD Thesis, **1988**.

[11] A. Klamt, G. Schüürmann, *J. Chem. Soc., Perkin Trans. 2* **1993**, *5*, 799-805.

[12] K. K. Baldrige, A. Klamt, *J. Chem. Phys.* **1997**, *106*, 6622-6633.

[13] M. D. Hanwell, D. E. Curtis, D. C. Lonie, T. Vandermeersch, E. Zurek, G. R. Hutchinson, *J. Cheminform.* **2012**, *4*, 17.

[14] WEBMO: Cundari, Thomas; Schmidt, J.R., www.webmo.net.

[15] M. Diez-Castellnou, A. Martinez, F. Mancin, *Adv. Phys. Org. Chem*. **2017**, *51*, 129-186.

[16] R. Abramson, K. K. Baldridge, *Mol. Phys.* **2012**, *110*, 2401-2412.

[17] R. Abramson, K. K. Baldridge, *J. Chem. Theory Comput.* **2013**, *9*, 1027-1035.

[18] J. Florián, A. Warshel, *J. Phys. Chem.* **1998**, *102*, 709-734.

[19] J. H. Kaplan, B. Forbush III, J. F. Hoffman, *Biochemistry* **1978**, *17*, 1929-1935.

[20] R. O. Schönleber, J. Bendig, V. Hagen, B. Giese, *Bioorg. Med. Chem.* **2002**, *10*, 97-101.

[21] J. C. Kern, M. Cancilla, D. Dooney, K. Kwasnjuk, R. Zhang, M. Beaumont, I. Figueroa, S. Hsieh, L. Liang, D. Tomazela et al., *J. Am. Chem. Soc.* **2016**, *138*, 1430-1445.

[22] Bruker, *SAINT*, *V8.40A*, Bruker AXS Inc., Madison, Wisconsin, USA.

[23] Bruker, *SADABS*, *2016/2:*, Bruker AXS Inc., Madison, Wisconsin, USA.

[24] G. M. Sheldrick, *Acta Cryst.* **2015**, *A71*, 3–8.

[25] G. M. Sheldrick, *Acta Cryst.* **2015**, *C71*, 3–8.

[26] C. R. Groom, I. J. Bruno, M. P. Lightfoot, S. C. Ward, *Acta Cryst.* **2016**, *B72*, 171–179.

[27] D. Kratzert, *FinalCif*, *V69*, https://www.xs3.uni-freiburg.de/research/finalcif.
